# Rate and Equilibrium Constants for the Addition of N-Heterocyclic Carbenes into Benzaldehydes: A Remarkable 2-Substituent Effect**

**DOI:** 10.1002/anie.201501840

**Published:** 2015-04-23

**Authors:** Christopher J Collett, Richard S Massey, James E Taylor, Oliver R Maguire, AnnMarie C O'Donoghue, Andrew D Smith

**Affiliations:** EaStCHEM, School of Chemistry, University of St AndrewsNorth Haugh, St Andrews, Fife, KY16 9ST (UK); Department of Chemistry, Durham UniversitySouth Road, Durham, DH1 3LE (UK)

**Keywords:** 2-substituent effect, kinetics, mechanistic studies, N-heterocyclic carbenes, organocatalysis

## Abstract

Rate and equilibrium constants for the reaction between N-aryl triazolium N-heterocyclic carbene (NHC) precatalysts and substituted benzaldehyde derivatives to form 3-(hydroxybenzyl)azolium adducts under both catalytic and stoichiometric conditions have been measured. Kinetic analysis and reaction profile fitting of both the forward and reverse reactions, plus onwards reaction to the Breslow intermediate, demonstrate the remarkable effect of the benzaldehyde 2-substituent in these reactions and provide insight into the chemoselectivity of cross-benzoin reactions.

Acyl anion equivalents generated from the reaction of N-heterocyclic carbenes (NHCs) with aldehydes are important catalytic intermediates that can undergo a range of carbon–carbon bond forming processes.[1] In this regard, NHC-catalyzed benzoin and Stetter reactions have been widely studied, with a number of efficient catalytic asymmetric methods available for both intra- and intermolecular reactions.[1,2] However, the development of cross-benzoin reactions has proven difficult in terms of the chemoselective formation of a single reaction product.[3] While efficient chemoselective NHC-catalyzed protocols for both intra- and intermolecular cross-benzoin reactions between aldehydes and ketones have been reported,[4] the reaction between two distinct aldehydes remains a significant synthetic challenge. As 2-substituted benzaldehydes are generally poor substrates for homo-benzoin reactions they have been widely utilized in cross-benzoin processes.[5] For example, Miller and Mennen reported the intramolecular cross-benzoin reaction between an arylaldehyde and a tethered aliphatic aldehyde to effect macrocyclization.[5b Connon and co-workers found that *N*-C_6_F_5_ triazolium NHC precatalyst **3** catalyzes intermolecular cross-benzoin reactions between 2-substituted benzaldehydes and aliphatic aldehydes with high levels of chemoselectivity (Scheme 1 a).[5c A selective cross-benzoin reaction between two benzaldehydes catalyzed by thiamine diphosphate dependent benzaldehyde lyase (BAL) was reported by Müller et al., with one 2-substituted benzaldehyde a prerequisite for good chemoselectivity.[6] Glorius and co-workers subsequently utilized this phenomenon in arylaldehyde cross-benzoin reactions using thiazolium NHC precatalyst **7** (Scheme 1 b).[5e,^7^] Gravel et al. have reported a triazolium NHC-catalyzed cross-benzoin process between benzaldehydes and alkyl aldehydes, with preliminary kinetic studies showing the reaction is at least first-order with respect to both aldehydes and that the chemoselectivity was determined at or after the C=C bond forming step.[5h

**Scheme 1:**
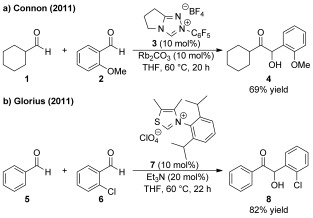
Cross-benzoin reactions using 2-substituted benzaldehydes.

Current explanations of the observed chemoselectivity in cross-benzoin reactions of arylaldehydes are usually simplistically based upon steric arguments. Previous to this investigation, it was commonly assumed that the presence of a 2-substituent decreases the rate of NHC addition into an arylaldehyde (Scheme 2).[8,9] The NHC **I** therefore preferably adds into aldehyde **II** to form least-hindered 3-(hydroxybenzyl)azolium adduct **IV**, which undergoes deprotonation to form Breslow intermediate **V**.[7,10] However, to account for the observed selectivity, intermediate **V** must now add into the more “hindered” 2-substituted benzaldehyde **VI**.[5c,[d,^6^] This steric argument is therefore inherently contradictory. There are currently no detailed mechanistic studies that offer insight into the rate of NHC additions into 2-substituted benzaldehydes, the effect of the *N*-aryl NHC substituent upon the rates of these processes, or the role of the 2-substituent in chemoselective cross-benzoin reactions of arylaldehydes. Building upon our previous mechanistic studies of NHC-catalyzed processes,[11] herein the remarkable effect of 2-arylaldehyde substitution upon equilibrium constants for 3-(hydroxybenzyl)azolium adduct formation is demonstrated. For the first time, individual rate constants for adduct formation have been determined under stoichiometric conditions and the effects of both aldehyde and *N*-aryl NHC substitution have been probed, with the results offering potential insight into the chemoselectivity of cross-benzoin processes.

**Scheme 2:**
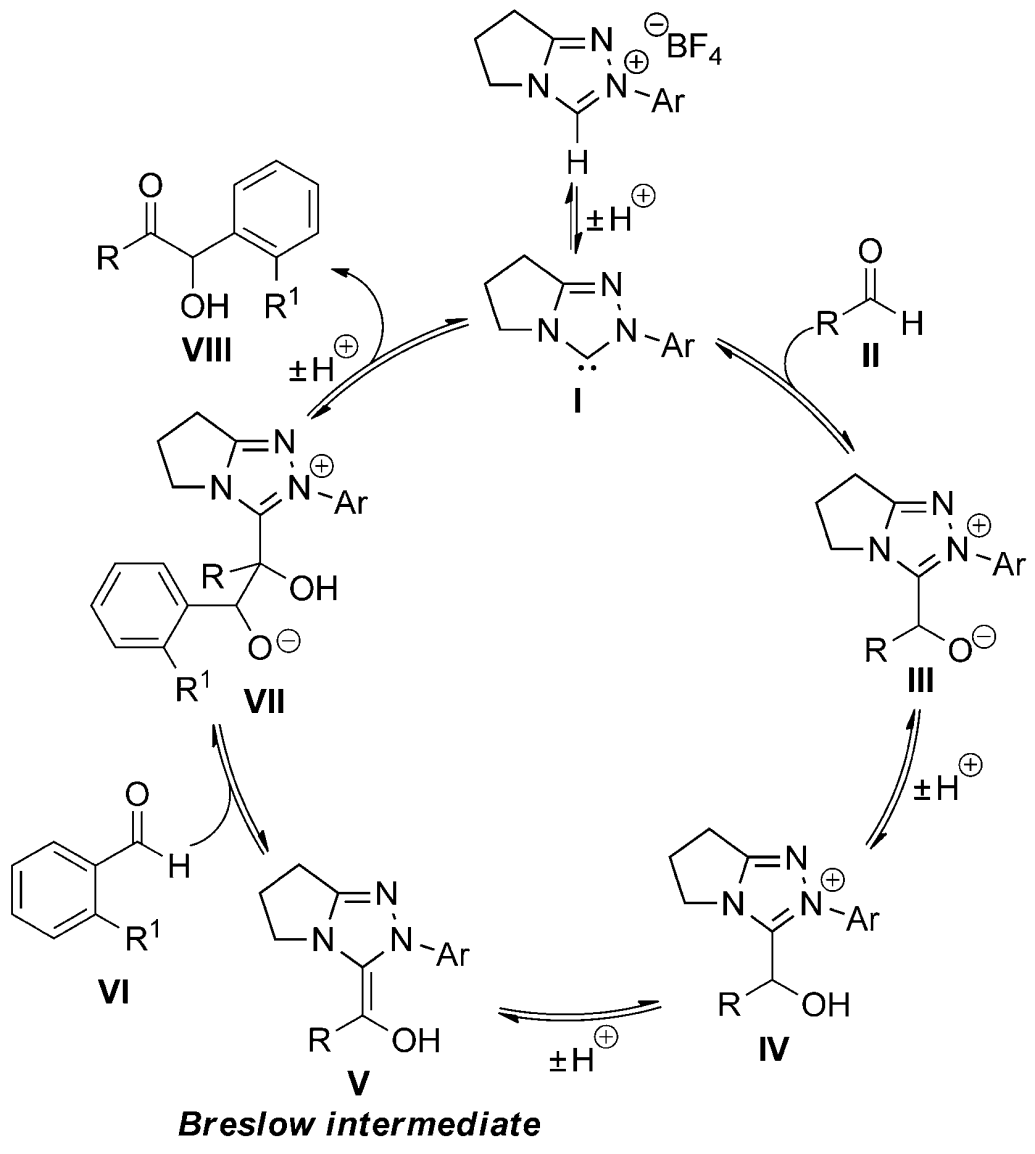
General mechanism for a cross-benzoin reaction.

First, the catalytic reactions between a range of substituted benzaldehydes (0.01 m) and NHC precatalyst **9**-**11** (0.002 m, 20 mol %) using Et_3_N (0.002 m, 20 mol %) in CD_2_Cl_2_ were monitored through in situ ^1^H NMR spectroscopy. Analysis of the resulting reaction profiles allowed equilibrium constants for adduct formation (*K*^exp^) to be determined (Table 1).[12] The results demonstrate the remarkable effect of having a heteroatom substituent in the 2-position of the benzaldehyde on *K*^exp^. For example, the reaction between NHC precatalyst **9** and 2-methoxybenzaldehyde **2** gave *K*^exp^=56 m^−1^ compared with *K*^exp^=3 m^−1^ for reaction with benzaldehyde **5** (Table 1, entries 1 and 2). As observed previously,[11] the 2,6-substituted NHC precatalysts **10** and **11** gave significantly higher *K*^exp^ values, although 2-methoxy aldehyde substitution again led to further prominent increases (Table 1, entries 3–6). The importance of the 2-heteroatom for this effect is demonstrated by reaction of NHC precatalyst **10** with 2-tolualdehyde **12**, which gives *K*^exp^=16 m^−1^ (Table 1, entry 7). The effect is not limited to 2-alkoxy substituents, as the reaction with 2-bromobenzaldehyde **14** gave *K*^exp^=332 m^−1^ whereas reaction with 4-bromobenzaldehyde **15** gave *K*^exp^=15 m^−1^ (Table 1, entries 9 and 10). The introduction of an additional heteroatom substituent in the 6-position further shifted the equilibrium in favor of adduct formation. For example, reaction of **10** with 2,6-difluorobenzaldehyde **17** gave *K*^exp^=785 m^−1^ whereas with 2-fluorobenzaldehyde **16**­ *K*^exp^=150 m^−1^ (Table 1, entries 11 and 12). The use of 2-pyridinecarboxaldehyde **18** also gave an equilibrium strongly in favor of the corresponding adduct, while reaction with 6-bromo-2-pyridinecarboxyaldehyde **19** exclusively gave 3-(hydroxybenzyl)azolium adduct **33** such that *K*^exp^ could not be measured (Table 1, entries 13 and 14). In most cases, the 3-(hydroxybenzyl)azolium salts could also be isolated from a stoichiometric reaction between the NHC precatalyst and the corresponding aldehyde in the presence of excess Et_3_N.

**Table 1:** Equilibrium constants *K* for 3-(hydroxybenzyl)azolium adduct formation.[a] 
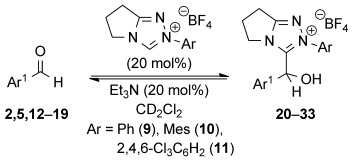

Entry	NHC	Ar^1^		*K*^exp^ [m^−1^]	Adduct	Yield [%][b]
1	**9**	Ph	**5**	3	**20**	3
2	**9**	2-MeOC_6_H_4_	**2**	56	**21**	24
3	**10**	Ph	**5**	31	**22**	9
4	**10**	2-MeOC_6_H_4_	**2**	143	**23**	28
5	**11**	Ph	**5**	39	**24**	–
6	**11**	2-MeOC_6_H_4_	**2**	601	**25**	69
7	**10**	2-MeC_6_H_4_	**12**	16	**26**	70
8	**10**	2-ROC_6_H_4_[c]	**13**	140	**27**	74
9	**10**	2-BrC_6_H_4_	**14**	332	**28**	63
10	**10**	4-BrC_6_H_4_	**15**	15	**29**	54
11	**10**	2-FC_6_H_4_	**16**	150	**30**	37
12	**10**	2,6-F_2_C_6_H_3_	**17**	785	**31**	71
13	**10**	2-pyridyl	**18**	303	**32**	–
14	**10**	6-Br-2-pyridyl	**19**	–	**33**	58

[a] Starting concentrations: aldehyde (0.01 m), NHC precatalyst (0.002 m), Et_3_N (0.002 m) in CD_2_Cl_2_ at 25 °C.

[b] Yield of isolated product from reaction between NHC precatalyst (1 equiv), aldehyde (1 equiv), and Et_3_N (2 equiv) in CH_2_Cl_2_.

[c] R=*E*-CH_2_CH=CHCOOEt.

To gain further insight into the dramatic effect of 2-heteroatom substitution, rate constants for 3-(hydroxybenzyl)azolium adduct formation were measured. First, the effect of the *N*-aryl NHC substituent was assessed, as no kinetic measurements have previously been made for triazolium-catalyzed benzoin or Stetter processes.[13,14] Reactions of aldehyde **13**, which is often employed as a model substrate for intramolecular Stetter reactions, were performed under pre-steady-state conditions using stoichiometric concentrations of NHC precatalysts in CD_3_OD with a Et_3_N:Et_3_N⋅HCl (2:1) buffer at 15 °C,[15] analogous to the conditions used by Leeper and White in their study of the thiazolium-catalyzed benzoin reaction.[13a Kinetic analysis of the reaction profiles obtained before significant product formation (<5 %) allowed pseudo second-order rate constants for 3-(hydroxybenzyl)azolium adduct formation (*k*_1_, m^−1^ s^−1^) and equilibrium constants (*K*^exp^, m^−1^) to be measured (Table 2).[16] Formation of the 3-(hydroxybenzyl)azolium adduct involves two distinct steps: the initial deprotonation of precatalyst by base and the subsequent reaction of the NHC with aldehyde. After the formation of adduct oxyanion, the base can be regenerated upon protonation at oxygen resulting in an overall pseudo second-order process under these experimental conditions.

**Table 2:** Measurement of rate and equilibrium constants for 3-(hydroxybenzyl)azolium adduct formation.[a] 
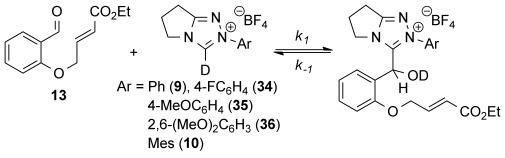

Entry	Ar	*k*_1_ [m^−1^ s^−1^]	*k*_−1_ [s^−1^]	*K*^exp^ [m^−1^]	*K*^fit^ [m^−1^][b]
1	Ph	1.52×10^−2^	4.76×10^−5^	319	394
2	4-FC_6_H_4_	4.89×10^−2^	9.45×10^−5^	383	433
3	4-MeOC_6_H_4_	1.28×10^−2^	3.09×10^−5^	414	555
4	2,6-(MeO)_2_C_6_H_3_	1.07×10^−2^	≤1.01×10^−7^	>1×10^5^	7034
5	Mes	3.85×10^−2^	1.25×10^−5^	3082	3414

[a] Starting concentrations: aldehyde **13** (0.04 m), NHC precatalyst (0.04 m) in CD_3_OD and 0.18 m Et_3_N:Et_3_N⋅HCl (2:1) buffer at 15 °C.

[b] Calculated through fitting of reaction profiles.

This is confirmed by the excellent fitting of reaction data to a kinetic expression describing a second-order reaction proceeding to a position of equilibrium.[12] The pseudo first-order rate constants for adduct dissociation (*k*_−1_, s^−1^) could also be calculated as *K*^exp^=*k*_1_/*k*_−1_. Additional estimates for *k*_1_ and *k*_−1_ were obtained from reaction profile fitting, with the values used to calculate the corresponding equilibrium constants (*K*^fit^). Pleasingly, the fitted values obtained are in good agreement with those obtained from kinetic analysis, with the largest discrepancy occurring for the reaction using NHC precatalyst **36** where adduct dissociation is negligible (Table 2, entry 4).[17]

Next, the reverse decay towards equilibrium was studied. Analysis of the ^1^H NMR reaction profiles for dissociation of the adducts of aldehyde **13** allowed rate and equilibrium constants of dissociation to be measured (*k*_d_, s^−1^ and *K*^diss^, m^−1^) and rate constants for association (*k*_a_, m^−1^ s^−1^) to be calculated (Table 3).[18] Although *k*_a_=*k*_1_ and *k*_d_=*k*_−1_ a distinction has been made to differentiate between the two methods of measurement. The dissociation analysis was not possible for the *N*-2,6-(MeO)_2_C_6_H_3_ adduct as the equilibrium lies so far towards the adduct that insufficient data could be obtained. Notably, the values for the equilibrium and rate constants measured from both the forward and reverse reactions at the same temperature are in good agreement with each other, showing that these methods can be used to give reliable measurements.

**Table 3:** Measurement of rate and equilibrium constants for 3-(hydroxybenzyl)azolium adduct dissociation.[a] 
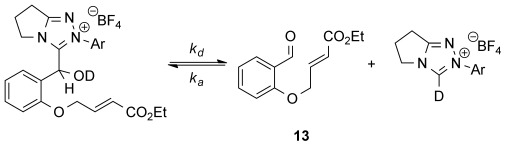

Entry	Ar	*k*_d_ [s^−1^]	*k*_a_ [m^−1^ s^−1^]	*K*^diss^ [m^−1^]	1/*K*^diss^ [m^−1^]
1	Ph	3.33×10^−4^	5.14×10^−2^	6.47×10^−3^	155
2	4-FC_6_H_4_	3.94×10^−4^	8.76×10^−2^	4.50×10^−3^	222
3	4-MeOC_6_H_4_	1.22×10^−4^	2.76×10^−2^	4.42×10^−3^	226
4	2,6-(MeO)_2_C_6_H_3_	ND	ND	–	–
5	Mes	5.34×10^−5^	9.90×10^−2^	5.40×10^−4^	1852

[a] Starting concentrations: 3-(hydroxybenzyl)azolium adduct (0.04 m) in CD_3_OD and 0.18 m Et_3_N:Et_3_N⋅HCl (2:1) buffer at 25 °C.

Comparing the *N*-aryl NHC precatalysts, the rate of adduct formation (*k*_1_ or *k*_a_) increases with more electron-withdrawing *N*-aryl substituents (4-F>4-H>4-MeO). This reflects the trend in p*K*_a_ for the NHC precatalysts (p*K*_a_ 4-F<4-H<4-MeO),[19] suggesting that the rate of 3-(hydroxybenzyl)azolium adduct formation is more influenced by the equilibrium for precatalyst deprotonation. However, *N*-Mes precatalyst **10** is an exception as its p*K*_a_ is similar to *N*-Ph precatalyst **9** (p*K*_a_ 17.7 and 17.8, respectively) but it reacts 2.5 times faster. This is postulated to be due to the orthogonal orientation of the mesityl substituent to the triazolium ring providing a more favorable approach of the aldehyde.[20] In all cases 3-(hydroxybenzyl)azolium adduct formation shows a degree of reversibility, however the kinetic data shows the rate of dissociation for the adduct derived from **13** and **10** is particularly slow, meaning that adduct formation is effectively irreversible in this case.[21]

Having established reliable methods for measuring equilibrium and rate constants for adduct formation this analysis was extended to look at substituted benzaldehydes (Table 4).[22] The reactions were performed using NHC precatalyst **9**, with comparable data obtained from both kinetic analysis and reaction profile fitting in all cases. The presence of a heteroatom in the aldehyde 2-position again has a marked effect, leading to significantly higher equilibrium constants for adduct formation.[23] The kinetic data gives an insight into the origin of this trend. For example, the rate of NHC addition into 2-methoxybenzaldehyde **2** is over 2.5 times faster than addition into benzaldehyde **5**, and over ten times as fast as addition into 4-methoxybenzaldehyde (Table 4, entries 1–3). A similar trend is seen comparing intramolecular Stetter substrate **13** with its 4-substituted analogue, demonstrating that the 2-substituent effect is not purely electronic in nature (Table 4, entries 4 and 5). In both cases the rate of the reverse process is also up to five times slower for 2-substituted benzaldehydes, reflecting the increased stability of these adducts. The importance of the heteroatom substituent is highlighted by the use of an analogue of **13** without the oxygen atom linker and 2- and 4-tolualdehyde, which all give equilibrium and rate constants comparable with benzaldehyde **5** (Table 4, entries 6–8). However, even in this case the rate of NHC addition into 2-tolualdehyde is nearly twice as fast as addition into 4-tolualdehyde (although the effect is smaller compared with heteroatom substituents).

**Table 4:** Measurement of rate and equilibrium constants using substituted benzaldehydes.[a] 
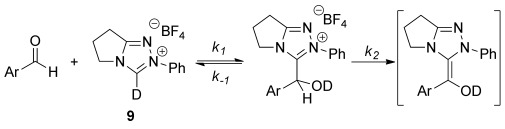

Entry	Ar	*k*_1_ [m^−1^ s^−1^]	*k*_−1_ [s^−1^]	*K*^exp^ [m^−1^]	*k*_2_ [s^−1^]
1	Ph	1.33×10^−2^	1.17×10^−3^	11.4	9.45×10^−6^
2	2-MeOC_6_H_4_	3.44×10^−2^	2.92×10^−4^	118	5.67×10^−6^
3	4-MeOC_6_H_4_	2.86×10^−3^	1.49×10^−3^	1.92	1.50×10^−6^
4	2-ROC_6_H_4_[c]	4.79×10^−2^	2.98×10^−4^	161	9.87×10^−6^
5[b]	4-ROC_6_H_4_[c]	3.58×10^−3^	1.00×10^−3^	3.58	–
6[b]	2-RCH_2_C_6_H_4_[c]	8.87×10^−3^	1.31×10^−3^	6.76	–
7	2-MeC_6_H_4_	1.15×10^−2^	7.82×10^−4^	14.7	3.59×10^−6^
8	4-MeC_6_H_4_	6.71×10^−3^	1.11×10^−3^	6.02	4.57×10^−6^

[a] Starting concentrations: aldehyde (0.04 m), NHC precatalyst **9** (0.04 m) in CD_3_OD and 0.18 m Et_3_N:Et_3_N⋅HCl (2:1) buffer at 25 °C.

[b] Reaction monitored at 15 °C.

[c] R=*E*-CH_2_CH=CHCOOEt.

Further kinetic analysis of the reaction profiles following the decreasing concentrations of the 3-(hydroxybenzyl)azolium adducts over time allow estimation of the pseudo first-order rate constants for deprotonation (*k*_2_, s^−1^) into the transiently formed Breslow intermediates (Table 4). The rate constants for deprotonation are of the same order of magnitude for all the aldehydes, including those containing a 2-substituent. Unlike the observed substituent effect on the first step (*k*_1_ and *K*), the observed order of reactivity on *k*_2_ reflects normal through-bond electronic effects on carbon acidity where electron-donating groups on the aldehyde decrease the rate of deprotonation. This is in agreement with our previous observations of normal electronic effects of the NHC *N*-aryl substituent on this deprotonation step.[11] Rate constants for deuterium exchange at the benzylic position of *O*-methylated 3-(hydroxybenzyl)azolium adducts were observed to decrease in the presence of electron-donating substituents (for example, 2-MeO) on the *N*-aryl ring.

The kinetic and equilibrium data of NHC addition into the benzaldehydes potentially offers insight into the observed chemoselectivity of cross-benzoin reactions. A representative cross-benzoin reaction between benzaldehyde **5** and 2-methoxybenzaldehyde **2** was performed using NHC precatalyst **3** (20 mol %) in CH_2_Cl_2_ at 45 °C (Scheme 3 a). The observed chemoselectivity is consistent with that previously reported,[5e with cross-product **37** favored and smaller amounts of homo-benzoin **38** and benzoin **39** also formed (Scheme 3 a). Similar product ratios were observed using NHC precatalyst **11**, although the conversion was lower (ca. 15 %). Monitoring the cross-reaction at 25 °C using NHC precatalyst **11** revealed a 10:1 mixture of 3-(hydroxybenzyl)azolium adducts **25**:**24** at equilibrium, again demonstrating a prominent 2-substituent effect in this system (Scheme 3 b). However, despite formation of adduct **25** being favored, cross-product **37** is derived from reaction of minor adduct **24**, indicating the chemoselectivity must be determined later in the reaction pathway.[24] This leads to three main possibilities for the origin of the observed chemoselectivity: 1) formation of the Breslow intermediate; 2) onwards reaction of the Breslow intermediate; 3) dissociation of the resulting tetrahedral adducts (Scheme 3 c).

**Scheme 3:**
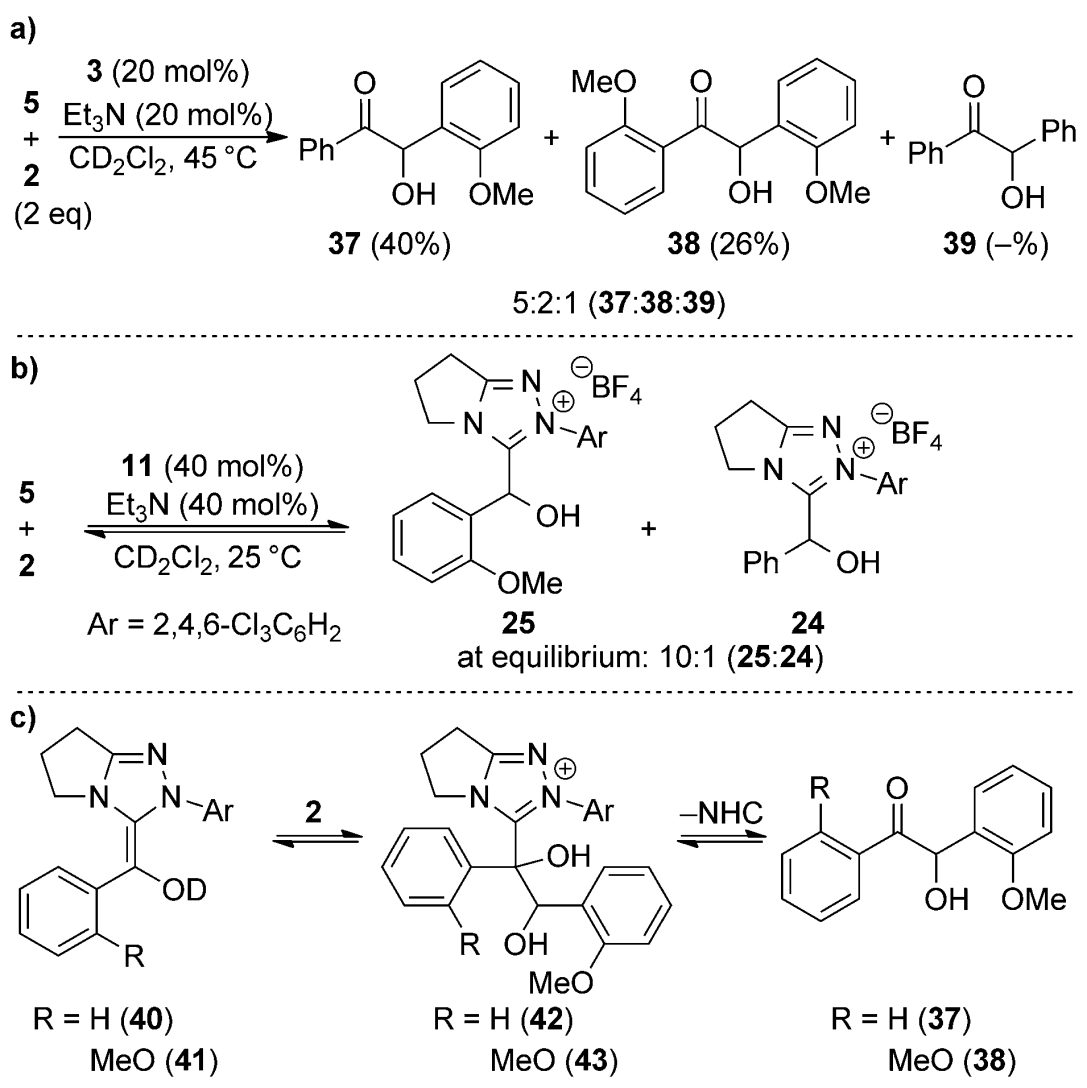
a) Cross-benzoin reaction; b) competition experiment; c) possible chemoselectivity determining steps in the cross-benzoin reaction.

The measured rate constants for Breslow intermediate formation show that 2-MeO substitution decreases *k*_2_ by a factor of about two relative to benzaldehyde **5** (Table 4, entries 1 and 2), however this does not outweigh the tenfold increase in equilibrium constant for adduct formation with a 2-MeO substituent and cannot account for the observed chemoselectivity. A difference in rate of the onwards reaction of the two Breslow intermediates **40** and **41** would account for the cross-benzoin selectivity. In both cases reaction with 2-methoxybenzaldehyde **2** will be comparatively fast over reaction with benzaldehyde **5** owing to the previously described 2-substituent effect. However, the increased steric hindrance around the nucleophilic carbon of **41** compared with **40** may decrease its relative rate of addition sufficiently to explain the formation of cross-benzoin **37**.[25]

Alternatively, NHC dissociation from tetrahedral intermediate **43** may be slow compared with **42**, again resulting in preferential formation of cross-product **37**. This would be consistent with the measured rate constants for dissociation (*k*_−1_) of the related 3-(hydroxybenzyl)azolium adducts in which a 4-fold difference was observed (Table 4, entries 1 and 2). However, accumulation of intermediates such as **42** and **43** have not been observed in any of our NMR experiments to date, or in earlier NMR studies by Leeper and White of the thiazolium-catalyzed benzoin reaction,[13a suggesting a faster rate of breakdown relative to the rate of formation from the relevant Breslow intermediate and aldehyde. Furthermore, monitoring reactions of NHC precatalyst **11** with either **37** or **38** gave about 10 % retro-benzoin products but no observable products consistent with formation of the corresponding tetrahedral adducts.[26] Additionally, a control experiment reacting NHC precatalyst **11** with acetophenone gave no observable products, suggesting that any NHC–ketone adducts formed rapidly dissociate. Therefore, it seems more likely that the chemoselectivity in cross-benzoin reactions is determined by the onwards reaction of the Breslow intermediate.

Although the increased rate of nucleophilic addition into benzaldehydes bearing a 2-heteroatom substituent is clearly evident, the origin of this phenomenon is unclear.[27] One possibility is that the presence of a lone pair on an atom in the 2-position changes the conformation of the aldehyde carbonyl such that it twists out of conjugation with the aryl ring. This ground state destabilization of aldehyde could result in increased reactivity towards nucleophiles. Alternatively increased product stability due to hydrogen bond formation between the 2-heteroatom substituent and the OH group of the 3-(hydroxybenzyl)azolium adducts could also contribute to the observed increase in both rate and equilibrium constants. These ground and product state effects could be realized in any nucleophilic addition to 2-substituted aldehydes of this type, including in the onward reaction of Breslow intermediates in cross-benzoin reactions.

In conclusion, measurements of equilibrium and rate constants for the reaction of triazolium NHC precatalysts with substituted benzaldehydes to give 3-(hydroxybenzyl)azolium adducts under both catalytic and stoichiometric conditions have been made. The results obtained from kinetic analysis and fitting data for both the forward and backwards processes show that nucleophilic addition into benzaldehydes bearing a 2-heteroatom substituent is particularly fast. By contrast, smaller substituent effects are observed on the rate of deprotonation of 3-(hydroxybenzyl)azolium adducts, which fall within the same order of magnitude regardless of aldehyde substitution. The results offer insight into the apparent inconsistency over the second aldehyde addition in cross-benzoin reactions, overturning the assumption that 2-substituted benzaldehydes are less reactive based upon steric arguments.
