# Supplementary material for: Rate and Equilibrium Constants for the Addition of N-Heterocyclic Carbenes into Benzaldehydes: A Remarkable 2-Substituent Effect
Source: Angew Chem Int Ed Engl. 2015 Apr 23;54(23):6887–92. doi: 10.1002/anie.201501840 (PMC4510784; doi:10.1002/anie.201501840)

Angewandte Chemie
Eine Zeitschrift der Gesellschaft Deutscher Chemiker

Supporting Information

German Edition: DOI:

Rate and Equilibrium Constants for the Addition of N-Heterocyclic Carbenes into Benzaldehydes: A Remarkable 2-Substituent Effect**

Christopher J. Collett, Richard S. Massey, James E. Taylor, Oliver R. Maguire, AnnMarie C. O'Donoghue, and Andrew D. Smith**

anie_201501840_sm_miscellaneous_information.pdf

Contents

General Information S2

Supplementary Tables S3

Starting Material Synthesis S7

Determination of Equilibrium Constants in CD_2Cl_2 (Table 1) S10

Isolation of 3-(Hydroxybenzyl)azolium Adducts **20-33** (Table 1) S25

Determination of Rate and Equilibrium Constants for 3-(Hydroxybenzyl)azolium Adduct Formation in CD_3OD (Table 2, Table S1) S33

Determination of Rate and Equilibrium Constants for 3-(Hydroxybenzyl)azolium Adduct Dissociation in CD_3OD (Table 3, Table S2) S55

Determination of Rate and Equilibrium Constants using Substituted Benzaldehydes in CD_3OD (Table 4, Table S4) S67

Determination of Rate and Equilibrium Constants for 3-(Hydroxybenzyl)azolium Adduct Formation in CD_3OD (Table S3) S88

Determination of Rate and Equilibrium Constants for 3-(Hydroxybenzyl)azolium Adduct Dissociation in CD_3OD (Table S5) S91

Cross-Benzoin Reaction (Scheme 3a) S94

Competition Experiment (Scheme 3b) S96

Retreatment Experiments S98

References S101

^{1}H and ^{13}C{^{1}H} NMR Spectra S102

General Information

Reactions were performed in flame-dried glassware under an Ar or N_2 atmosphere unless otherwise stated. Anhydrous CH_2Cl_2, Et_2O, THF and toluene were obtained from an MBraun SPS-800 system. Petrol is defined as petroleum ether 40–60 °C. All other solvents and commercial reagents were used as received without further purification unless otherwise stated. Room temperature (rt) refers to 20–25 °C.

Analytical thin layer chromatography was performed on pre-coated aluminium plates (Kieselgel 60 F_{254} silica). Plates were visualised under UV light (254 nm) or by staining with either phosphomolybdic acid or $KMnO_4$ followed by heating. Flash column chromatography was performed on Kieselgel 60 silica in the solvent system stated under a positive pressure of compressed air.

Melting points were recorded on an Electrothermal 9100 melting point apparatus.

Infrared spectra (ν_{max}) were recorded on a Shimadzu IRAffinity-1 Fourier transform IR spectrophotometer using either thin film or solid using Pike MIRacle ATR accessory. Analysis was carried out using Shimadzu IRsolution v1.50 and only characteristic peaks are reported.

NMR spectra were recorded on Oxford Varian Unity Inova 500 MHz, Varian Unity 300 MHz, Bruker Ultrashield 400 MHz, Bruker Avance 500 MHz, Bruker Ascend 500 MHz, Bruker Avance 400 MHz and Bruker Avance 300 MHz NMR spectrometers. In $CDCl_3$, ^1H and $^{13}C\{^1H\}$ NMR chemical shifts are reported relative to $CHCl_3$ at 7.27 ppm and 77.0 ppm, respectively. In d_4-methanol, ^1H and $^{13}C\{^1H\}$ NMR chemical shifts are reported relative to CHD_2OD at 3.31 and 49.0 ppm, respectively. In d_6-DMSO, ^1H and $^{13}C\{^1H\}$ NMR chemical shifts are reported relative to DMSO at 2.50 ppm and 39.5, respectively. In CD_2Cl_2, ^1H and $^{13}C\{^1H\}$ NMR chemical shifts are reported relative to CH_2Cl_2 at 5.31 ppm and 53.8 ppm, respectively. Coupling constants (*J*) are reported in Hertz (Hz). Multiplicities are indicated by: br s (broad singlet), s (singlet), d (doublet), t (triplet), q (quartet) and m (multiplet).

Mass spectrometry (m/z) data were acquired by electrospray ionisation (ES), electron impact (EI), chemical ionisation (CI), atmospheric pressure chemical ionisation (APCI) or nanospray ionisation (NSI) at the EPSRC UK National Mass Spectrometry Facility at Swansea University.

Supplementary Tables

Table S1 demonstrates that the same trends are observed at both 15 °C and 25 °C, indicating that the kinetic analysis is valid in cases where Stetter product formation is more significant. Consistent values are also obtained from kinetic analysis and reaction profile fitting in all cases. The largest discrepancy is observed for NHC precatalyst **36** where adduct dissociation is negligible.

Table S1 Measurement of rate and equilibrium constants for 3-(hydroxybenzyl)azolium adduct formation.[a]

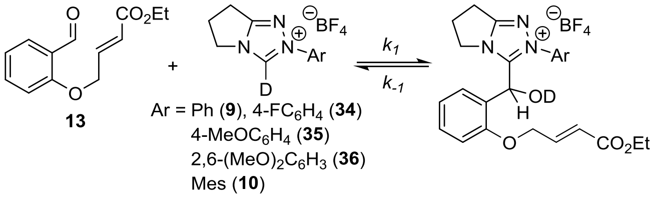

Entry	Ar	k_1 ($\text{M}^{-1}\text{s}^{-1}$)	k_{-1} (s^{-1})	K^{exp} (M^{-1})	k_1^{fit} ($\text{M}^{-1}\text{s}^{-1}$)[b]	k_{-1}^{fit} (s^{-1})[b]	K^{fit} (M^{-1})[b]
**Measured at 15 °C**							
1	Ph	1.52×10^{-2}	4.76×10^{-5}	319	2.29×10^{-2}	5.80×10^{-5}	394
2	$4\text{-FC}_6\text{H}_4$	4.89×10^{-2}	9.45×10^{-5}	383	5.54×10^{-2}	1.28×10^{-4}	433
3	$4\text{-MeOC}_6\text{H}_4$	1.28×10^{-2}	3.09×10^{-5}	414	1.37×10^{-2}	2.47×10^{-5}	555
4	$2,6\text{-(MeO)}_2\text{C}_6\text{H}_3$	1.07×10^{-2}	$\leq 1.01 \times 10^{-7}$	$>1 \times 10^{5}$	9.19×10^{-3}	1.31×10^{-7}	7034
5	Mes	3.85×10^{-2}	1.25×10^{-5}	3082	3.79×10^{-2}	1.11×10^{-5}	3414
**Measured at 25 °C**							
6	Ph	4.79×10^{-2}	2.98×10^{-4}	161	4.56×10^{-2}	2.94×10^{-4}	155
7	$4\text{-FC}_6\text{H}_4$	1.00×10^{-1}	5.47×10^{-4}	183	1.11×10^{-1}	5.27×10^{-4}	210
8	$4\text{-MeOC}_6\text{H}_4$	2.83×10^{-2}	1.24×10^{-4}	228	2.74×10^{-2}	1.33×10^{-4}	207
9	$2,6\text{-(MeO)}_2\text{C}_6\text{H}_3$	2.04×10^{-2}	$\leq 1.39 \times 10^{-6}$	>14000	–	–	–
10	Mes	7.26×10^{-2}	5.26×10^{-5}	1380	7.50×10^{-2}	5.24×10^{-5}	1431

[a] Starting concentrations: aldehyde **13** (0.04 M), NHC precatalyst (0.04 M) in CD_3OD and 0.18 M $Et_3N:Et_3N\cdot HCl$ (2:1) buffer. [b] Calculated through fitting of reaction profiles.

Table S2 shows that consistent data for adduct dissociation is obtained from both kinetic analysis and reaction profile fitting data. Importantly, the values for equilibrium and rate constants measured from both the forward and reverse reactions are in good agreement with each other, showing that these methods can be used to give reliable measurements.

Table S2 Measurement of rate and equilibrium constants for 3-(hydroxybenzyl)azolium adduct dissociation.[a]

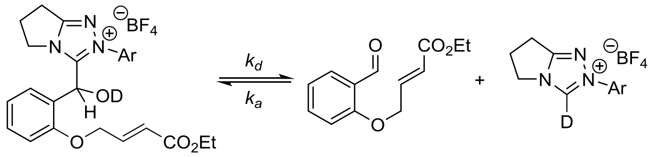

13

Entry	Ar	k_d (s^{-1})	k_a ($M^{-1}s^{-1}$)	K^{diss} (M)	$1/K^{diss}$ (M^{-1})
1	Ph	3.33×10^{-4}	5.14×10^{-2}	6.47×10^{-3}	155
2	4-FC_6H_4	3.94×10^{-4}	8.76×10^{-2}	4.50×10^{-3}	222
3	4-$MeOC_6H_4$	1.22×10^{-4}	2.76×10^{-2}	4.42×10^{-3}	226
4	2,6-$(MeO)_2C_6H_3$	ND	ND	–	–
5	Mes	5.34×10^{-5}	9.90×10^{-2}	5.40×10^{-4}	1852
**Reaction profile fitting data[b]**					
6	Ph	3.72×10^{-4}	5.29×10^{-2}	–	142
7	4-FC_6H_4	5.11×10^{-4}	9.97×10^{-2}	–	195
8	4-$MeOC_6H_4$	1.26×10^{-4}	2.87×10^{-2}	–	227
9	2,6-$(MeO)_2C_6H_3$	2.59×10^{-6}	9.80×10^{-2}	–	3788
10	Mes	5.18×10^{-5}	7.92×10^{-2}	–	1529

[a] Starting concentrations: 3-(hydroxybenzyl)azolium adduct (0.04 M) in CD_3OD and 0.18 M Et_3N:$Et_3N \cdot HCl$ (2:1) buffer at 25 °C. [b] Calculated through fitting of reaction profiles.

The reactions of benzaldehyde **5** with a range of NHC precatalysts show the same trends as for aldehyde **13**, suggesting that the effect of the *N*-aryl substituent is independent of aldehyde substitution (Table S3).

Table S3 Measurement of rate and equilibrium constants of NHC precatalyst addition into benzaldehyde **5**.[a]

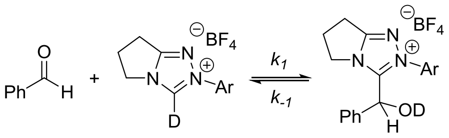

5

Entry	Ar	k_1 (M^{-1}s^{-1})	k_{-1} (s^{-1})	K^{exp} (M^{-1})	k_1^{fit} (M^{-1}s^{-1})[b]	k_{-1}^{fit} (s^{-1})[b]	K^{fit} (M^{-1})[b]
1	Ph	1.33×10^{-2}	1.17×10^{-3}	11	1.16×10^{-2}	1.01×10^{-3}	12
2	4-FC_6H_4	2.83×10^{-2}	1.83×10^{-3}	16	2.59×10^{-2}	1.65×10^{-3}	16
3	4-$MeOC_6H_4$	9.11×10^{-3}	5.36×10^{-4}	17	7.92×10^{-3}	4.42×10^{-4}	18
4	Mes	3.29×10^{-2}	2.06×10^{-4}	160	2.76×10^{-2}	1.64×10^{-4}	168

[a] Starting concentrations: benzaldehyde **5** (0.3 M), NHC precatalyst (0.3 M) in CD_3OD and 0.18 M Et_3N:Et_3N·HCl (2:1) buffer at 25 °C. [b] Calculated through fitting of reaction profiles.

Table S4 shows data from the reaction of NHC precatalyst **9** with a range of substituted benzaldehydes obtained from both kinetic analysis and reaction profile fitting.

Table S4 Measurement of rate and equilibrium constants using substituted benzaldehydes.[a]

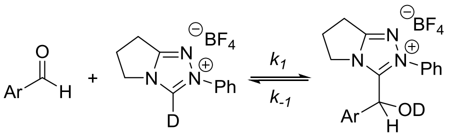

9

Entry	Ar	k_1 (M^{-1}s^{-1})	k_{-1} (s^{-1})	K (M^{-1})	k_1^{fit} (M^{-1}s^{-1})[b]	k_{-1}^{fit} (s^{-1})[b]	K^{fit} (M^{-1})[b]
1	Ph	1.33×10^{-2}	1.17×10^{-3}	11.4	1.16×10^{-2}	1.01×10^{-3}	11.5
2	2-$MeOC_6H_4$	3.44×10^{-2}	2.92×10^{-4}	118	2.78×10^{-2}	2.26×10^{-4}	123
3	4-$MeOC_6H_4$	2.86×10^{-3}	1.49×10^{-3}	1.92	2.59×10^{-3}	1.32×10^{-3}	1.96
4	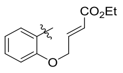	4.79×10^{-2}	2.98×10^{-4}	161	4.56×10^{-2}	2.94×10^{-4}	155
5[c]	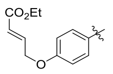	3.58×10^{-3}	1.00×10^{-3}	3.58	–	–	–
6[c]	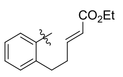	8.87×10^{-3}	1.31×10^{-3}	6.76	–	–	–
7	2-MeC_6H_4	1.15×10^{-2}	7.82×10^{-4}	14.7	1.07×10^{-2}	7.03×10^{-4}	15.2
8	4-MeC_6H_4	6.71×10^{-3}	1.11×10^{-3}	6.02	6.22×10^{-3}	1.01×10^{-3}	6.16

[a] Starting concentrations: aldehyde (0.04 M), NHC precatalyst **9** (0.04 M) in CD_3OD and 0.18 M Et_3N:Et_3N·HCl (2:1) buffer at 25 °C. [b] Calculated through fitting of reaction profiles. [c] Reaction monitored at 15 °C.

Rate and equilibrium constants from substituted benzaldehyde 3-(hydroxybenzyl)azolium adduct dissociations were also measured in some cases, with data obtained comparable to that from the corresponding forward process (Table S5).

Table S5 Measurement of rate and equilibrium constants for 3-(hydroxybenzyl)azolium adduct dissociation[a]

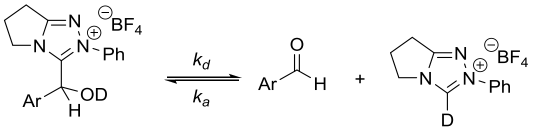

9

Entry	Ar	k_d (s^{-1})	k_a ($M^{-1}s^{-1}$)	K^{diss} (M)	$1/K^{diss}$ (M^{-1})
1	Ph	1.21×10^{-3}	1.61×10^{-2}	7.50×10^{-2}	13.3
2	2-$MeOC_6H_4$	2.76×10^{-4}	3.73×10^{-2}	7.40×10^{-3}	135
3	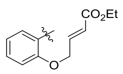	3.33×10^{-4}	5.14×10^{-2}	6.47×10^{-3}	155
4	4-MeC_6H_4	1.27×10^{-3}	9.07×10^{-3}	1.40×10^{-1}	7.1

[a] Starting concentrations: 3-(hydroxybenzyl)azolium adduct (0.04 M) in CD_3OD and 0.18 M Et_3N:$Et_3N \cdot HCl$ (2:1) buffer at 25 °C.

Starting Material Synthesis

(*E*)-Ethyl 4-(2-formylphenoxy)but-2-enoate 13

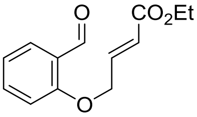

A solution of salicylaldehyde (6.10 g, 50.0 mmol), ethyl 4-bromocrotonate (13.3 g, 55.0 mmol) and K_2CO_3 (8.65 g, 12.5 mmol) in dry DMF (5 mL) was stirred for 2 h at rt. The crude mixture was filtered and Et_2O (100 mL) and H_2O (100 mL) were added to the filtrate. The aqueous layer was extracted with Et_2O (2 × 50 mL) and the combined organic phases were dried ($MgSO_4$) and concentrated *in vacuo* to give the crude product which after recrystallisation (EtOAc) afforded the title compound **13** as a white solid (4.60 g, 40%), with data are in accordance with the literature.[1] mp 68-70 °C; {lit.[1] mp 66-69 °C}; ^{1}H NMR (400 MHz, CD_3OD) δ_H: 1.29 (3H, t, *J* 7.1, CH_2CH_3), 4.21 (2H, q, *J* 7.1, CH_2CH_3), 4.92 (2H, dd, *J* 4.1, 2.1, CH_2CH), 6.22 (1H, dt, *J* 15.8, 2.1, CH_2CHCH), 7.09-7.17 (3H, m, $CHCHCO_2Et$ and 4,6-Ar*H*), 7.62 (1H, ddd, *J* 8.5, 7.3, 1.8, 5-Ar*H*), 7.80 (1H, dd, *J* 7.7, 1.8, 3-Ar*H*), 10.56 (1H, d, *J* 0.7, C(O)*H*).

(*E*)-Ethyl 4-(4-formylphenoxy)but-2-enoate S1

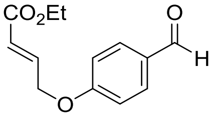

A solution of 4-hydroxybenzaldehyde (1.22 g, 10.0 mmol), ethyl 4-bromocrotonate (2.41 g, 12.5 mmol) and K_2CO_3 (1.38 g, 10.0 mmol), in dry DMF (5 mL), was stirred for 2 h at rt. The crude mixture was filtered and Et_2O (100 mL) and H_2O (100 mL) were added to the filtrate. The aqueous layer was extracted with Et_2O (2 × 50 mL) and the combined organic phases were dried ($MgSO_4$) and concentrated *in vacuo* to give the crude product, which was purified by recrystallisation (Et_2O) to yield the title compound **S1** as a white solid (901 mg, 39%). mp 59-62 °C; ν_{max} (neat) 1717 (C=O), 1690 (C-O), 1600 (C-O), 1512 (C=C), 1483 (C=C), 1388 (C-O), 1215, 1159; ^{1}H NMR (300 MHz, $CDCl_3$) δ_H: 1.29 (3H, t, *J* 7.1, CH_3), 4.21 (2H, q, *J* 7.1, CH_2CH_3), 4.78 (2H, dd, *J* 4.1, 2.1, CH_2CH), 6.17 (1H, dt, *J* 15.8, 2.1, CH_2CHCH), 6.98-7.10 (3H, m, CH_2CHCH and 2,6-Ar*H*), 7.81-7.86 (2H, m, 3,5-Ar*H*), 9.88 (1H, s, C(O)*H*); ^{13}C{^{1}H} NMR (75 MHz, $CDCl_3$) δ_C: 14.3 (CH_3), 60.8 (CH_2CH_3), 66.7 (OCH_2), 115.0 (2,6-ArCH), 122.7 (CH_2CHCH), 130.5 (4-Ar*C*), 132.1 (3,5-Ar*C*H), 141.2 (CH_2CHCH), 163.0 (1-Ar*C*), 165.9 (*C*(O)OEt), 190.8 (*C*(O)H); *m/z* (NSI^+) 235 ($[M]^+$, 100%); HRMS (NSI^+) $C_{13}H_{15}O_4$ $[M]^+$ found 235.0958, requires 235.0965 (-2.9 ppm).

(*E*)-Ethyl 5-(2-formylphenyl)pent-2-enoate S3

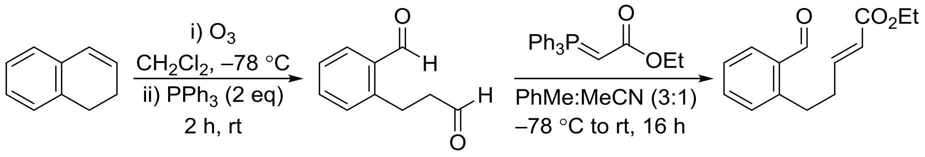

A solution of 1,2-dihydronapthalene (1.00 g, 7.68 mmol) in CH_2Cl_2 (300 mL) was cooled to -78 °C. A stream of O_3 was passed through the solution until a blue colour persisted, followed by a steam of oxygen until the solution was colourless. Triphenylphosphine (4.03 g, 15.4 mmol) was added and the solution was stirred for 2 h rt. Concentration *in vacuo*, followed by silica chromatography (30:70 petrol:diethyl ether) gave 2-(3-oxopropyl)benzaldehyde **S2** as a colourless oil (1.02 g, 82%), with data are in accordance with the literature.[2] ^{1}H NMR (300 MHz, $CDCl_3$) δ_H: 2.77 (2H, td, *J* 7.5, 1.3, C*H*$_2$C(O)H), 3.34 (2H, t, *J* 7.5, C*H*$_2$), 7.31 (1H, dt, *J* 7.5, 0.6, 5-Ar*H*), 7.42 (1H, dt, *J* 7.5, 1.3, 3-Ar*H*), 7.51 (1H, td, *J* 7.5, 1.6, 4-Ar*H*), 7.79 (1H, dd, *J* 7.5, 1.5, 2-Ar*H*), 9.80 (1H, t, *J* 1.3, C(O)*H*), 10.15 (1H, s, ArC(O)*H*).

2-(3-Oxopropyl)benzaldehyde **S2** (2.70 g, 16.6 mmol) was dissolved in PhMe:MeCN (3:1, 50 mL) and cooled to -78 °C. Ethyl 2-(triphenylphosporanylidene) acetate (4.88 g, 14.0 mmol) was added and the solution was allowed to warm to rt and was stirred for 16 h. Concentration *in vacuo*, followed by silica chromatography (80:20 petrol:diethyl ether) gave the title compound **S3** as a colourless oil. (1.10 g, 34%). ν_{max} (neat) 1702 (C=O), 1698 (C=O), 1653 (C-O), 1600 (C-O), 1512 (C=C), 1452 (C=C), 1313 (C-O), 1276, 1190; ^{1}H NMR (300 MHz, $CDCl_3$) δ_H: 1.28 (3H, t, *J* 7.1, CH$_2$C*H*$_3$), 2.47-2.55 (2H, m, C*H*$_2$CH), 3.20 (2H, t, *J* 7.8, ArC*H*$_2$), 4.18 (2H, q, *J* 7.1, C*H*$_2$CH$_3$), 5.85 (1H, dt, *J* 15.6, 7.6, CH$_2$CHC*H*), 7.01 (1H, dt, *J* 15.6, 6.9, CH$_2$C*H*CH), 7.27 (1H, dd, *J* 7.5, 0.4, 5-Ar*H*), 7.42 (1H, td, *J* 7.5, 1.3, 3-Ar*H*), 7.52 (1H, td, *J* 7.5, 1.6, 4-Ar*H*), 7.82 (1H, dd, *J* 7.6, 1.5, 2-Ar*H*), 10.19 (1H, s, C(O)*H*); ^{13}C{^{1}H} (75 MHz, CD_2Cl_2) δ_C: 14.3 (*C*H$_3$), 31.5 (Ar*C*H$_2$), 33.9 (*C*H$_2$CH), 60.2 (*C*H$_2$CH$_3$), 122.1 (CH$_2$CH*C*H), 127.0 (3-Ar*C*H), 131.1 (5-Ar*C*H), 133.7 (4-Ar*C*H), 133.8 (2-Ar*C*H), 143.2 (6-Ar*C*), 147.6 (CH$_2$*C*HCH), 166.4 (*C*(O)OEt), 192.7 (*C*(O)H); *m/z* (APCI$^+$) 233 ([M]$^+$, 100%); HRMS (APCI$^+$) $C_{13}H_{15}O_4$ [M]$^+$ found 233.1175, requires 233.1172 (+1.2 ppm).

(1-D)-2-Methoxybenzaldehyde *d*-2

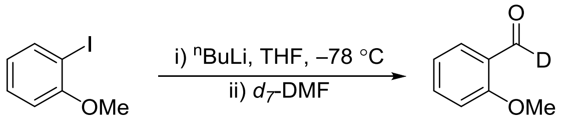

***d*-2**

Based upon a literature procedure,[3] ^{n}BuLi (1.35 M in hexanes, 6.5 mL, 8.8 mmol) was added dropwise through a dropping funnel to a solution of 2-iodoanisole (1.0 mL, 8.0 mmol) in THF (45 mL) at –78 °C. The reaction was stirred for 30 min before d7-DMF (0.68 mL, 8.8 mmol) was added and the solution stirred for a further 30 min at –78 °C. The reaction was quenched with a few drops of H_2SO_4 followed by aqueous NH_4Cl (40 mL) and allowed to warm to rt. The solution was extracted with Et_2O (3 × 75 mL) and the combined organics dried ($MgSO_4$) and concentrated *in vacuo*. The crude product was purified by silica chromatography (90:10 hexane: EtOAc) to give ***d*-2** as a yellow oil (1.02 g, 93%, >99% D), with data in accordance with the literature.[3] ^{1}H NMR (400 MHz, CD_2Cl_2) δ_H: 3.93 (3H, s, OC*H*$_3$), 6.67–7.05 (2H, m, ArC(3,5)*H*), 7.56 (1H, ddd, *J* 8.7, 6.9, 1.8, ArC(4)*H*), 7.84 (1H, dd, *J* 7.7, 1.9, ArC(6)*H*).

Determination of Equilibrium Constants in CD_2Cl_2 (Table 1)

In an NMR tube, aldehyde (0.0175 mmol) and triazolium salt (0.0075 mmol) were dissolved in CD_2Cl_2 (0.75 mL). The reaction was initiated by the addition of NEt_3 (1 μL, 0.0075 mmol) and monitored by ^{1}H NMR spectroscopy (400 MHz) at 25 ºC. The concentrations of reactants and intermediates determined from the integral of the species itself, relative to the integral of the internal standard tetramethylsilane (TMS). The concentration of the internal standard was set relative to the integral corresponding to aldehyde at t=0, which corresponded to a known starting concentration of 0.01 M. In all cases, product formation (either homo-benzoin or Stetter) was minimal, with < 5% observed by the latest time point.

The concentration of aldehyde was determined using the singlet at ~10-11 ppm (A), corresponding to the aldehyde C=O(H). The singlet at ~10.0 ppm (B) was used to determine the concentration of NHC precursor, which was assigned to the triazole CH. Initially a new set of peaks was observed, which was assigned to the 3-(hydroxybenzyl)azolium salt, the concentration of which was calculated from the singlet at ~6.2 ppm (C), corresponding to the C(*H*)(OH) proton. To ensure there was very little product present, the appearance of a triplet at 4.30 ppm was monitored, which corresponds to one of the CH_2 protons on the 6-membered ring of the Stetter product. The reaction was monitored until no change in the concentrations of any of the species was observed.

Over the course of the experiment, no other significant peaks were observed. The concentrations could be verified using other peaks, with no significant variation in calculated concentration.

Table 1, Entry 1

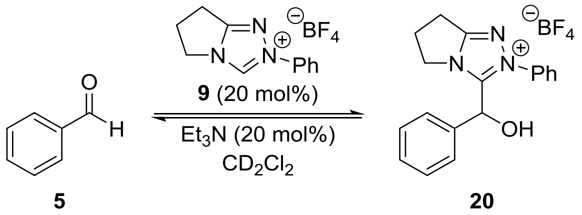

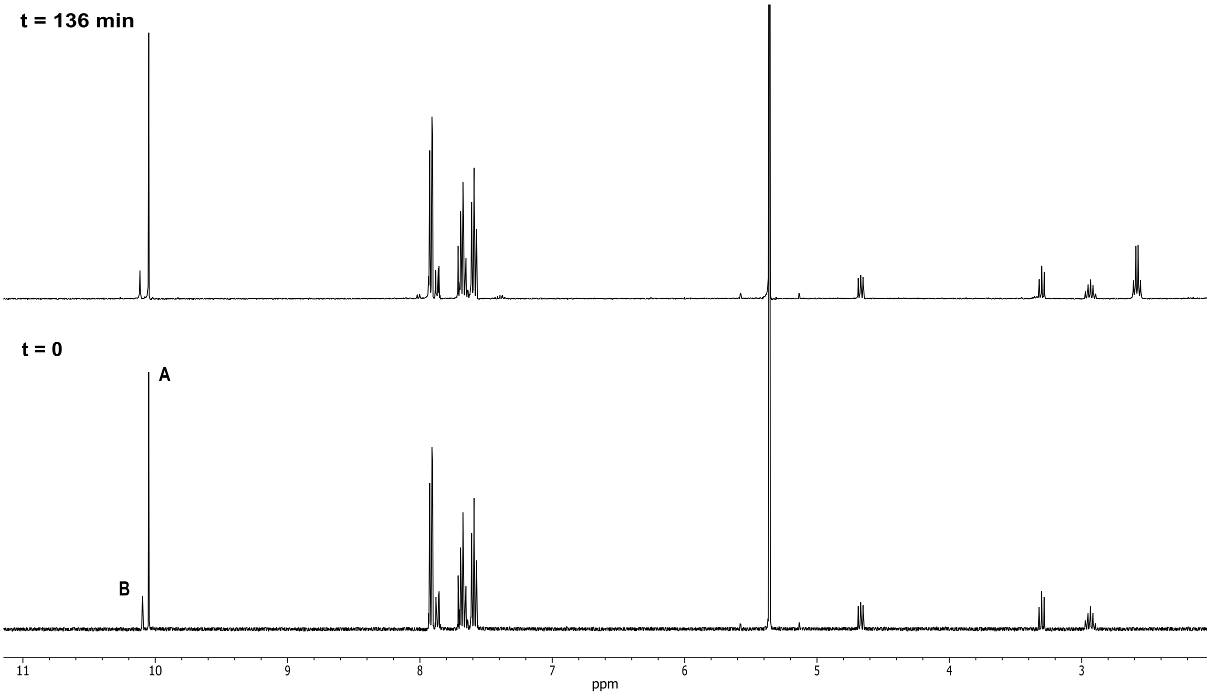

Figure S1. Representative ^{1}H NMR spectra (500 MHz) for reaction of benzaldehyde **5** (0.01 M) with *N*-Ph NHC precursor **9** (0.002 M) and Et_3N (0.002 M) in CD_2Cl_2 at 25 °C. A = PhC*H*O, B = NHC precursor NC*H*N

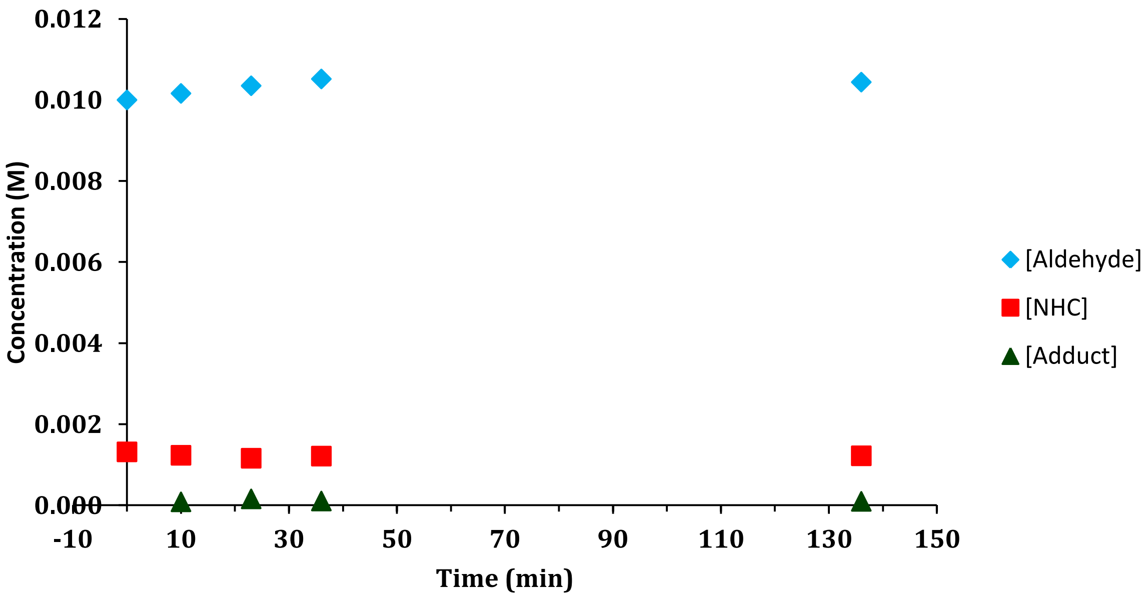

Figure S2. Reaction profile displaying concentration of species present against time for reaction of benzaldehyde **5** (0.01 M) with *N*-Ph NHC precursor **9** (0.002 M) and Et_3N (0.002 M) in CD_2Cl_2 at 25 °C

Table 1, Entry 2

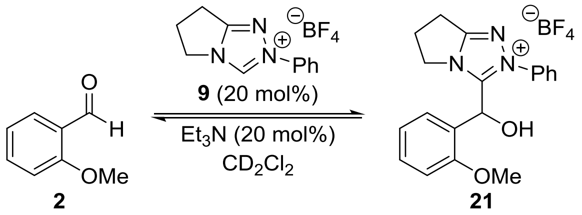

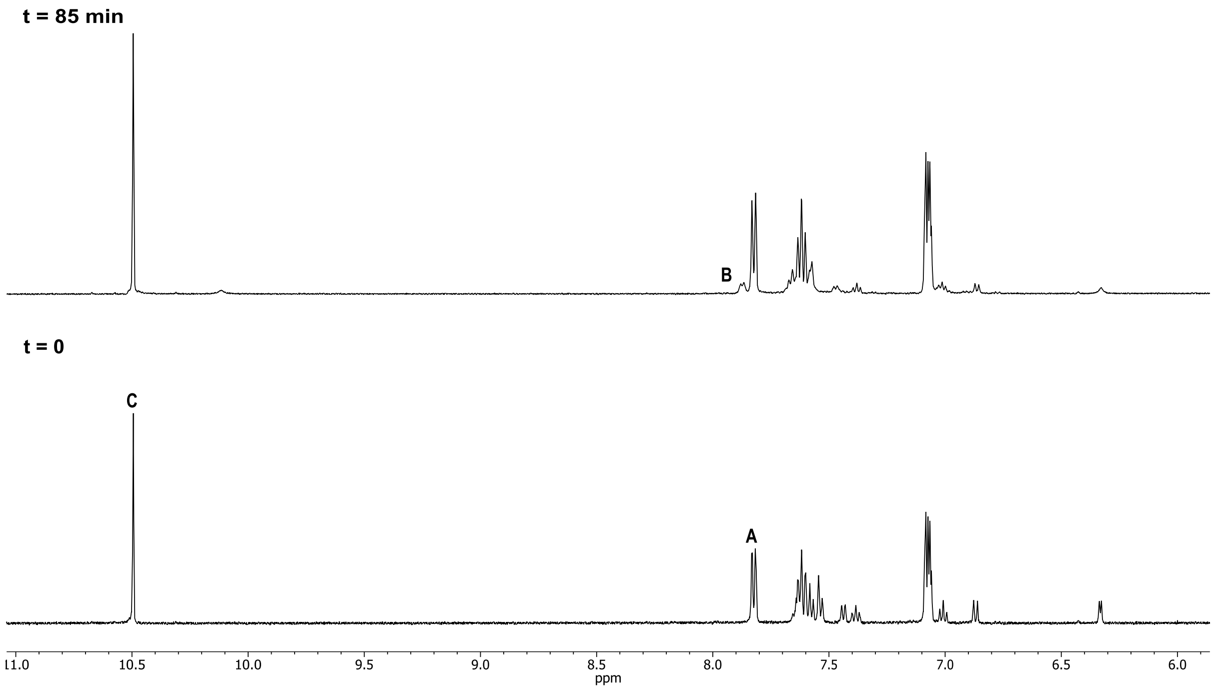

Figure S3. Representative ^{1}H NMR spectra (500 MHz) for reaction of 2-methoxybenzaldehyde **2** (0.01 M) with *N*-Ph NHC precursor **9** (0.002 M) and Et_3N (0.002 M) in CD_2Cl_2 at 25 °C. A = ArC*H*O, B = NHC precursor, C = adduct.

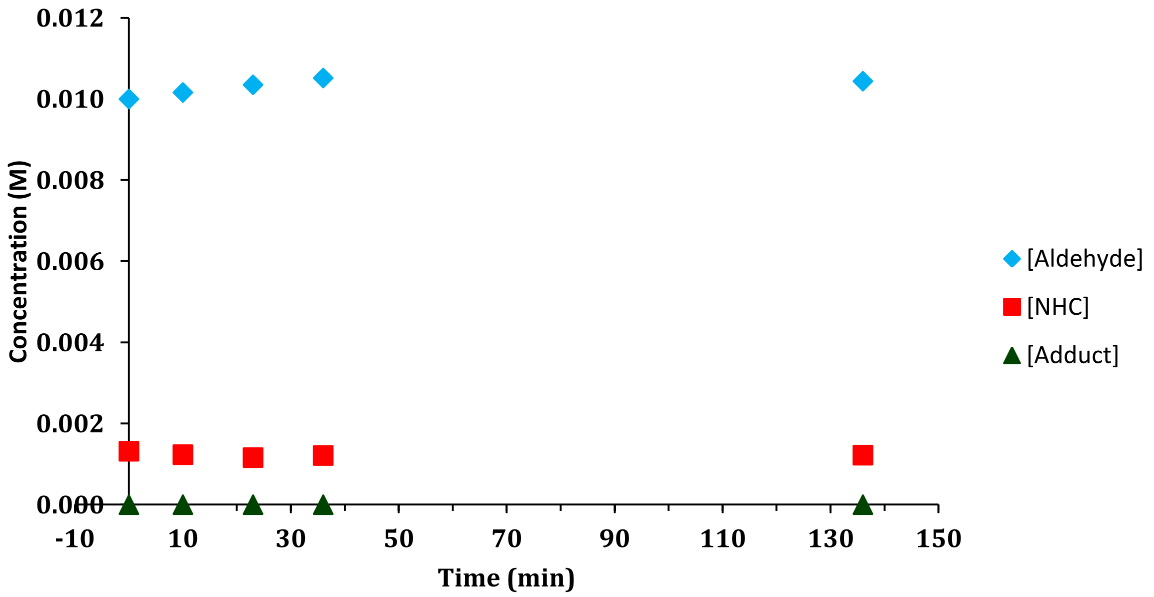

Figure S4. Reaction profile displaying concentration of species present against time for reaction of 2-methoxy benzaldehyde **2** (0.01 M) with *N*-Ph NHC precursor **9** (0.002 M) and Et_3N (0.002 M) in CD_2Cl_2 at 25 °C

Table 1, Entry 3

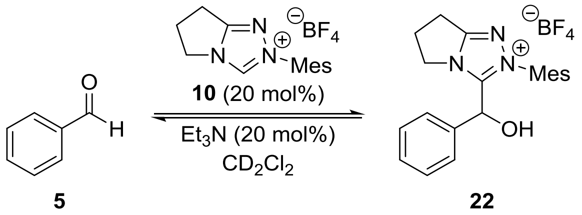

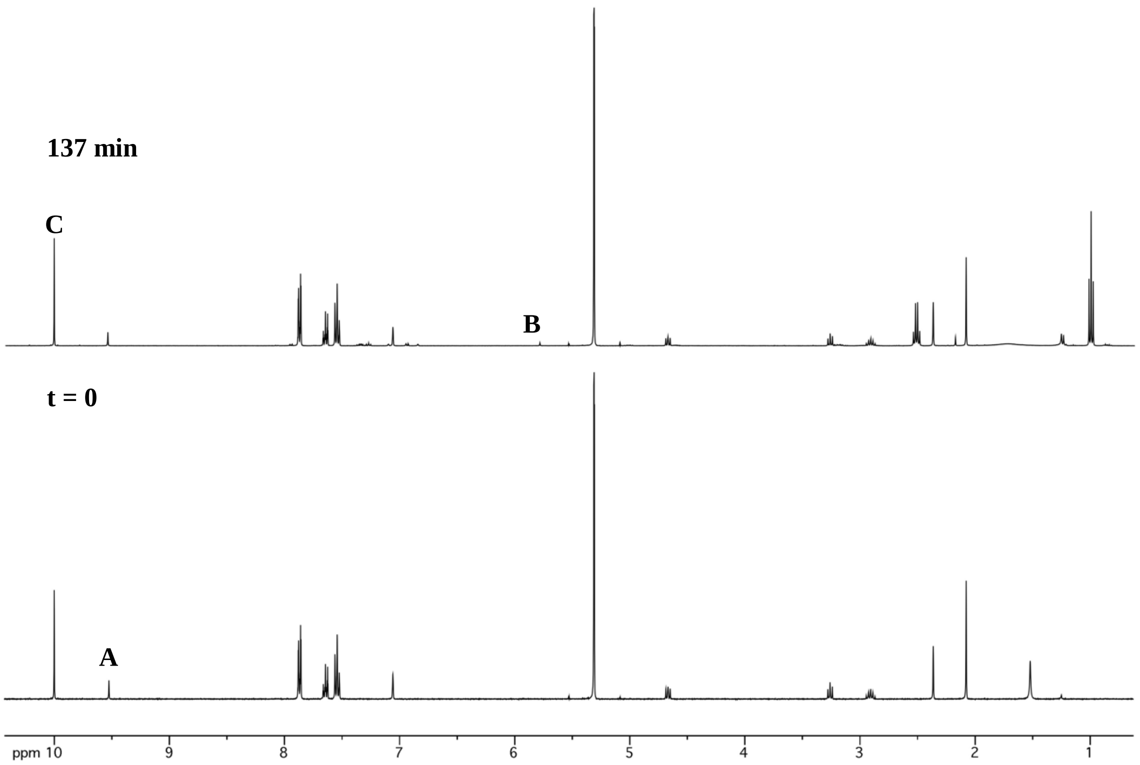

Figure S5. Representative ^{1}H NMR spectra (500 MHz) for reaction of benzaldehyde **5** (0.01 M) with *N*-Mes NHC precursor **10** (0.002 M) and Et_3N (0.002 M) in CD_2Cl_2 at 25 °C. A = NHC precursor NC*H*N, B = adduct C(α)*H*, C = PhC*H*O.

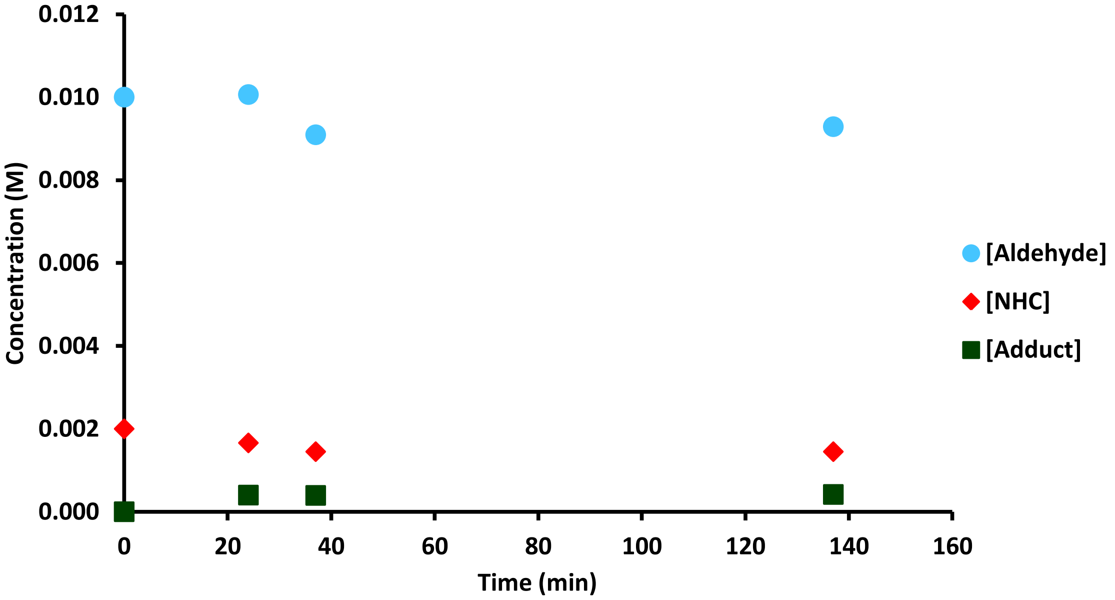

Figure S6. Reaction profile displaying concentration of species present against time for reaction of benzaldehyde **5** (0.01 M) with *N*-Mes NHC precursor **10** (0.002 M) and Et_3N (0.002 M) in CD_2Cl_2 at 25 °C.

Table 1, Entry 4

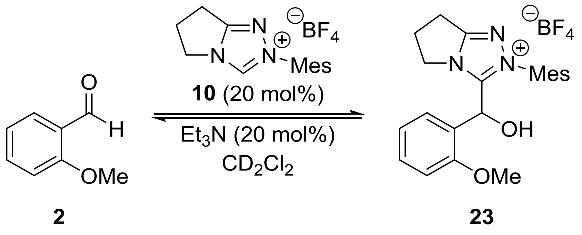

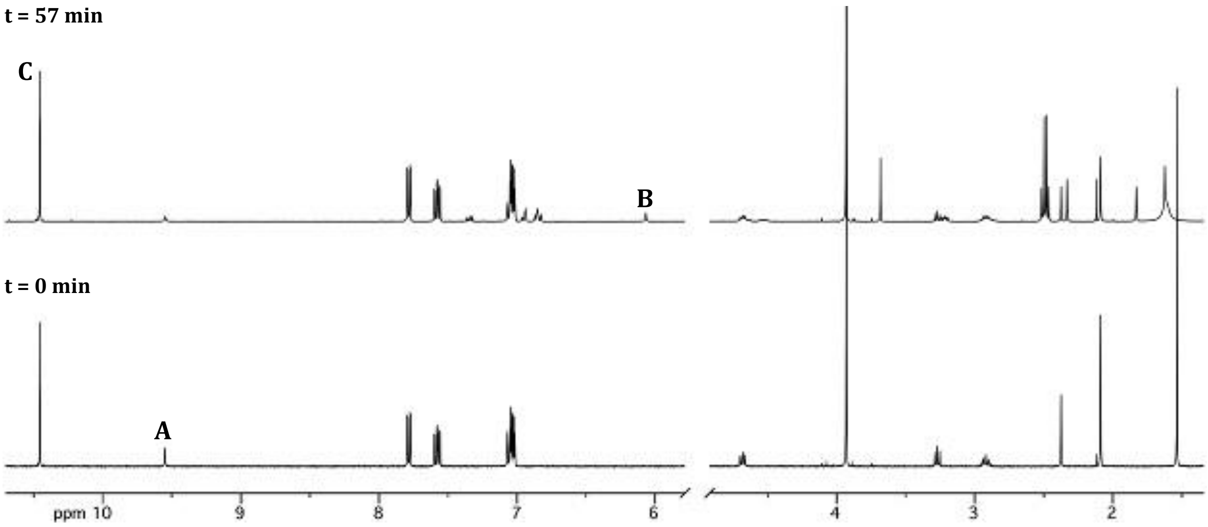

Figure S7. Representative ^{1}H NMR spectra (500 MHz) for reaction of 2-methoxybenzaldehyde **2** (0.01 M) with *N*-Mes NHC precursor **10** (0.002 M) and Et_3N (0.002 M) in CD_2Cl_2 at 25 °C. A = NHC precursor NC*H*N, B = adduct C(α)*H*, C = ArC*H*O.

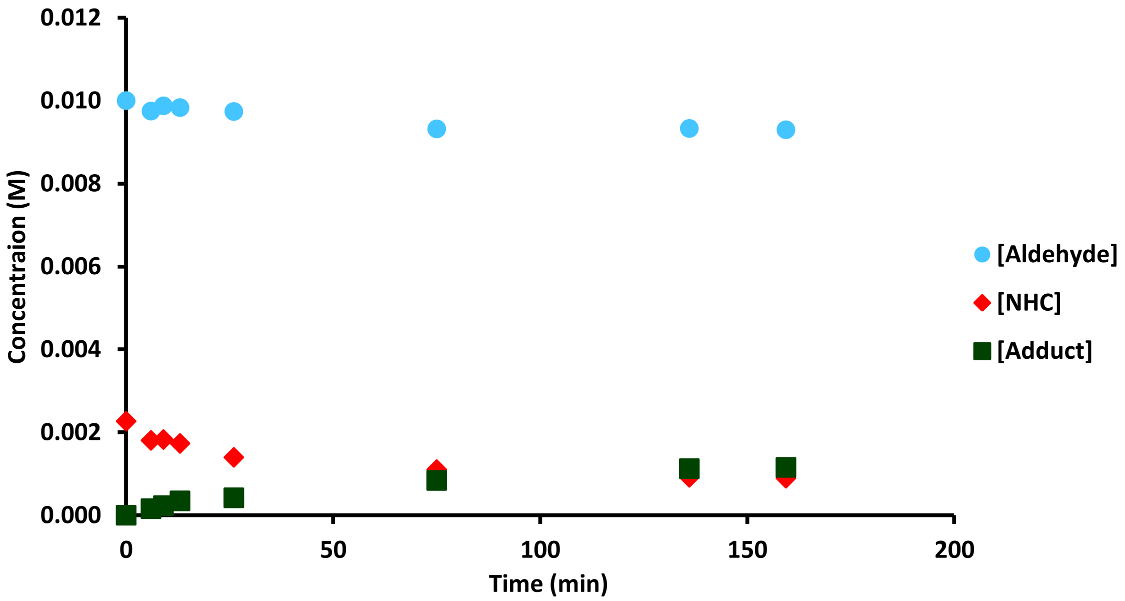

Figure S8. Reaction profile displaying concentration of species present against time for reaction of 2-methoxybenzaldehyde **2** (0.01 M) with *N*-Mes NHC precursor **10** (0.002 M) and Et_3N (0.002 M) in CD_2Cl_2 at 25 °C.

Table 1, Entry 5

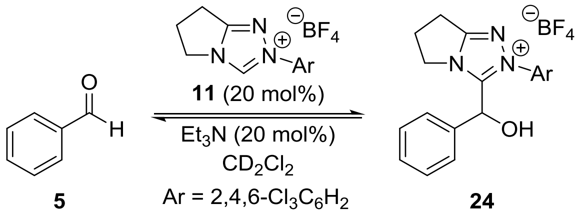

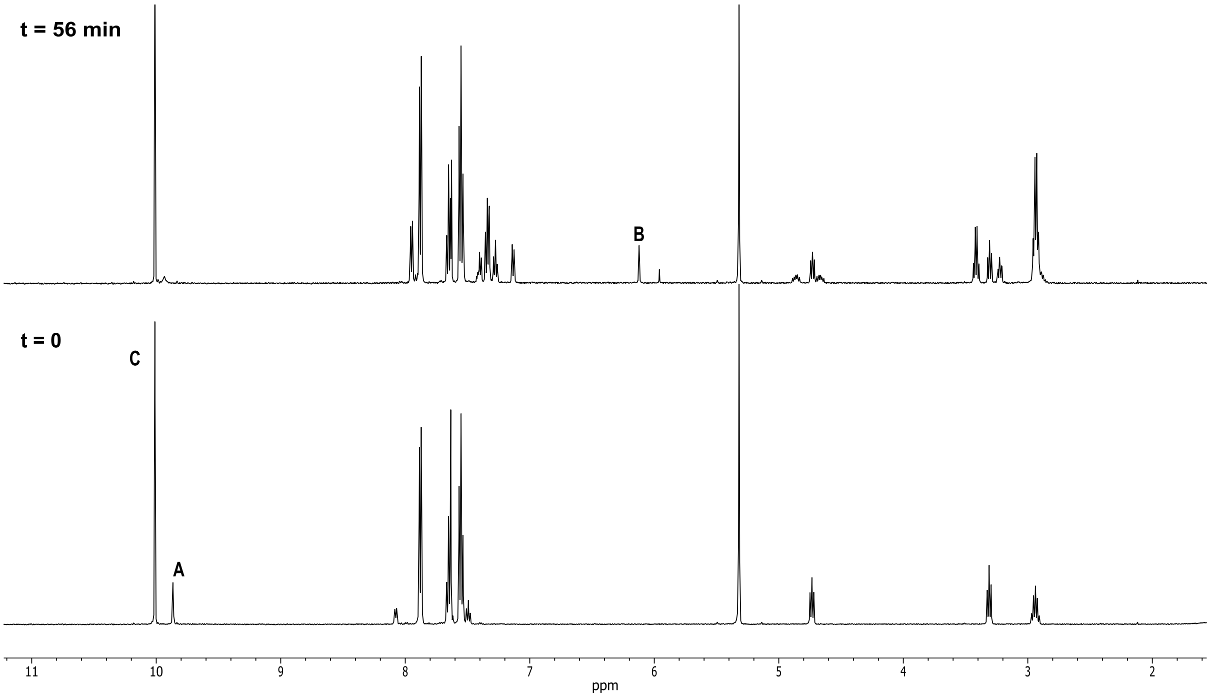

Figure S9. Representative ^{1}H NMR spectra (500 MHz) for reaction of benzaldehyde **5** (0.04 M) with *N*-2,4,6-$CLl_3C_6H_2$ NHC precursor **11** (0.008 M) and Et_3N (0.008 M) in CD_2Cl_2 at 25 °C. A = NHC precursor NC*H*N, B = adduct C(α)*H*, C = PhC*H*O.

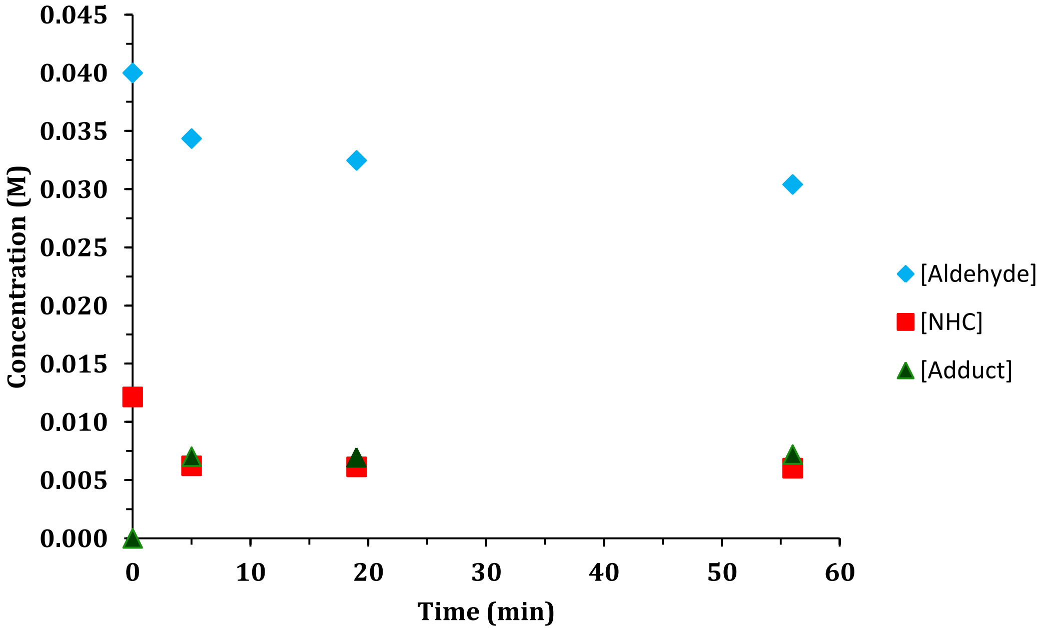

Figure S10. Reaction profile displaying concentration of species present against time for reaction of benzaldehyde **5** (0.04 M) with *N*-2,4,6-$CLl_3C_6H_2$ NHC precursor **11** (0.008 M) and Et_3N (0.008 M) in CD_2Cl_2 at 25 °C

Table 1, Entry 6

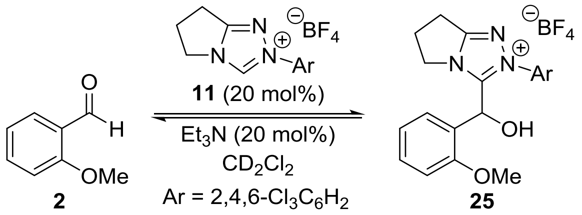

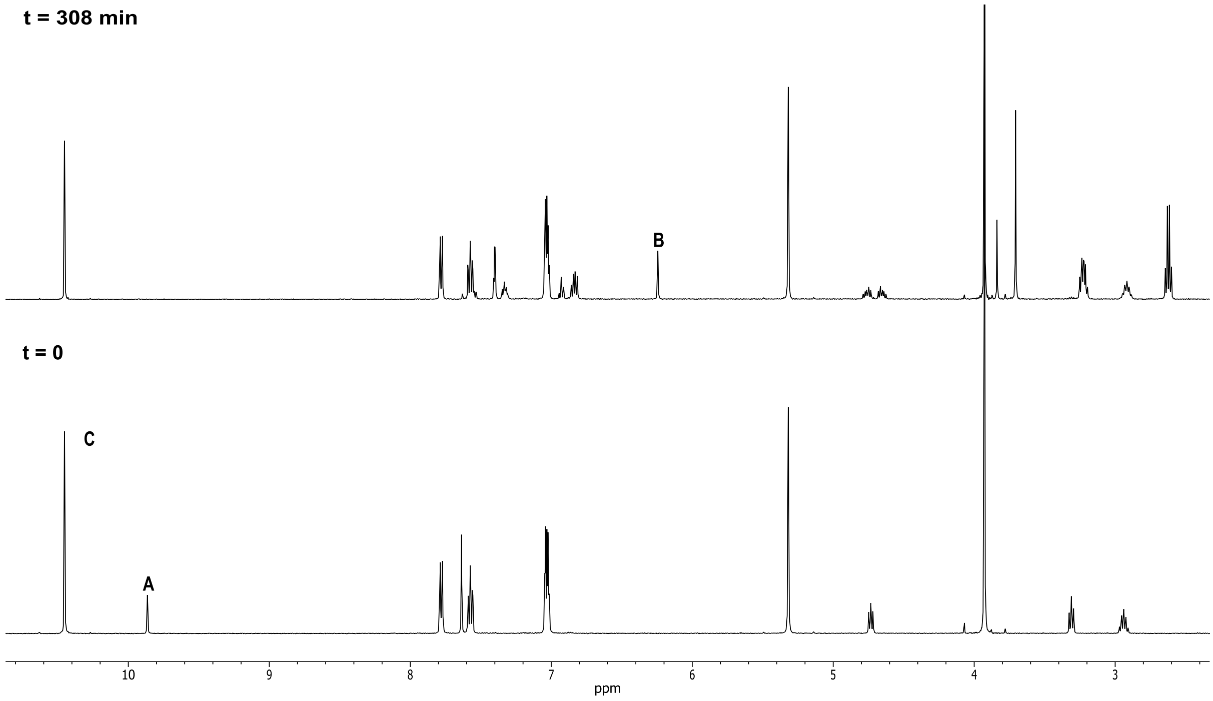

Figure S11. Representative ^{1}H NMR spectra (500 MHz) for reaction of 2-methoxybenzaldehyde **2** (0.04 M) with *N*-2,4,6-CLl$_3$C$_6$H$_2$ NHC precursor **11** (0.008 M) and Et$_3$N (0.008 M) in CD$_2$Cl$_2$ at 25 °C. A = NHC precursor NC*H*N, B = adduct C(α)*H*, C = PhC*H*O.

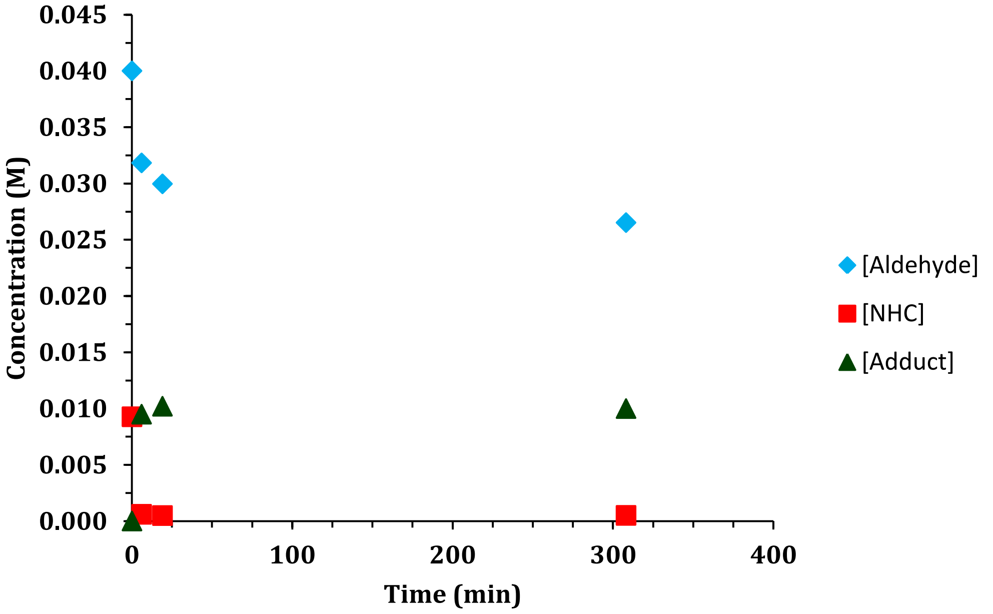

Figure S12. Reaction profile displaying concentration of species present against time for reaction of 2-methoxy benzaldehyde **2** (0.04 M) with *N*-2,4,6-CLl$_3$C$_6$H$_2$ NHC precursor **11** (0.008 M) and Et$_3$N (0.008 M) in CD$_2$Cl$_2$ at 25 °C

Table 1, Entry 7

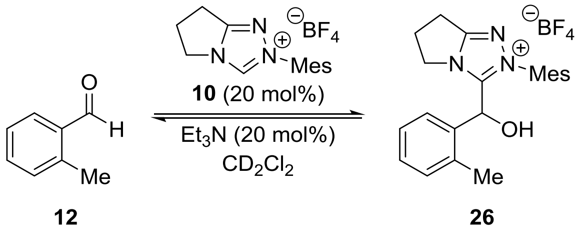

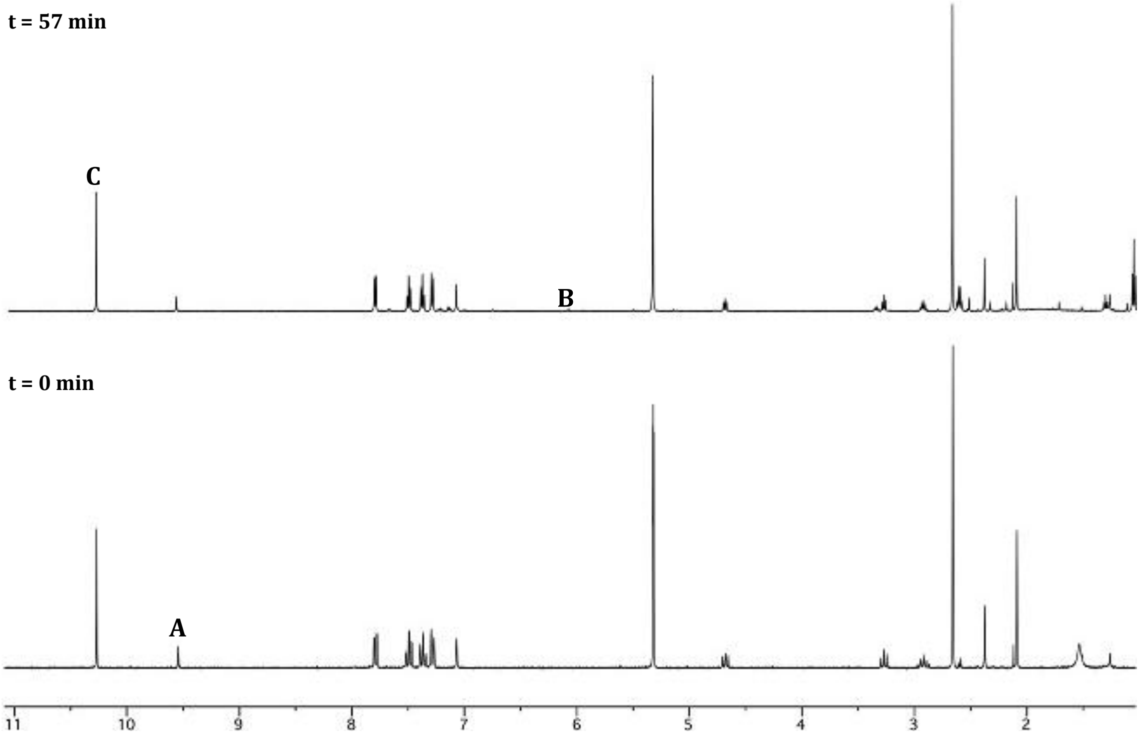

Figure S13. Representative ^{1}H NMR spectra (500 MHz) for reaction of 2-tolualdehyde **12** (0.01 M) with *N*-Mes NHC precursor **10** (0.002 M) and Et_3N (0.002 M) in CD_2Cl_2 at 25 °C. A = NHC precursor NC*H*N, B = adduct C(α)*H*, C = ArC*H*O.

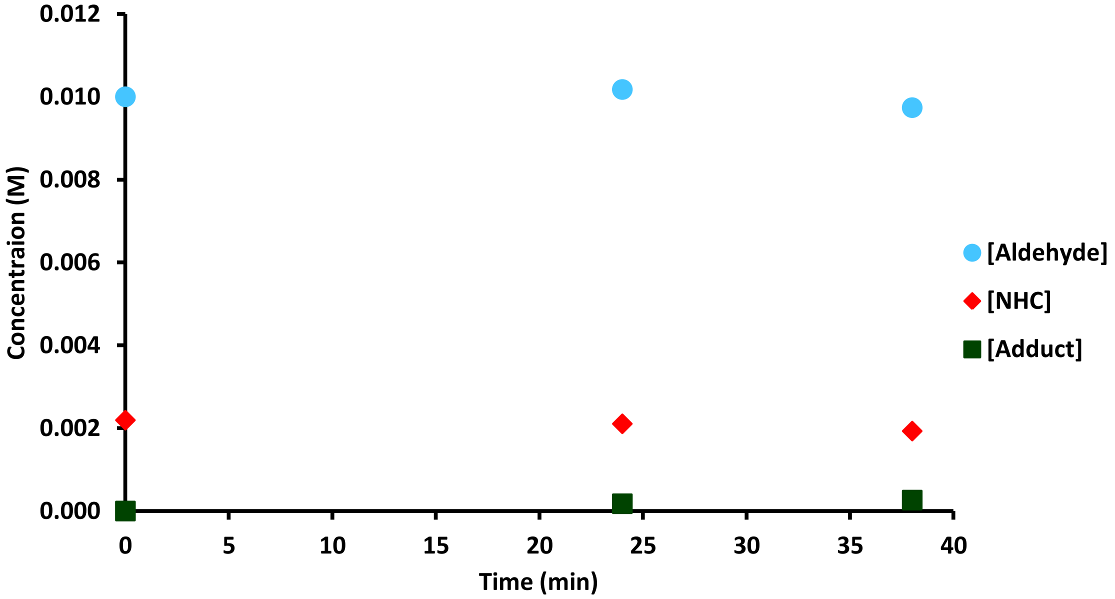

Figure S14. Reaction profile displaying concentration of species present against time for reaction of 2-tolualdehyde **12** (0.01 M) with *N*-Mes NHC precursor **10** (0.002 M) and Et_3N (0.002 M) in CD_2Cl_2 at 25 °C.

Table 1, Entry 8

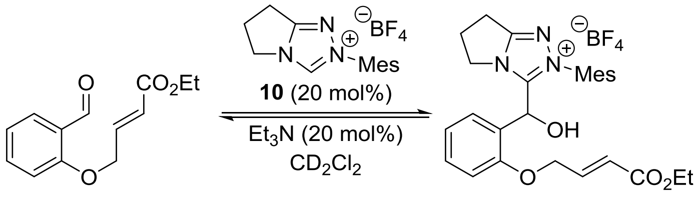

13 27

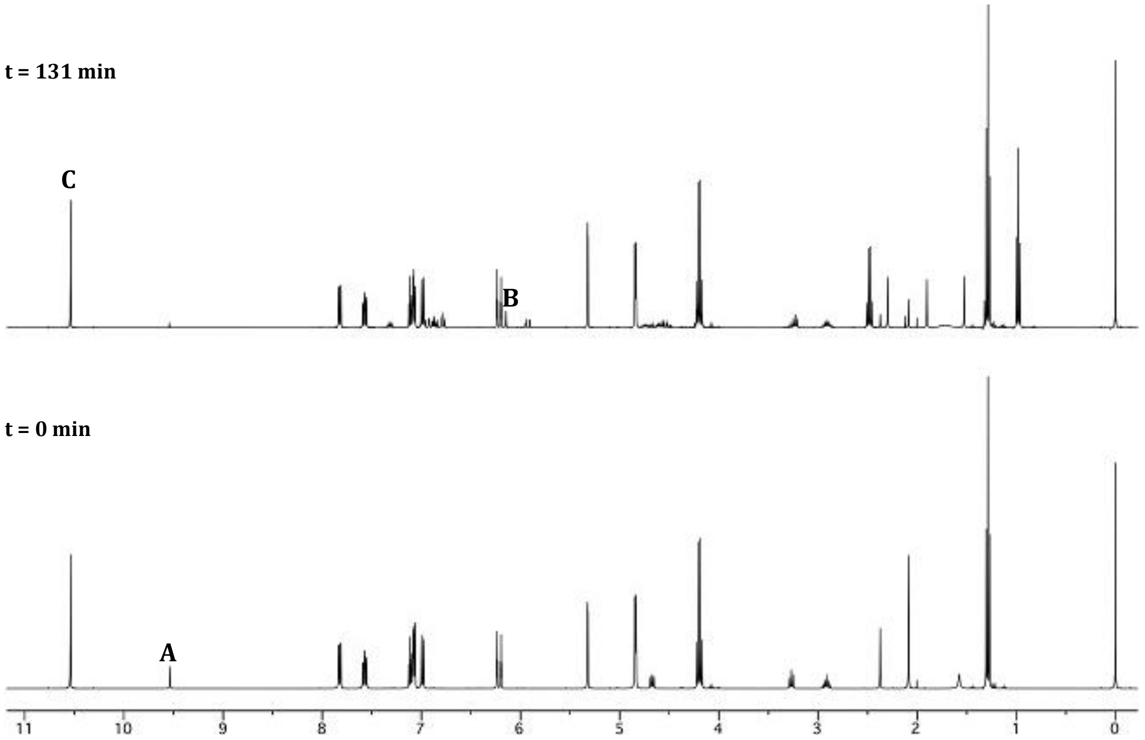

Figure S15. Representative ^{1}H NMR spectra (500 MHz) for reaction of aldehyde **13** (0.01 M) with *N*-Mes NHC precursor **10** (0.002 M) and Et_3N (0.002 M) in CD_2Cl_2 at 25 °C. A = NHC precursor NC*H*N, B = adduct C(α)*H*, C = ArC*H*O.

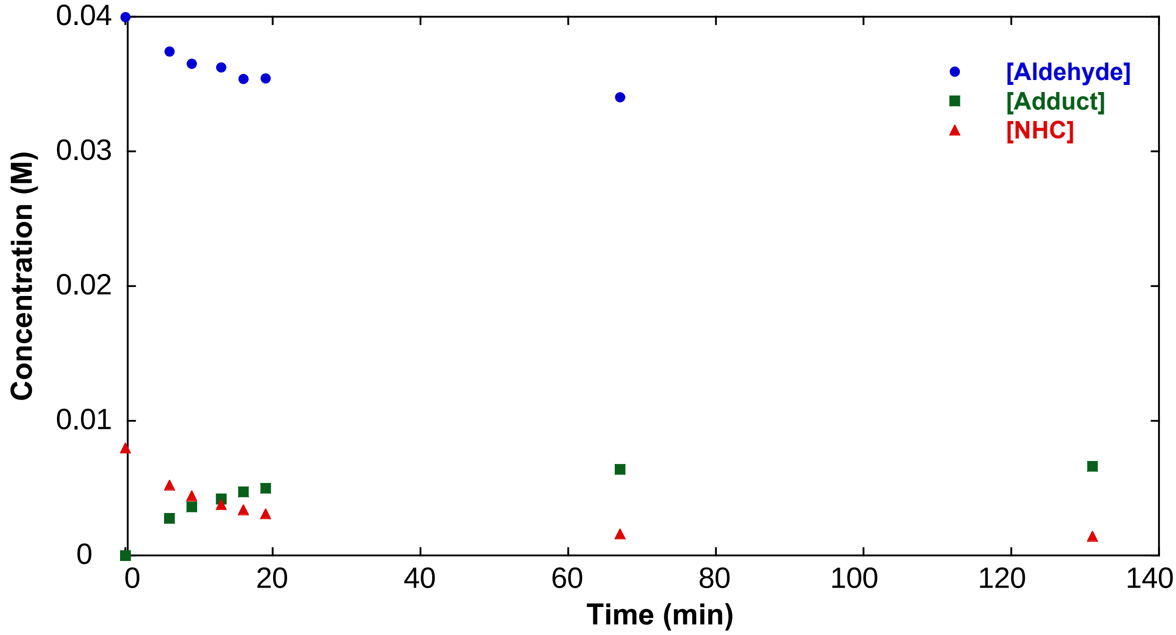

Figure S16. Reaction profile displaying concentration of species present against time for reaction of aldehyde **13** (0.01 M) with *N*-Mes NHC precursor **10** (0.002 M) and Et_3N (0.002 M) in CD_2Cl_2 at 25 °C.

Table 1, Entry 9

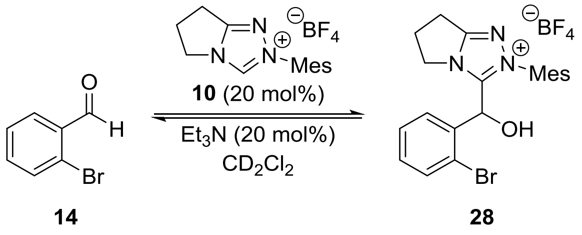

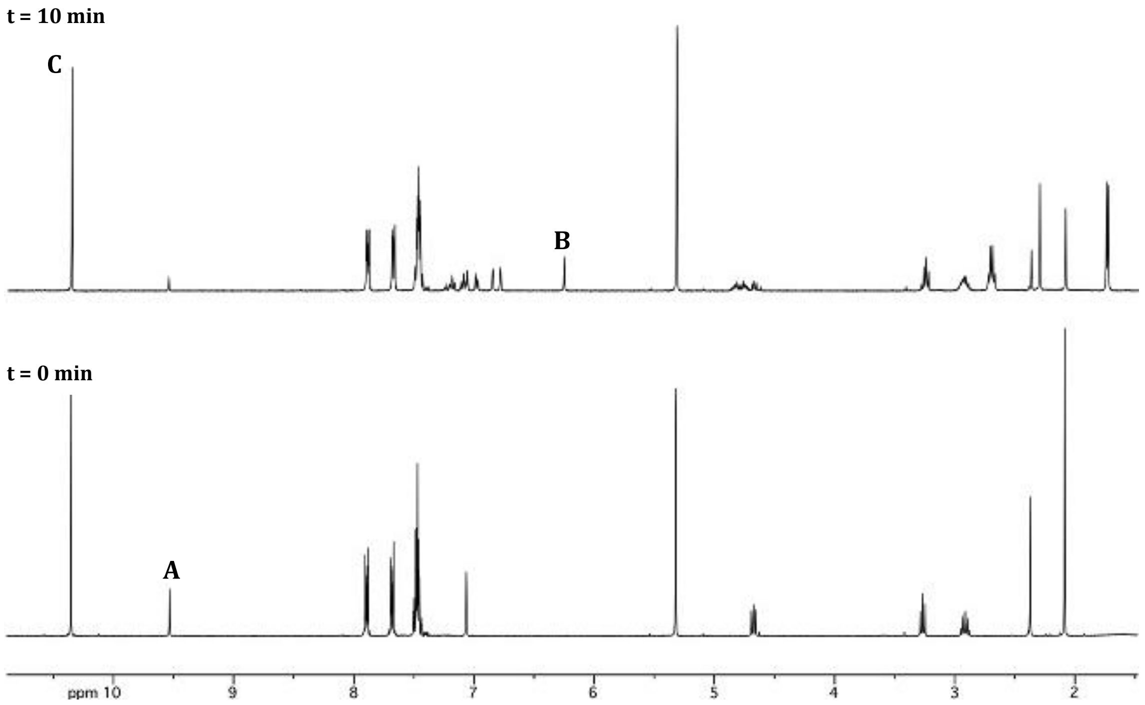

Figure S17. Representative ^{1}H NMR spectra (500 MHz) for reaction of 2-bromobenzaldehyde **14** (0.01 M) with *N*-Mes NHC precursor **10** (0.002 M) and Et_3N (0.002 M) in CD_2Cl_2 at 25 °C. A = NHC precursor NC*H*N, B = adduct C(α)*H*, C = ArC*H*O.

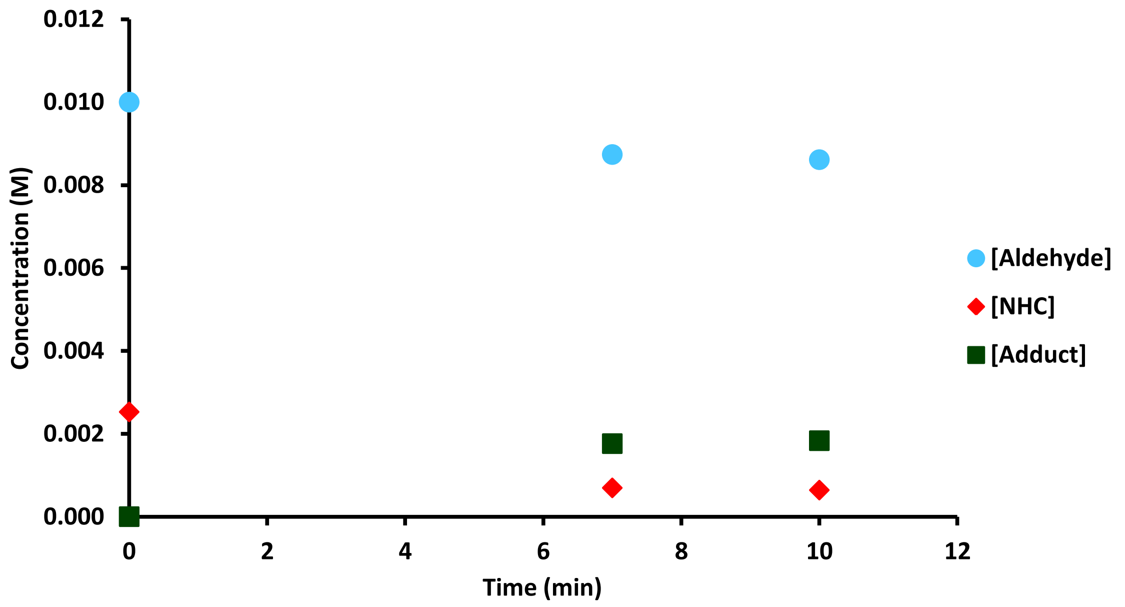

Figure S18. Reaction profile displaying concentration of species present against time for reaction of 2-bromobenzaldehyde **14** (0.01 M) with *N*-Mes NHC precursor **10** (0.002 M) and Et_3N (0.002 M) in CD_2Cl_2 at 25 °C.

Table 1, Entry 10

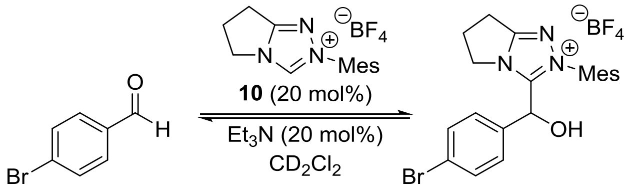

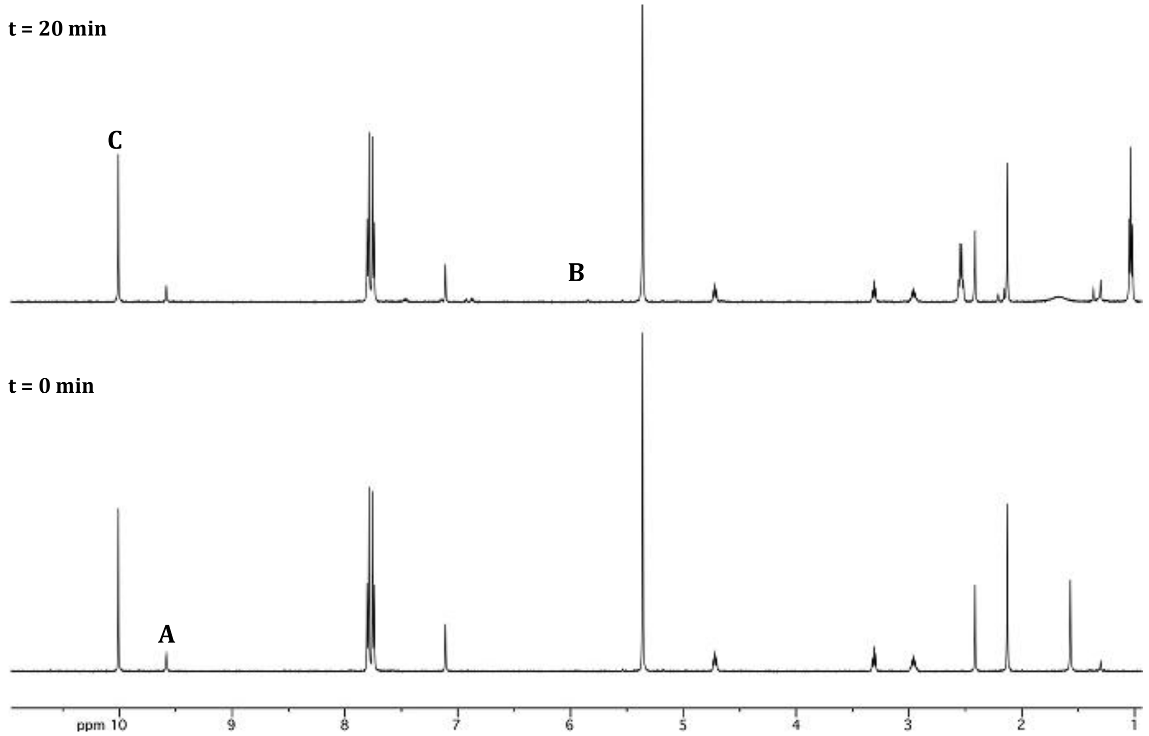

Figure S19. Representative ^{1}H NMR spectra (500 MHz) for reaction of 4-bromobenzaldehyde **15** (0.01 M) with *N*-Mes NHC precursor **10** (0.002 M) and Et_3N (0.002 M) in CD_2Cl_2 at 25 °C. A = NHC precursor NC*H*N, B = adduct C(α)*H*, C = ArC*H*O.

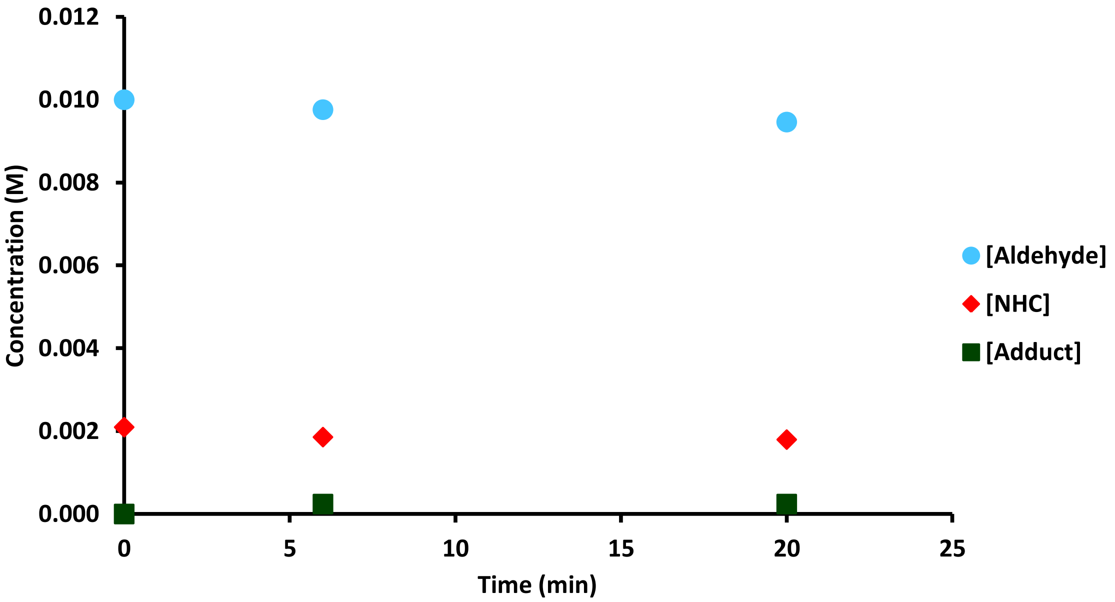

Figure S20. Reaction profile displaying concentration of species present against time for reaction of 4-bromobenzaldehyde **15** (0.01 M) with *N*-Mes NHC precursor **10** (0.002 M) and Et_3N (0.002 M) in CD_2Cl_2 at 25 °C.

Table 1, Entry 11

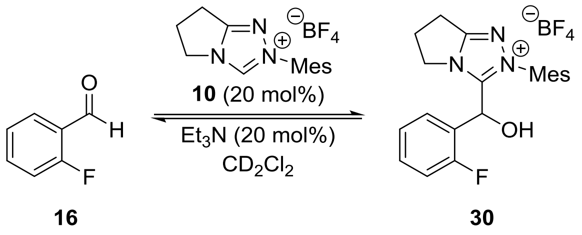

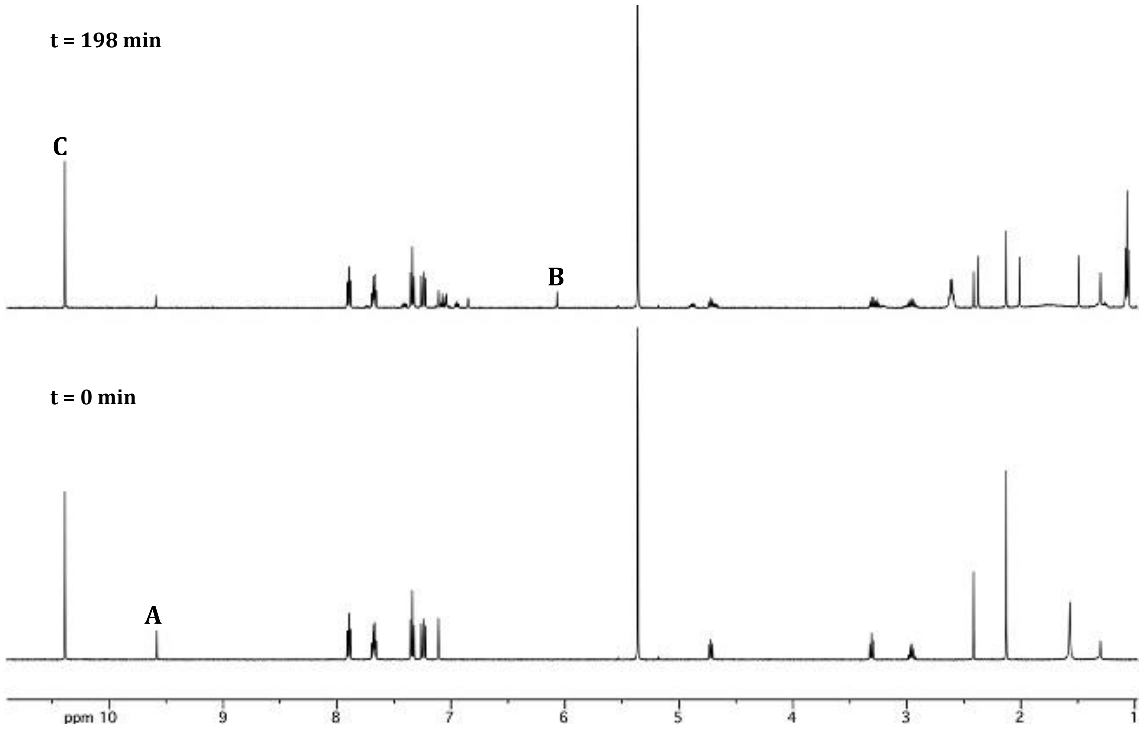

Figure S21. Representative ^{1}H NMR spectra (500 MHz) for reaction of 2-fluorobenzaldehyde **16** (0.01 M) with *N*-Mes NHC precursor **10** (0.002 M) and Et_3N (0.002 M) in CD_2Cl_2 at 25 °C. A = NHC precursor NC*H*N, B = adduct C(α)*H*, C = ArC*H*O.

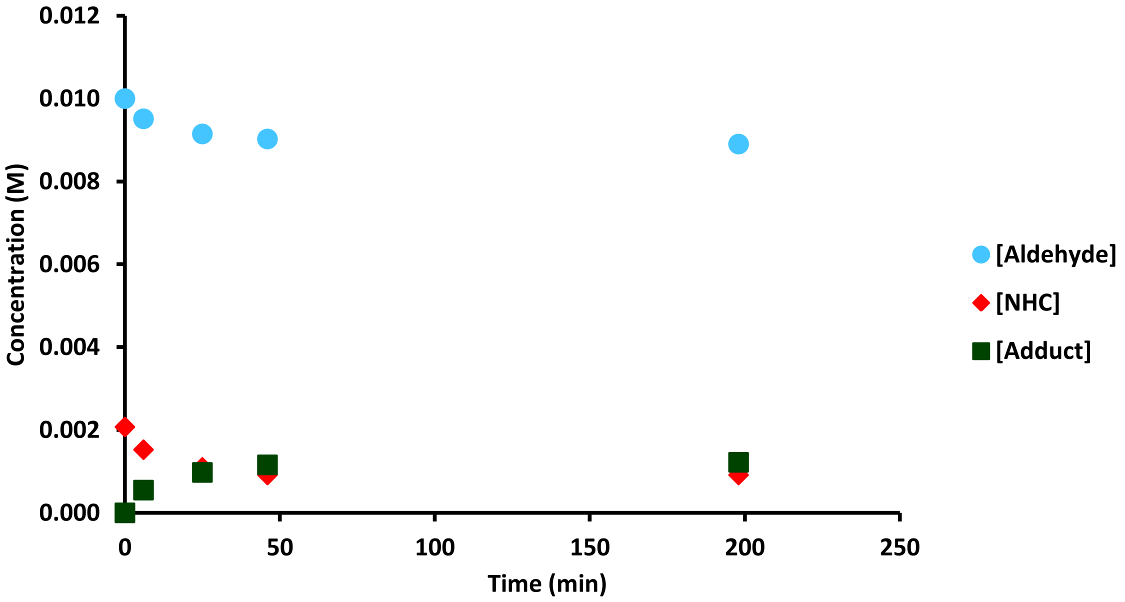

Figure S22. Reaction profile displaying concentration of species present against time for reaction of 2-fluorobenzaldehyde **16** (0.01 M) with *N*-Mes NHC precursor **10** (0.002 M) and Et_3N (0.002 M) in CD_2Cl_2 at 25 °C.

Table 1, Entry 12

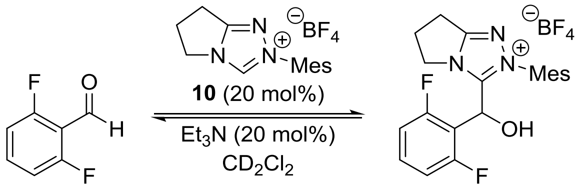

17 **31**

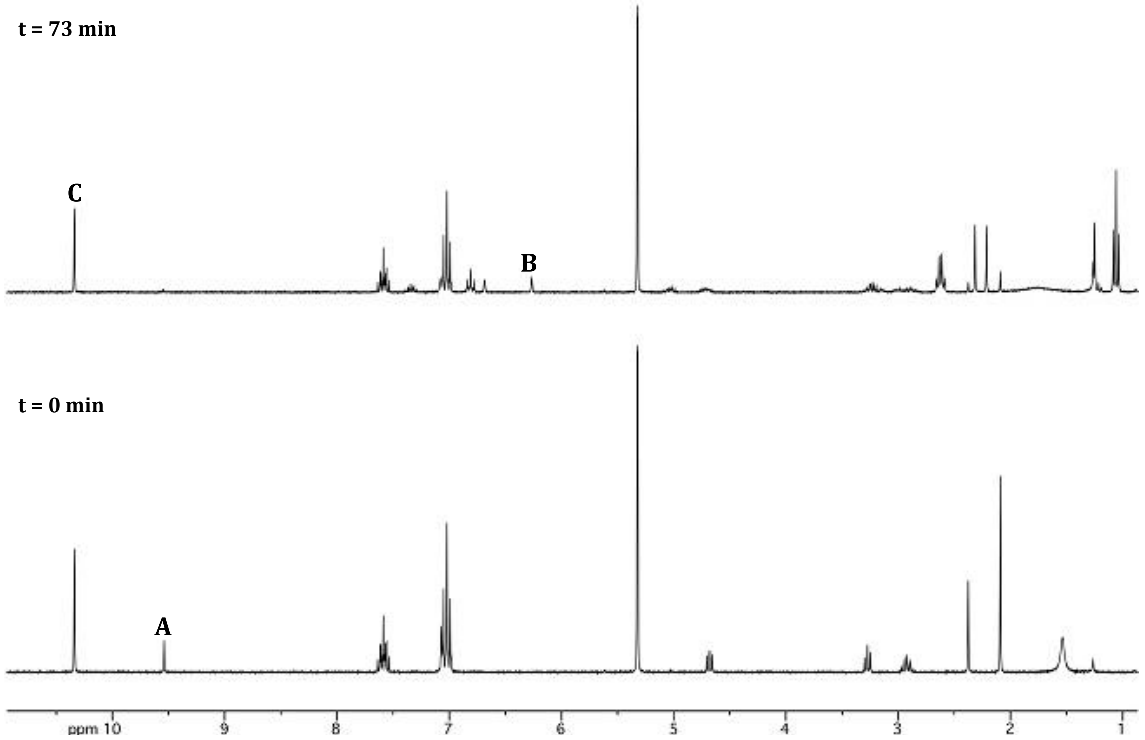

Figure S23. Representative ^{1}H NMR spectra (500 MHz) for reaction of 2,6-difluorobenzaldehyde **17** (0.01 M) with *N*-Mes NHC precursor **10** (0.002 M) and Et_3N (0.002 M) in CD_2Cl_2 at 25 °C. A = NHC precursor NC*H*N, B = adduct C(α)*H*, C = ArC*H*O.

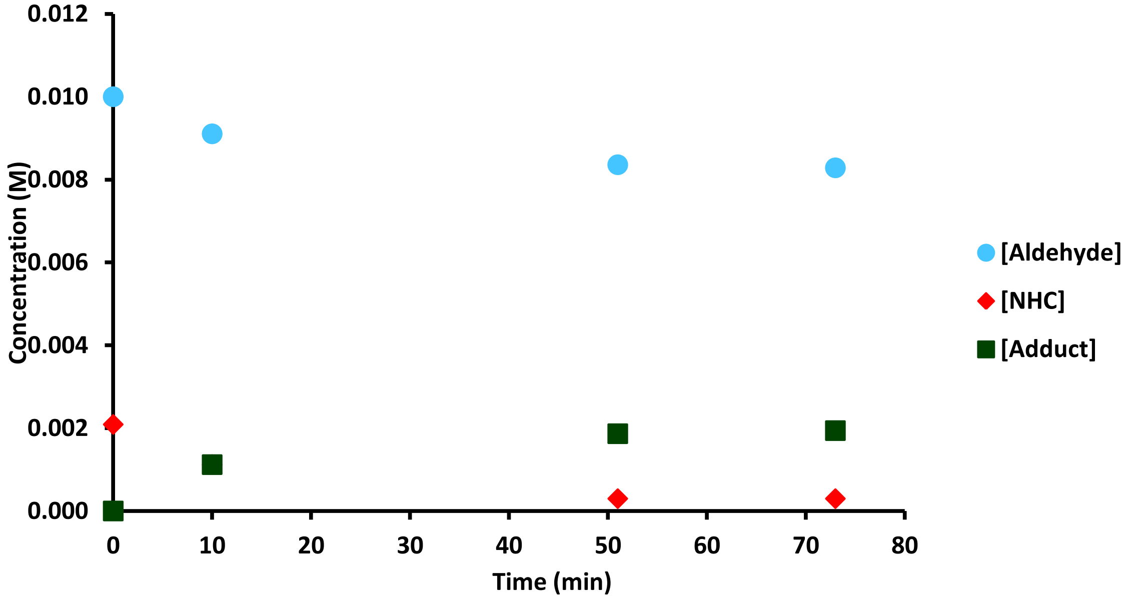

Figure S24. Reaction profile displaying concentration of species present against time for reaction of 2,6-difluorobenzaldehyde **17** (0.01 M) with *N*-Mes NHC precursor **10** (0.002 M) and Et_3N (0.002 M) in CD_2Cl_2 at 25 °C.

Table 1, Entry 13

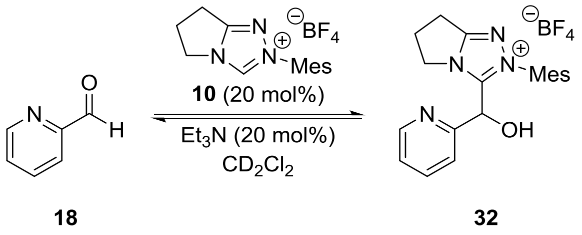

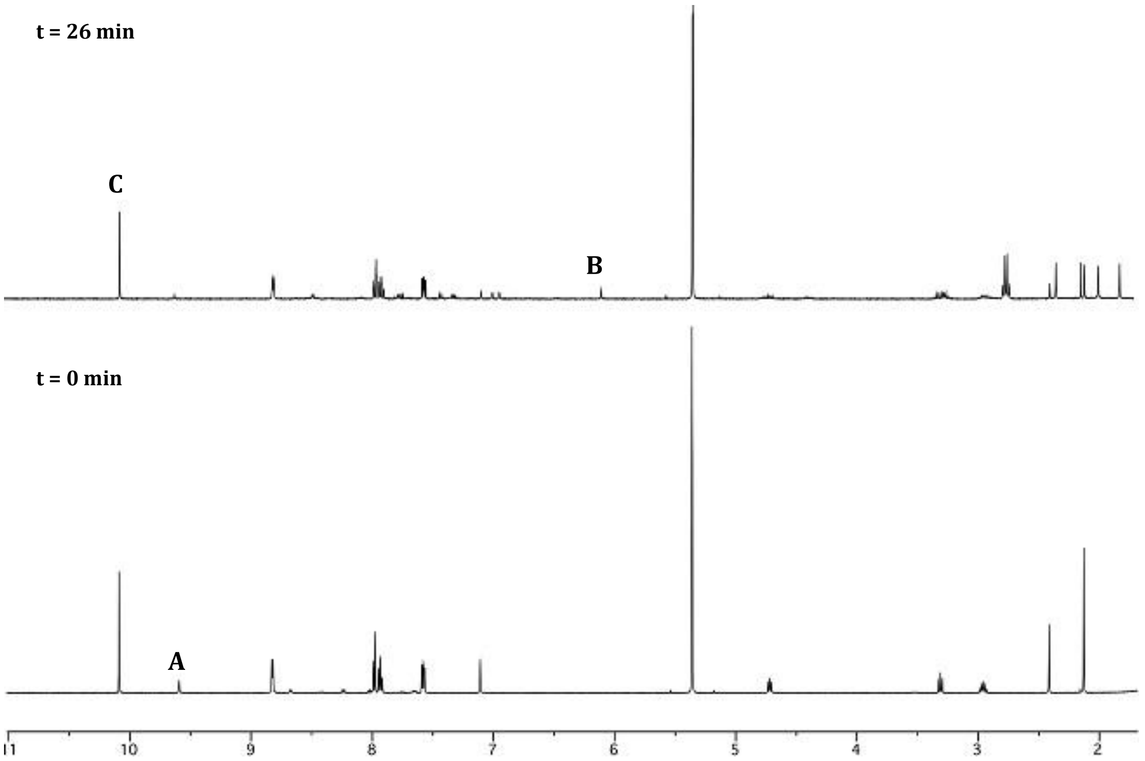

Figure S25. Representative ^{1}H NMR spectra (500 MHz) for reaction of 2-pyridinecarboxaldehyde **18** (0.01 M) with *N*-Mes NHC precursor **10** (0.002 M) and Et_3N (0.002 M) in CD_2Cl_2 at 25 °C. A = NHC precursor NC*H*N, B = adduct C(α)*H*, C = ArC*H*O.

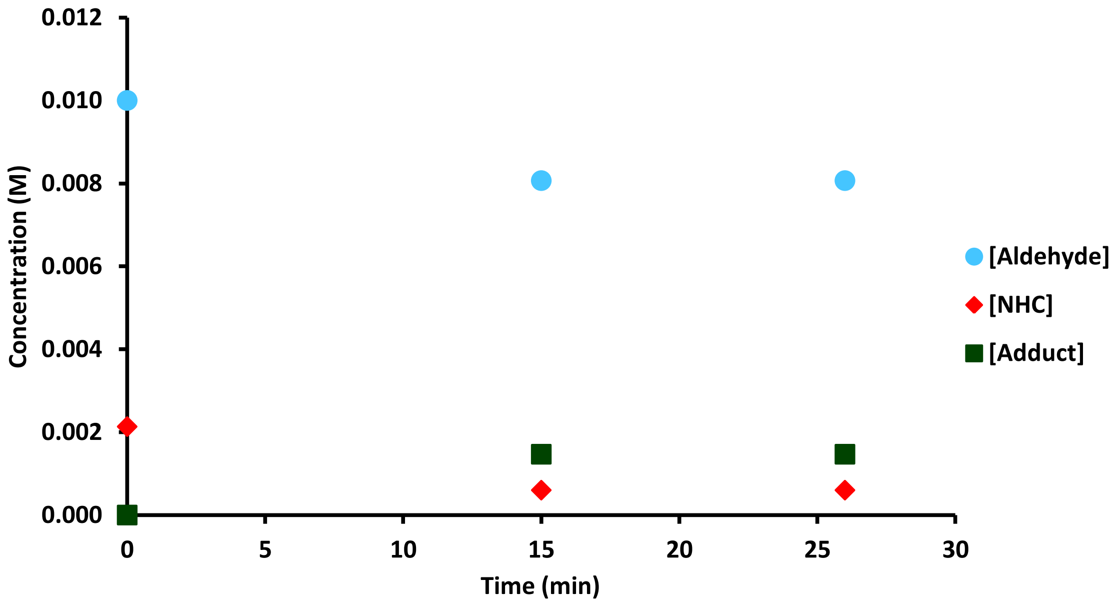

Figure S26. Reaction profile displaying concentration of species present against time for reaction of 2-pyridinecarboxaldehyde **18** (0.01 M) with *N*-Mes NHC precursor **10** (0.002 M) and Et_3N (0.002 M) in CD_2Cl_2 at 25 °C.

Table 1, Entry 14

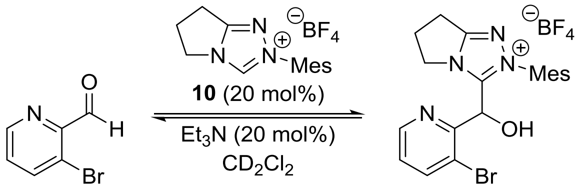

19 33

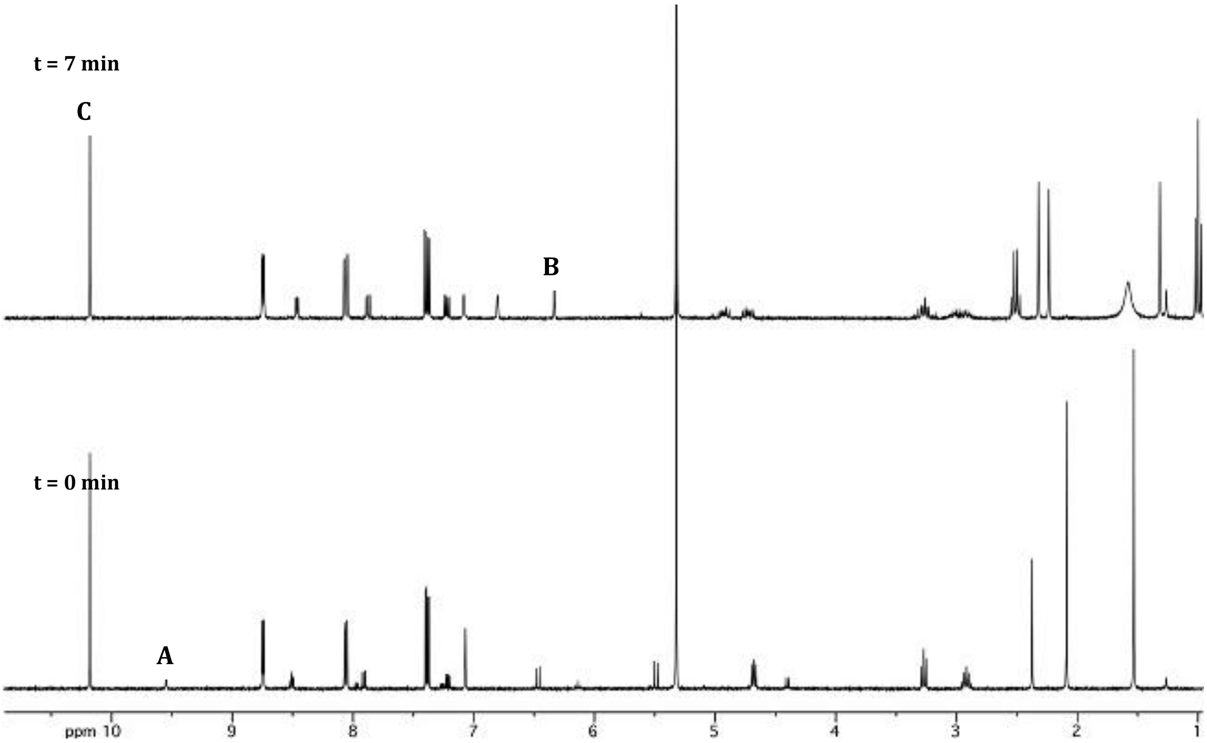

Figure S27. Representative ^{1}H NMR spectra (500 MHz) for reaction of 6-bromo-2-pyridinecarboxaldehyde **19** (0.01 M) with *N*-Mes NHC precursor **10** (0.002 M) and Et_3N (0.002 M) in CD_2Cl_2 at 25 °C. A = NHC precursor NC*H*N, B = adduct C(α)*H*, C = ArC*H*O.

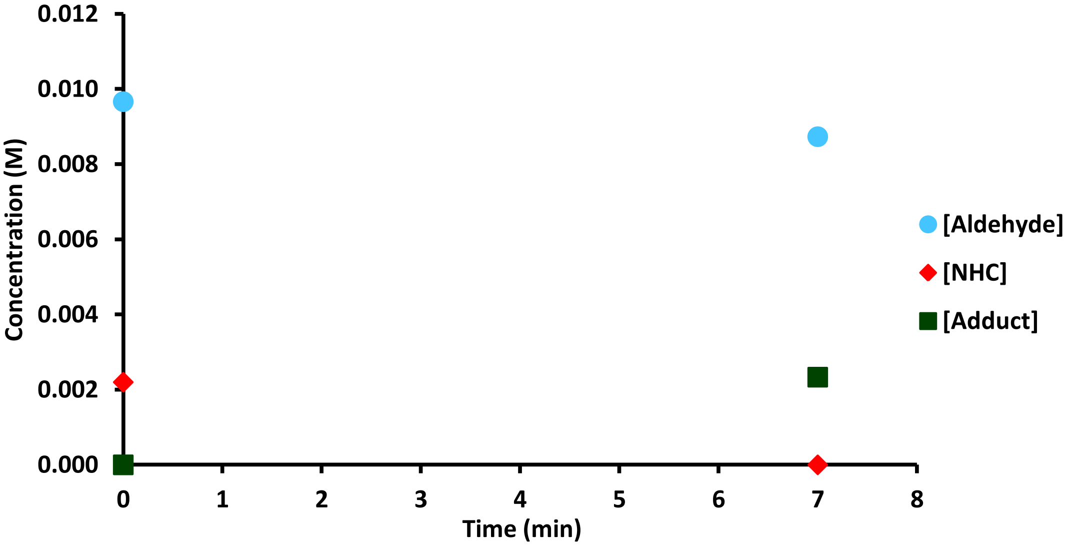

Figure S28. Reaction profile displaying concentration of species present against time for reaction of 6-bromo-2-pyridinecarboxaldehyde **19** (0.01 M) with *N*-Mes NHC precursor **10** (0.002 M) and Et_3N (0.002 M) in CD_2Cl_2 at 25 °C.

Isolation of 3-(Hydroxybenzyl)azolium Adducts 20-33 (Table 1)

General Procedure A: Synthesis of 3-(hydroxybenzyl)azolium salts

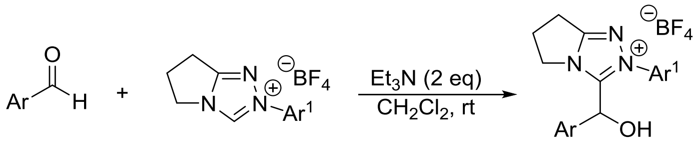

The appropriate NHC precatalyst (0.16 mmol, 1 eq) was suspended in CH_2Cl_2 (10 mL) and Et_3N (0.32 mmol, 2 eq) was added. The solution was stirred for 10 min before the appropriate aldehyde (0.16 mmol, 1 eq) was added and the reaction was stirred at rt for the time stated. The reaction mixture was concentrated *in vacuo* and the crude product was purified by column chromatography over silica (typically 20:80 acetone:CH_2Cl_2).

3-(Hydroxy(phenyl)methyl)-2-phenyl-6,7-dihydro-5*H*-pyrrolo[2,1-*c*][1,2,4]triazol-2-ium tetrafluoroborate 20

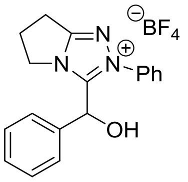

As previously reported.[4] Benzaldehyde **5** (186 µL, 1.83 mmol) was added to a solution of NHC precatalyst **9** (0.5 g, 1.83 mmol) and Et_3N (510 µL, 3.66 mmol) in CH_2Cl_2 (25 mL) was added. After stirring at rt for 3 h, the solution was washed once with aqueous 0.1 M HCl (30 mL) and concentrated under reduced pressure. The crude product was diluted with a small amount of methanol for purification by preparative LC-MS. The combined fractions were evaporated under reduced pressure to yield the title compound as a pale yellow oil (0.018 g, 3%). ν_{max} (neat): 1589, 1496, 1455, 1390, 1342, 1192, 1046, 764, 693; ^{1}H NMR (500 MHz, CD_3OD): δ_H 2.85 (2H, m, C*H$_2$*), 3.21 (2H, td, *J* 7.9, 2.8, C*H$_2$*), 4.44 (1H, dt, *J* 12.4, 7.5, C*H*H), 4.60 (1H, dt, *J* 12.4, 7.5, CH*H*), 6.21 (1H, s, C*H*), 7.19 (2H, d, *J* 7.7, Ar*H*), 7.28–7.34 (3H, m, Ar*H*), 7.46 (2H, d, *J* 8.3, Ar*H*), 7.53 (2H, t, *J* 7.8, Ar*H*), 7.61 (1H, t, *J* 7.5, Ar*H*); ^{13}C{^{1}H} NMR (125 MHz, CD_3OD): δ_C 22.3 (*C*H$_2$), 27.9 (*C*H$_2$), 50.1 (*C*H$_2$), 69.2 (*C*H), 127.1 (2 × Ar*C*H), 128.2 (2 × Ar*C*H), 130.1 (2 × Ar*C*H), 130.5 (Ar*C*H), 130.9 (2 × Ar*C*H), 132.6 (Ar*C*H), 136.7 (Ar*C*N), 137.9 (Ar*C*), 153.5 (N*C*N), 164.0 (N*C*N); *m/z* (ES+): 292 ([M–BF$_4$]$^+$, 100%); HRMS (ES+): [M–BF$_4$]$^+$ $C_{18}H_{18}N_3O$ requires 292.1450, found 292.1461.

3-(Hydroxy(2-methoxyphenyl)methyl)-2-phenyl-6,7-dihydro-5*H*-pyrrolo[2,1-*c*][1,2,4]triazol-2-ium tetrafluoroborate 21

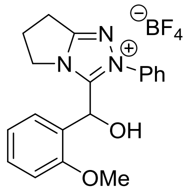

As previously reported.[4] 2-Methoxybenzaldehyde **2** (0.15 g, 1.10 mmol) was added to a solution of NHC precatalyst **9** (0.3 g, 1.10 mmol) and Et_3N (306 µL, 2.20 mmol) in CH_2Cl_2 (25 mL). After stirring at r.t. for 3 h, the solution was washed once with aqueous 0.1 M HCl (30 mL) and concentrated under reduced pressure. The crude product was purified by column chromatography (5:2 chloroform : hexane). The combined fractions were evaporated under reduced pressure to yield the title compound as an orange solid (0.12 g, 24%). mp 72–74 °C; ν_{max} (neat): 3124, 1599, 1491, 1466, 1395, 1287, 1245, 1046, 1019, 759, 695; ^{1}H NMR (600 MHz, CD_3OD): δ_H 2.74–2.89 (2H, m, C*H₂*), 3.20 (2H, t, *J* 8.2, C*H₂*), 3.59 (3H, s, C*H₃*), 4.15 (1H, m, C*H*H), 4.50 (1H, m, CH*H*), 6.29 (1H, s, C*H*), 6.93 (2H, dd, *J* 8.1, Ar*H*), 7.30 (1H, td, *J* 7.9, 1.7, Ar*H*), 7.38 (1H, dd, *J* 7.6, 1.3, Ar*H*), 7.53 (4H, d, *J* 4.2, Ar*H*), 7.56–7.61 (1H, m, Ar*H*); ^{13}C{^{1}H} NMR (151 MHz, CD_3OD): δ_C 22.4 (*C*H_2), 28.1 (*C*H_2), 49.3 (*C*H_2), 56.4 (*C*H_3), 64.2 (*C*H), 112.0 (Ar*C*H), 121.9 (Ar*C*H), 125.2 (Ar*C*), 126.8 (2 × Ar*C*H), 129.1 (Ar*C*H), 130.8 (2 × Ar*C*H), 132.0 (Ar*C*H), 132.4 (Ar*C*H), 136.9 (Ar*C*N), 153.4 (N*C*N), 157.3 (Ar*C*O), 163.6 (N*C*N); *m/z* (ES+): 323 ([M+H–BF_4]$^+$, 100%), 322 ([M–BF_4] $^+$, 40%); HRMS (ES+): [M–BF_4]$^+$ $C_{18}H_{17}N_3OF$ requires 322.1556, found 322.1566.

3-(Hydroxy(phenyl)methyl)-2-mesityl-6,7-dihydro-5*H*-pyrrolo[2,1-*c*][1,2,4]triazol-2-ium tetrafluoroborate 22

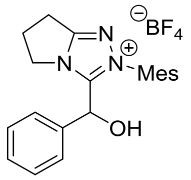

As previously reported.[4] General procedure A was employed, using triazolium salt **10** (500 mg, 1.83 mmol), benzaldehyde **5** (186 µL, 1.83mmol) and NEt_3 (510 µL, 3.66 mmol). After stirring at rt for 3 h the solution was washed once with aqueous 0.1 M HCl (30 mL) and concentrated *in vacuo*. The crude product was diluted with a small amount of methanol for purification by preparative LC-MS. The combined fractions were evaporated under reduced pressure to yield **22** as a brown oil (0.067 g, 9%). ν_{max} (neat) 3450, 1591, 1454, 1383, 1284, 1048, 854, 738, 698; ^{1}H NMR (500 MHz, CD_3OD) δ_H: 1.38 (3H, s, C*H₃*), 2.15 (3H, s, C*H₃*), 2.38 (3H, s, C*H₃*), 2.92 (2H, m, C*H₂*), 3.24 (2H, t, *J* 7.9, C*H₂*), 4.70 (1H, dt, *J* 12.4, 7.5, C*H*H), 4.78 (1H, dt, *J* 12.4, 7.5, CH*H*), 6.81 (1H, s, C*H*), 6.92 (1H, s, Ar*H*), 7.08 (2H, dd, *J* 7.9, 1.4, Ar*H*), 7.17 (1H, s, Ar*H*), 7.30 (2H, t, *J* 7.8, Ar*H*), 7.37 (1H, t, *J* 7.5, Ar*H*); ^{13}C{^{1}H}

NMR (126 MHz, CD_3OD) δ_C: 17.0 (*C*H_3), 17.4 (*C*H_3), 21.2 (*C*H_3), 22.5 (*C*H_2), 27.9 (*C*H_2), 50.5 (*C*H_2), 69.8 (*C*H), 127.3 (2 × Ar*C*H), 129.0 (2 × Ar*C*H), 129.6 (2 × Ar*C*H), 129.8 (Ar*C*H), 130.9 (Ar*C*), 135.0 (Ar*C*), 136.5 (Ar*C*), 142.7 (Ar*C*), 153.0 (N*C*N), 163.6 (N*C*N); *m/z* (ES+): 335 ($[M+H–BF_4]^+$, 100%), 334 ($[M–BF_4]^+$, 47%); HRMS (ES^+) $C_{21}H_{24}N_3O$ $[M–BF_4]^+$ found 334.1923, requires 334.1919.

3-(Hydroxy(2-methoxyphenyl)methyl)-2-mesityl-6,7-dihydro-5*H*-pyrrolo[2,1-*c*][1,2,4]triazol-2-ium tetrafluoroborate 23

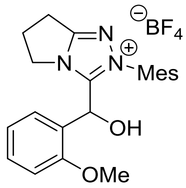

As previously reported.[4] General procedure A, using triazolium salt **10** (50.4 mg, 0.16 mmol), 2-methoxybenzaldehyde **2** (19.2 μL, 0.16 mmol) and NEt_3 (44.6 μL, 0.32 mmol) gave, with 15 min reaction time, **23** as a clear colourless oil (20 mg, 28%); ν_{max} (neat) 2926 (C-H), 1591 (C-O), 1492 (C=C), 1465 (C=C), 1440 (C=C), 1250 (C-O), 1087, 1070, 1020; ^1H NMR (400 MHz, CD_2Cl_2) δ_H: 1.61 (3H, s, ArC*H*$_3$), 1.80 (3H, s, ArC*H*$_3$), 2.32 (3H, s, ArC*H*$_3$), 2.83–2.96 (2H, m, NCH_2CH_2), 3.14–3.25 (2H, m, $NCH_2CH_2CH_2$), 3.67 (3H, s, OC*H*$_3$), 4.53 (1H, ddd, *J* 12.3, 8.4, 6.0, NCH_AH_B), 4.64 (1H, ddd, *J* 12.3, 8.4, 6.6, NCH_AH_B), 5.09 (1H, br d, *J* 4.2, O*H*), 6.06 (1H, br d, *J* 3.7, C(OH)(*H*)), 6.79–6.88 (3H, m, 4,6-Ar*H* and 3,5-NArC*H*), 6.90–6.95 (2H, m, 3-Ar*H* and 3,5-NArC*H*), 7.33 (1H, td, *J* 7.9, 1.5, 5-Ar*H*); $^{13}C\{^1H\}$ NMR (125 MHz, CD_2Cl_2) δ_C: 17.0 (Ar*C*H_3), 17.0 (Ar*C*H_3), 21.4 (Ar*C*H_3), 22.5 ($NCH_2CH_2CH_2$), 27.6 (NCH_2CH_2), 48.9 (N*C*H_2), 56.3 (O*C*H_3), 64.3 (*C*(H)(OH)), 111.0 (6-Ar*C*H), 121.6 (4-Ar*C*H), 123.3 (2-Ar*C*), 128.9 (3-Ar*C*H), 129.7 (3,5-NAr*C*H), 129.8 (3,5-NAr*C*H), 131.3 (4-NAr*C*), 131.6 (5-Ar*C*H), 136.2 (2,6-NAr*C*), 136.6 (2,6-NAr*C*), 142.5 (1-NAr*C*), 153.5 (N*C*N-Ar), 156.5 (1-Ar*C*), 162.2 (N*C*N); *m/z* (NSI^+) 364 ($[M–BF_4^-]^+$, 100%); HRMS (NSI^+) $C_{22}H_{26}N_3O_2$ $[M–BF_4^-]^+$ found 364.2013, requires 364.2020 (–1.8 ppm).

3-(Hydroxy(2-methoxyphenyl)methyl)-2-(2,4,6-trichlorophenyl)-6,7-dihydro-5*H*-pyrrolo[2,1-*c*][1,2,4]triazol-2-ium tetrafluoroborate 25

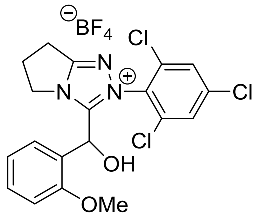

As previously reported.[4] General procedure A, using triazolium salt **11** (50 mg, 0.13 mmol), 2-methoxybenzaldehyde **2** (16.1 μL, 0.13 mmol) and NEt_3 (37.0 μL, 0.27 mmol) gave, with 2 min reaction time, **25** as a white solid (47 mg, 69%); mp 87–90 °C; υ_{max} (neat) 1600 (C-O), 1574 (C=C), 1559 (C=C), 1493 (C=C), 1251 (C-O), 1083, 1071, 914; ^{1}H NMR (400 MHz, CD_3OD) δ_H: 2.93 (2H, quintet, *J* 7.6, CH_2), 3.30–3.34 (2H, m, CH_2), 3.72 (3H, s, OCH_3), 4.62–4.74 (2H, m, CH_2), 6.25 (1H, s, C(OH)(*H*)), 6.88 (1H, td, *J* 7.5, 0.7, Ar*H*), 6.94 (1H, d, *J* 8.2, Ar*H*), 7.16 (1H, dd, *J* 7.6, 1.6, Ar*H*), 7.34–7.39 (1H, m, Ar*H*), 7.60 (1H, d, *J* 2.2, 2,4,6-$Cl_3C_6H_2$ Ar*H*), 7.81 (1H, d, *J* 2.2, 2,4,6-$Cl_3C_6H_2$ Ar*H*); ^{13}C{^{1}H} NMR (100 MHz, CD_3OD) δ_C: 22.5, 28.1, 50.5, 56.3, 65.8, 112.2, 122.1, 124.5, 130.1, 130.3, 130.5, 130.8, 132.8, 135.4, 136.4, 140.4, 156.4, 157.9, 164.8; *m/z* (NSI^+) 424 ([M–BF_4^-]$^+$, 100%); HRMS (NSI^+) $C_{19}H_{17}{}^{35}Cl_3N_3O_2$ [M–BF_4^-]$^+$ found 424.0374, requires 424.0381 (–1.6 ppm).

3-(Hydroxy(*o*-tolyl)methyl)-2-mesityl-6,7-dihydro-5*H*-pyrrolo[2,1-*c*][1,2,4]triazol-2-ium tetrafluoroborate 26

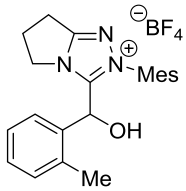

General procedure A, using triazolium salt **10** (81.9 mg, 0.262 mmol), 2-tolubenzaldehyde **12** (30.0 μL, 0.262mmol) and NEt_3 (72.5 μL, 0.524 mmol) gave, with 15 min reaction time, **26** as a white solid (79.2 mg, 70%). mp 68-75 °C; ν_{max} (neat) 2889 (C-H), 1591 (C-O), 1493 (C=C), 1452 (C=C), 1383 (C-O), 1285, 1271, 1084, 955, 845; ^{1}H NMR (500 MHz, CD_2Cl_2) δ_H: 1.10 (3H, s, NArCH_3), 1.69 (3H, s, ArCH_3), 2.14 (3H, s, NArCH_3), 2.31 (3H, s, NArCH_3), 2.90–3.05 (2H, m, NCH_2CH_2), 3.18–3.28 (2H, m, NCH_2CH_2CH_2), 4.71–4.77 (1H, m, NCH_AH_B), 4.96–5.02 (1H, m, NCH_AH_B), 6.04 (1H, s, C(OH)(*H*)), 6.74 (1H, s, 3,5-NArC*H*), 7.00 (1H, d, Ar*H*) 7.03–7.07 (2H, d, 3,5-NArC*H* and Ar*H*), 7.13 (1H, t, *J* 7.4, Ar*H*), 7.23 (1H, t, *J* 7.4, Ar*H*); ^{13}C{^{1}H} NMR (125 MHz, CD_2Cl_2) δ_C: 16.2 (NAr*C*H_3), 17.1 (NAr*C*H_3), 18.0 (NAr*C*H_3), 21.2 (Ar*C*H_3), 22.1 (NCH_2CH_2*C*H_2), 27.4 (NCH_2*C*H_2), 49.9 (N*C*H_2), 66.2 (*C*(H)(OH)), 127.2 (Ar*C*H), 128.4 (Ar*C*H), 129.7 (3,5-NAr*C*H), 130.1 (3,5-NAr*C*H), 130.2 (Ar*C*H), 130.6 (4-NAr*C*), 131.4 (Ar*C*H), 133.8 (Ar*C*), 135.8 (2,6-NAr*C*), 136.7 (2,6-

NAr*C*), 142.9 (1-NAr*C*), 154.6 (N*C*N-Ar), 162.8 (N*C*N); *m/z* (NSI^+) 348 ($[M–BF_4^-]^+$, 100%); HRMS (NSI^+) $C_{22}H_{26}N_3O$ $[M–BF_4^-]^+$ found 348.2078, requires 348.2070 (+2.2 ppm).

(*E*)-3-((2-((4-Ethoxy-4-oxobut-2-en-1-yl)oxy)phenyl)(hydroxy)methyl)-2-mesityl-6,7-dihydro-5*H*-pyrrolo[2,1-*c*][1,2,4]triazol-2-ium tetrafluoroborate 27

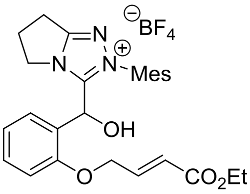

As previously reported.[4] General procedure A, using triazolium salt **10** (170 mg, 0.54 mmol), aldehyde **13** (127 mg, 0.54 mmol) and NEt_3 (150 µL, 1.08 mmol) gave, with 30 min reaction time, **27** as a white solid (220 mg, 74%). mp 57–59 °C; ν_{max} (KBr) 2927 (C-H), 1718 (C=O), 1593 (C=C), 1496 (C=C), 1456, 1306 (C-H), 1050 (C-O); ^{1}H NMR (400 MHz, d_6-DMSO) δ_H: 1.25 (3H, t, *J* 7.1, C*H*$_3$), 1.35 (3H, s, ArC*H*$_3$), 1.99 (3H, s, ArC*H*$_3$), 2.25 (3H, s, ArC*H*$_3$), 2.75–2.86 (2H, m, NCH$_2$C*H*$_2$), 3.19 (2H, t, *J* 7.8, NCH$_2$CH$_2$C*H*$_2$), 4.18 (2H, qd, *J* 7.1, 4.6, C*H*$_2$CH$_3$), 4.35 (1H, ddd, *J* 17.1, 4.2, 2.0, OC*H*$_A$H$_B$CH), 4.58 (2H, t, *J* 7.4, C*H*$_2$), 4.67 (1H, ddd *J* 16.1, 4.2, 2.0, OCH$_A$*H*$_B$CH), 5.91 (1H, dt, *J* 15.8, 1.9, CHC*H*C=O), 6.14 (1H, d, *J* 5.1, C(OH)(*H*)), 6.82–6.88 (2H, m, C*H*CHC=O and 4-Ar*H*), 6.89–6.95 (2H, m, 6-Ar*H* and 3,5-NAr*H*), 6.98 (1H, br s, 3,5-NAr*H*), 7.20 (1H, d, *J* 5.2, O*H*), 7.24 (1H, dd, *J* 7.6, 1.6, 3-Ar*H*), 7.35 (1H, ddd, *J* 8.3, 7.4, 1.6, 5-Ar*H*); ^{13}C{^{1}H} NMR (100 MHz, d_6-DMSO) δ_C: 14.2 (*C*H$_2$*C*H$_3$), 16.1 (Ar*C*H$_3$), 16.8 (Ar*C*H$_3$), 20.7 (Ar*C*H$_3$), 21.5 (NCH$_2$CH$_2$*C*H$_2$), 26.7 (NCH$_2$*C*H$_2$), 48.9 (N*C*H$_2$), 60.3 (*C*H$_2$CH$_3$), 61.7 (*C*(H)(OH)), 66.4 (O*C*H$_2$CH), 111.9 (6-Ar*C*H), 121.2 (CH*C*HC=O), 121.2 (4-Ar*C*H), 124.2 (2-Ar*C*), 128.9 (3,5-NAr*C*H), 129.3 (3,5-NAr*C*H), 129.5 (3-Ar*C*H), 130.5 (4-NAr*C*), 131.0 (5-Ar*C*H), 134.8 (2,6-NAr*C*), 135.7 (4-NAr*C*), 141.3 (C*H*CHC=O), 152.9 (N*C*N-Ar), 154.2 (1-Ar*C*), 162.8 (N*C*N), 165.2 (*C*=O); *m/z* (NSI^+) 462 ($[M–BF_4^-]^+$, 100%); HRMS (NSI^+) $C_{27}H_{32}N_3O_4$ $[M–BF_4^-]^+$ found 462.2383, requires 462.2387 (–0.9 ppm).

3-((2-Bromophenyl)(hydroxy)methyl)-2-mesityl-6,7-dihydro-5*H*-pyrrolo[2,1-*c*][1,2,4]triazol-2-ium tetrafluoroborate 28

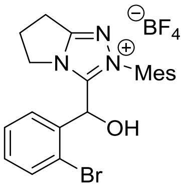

General procedure A, using triazolium salt **10** (100 mg, 0.317 mmol), 2-bromobenzaldehyde **14** (37.0 µL, 0.317 mmol) and NEt_3 (88.4 µL, 0.634 mmol) gave, with 15 min reaction time, **28** as a white solid (100 mg, 63%). mp 69–72 °C; ν_{max} (neat) 2972 (C-H), 1589 (C-O), 1494 (C=C), 1471 (C=C), 1435

(C=C), 1282 (C-O), 1082, 1068, 956; ^{1}H NMR (500 MHz, CD_2Cl_2) δ_H: 1.71 (3H, s, ArC*H*$_3$), 1.74 (3H, s, ArC*H*$_3$), 2.30 (3H, s, ArC*H*$_3$), 2.89–3.02 (2H, m, NCH_2CH_2), 3.20–3.30 (2H, m, $NCH_2CH_2CH_2$), 4.73–4.85 (2H, m, NCH_2), 6.25 (1H, s, C(OH)(*H*)), 6.80 (1H, s, 3,5-NArC*H*), 6.84 (1H, s, 3,5-NArC*H*), 6.99 (1H, dd, *J* 7.8, 1.5, 3-Ar*H*), 7.11 (1H, td, *J* 7.6, 1.0, 4-Ar*H*), 7.20 (1H, td, *J* 7.7, 1.6, 5-Ar*H*), 7.47 (1H, dd, *J* 8.0, 1.1, 6-Ar*H*); ^{13}C{^{1}H} NMR (125 MHz, CD_2Cl_2) δ_C: 17.1 (Ar*C*H$_3$), 17.3 (Ar*C*H$_3$), 21.4 (Ar*C*H$_3$), 22.6 ($NCH_2CH_2CH_2$), 27.5 (NCH_2CH_2), 49.4 (N*C*H$_2$), 67.6 (*C*(H)(OH)), 122.5 (2-Ar*C*), 128.5 (4-Ar*C*H), 129.8 (3,5-NAr*C*H), 129.9 (3,5-NAr*C*H), 130.2 (3-Ar*C*H), 131.1 (4-NAr*C*), 131.6 (5-Ar*C*H), 133.3 (6-Ar*C*H), 134.4 (1-Ar*C*), 136.0 (2,6-NAr*C*), 136.8 (2,6-NAr*C*), 142.7 (1-NAr*C*), 152.4 (N*C*N-Ar), 162.7 (N*C*N); *m/z* (NSI$^+$) 412 ([M(^{79}Br)–BF_4^-]$^+$, 100%); HRMS (NSI$^+$) $C_{21}H_{23}{}^{79}BrN_3O$ [M(^{79}Br)–BF_4]$^+$ found 412.1009, requires 412.1019 (–2.4 ppm).

3-((4-Bromophenyl)(hydroxy)methyl)-2-mesityl-6,7-dihydro-5*H*-pyrrolo[2,1-*c*][1,2,4]triazol-2-ium tetrafluoroborate 29

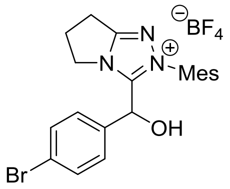

General procedure A, using triazolium salt **10** (100 mg, 0.317 mmol), 4-bromobenzaldehyde **15** (59.1 μL, 0.317 mmol) and NEt_3 (88.4 μL, 0.634 mmol) gave, with 30 min reaction time, **29** as a white solid (85.0 mg, 54%). mp 77–82 °C; ν_{max} (neat) 1709 (C-O), 1591 (C=C), 1487 (C=C), 1451 (C=C), 1285 (C-O), 1079, 1010; ^{1}H NMR (500 MHz, CD_3OD) δ_H: 1.47 (3H, s, ArC*H*$_3$), 2.11 (3H, s, ArC*H*$_3$), 2.37 (3H, s, ArC*H*$_3$), 2.88–2.94 (2H, m, NCH_2CH_2), 3.22–3.25 (2H, m, $NCH_2CH_2CH_2$), 4.65–4.76 (2H, m, NCH_2), 5.84 (1H, s, C(OH)(*H*)), 6.93 (1H, s, 3,5-NArC*H*), 7.01–7.03 (2H, m 3,5-Ar*H*), 7.13 (1H, s, 3,5-NArC*H*), 7.44-7.46 (2H, m 2,6-Ar*H*); ^{13}C{^{1}H} NMR (125 MHz, CD_3OD) δ_C: 17.1 (Ar*C*H$_3$), 17.2 (Ar*C*H$_3$), 21.2 (Ar*C*H$_3$), 22.4 ($NCH_2CH_2CH_2$), 27.8 (NCH_2CH_2), 50.5 (N*C*H$_2$), 69.2 (*C*(H)(OH)), 124.8 (4-Ar*C*), 130.3 (3,5-Ar*C*H), 130.7 (3,5-NAr*C*H), 130.8 (3,5-NAr*C*H), 132.0 (4-NAr*C*), 133.1 (2,6-Ar*C*H), 136.2 (2,6-NAr*C*), 136.8 (1-Ar*C*), 137.4 (2,6-NAr*C*), 143.9 (1-NAr*C*), 153.4 (N*C*N-Ar), 164.9 (N*C*N); *m/z* (NSI$^+$) 412 ([M(^{79}Br)–BF_4^-]$^+$, 100%); HRMS (NSI$^+$) $C_{21}H_{23}{}^{79}BrN_3O$ [M(^{79}Br)–BF_4^-]$^+$ found 412.1014, requires 412.1019 (–1.2 ppm).

3-((2-Fluorophenyl)(hydroxy)methyl)-2-mesityl-6,7-dihydro-5*H*-pyrrolo[2,1-*c*][1,2,4]triazol-2-ium tetrafluoroborate 30

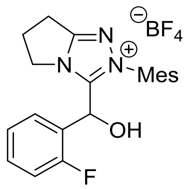

General procedure A, using triazolium salt **10** (81.9 mg, 0.262 mmol), 2-fluorobenzaldehyde **16** (27.6 μL, 0.262 mmol) and NEt_3 (72.5 μL, 0.524 mmol) gave, with 15 min reaction time, **30** as a white solid (43.4 mg, 37%). mp 148–154 °C; ν_{max} (neat) 2966 (C-H), 1592 (C-O), 1491 (C=C), 1448 (C=C), 1435 (C=C), 1289 (C-O), 1223, 1091, 1048, 990; ^{1}H NMR (500 MHz, CD_2Cl_2) δ_H: 1.44 (3H, s, ArCH_3), 1.96 (3H, s, ArCH_3), 2.33 (3H, s, ArCH_3), 2.85–3.00 (2H, m, NCH_2CH_2), 3.17–3.28 (2H, m, $NCH_2CH_2CH_2$), 4.63–4.68 (1H, m, NCH_AH_B), 4.78–4.83 (1H, m, NCH_AH_B), 6.03 (1H, s, C(OH)(*H*)), 6.80 (1H, s, 3,5-NArC*H*), 6.89 (1H, td, *J* 7.5, 1.6, Ar*H*), 7.00 (1H, s, 3,5-NArC*H*), 7.01–7.05 (2H, m, Ar*H*), 7.35–7.40 (1H, m, Ar*H*); ^{19}F{^{1}H} NMR (470 MHz, CD_2Cl_2) δ_F: –152.5 (BF_4), –152.4 (BF_4), –118.4 (C*F*); ^{13}C{^{1}H} NMR (125 MHz, CD_2Cl_2) δ_C: 16.8 (ArCH$_3$), 17.1 (ArCH$_3$), 21.5 (ArCH$_3$), 22.4 ($NCH_2CH_2CH_2$), 27.5 (NCH_2CH_2), 49.5 (d, *J* 3.2, NCH_2), 64.7 (d, *J* 2.5, *C*(H)(OH)), 116.2 (d, *J* 20.9, Ar*C*H), 122.7 (d, *J* 13.2, Ar*C*), 125.4 (d, *J* 3.1, Ar*C*H), 129.9 (3,5-NAr*C*H), 130.0 (d, *J* 3.1, Ar*C*H), 130.8 (4-NAr*C*), 132.5 (d, *J* 8.7, Ar*C*H), 135.8 (2,6-NAr*C*), 136.5 (2,6-NAr*C*), 143.0 (1-NAr*C*), 152.9 (N*C*N-Ar), 160.6 (d, *J* 247.7, Ar*C*), 162.8 (N*C*N); *m/z* (NSI$^+$) 352 ([M–BF_4^-]$^+$, 100%); HRMS (NSI$^+$) $C_{21}H_{23}N_3OF$ [M–BF_4^-]$^+$ found 352.1819, requires 352.1820 (–0.2 ppm).

3-((2,6-Difluorophenyl)(hydroxy)methyl)-2-mesityl-6,7-dihydro-5*H*-pyrrolo[2,1-*c*][1,2,4]triazol-2-ium tetrafluoroborate 31

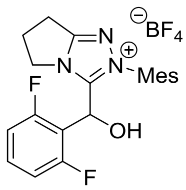

General procedure A, using triazolium salt **10** (81.9 mg, 0.262 mmol), 2,6-difluorobenzaldehyde **17** (28.3 μL, 0.262 mmol) and NEt_3 (72.5 μL, 0.524 mmol) gave, with 15 min reaction time, **31** as a white solid (84.8 mg, 71%). mp 160–163 °C; ν_{max} (neat) 2899 (C-H), 1652 (C-O), 1592 (C=C), 1469 (C=C), 1290 (C-O), 1126, 1099, 993, 999; ^{1}H NMR (500 MHz, CD_2Cl_2) δ_H: 1.25 (3H, s, ArCH_3), 2.20 (3H, s, ArCH_3), 2.31 (3H, s, ArCH_3), 2.84–2.92 (1H, m, $NCH_2CH_AH_B$), 2.95–3.04 (1H, m, $NCH_2CH_AH_B$), 3.17–3.30 (2H, m, $NCH_2CH_2CH_2$), 4.71 (1H, ddd, *J* 5.7, 8.6, 12.8, NCH_AH_B), 4.98 (1H, ddd, *J* 5.7, 8.6, 12.8, NCH_AH_B), 6.25 (1H, s, C(OH)(*H*)), 6.69 (1H, s, 3,5-NArC*H*), 6.81 (2H, t, *J* 8.4, Ar*H*), 7.07 (1H, s, 3,5-NArC*H*), 7.35 (1H, tt, *J* 6.6, 8.5 Ar*H*); ^{19}F{^{1}H} NMR (470 MHz, CD_2Cl_2)

δ_F: –152.6 (BF_4), –152.5 (BF_4), –115.3 (CF); ^{13}C{^{1}H} NMR (125 MHz, CD_2Cl_2) δ_C: 16.4 (ArCH_3), 17.3 (ArCH_3), 21.4 (ArCH_3), 22.3 ($NCH_2CH_2CH_2$), 27.6 (NCH_2CH_2), 50.1 (d, *J* 3.2, NCH_2), 60.1 (t, *J* 3.7, *C*(H)(OH)), 112.4 (dd, *J* 3.8, 20.9, Ar*C*H), 129.7 (3,5-NAr*C*H), 130.2 (NAr*C*), 130.5 (3,5-NAr*C*H), 133.2 (t, *J* 10.7, Ar*C*H), 135.8 (d, *J* 8.5, Ar*C*), 143.1 (1-NAr*C*), 153.2 (N*C*N-Ar), 161.0 (dd, *J* 252.1, 6.7 Ar*C*F), 163.0 (N*C*N); *m/z* (NSI^+) 370 ($[M–BF_4^-]^+$, 100%); HRMS (NSI^+) $C_{21}H_{22}N_3OF_2$ $[M–BF_4^-]^+$ found 370.1722, requires 370.1725 (–0.9 ppm).

3-((3-Bromopyridin-2-yl)(hydroxy)methyl)-2-mesityl-6,7-dihydro-5*H*-pyrrolo[2,1-*c*][1,2,4]tri-azol-2-ium tetrafluoroborate 33

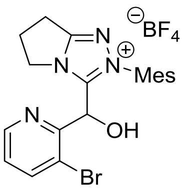

General procedure A, using triazolium salt **10** (81.9 mg, 0.262 mmol), 3-bromopyridine-2-carboxaldehyde **19** (48.4 µL, 0.262 mmol) and NEt_3 (72.5 µL, 0.524 mmol) gave, with 10 min reaction time, **33** as a white solid (75.6 mg, 58%). mp 118–125 °C; ν_{max} (neat) 2993 (C-H), 1595 (C-O), 1502 (C=C), 1433 (C=C), 1386 (C=C), 1283 (C-O), 1084, 1078, 1012; ^{1}H NMR (400 MHz, CD_2Cl_2) δ_H: 1.31 (3H, s, ArCH_3), 2.24 (3H, s, ArCH_3), 2.32 (3H, s, ArCH_3), 2.85–3.05 (2H, m, NCH_2CH_2), 3.19-3.34 (2H, m, $NCH_2CH_2CH_2$), 4.74 (1H, ddd, *J* 5.8, 8.8, 12.5, NCH_AH_B), 4.91 (1H, ddd, *J* 6.4, 8.7, 12.5, NCH_AH_B), 6.32 (1H, s, C(OH)(*H*)), 6.80 (1H, s, 3,5-NArC*H*), 7.08 (1H, s, 3,5-NArC*H*), 7.23 (1H, dd, *J* 4.6, 8.2, Ar*H*), 7.87 (1H, dd, *J* 1.4, 8.2, Ar*H*), 8.47 (1H, dd, *J* 1.4, 4.6, Ar*H*); ^{13}C{^{1}H} NMR (100 MHz, CD_2Cl_2) δ_C: 16.9 (ArCH_3), 17.6 (ArCH_3), 21.5 (ArCH_3), 22.5 ($NCH_2CH_2CH_2$), 27.5 (NCH_2CH_2), 49.4 (NCH_2), 68.4 (*C*(H)(OH)), 121.4 (Ar*C*), 126.6 (Ar*C*H), 130.1 (3,5-NAr*C*H), 130.5 (4-NAr*C*), 130.6 (3,5-NAr*C*H), 136.1 (2,6-NAr*C*), 136.2 (2,6-NAr*C*), 142.2 (Ar*C*H), 143.1(1-NAr*C*), 149.1 (Ar*C*H), 153.2 (Ar*C*), 153.3 (N*C*N-Ar), 163.4 (N*C*N); *m/z* (NSI^+) 413 ($[M(^{79}Br)–BF_4^-]^+$, 100%); HRMS (NSI^+) $C_{20}H_{22}{}^{79}BrN_4O$ $[M(^{79}Br)–BF_4^-]^+$ found 413.0968, requires 413.0972 (–0.8 ppm).

Determination of Rate and Equilibrium Constants for 3-(Hydroxybenzyl)azolium Adduct Formation in CD_3OD (Table 2, Table S1)

General Experimental Procedure

In an NMR tube, aldehyde **13** (30 mmol) and the appropriate NHC precatalyst (30 mmol) were dissolved in CD_3OD (650 µL). The reaction was initiated by the addition of 100 µL of a solution of NEt_3 (0.795 M) and $Et_3N{\cdot}HCl$ (0.405 M) in CD_3OD. This gave an overall aldehyde and NHC concentration of 0.04 M and a total buffer concentration of 0.16 M. The reaction was monitored by ^1H NMR spectroscopy on a Bruker Avance 500 MHz NMR spectrometer, with the probe set at 15 or 25 °C. Spectra were taken at 5 min intervals for the first hour, followed by 20 min intervals over the next ~16 hours.

Over the course of the reaction it was possible to observe the changes in concentrations of NHC, aldehyde **13**, the corresponding 3-(hydroxybenzyl)azolium adduct and the Stetter product. It was also possible to observe formation of deuterated 3-(hydroxybenzyl)azolium salts (D-adducts), formed *via* deuteration of the Breslow intermediate. In all cases adduct formation was fast relative to product formation.

Aldehyde-Methanol Adduct Equilibrium

In methanol, aldehyde **13** exists in equilibrium with hemiacetal **S4** (Scheme S1) that can be observed under the reaction conditions.

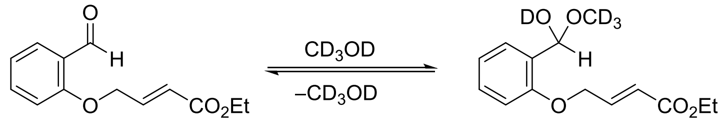

Scheme S1. Aldehyde-hemiacetal equilibrium.

The equilibrium constant for this process, K^{hem}, is described by Equation 1. A value for K^{hem} of 0.121 was determined for the reaction conditions using the integral of the hemiacetal CH singlet at 5.88 ppm divided by the singlet 10.47 ppm, corresponding to aldehyde **13**. The fraction of aldehyde present at equilibrium, f_{ald}, is described by Equation 2, giving a value for f_{ald} of 0.892 for aldehyde **13** under the reaction conditions. To enable accurate estimation of aldehyde species present in the reaction, a correction was made using the calculated f_{ald} value.

$$K^{hem} = \frac{[\text{hemiacetal}]}{[\text{aldehyde}]} \quad \text{(Eq 1)}$$

$$f_{ald} = \frac{[\text{aldehyde}]}{([\text{hemiacetal}] + [\text{aldehyde}])} \quad \text{(Eq 2)}$$

Table 2 Entry 1, Table S1 Entry 6

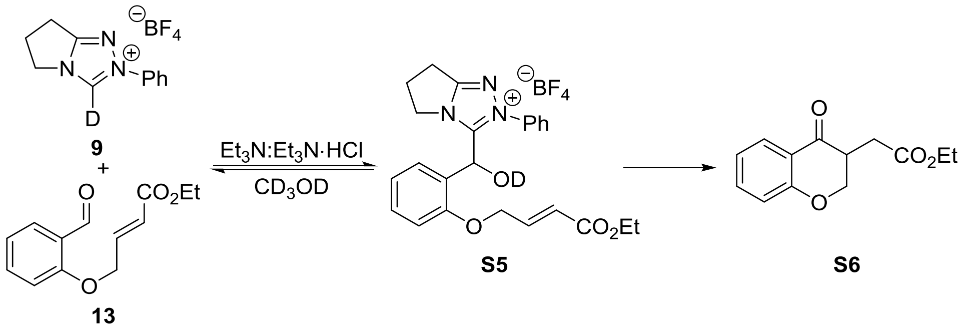

Determination of Concentration

Using the general procedure described above, the intramolecular Stetter reaction of aldehyde **13** and triazolium precatalyst **9** was monitored using ^{1}H NMR, with representative NMR spectra over the course of the experiment and spectra of starting materials and intermediates given in Figures S29 and S30.

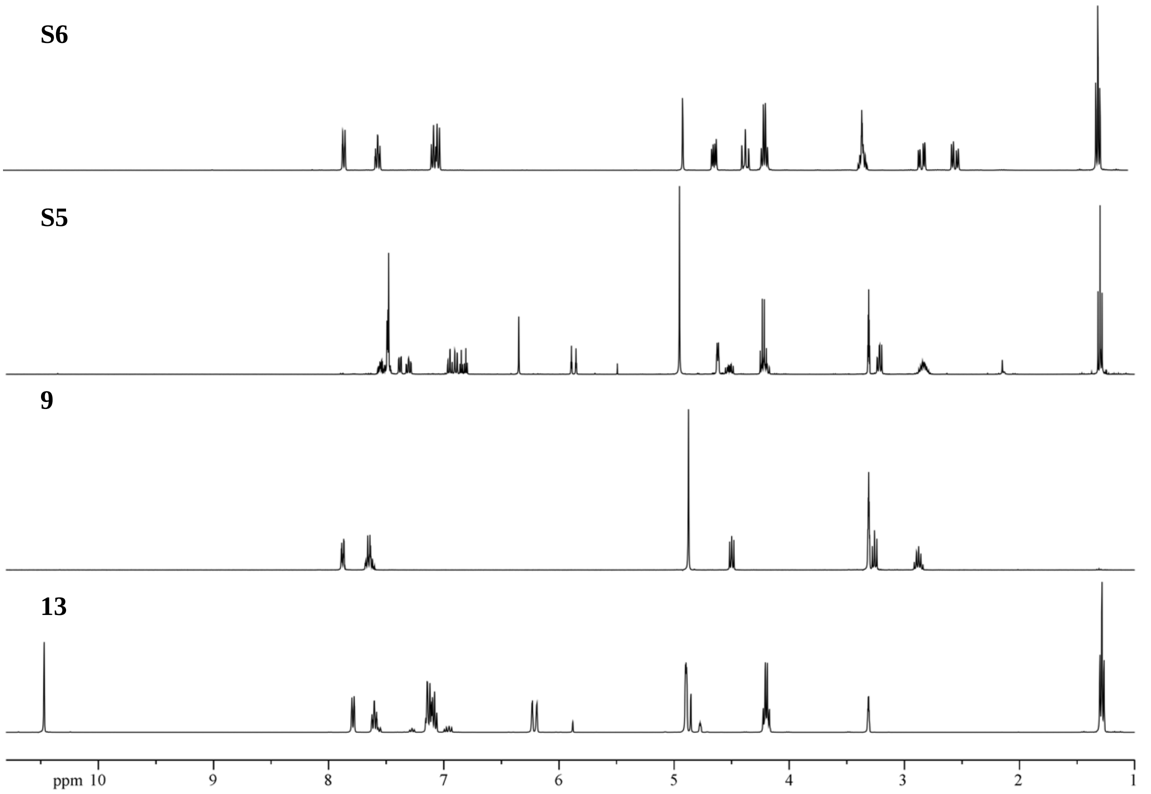

Figure S29. ^{1}H NMR spectra of aldehyde **13**, NHC precursor **9**, adduct **S5** and product **S6** in CD_3OD.

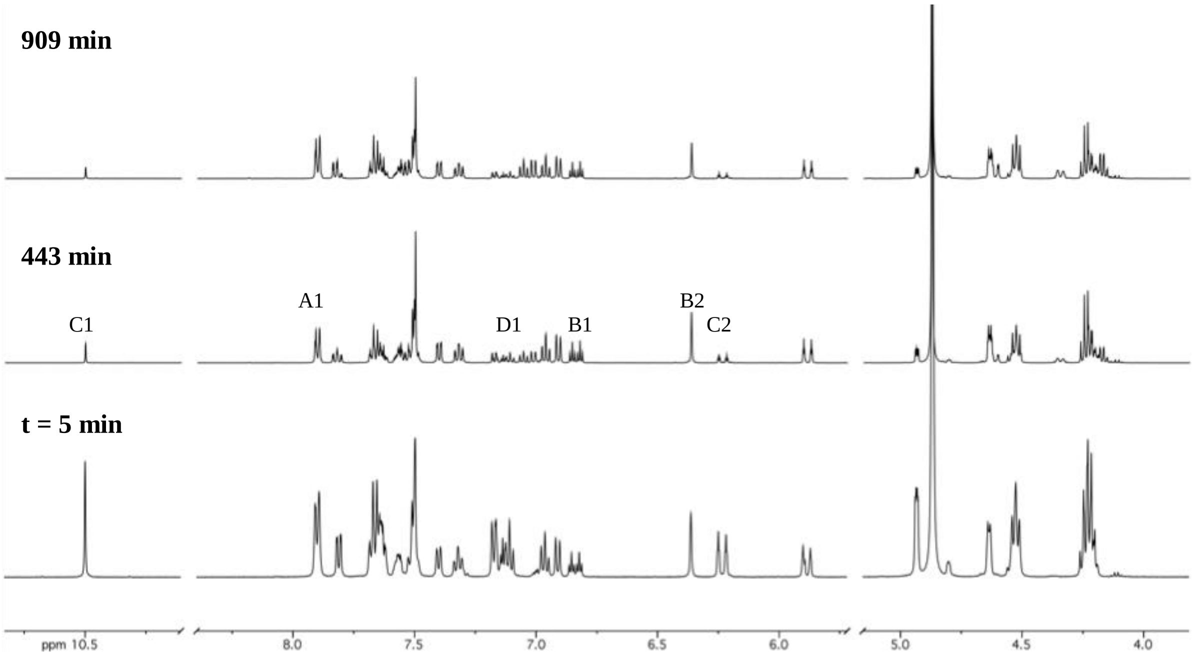

Figure S30. Representative ^{1}H NMR spectra for the intramolecular Stetter reaction of aldehyde **13** with triazolium salt **9** in Et_3N:$Et_3N{\cdot}HCl$ and CD_3OD at 25 °C.

The concentration of NHC precursor was determined using the peak at 7.89 ppm (A1), which corresponds to two *ortho*-aromatic protons on the phenyl ring. It was possible to observe the peaks for the other three aromatic protons of the *N*-Ph substituent in the multiplet at 7.60-7.68. However, these peaks were not used to calculate NHC precursor concentration as they overlapped with signals for the hemiacetal form of aldehyde **13**. Signals corresponding to the CH_2 groups of the five membered ring at 2.88, 3.25 and 4.62 ppm were not used as they overlapped with the corresponding protons on the adduct **S5**.

Over the course of the reaction, the build up of adduct **S5** was observed, the total quantity of which was defined using the doublet of triplets at 6.82 ppm (B1), corresponding to the β-CH of the alkene of **S5**. It was also possible to use aromatic signals at 6.90, 6.95, 7.31 and 7.39 ppm, which gave comparable results.

In order to convert the relative integral values to concentrations of the species present, the total of the integrals for A1 and B1 were used to give the total amount of triazolium containing species present in both free catalyst and adduct forms (Equations 3 and 4).

$$[\mathrm{catalyst}] = \frac{(A_{\mathrm{A1}}/2)}{(A_{\mathrm{B1}} + (A_{\mathrm{A1}}/2))} \times 0.04 \qquad \text{(Eq 3)}$$

$$[\mathrm{adduct}]_{\mathrm{tot}} = \frac{(A_{\mathrm{B1}})}{(A_{\mathrm{B1}} + (A_{\mathrm{A1}}/2))} \times 0.04 \qquad \text{(Eq 4)}$$

The signal for the C(α)-H of adduct **S5** fell at 6.35 ppm (B2), which allowed determination of the protonated form ([H-adduct]) using Equation 5 Thus the concentration of D-adduct could be obtained from the difference between the concentrations of total and protonated adduct species present (Equation 6).

$$[\text{H-adduct}]_{\text{tot}} = \frac{(A_{\text{B2}})}{(A_{\text{B1}} + (A_{\text{A1}}/2))} \times 0.04 \qquad \text{(Eq 5)}$$

$$[\text{D-adduct}] = [\text{adduct}]_{\text{tot}} - [\text{adduct}] \qquad \text{(Eq 6)}$$

From the singlet at 10.49 ppm (C1) and the doublet of triplets at 6.22 ppm (C2) it was possible to calculate the total concentration of protonated aldehyde **13** and total aldehyde using Equations 7 and 8, with a correction made for the fraction of aldehyde present in hemiacetal form, f_{ald}. In the case of C2, which was assigned to the CH α to the ester group, close inspection reveals the equivalent peaks for the hemiacetal to be contained within these signals, so no correction was required. Signals for the aromatic protons were also observed at 7.81 and 7.08 to 7.71 ppm. These peaks were not used to determine aldehyde concentration but did integrate approximately equally.

$$[\text{H-aldehyde}] = \frac{1}{f_{\text{ald}}} \times \frac{(A_{\text{C1}})}{(A_{\text{B1}} + (A_{\text{A1}}/2))} \times 0.04 \qquad \text{(Eq 7)}$$

$$[\text{aldehyde}]_{\text{tot}} = \frac{(A_{\text{C2}})}{(A_{\text{B1}} + (A_{\text{A1}}/2))} \times 0.04 \qquad \text{(Eq 8)}$$

The potential concentration of any deuterated aldehyde, formed by dissociation of D-adduct is given by Equation 9. However none was observed at detectable concentrations over the course of any experiments so this pathway could be disregarded.

$$[\text{D-aldehyde}] = [\text{aldehyde}]_{\text{tot}} - [\text{H-aldehyde}] \qquad \text{(Eq 9)}$$

The quantity of Stetter product **S6** was determined using the triplet at 7.04 ppm, D1 (Equation 10), corresponding to the aromatic CH at the 5 position of the aromatic ring.

$$[\text{product}] = \frac{(A_{\text{D1}})}{(A_{\text{B1}} + (A_{\text{A1}}/2))} \times 0.04 \qquad \text{(Eq 10)}$$

Assignments were confirmed by comparison with pure samples, with total concentrations corresponding to the initial 0.04 M concentrations of NHC precursor and aldehyde.

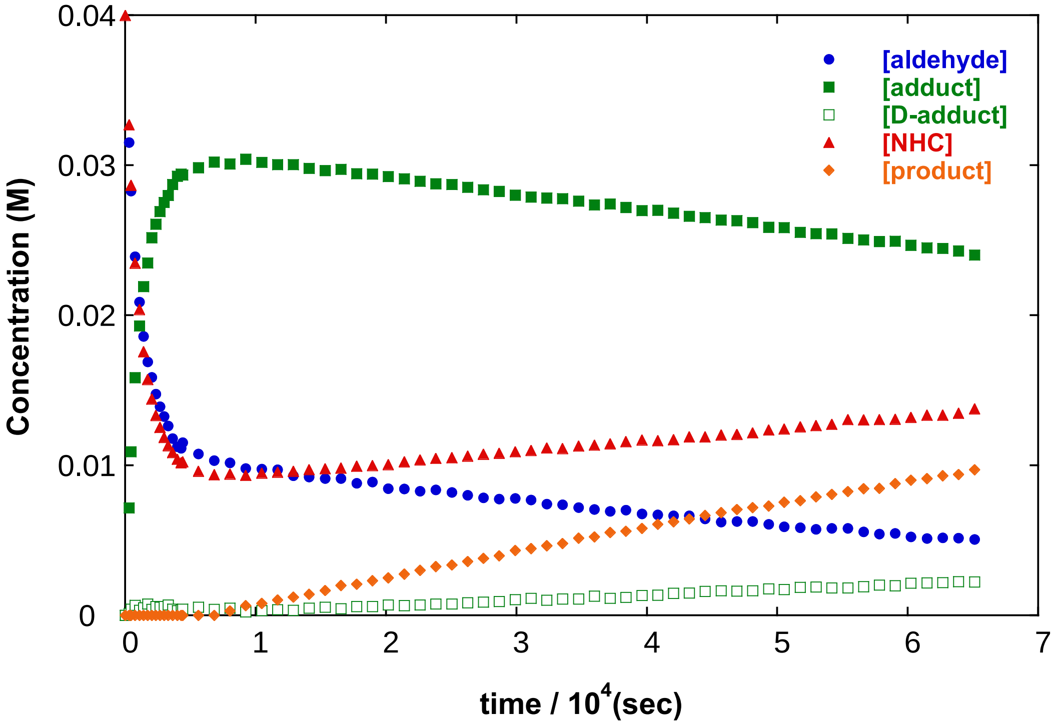

Figure S31. Reaction profile displaying concentration of species present against time for the intramolecular Stetter reaction using *N*-Ph NHC precursor **9** in Et_3N:$Et_3N \cdot HCl$ and CD_3OD at 15 °C.

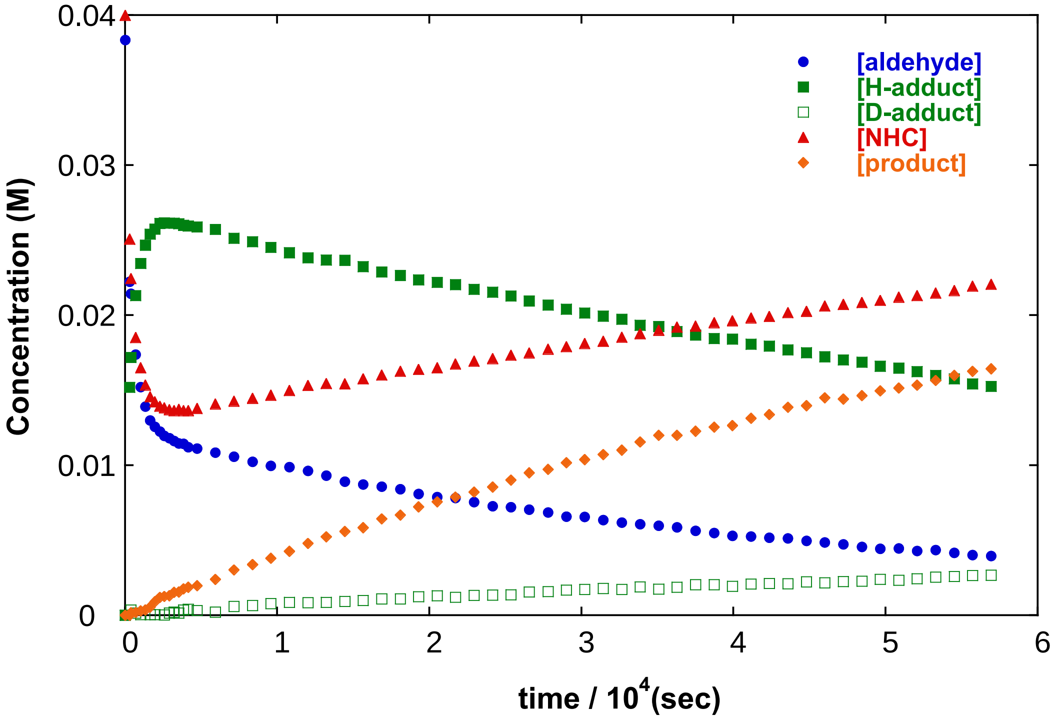

Figure S32. Reaction profile displaying concentration of species present against time for the intramolecular Stetter reaction using *N*-Ph NHC precursor **9** in Et_3N:$Et_3N \cdot HCl$ and CD_3OD at 25 °C.

In an attempt to obtain additional estimates for k_1 and k_{-1}, the data up to the formation of equilibrium concentrations of adduct were fitted using global fitting software (Berkeley Madonna version 8.3.18). The data was fitted to a kinetic model according to Equation 11.

$$\frac{d[\text{H-adduct}]}{dt} = k_1[\text{NHC}][\text{aldehyde}] - k_{-1}[\text{H-adduct}] \qquad \text{(Eq 11)}$$

In some cases, the starting aldehyde or NHC precursor concentration was adjusted to compensate in any error in making solution preparation to obtain the best fit. The adjustment was never greater than ±1 mM (±3%). In general, good visual agreement between the data points and the fitted line were observed.

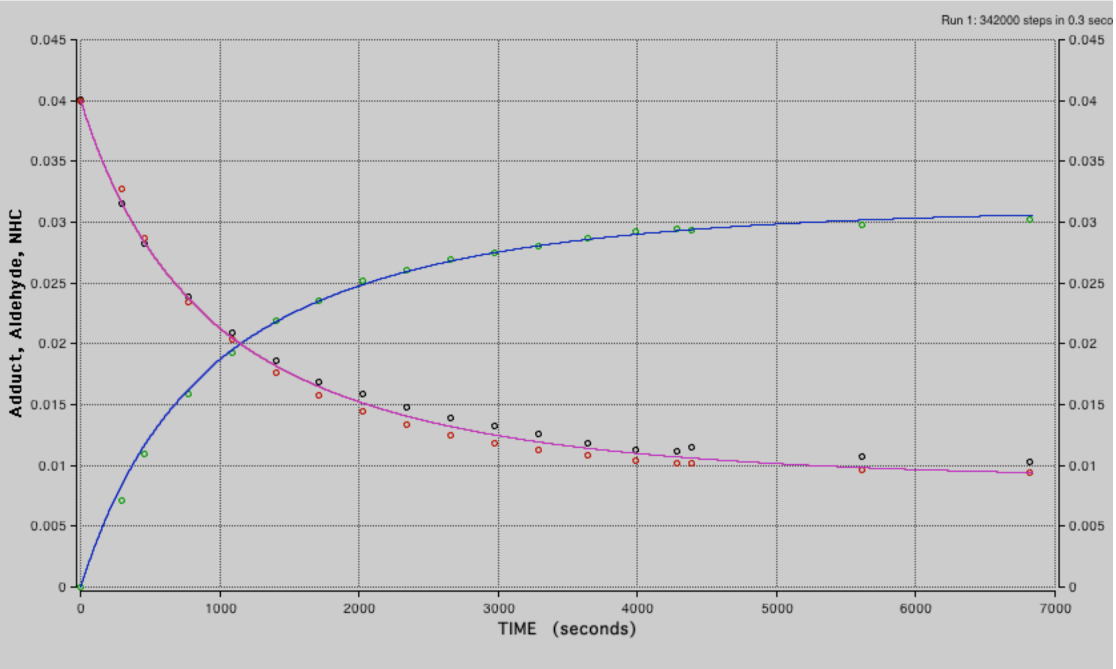

Figure S33. Plot showing formation of adduct during the Stetter reaction using *N*-Ph NHC precursor **9** at 15 °C, up to the equilibrium concentrations. Open circles show the experimental data, with the solid line representing the fit to the kinetic model. Fitting data from t = 0 to t = 7000 s from Figure S31.

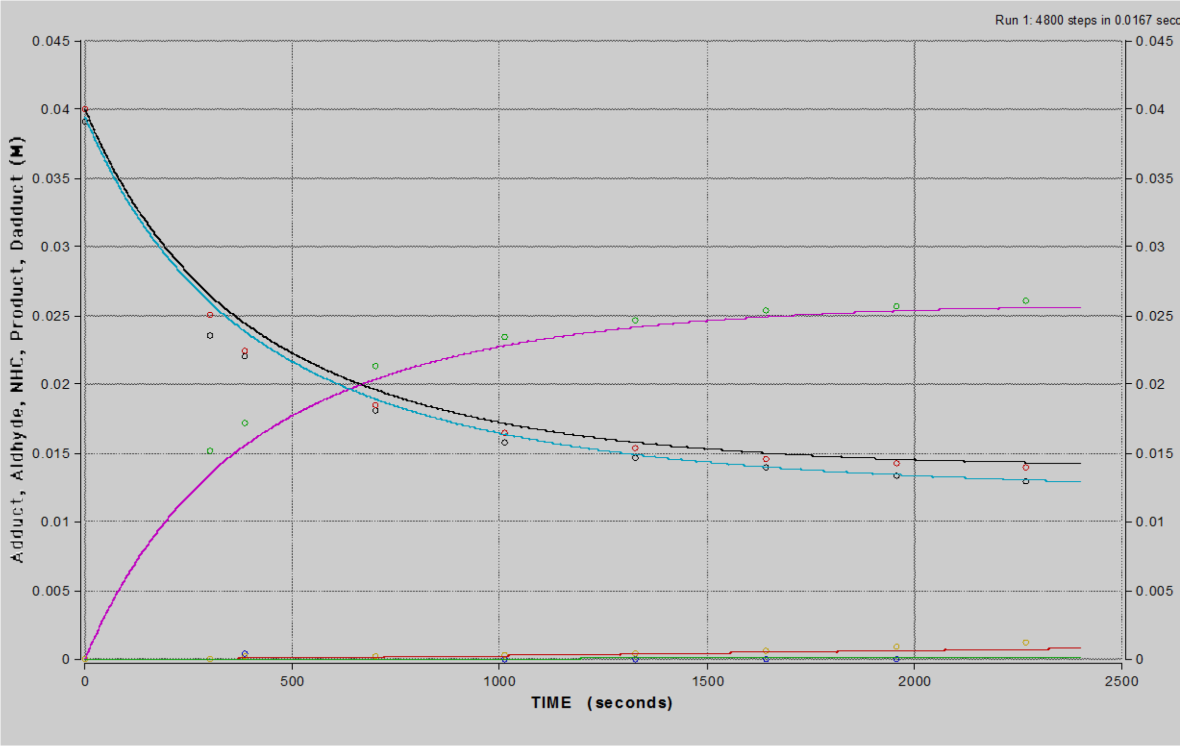

Figure S34. Plot showing formation of adduct during the Stetter reaction using *N*-Ph NHC precursor **9** at 25 °C, up to the equilibrium concentrations. Open circles show the experimental data, with the solid line representing the fit to the kinetic model. Fitting data from t = 0 to t = 2500 s from Figure S32.

Table 2 Entry 2, Table S1 Entry 7

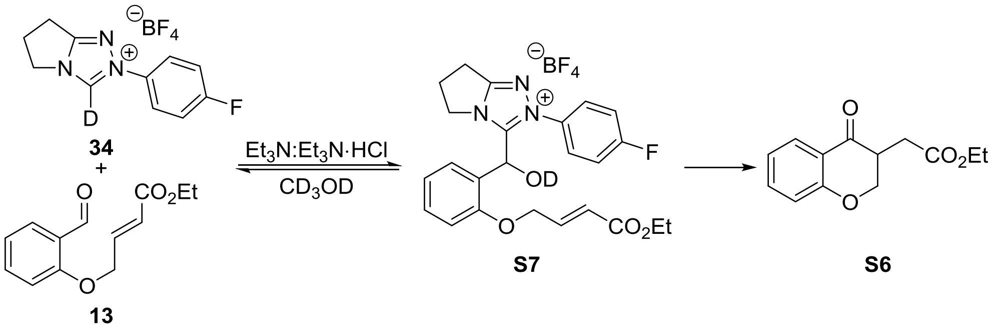

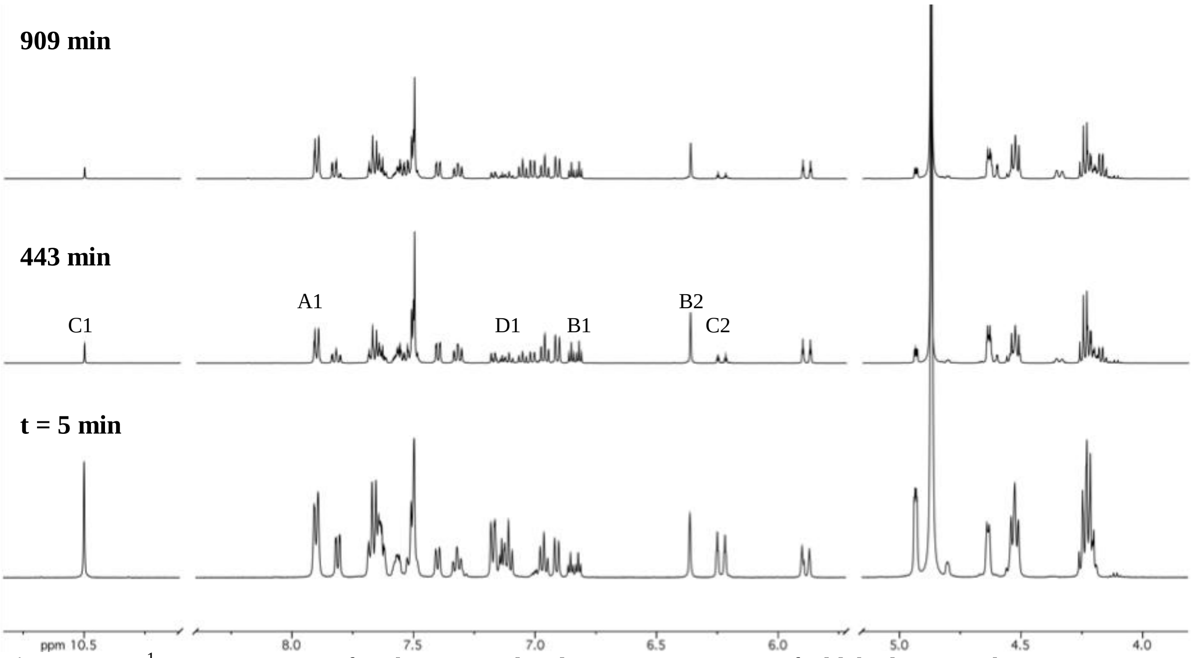

Figure S35. ^{1}H NMR spectra for the intramolecular Stetter reaction of aldehyde **13** with *N*-4-FC_6H_4 NHC precursor **34** in Et_3N:Et_3N·HCl and CD_3OD at 15 °C.

The concentration of NHC precursor was determined using the peak at 7.89 ppm (A1), which corresponds to two aromatic protons on the phenyl ring. From the singlet at 10.49 ppm (C1) it was possible to calculate the concentration of aldehyde **13**. The quantity of the C(α)-H 3-(hydroxybenzyl)azolium salt (H-adduct) was defined using the singlet at 6.42 ppm (B2), corresponding to the benzylic C(α)-H. The quantity of Stetter product **S6** was determined using the triplet at 7.04 ppm (D1), corresponding to the aromatic CH at the 5-position. Concentrations were determined as previously described using the Equations 3-10 and the data was fitted using global fitting software (Berkeley Madonna version 8.3.18) according to a kinetic model described by Equation 11.

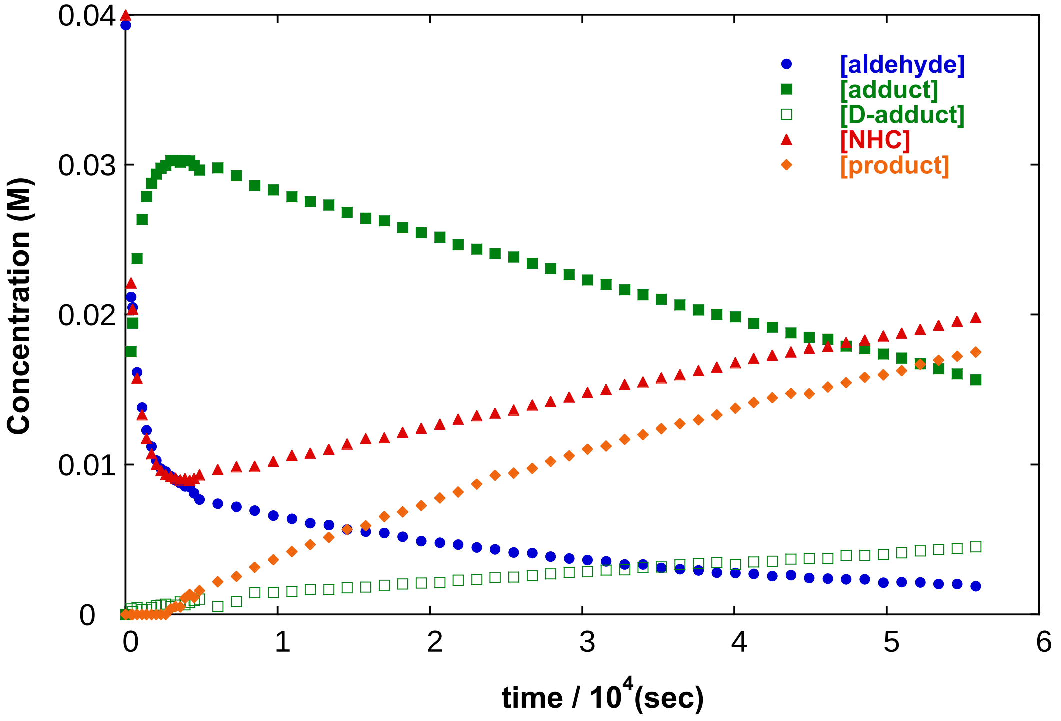

Figure S36. Reaction profile displaying concentration of species present against time for the intramolecular Stetter reaction using *N*-4-FC_6H_4 NHC precursor **34** in Et_3N:$Et_3N·HCl$ and CD_3OD at 15 °C.

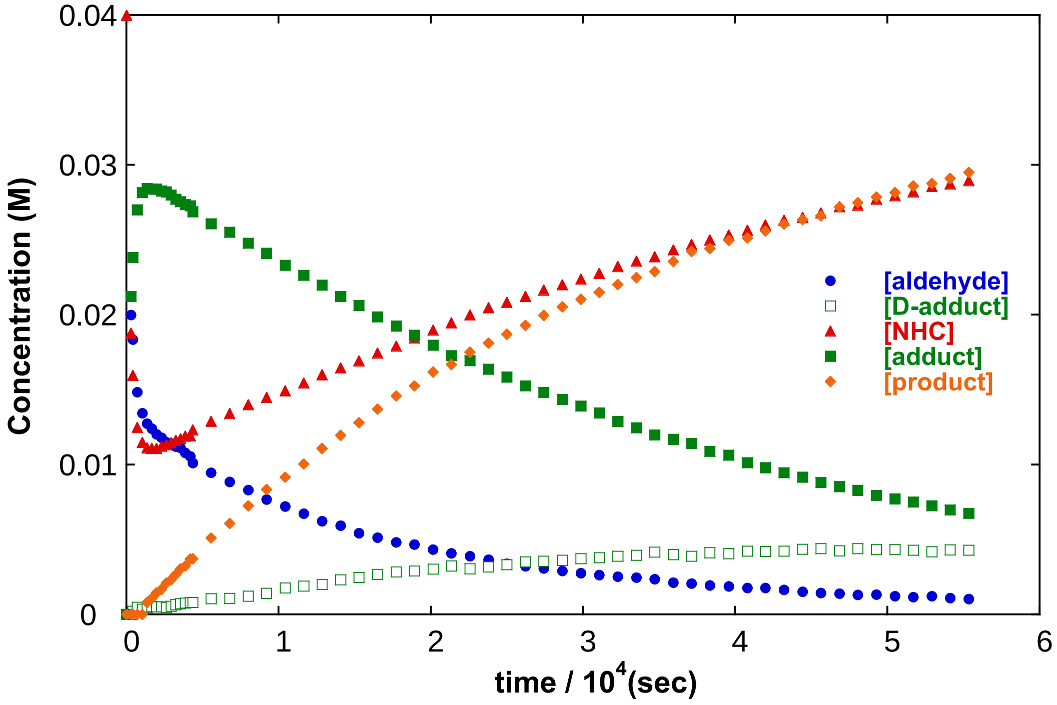

Figure S37. Reaction profile displaying concentration of species present against time for the intramolecular Stetter reaction using *N*-4-FC_6H_4 NHC precursor **34** in Et_3N:$Et_3N·HCl$ and CD_3OD at 25 °C.

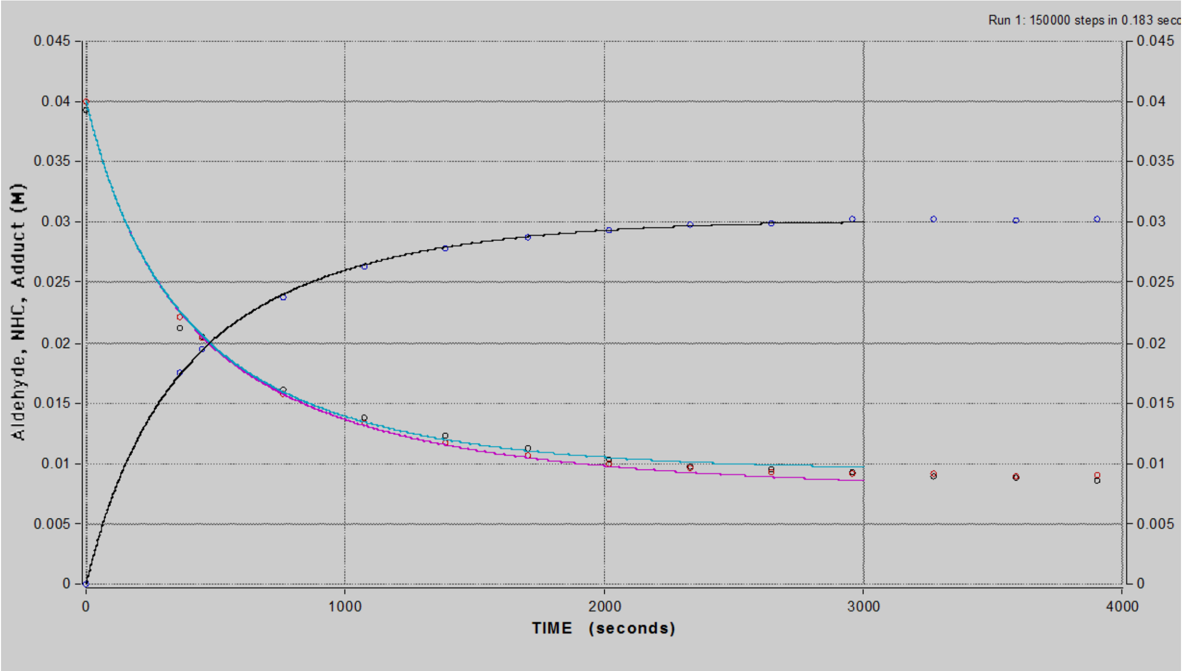

Figure S38. Plot showing formation of adduct during the Stetter reaction using *N*-4-FC_6H_4 NHC precursor **34** at 15 °C, up to the equilibrium concentrations. Open circles show the experimental data, with the solid line representing the fit to the kinetic model. Fitting data from t = 0 to t = 4000 s from Figure S36.

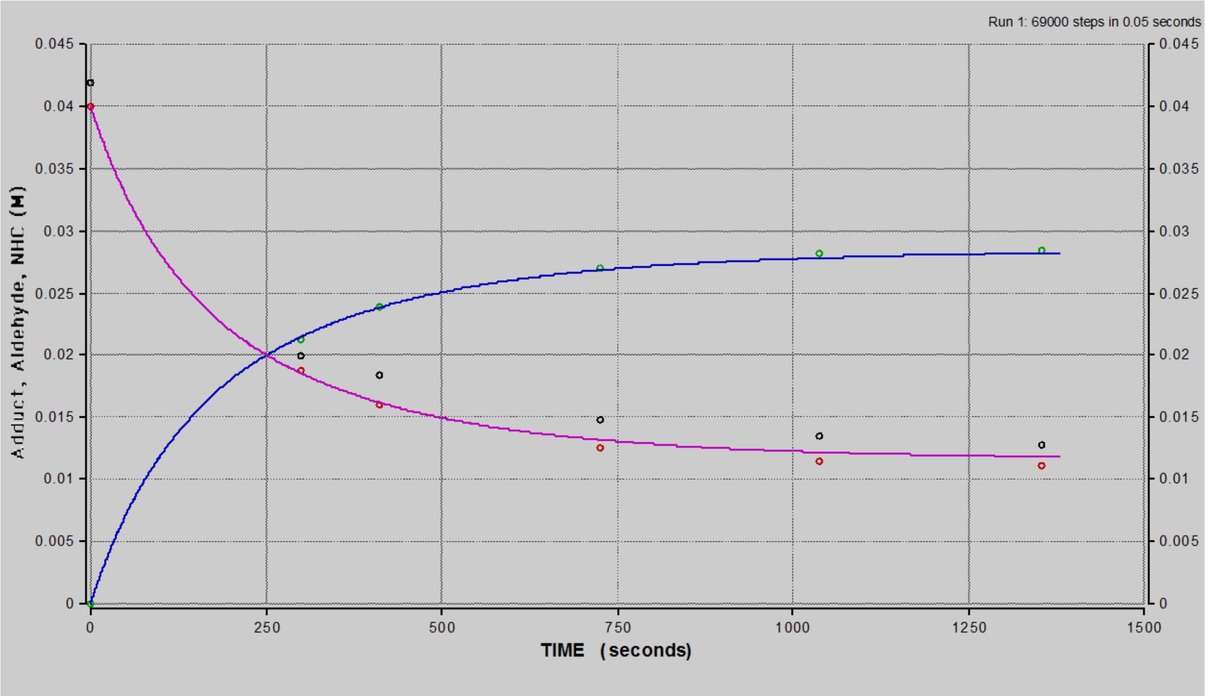

Figure S39. Plot showing formation of adduct during the Stetter reaction using *N*-4-FC_6H_4 NHC precursor **34** at 25 °C, up to the equilibrium concentrations. Open circles show the experimental data, with the solid line representing the fit to the kinetic model. Fitting data from t = 0 to t = 1500 s from Figure S37.

Table 2 Entry 3, Table S1 Entry 8

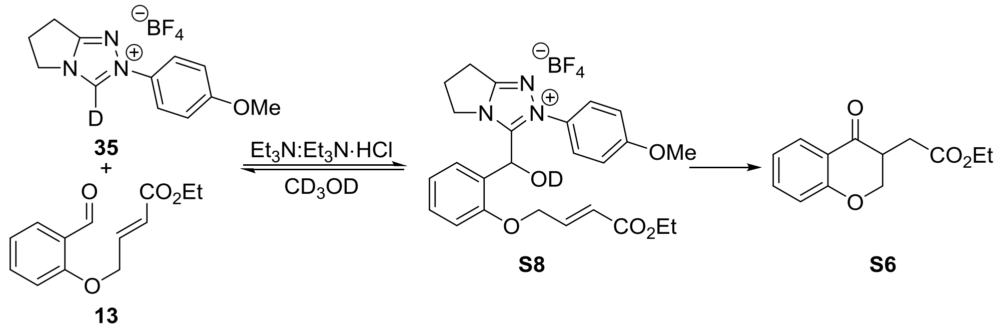

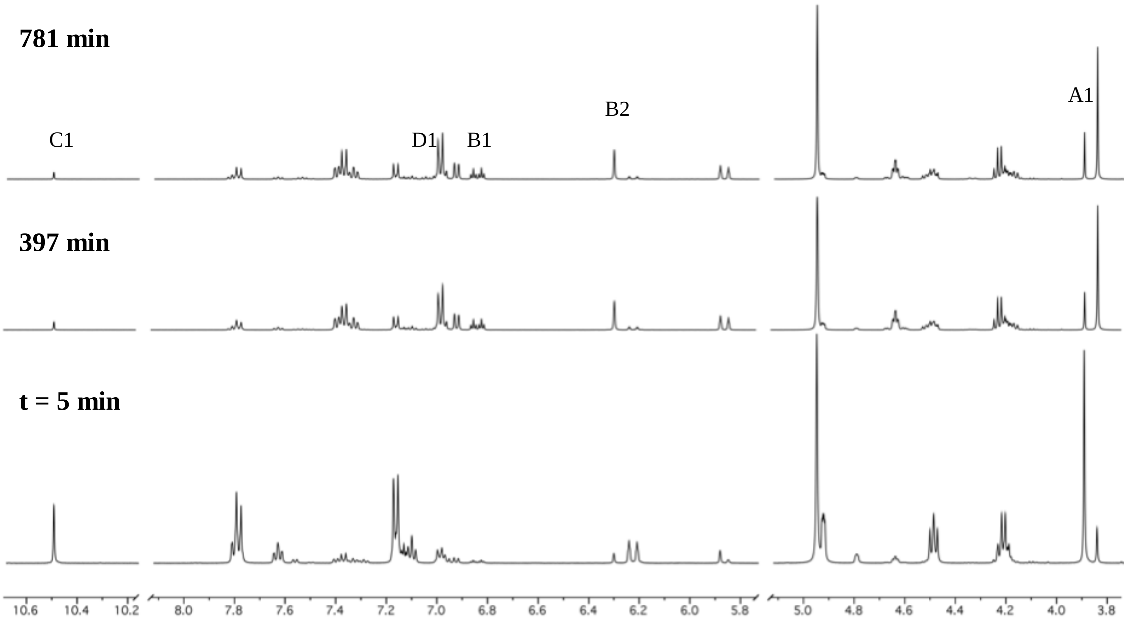

Figure S40. ^{1}H NMR spectra for the intramolecular Stetter reaction of aldehyde **13** with *N*-$MeOC_6H_4$ NHC precursor **35** in Et_3N:Et_3N·HCl and CD_3OD at 15 °C.

Concentrations of the species present were determined in a similar fashion to those described earlier. In this case, the singlet at 3.88 ppm (A1), relating to the 4-methoxy group of **35** was used to determine triazolium salt concentration. Otherwise concentrations were calculated as before, using Equations 12-16. The data was fitted using global fitting software (Berkeley Madonna version 8.3.18) according to a kinetic model described by Equation 11.

$$[\text{catalyst}] = \frac{(A_{A1}/3)}{(A_{B1} + (A_{A1}/3))} \times 0.04 \qquad \text{(Eq 12)}$$

$$[\text{aldehyde}] = \frac{1}{f_{ald}} \times \frac{(A_{C1})}{(A_{B1} + (A_{A1}/3))} \times 0.04 \qquad \text{(Eq 13)}$$

$$[\text{adduct}]_{tot} = \frac{(A_{B1})}{(A_{B1} + (A_{A1}/3))} \times 0.04 \qquad \text{(Eq 14)}$$

$$[\text{H-adduct}]_{tot} = \frac{(A_{B2})}{(A_{B1} + (A_{A1}/3))} \times 0.04 \qquad \text{(Eq 15)}$$

$$[\text{product}] = \frac{(A_{D1})}{(A_{B1} + (A_{A1}/3))} \times 0.04 \qquad \text{(Eq 16)}$$

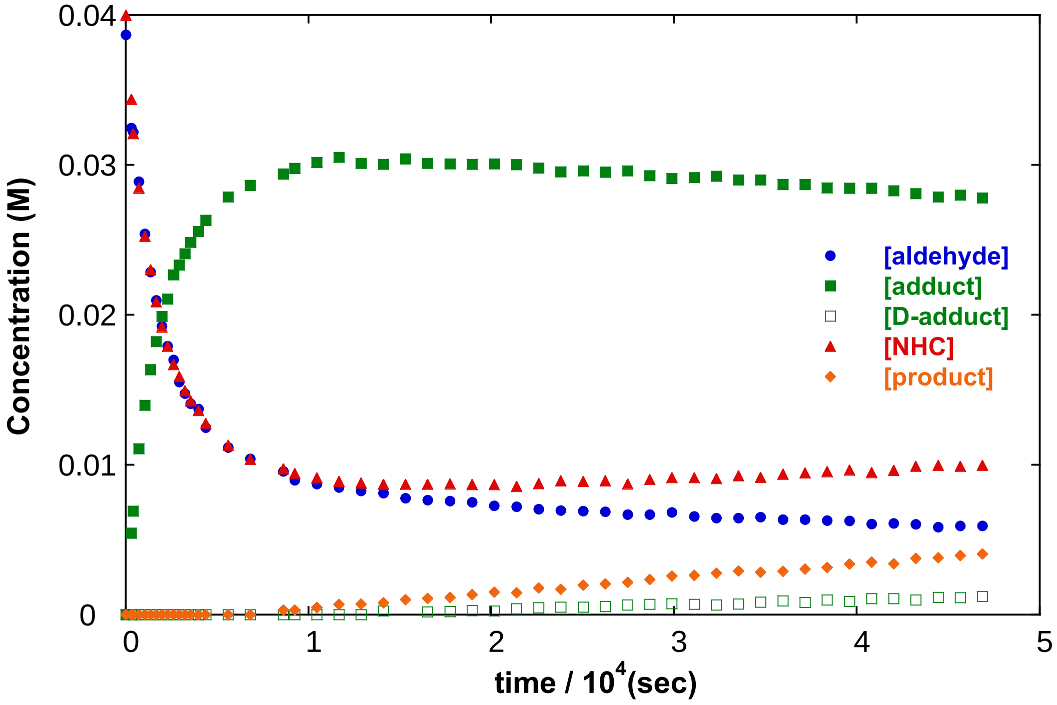

Figure S41. Reaction profile displaying concentration of species present against time for the intramolecular Stetter reaction using *N*-$MeOC_6H_4$ NHC precursor **35** in Et_3N:$Et_3N{\cdot}HCl$ and CD_3OD at 15 °C.

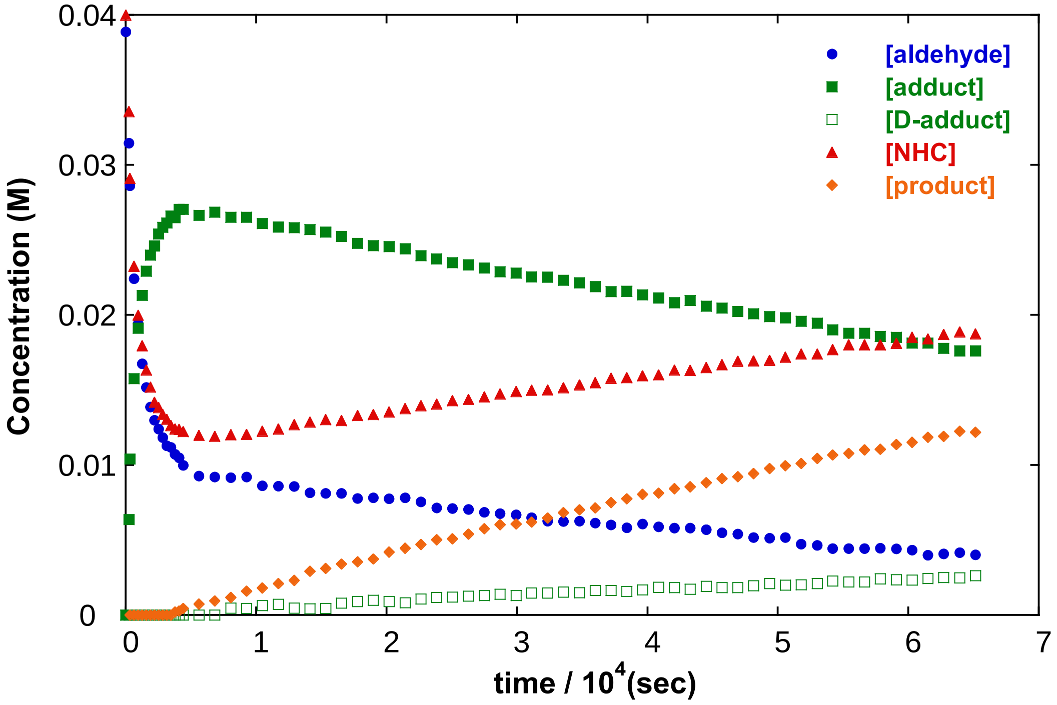

Figure S42. Reaction profile displaying concentration of species present against time for the intramolecular Stetter reaction using *N*-$MeOC_6H_4$ NHC precursor **35** in Et_3N:$Et_3N{\cdot}HCl$ and CD_3OD at 25 °C.

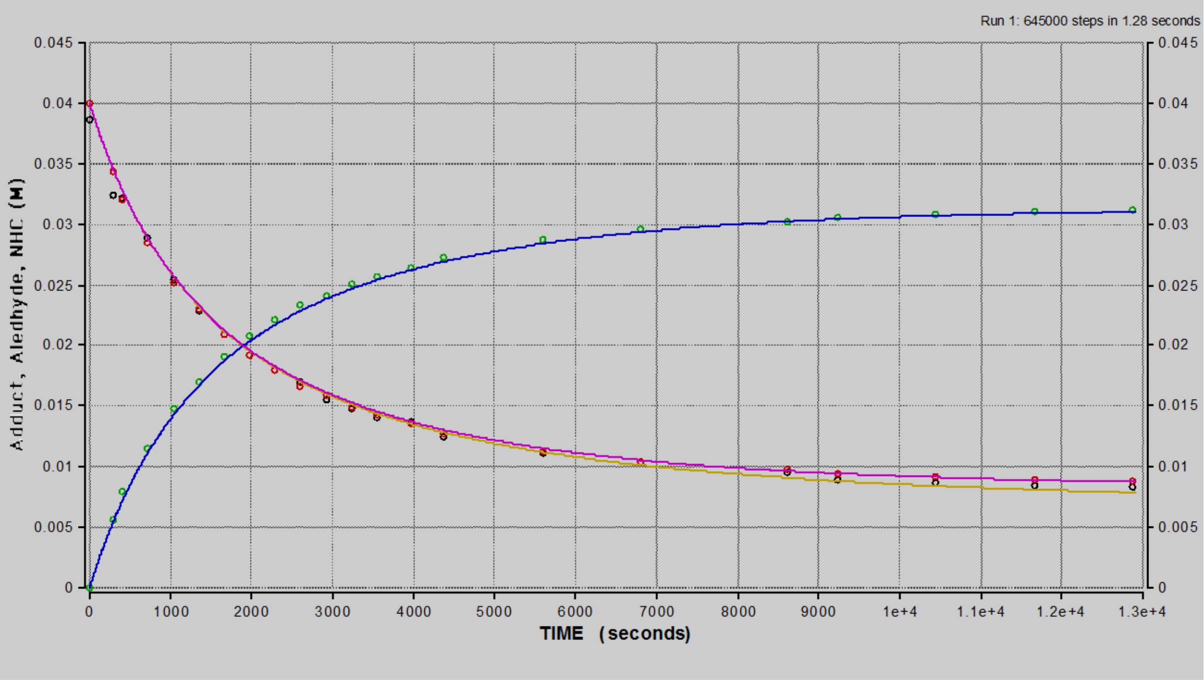

Figure S43. Plot showing formation of adduct during the Stetter reaction using *N*-MeOC_6H_4 NHC precursor **35** at 15 °C, up to the equilibrium concentrations. Open circles show the experimental data, with the solid line representing the fit to the kinetic model. Fitting data from t = 0 to t = 13000 s from Figure S41.

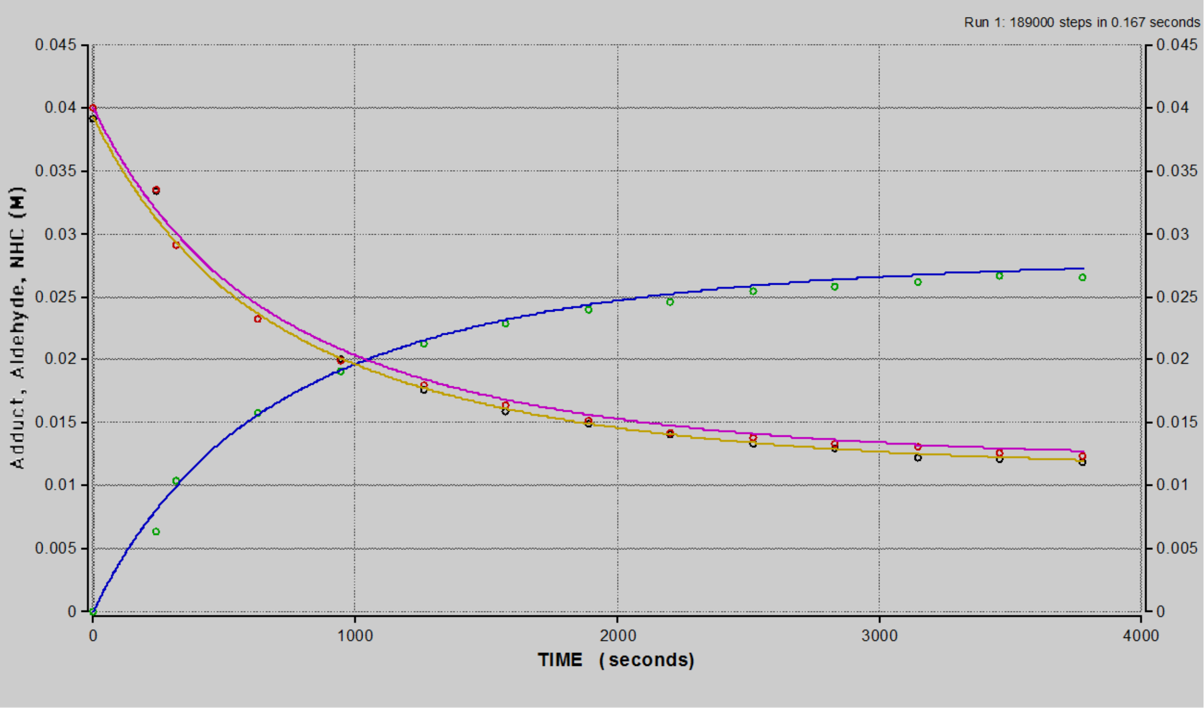

Figure S44. Plot showing formation of adduct during the Stetter reaction using *N*-MeOC_6H_4 NHC precursor **35** at 25 °C, up to the equilibrium concentrations. Open circles show the experimental data, with the solid line representing the fit to the kinetic model. Fitting data from t = 0 to t = 4000 s from Figure S42.

Table 2 Entry 4, Table S1 Entry 9

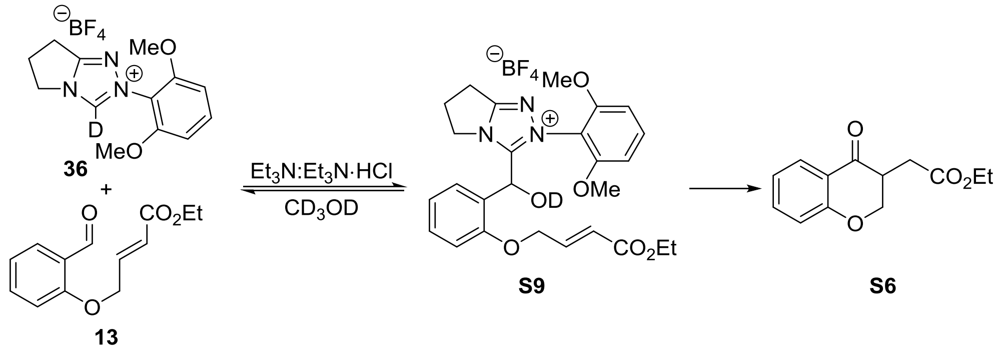

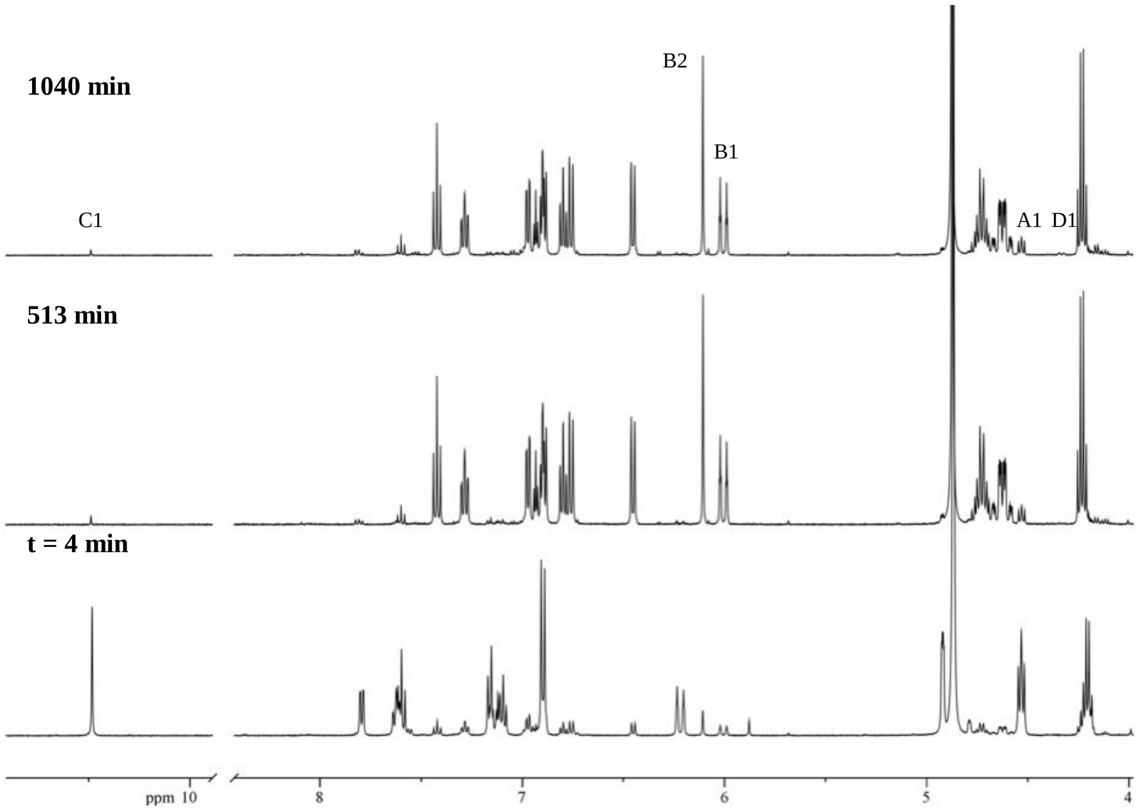

Figure S45. ^{1}H NMR spectra for the intramolecular Stetter reaction of aldehyde **13** with *N*-2,6-$(MeO)_2C_6H_3$ NHC precursor **36** in Et_3N:Et_3N·HCl and CD_3OD at 15 °C.

The concentration of NHC precursor was determined using the triplet at 4.5 ppm, corresponding to the backbone CH_2 of **36**. Otherwise, concentrations were determined as previously described using the Equations 3-10 and the data was fitted using global fitting software (Berkeley Madonna version 8.3.18) according to a kinetic model described by Equation 11.

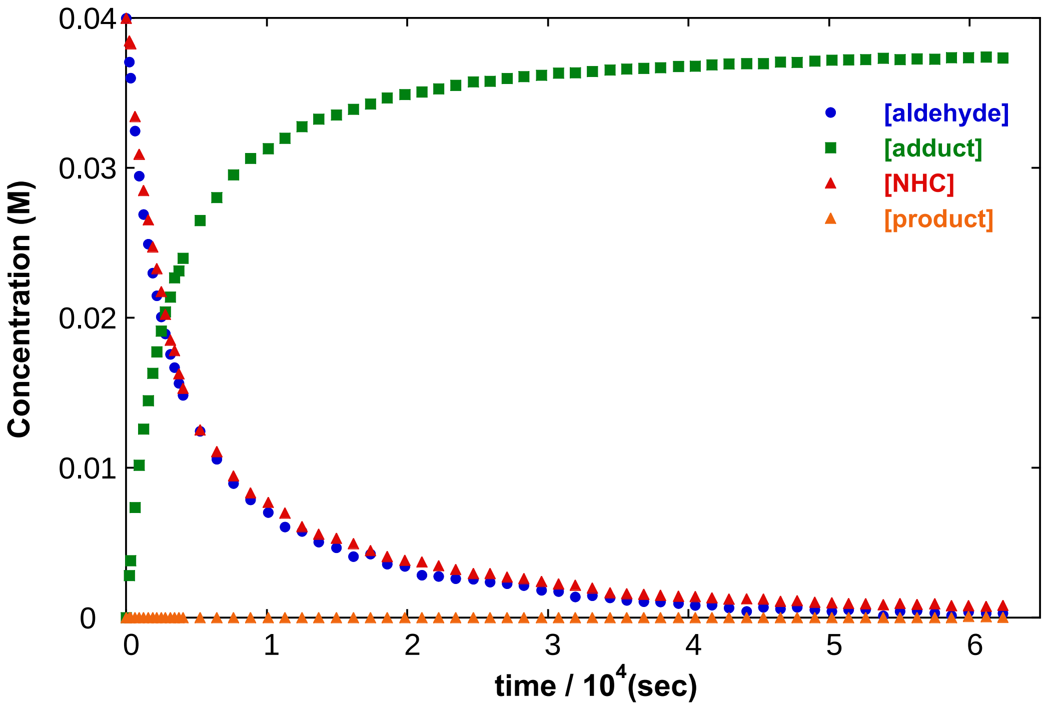

Figure S46. Reaction profile displaying concentration of species present against time for the intramolecular Stetter reaction using *N*-2,6-$(MeO)_2C_6H_3$ NHC precursor **36** in Et_3N:Et_3N·HCl and CD_3OD at 15 °C.

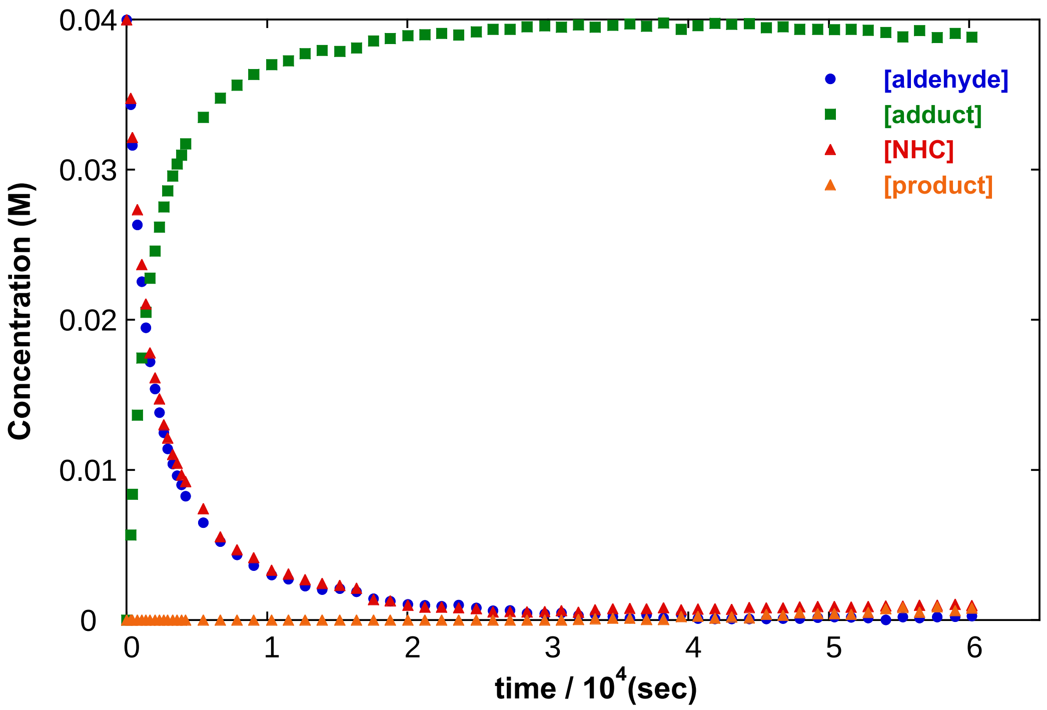

Figure S47. Reaction profile displaying concentration of species present against time for the intramolecular Stetter reaction using *N*-2,6-$(MeO)_2C_6H_3$ NHC precursor **36** in Et_3N:Et_3N·HCl and CD_3OD at 25 °C.

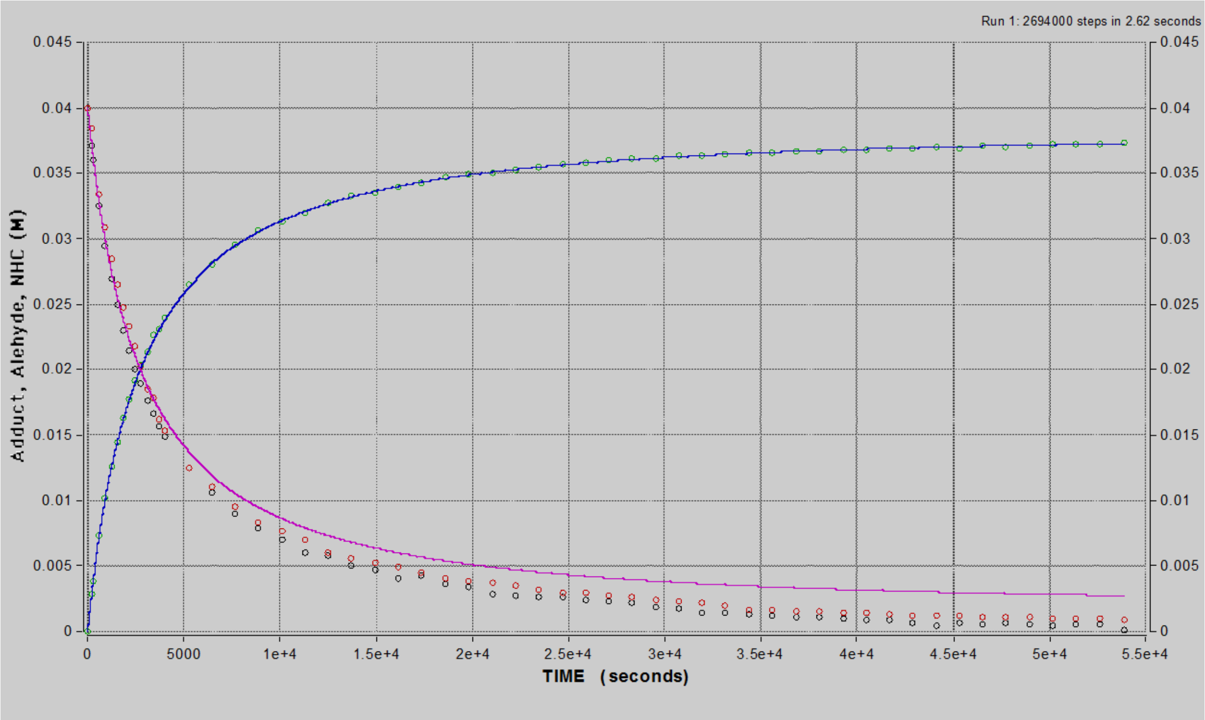

Figure S48. Plot showing formation of adduct during the Stetter reaction using *N*-2,6-$(MeO)_2C_6H_3$ NHC precursor **36** at 15 °C, up to the equilibrium concentrations. Open circles show the experimental data, with the solid line representing the fit to the kinetic model. Fitting data from t = 0 to t = 55000 s from Figure S46.

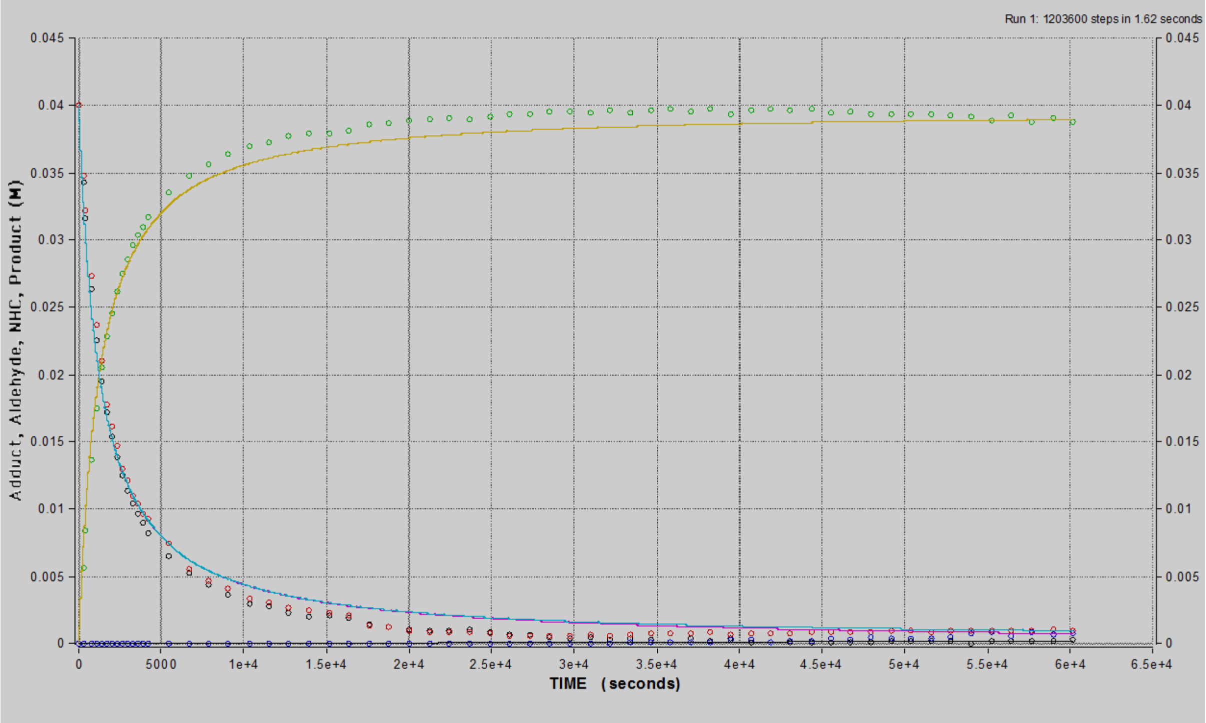

Figure S49. Plot showing formation of adduct during the Stetter reaction using *N*-2,6-$(MeO)_2C_6H_3$ NHC precursor **36** at 25 °C, up to the equilibrium concentrations. Open circles show the experimental data, with the solid line representing the fit to the kinetic model. Fitting data from t = 0 to t = 60000 s from Figure S47.

Table 2 Entry 5, Table S1 Entry 10

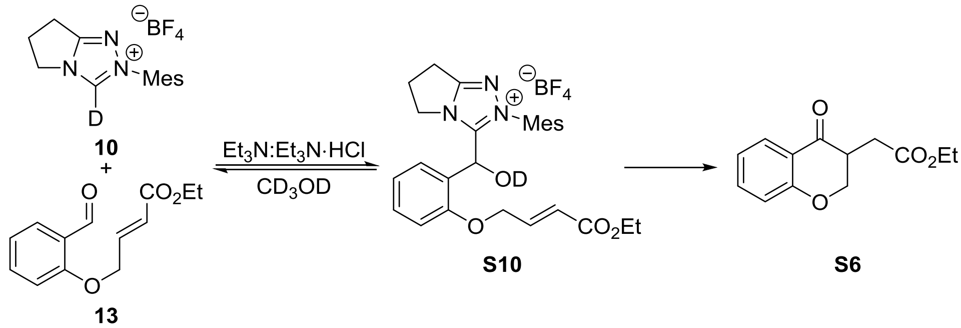

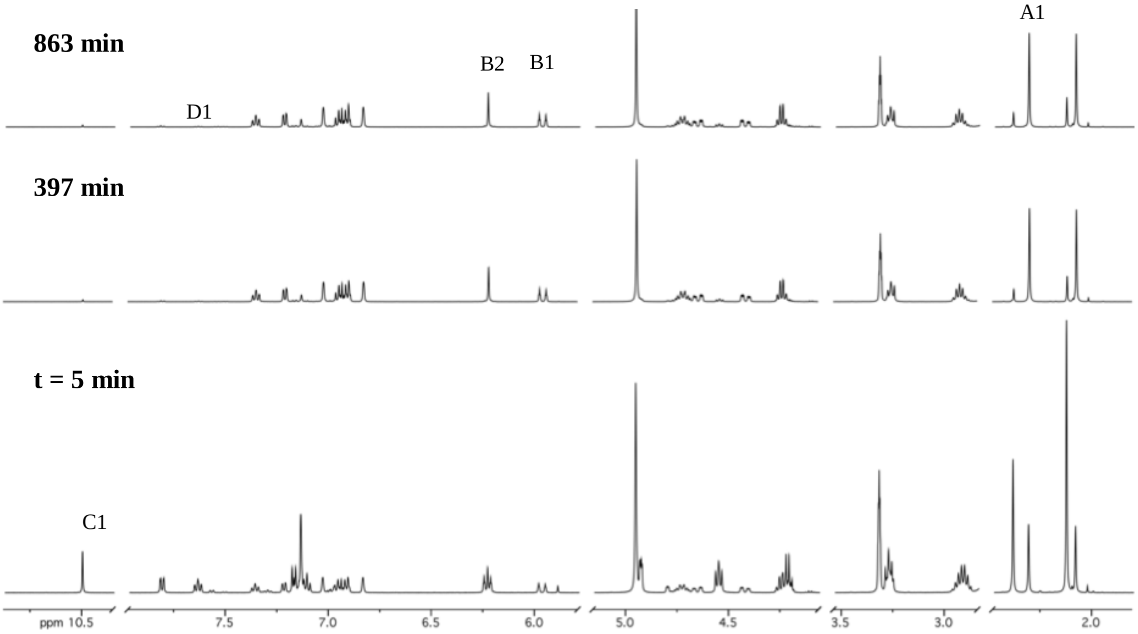

Figure S50. ^{1}H NMR spectra for the intramolecular Stetter reaction of aldehyde **13** with *N*-Mes NHC precursor **10** in Et_3N:Et_3N·HCl and CD_3OD at 15 °C.

For this system, the singlet at 2.38 ppm (A1), relating to the methyl group at the 4-position of the *N*-Mes substituent of **10** was used to determine triazolium salt concentration with concentrations calculated as before using Equations 12-16. Furthermore, there was overlap of the CH singlet of 3-(hydroxybenzyl)azolium salt **S10** at 6.22 ppm with the doublet of triplets for the CH α to the ester group of aldehyde **13** (B2). To correct for the overlap in the calculation of [H-adduct], a subtraction was made for the amount of aldehyde present (Equation 17). As before, the integrals were converted into concentrations relative to the total amount of triazolium-containing species. The data was fitted using global fitting software (Berkeley Madonna version 8.3.18) according to a kinetic model described by Equation 11.

$$[\text{H-adduct}] = \frac{(A_{B2} - (A_{C1} \times (1/f_{ald})))}{(A_{B1} + (A_{A1}/3))} \times 0.04 \qquad \text{(Eq 17)}$$

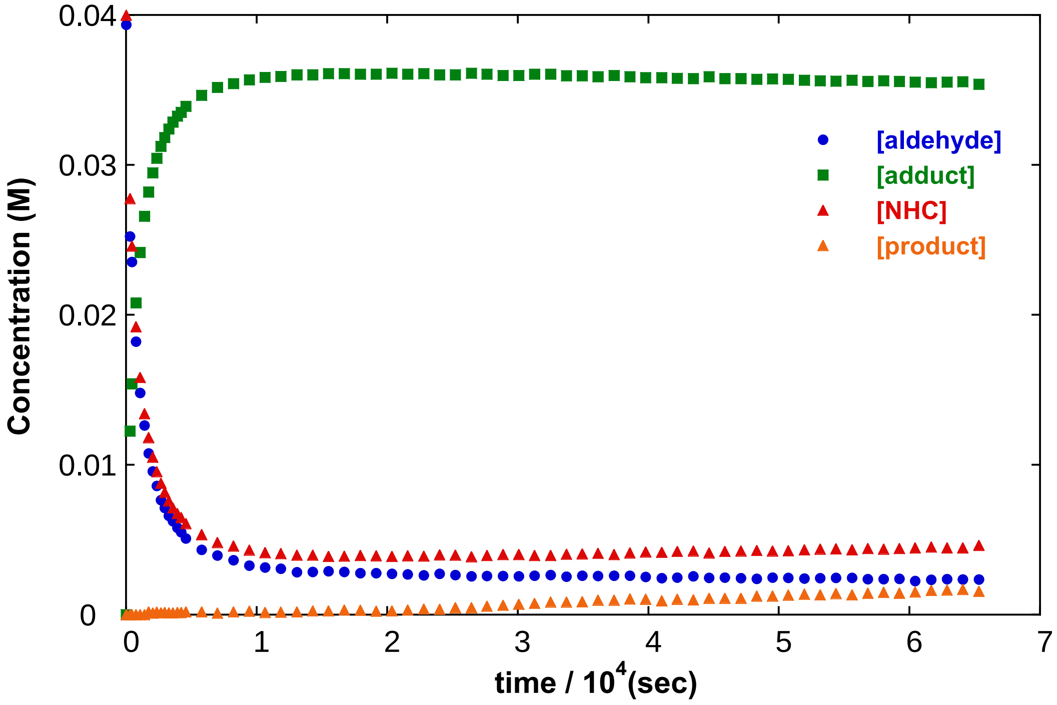

Figure S51. Reaction profile displaying concentration of species present against time for the intramolecular Stetter reaction using *N*-Mes NHC precursor **10** in Et_3N:Et_3N·HCl and CD_3OD at 15 °C.

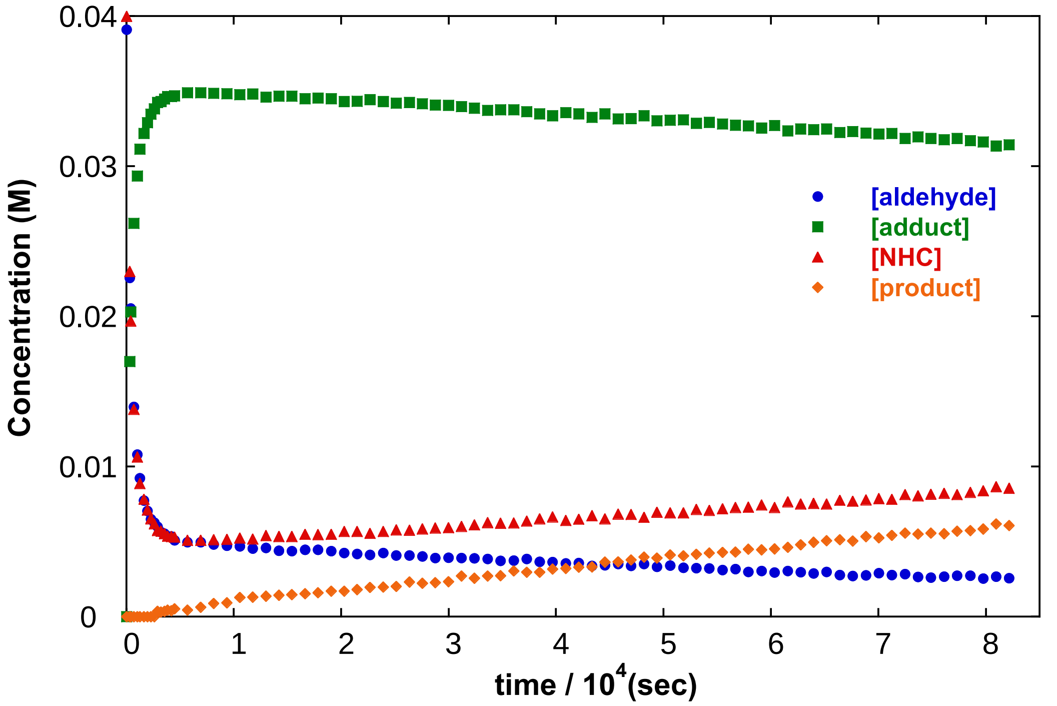

Figure S52. Reaction profile displaying concentration of species present against time for the intramolecular Stetter reaction using *N*-Mes NHC precursor **10** in Et_3N:Et_3N·HCl and CD_3OD at 25 °C.

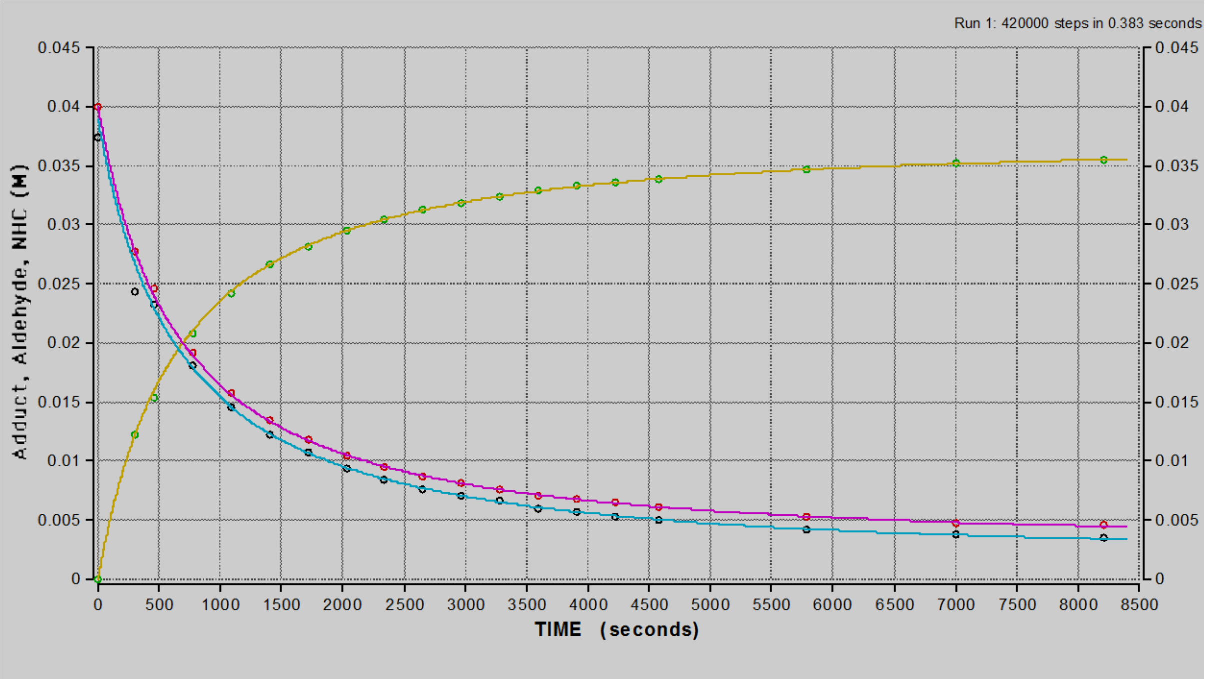

Figure S53. Plot showing formation of adduct during the Stetter reaction using *N*-Mes NHC precursor **10** at 15 °C, up to the equilibrium concentrations. Open circles show the experimental data, with the solid line representing the fit to the kinetic model. Fitting data from t = 0 to t = 8500 s from Figure S51.

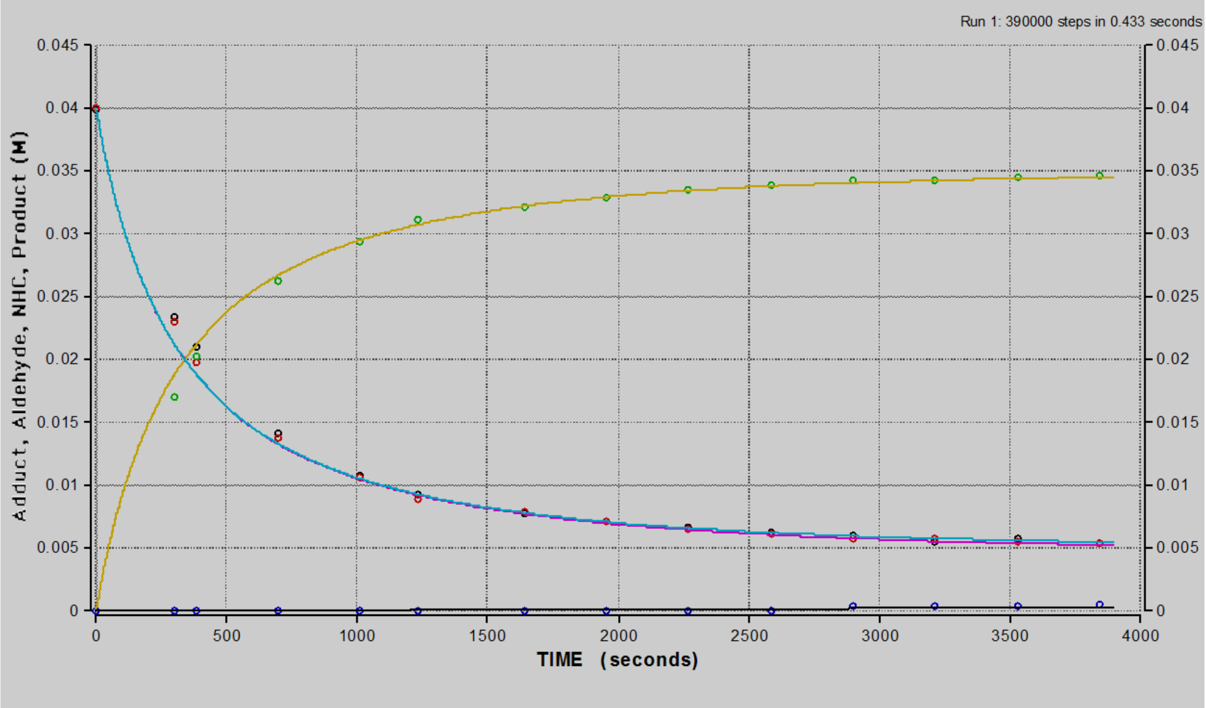

Figure S54. Plot showing formation of adduct during the Stetter reaction using *N*-Mes NHC precursor **10** at 25 °C, up to the equilibrium concentrations. Open circles show the experimental data, with the solid line representing the fit to the kinetic model. Fitting data from t = 0 to t = 4000 s from Figure S52.

Determination of k_1, k_{-1} and K

The 3-(hydroxybenzyl)azolium equilibrium in the forward direction is shown in Scheme S2. Second-order rate constants for the formation of the 3-(hydroxybenzyl)azolium (k_1, M^{-1} s^{-1}) can be calculated from the consumption of aldehyde or NHC precursor up to the point where equilibrium is reached, before significant product formation. The concentration of aldehyde can be expressed in Equation 18, which assuming [NHC] = [aldehyde], may be written as Equation 19.

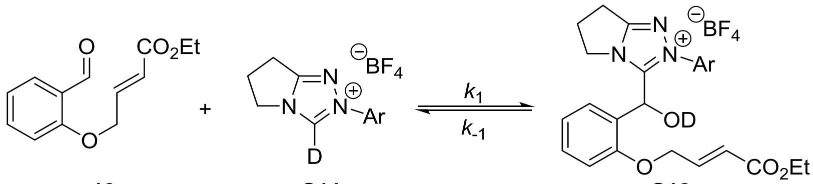

Scheme S2. Formation of 3-(hydroxybenzyl)azolium salt.

$$\frac{d[\text{aldehyde}]}{dt} = -k_1[\text{NHC}][\text{aldehyde}] + k_{-1}[\text{H-adduct}] \quad \text{(Eq 18)}$$

$$\frac{d[\text{aldehyde}]}{dt} = -k_1[\text{aldehyde}]^2 + k_{-1}\frac{[\text{aldehyde}]_e^2}{([\text{aldehyde}]_0 - [\text{aldehyde}]_e)} - ([\text{aldehyde}]_0 - [\text{aldehyde}]) \quad \text{(Eq 19)}$$

With initial concentrations of [adduct] = 0 and $[\text{NHC}]_0 = [\text{aldehyde}]_0 = b$, integration of Equation 19 gives Equation 20, where $y = ([\text{aldehyde}]_0 - [\text{aldehyde}]) = ([\text{NHC}]_0 - [\text{NHC}])$ and $y_e = ([\text{aldehyde}]_0 - [\text{aldehyde}]_e) = ([\text{NHC}]_0 - [\text{NHC}]_e)$.[5] Therefore the slope of a semilogarithmic plot of $(y_e(b^2 - yy_e))/(b^2(y_e - y))$ against time divided by $(y_e)/(b^2-y_e^2)$ can be used to estimate values for k_1, in the build up to equilibrium.

$$\frac{y_e}{(b^2 - y_e^2)}\ln\frac{y(b^2 - yy_e)}{b^2(y_e - y)} = k_1 t \quad \text{(Eq 20)}$$

Semilogarithmic plots of $(y_e(\text{b}^2 - yy_e)/(b^2(y_e - y))$, followed over the first three half-lives, are shown in Figures 55-56.

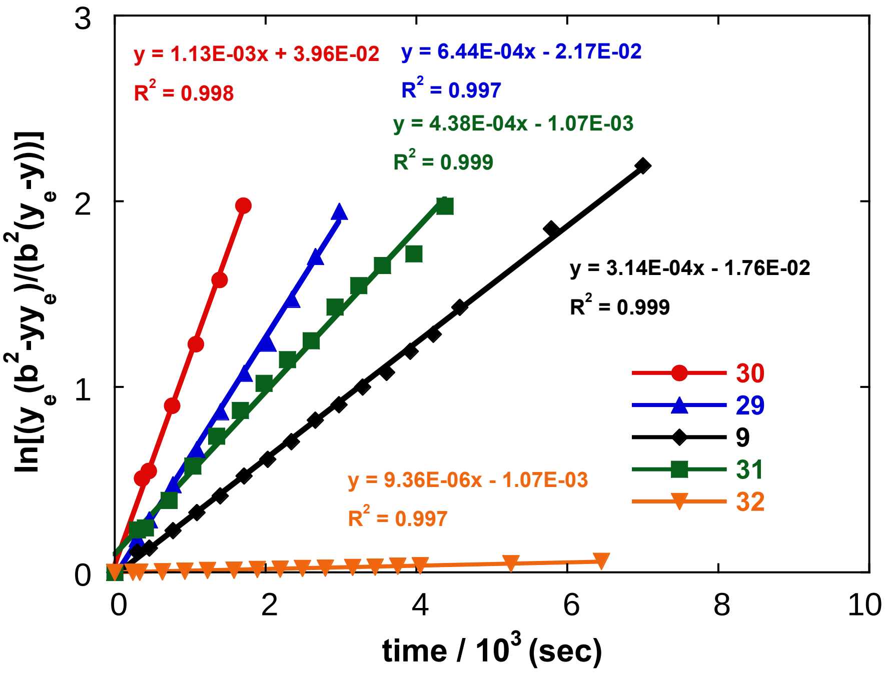

Figure S55. Semilogarithmic plots of $(y_e(b^2 - yy_e)/(b^2(y_e - y))$ against time, obtained from the reaction of aldehyde **13** (0.04 M) with a range of triazolium salts (0.04 M) in Et_3N:$Et_3N{\cdot}HCl$ and CD_3OD at 15 °C.

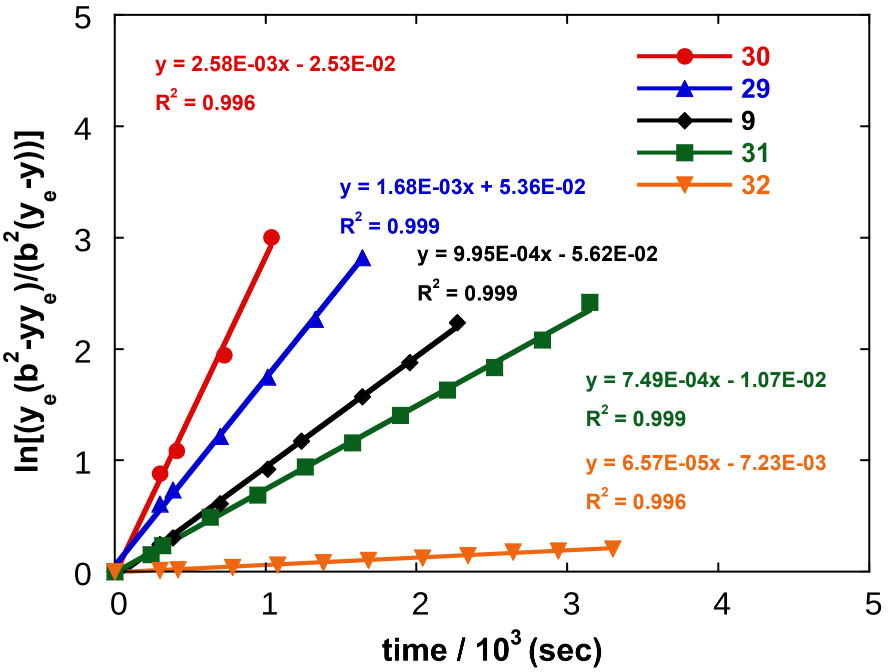

Figure S56. Semilogarithmic plots of $(y_e(b^2 - yy_e)/(b^2(y_e - y))$ against time, obtained from the reaction of aldehyde **13** (0.04 M) with a range of triazolium salts (0.04 M) in Et_3N:$Et_3N{\cdot}HCl$ and CD_3OD at 25 °C.

According to Equation 21, values for the equilibrium constant K^{exp} (M^{-1}) could be estimated from concentrations of species present once maximum 3-(hydroxybenzyl)azolium concentration was achieved. From the data collected at 15 ºC and 25 ºC, K^{exp} and k_1 values were used to estimate k_{-1} (s^{-1}) values.

$$K^{\exp} = \frac{k_1}{k_{-1}} = \frac{[\text{H-adduct}]_{eq}}{[\text{NHC}]_{eq}[\text{aldehyde}]_{eq}} \qquad \text{(Eq 21)}$$

Determination of Rate and Equilibrium Constants for 3-(Hydroxybenzyl)azolium Adduct Dissociation in CD_3OD (Table 3, Table S2)

General Procedure

In an NMR tube, 3-(hydroxybenzyl)azolium salt (30 mmol) and $Et_3N{\cdot}HCl$ (0.405 M) was dissolved in CD_3OD (650 μL) containing 0.03% v/v TMS. The reaction was initiated by the addition of 100 μL of a solution of Et_3N (0.795 M) in CD_3OD. This gave an overall adduct concentration of 0.04 M and a total buffer concentration of 0.16 M. The reaction was monitored by ^1H NMR spectroscopy on a Bruker Avance 500 MHz NMR spectrometer with the probe set at 25 °C. Spectra were taken at 5 min intervals for the first hour, followed by 20 min intervals over the next ~16 hours. The concentration of each species present was determined as previously described.

Table 3, Entry 1

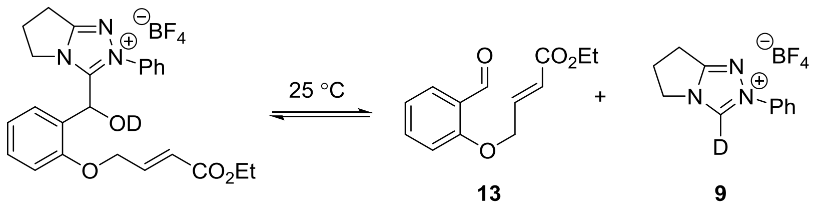

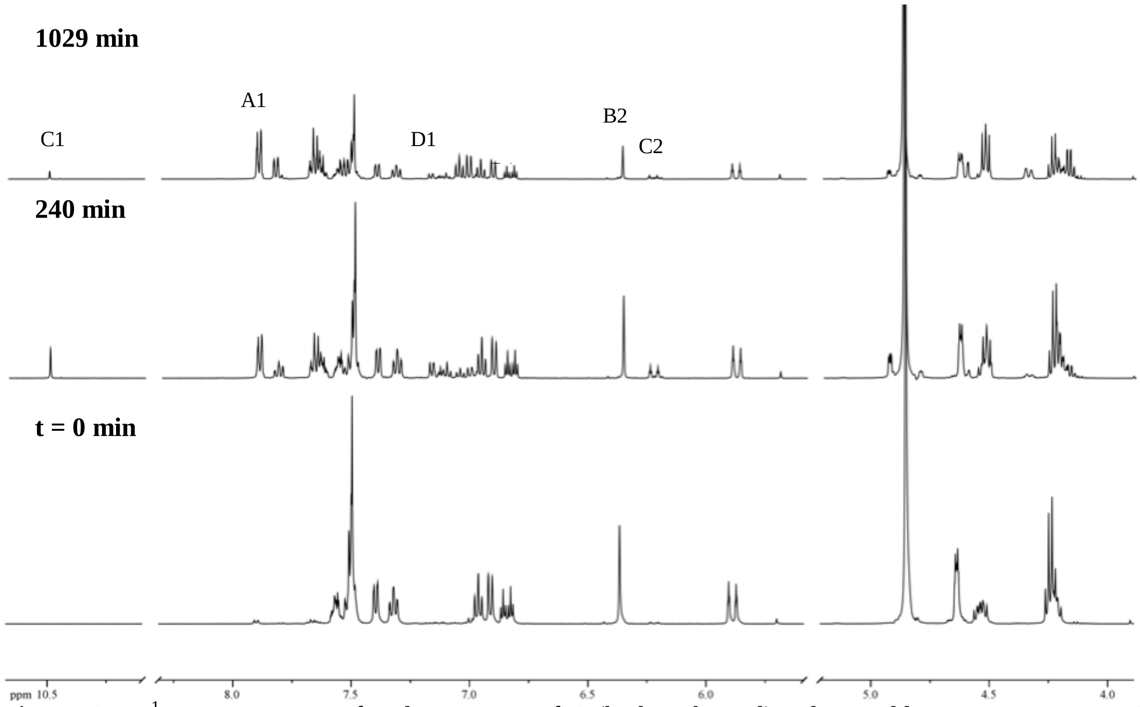

Figure S57. ^1H NMR spectra for dissociation of 3-(hydroxybenzyl)azolium adduct in Et_3N:$Et_3N{\cdot}HCl$ and CD_3OD at 25 °C.

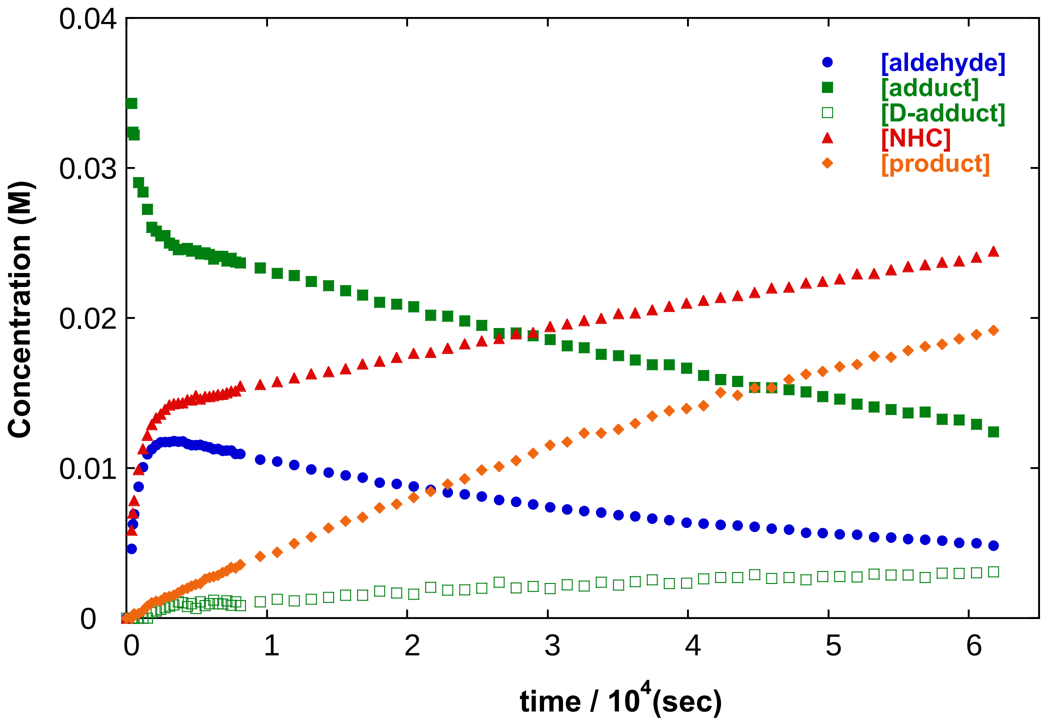

Figure S58. Reaction profile for dissociation of 3-(hydroxybenzyl)azolium adduct in Et_3N:$Et_3N·HCl$ and CD_3OD at 25 °C.

Table 3, Entry 2

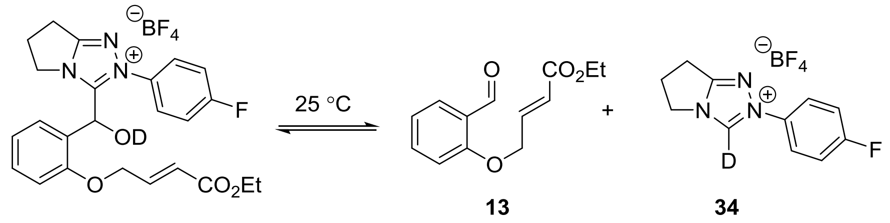

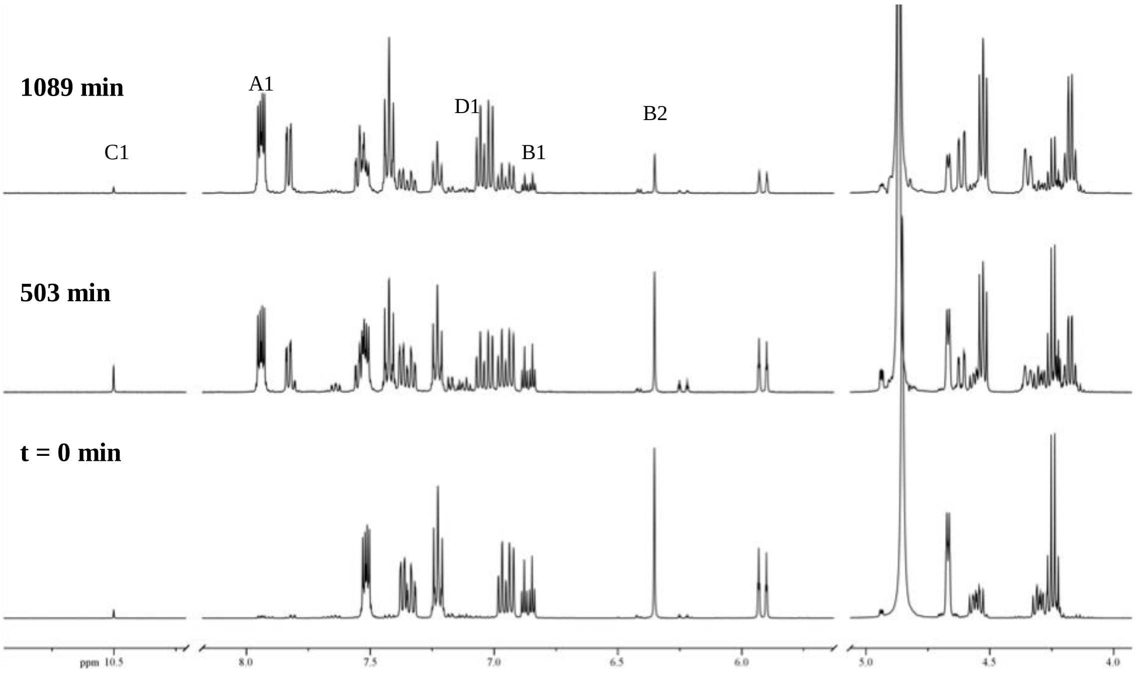

Figure S59. ^{1}H NMR spectra for dissociation of 3-(hydroxybenzyl)azolium adduct in Et_3N:$Et_3N·HCl$ and CD_3OD at 25 °C.

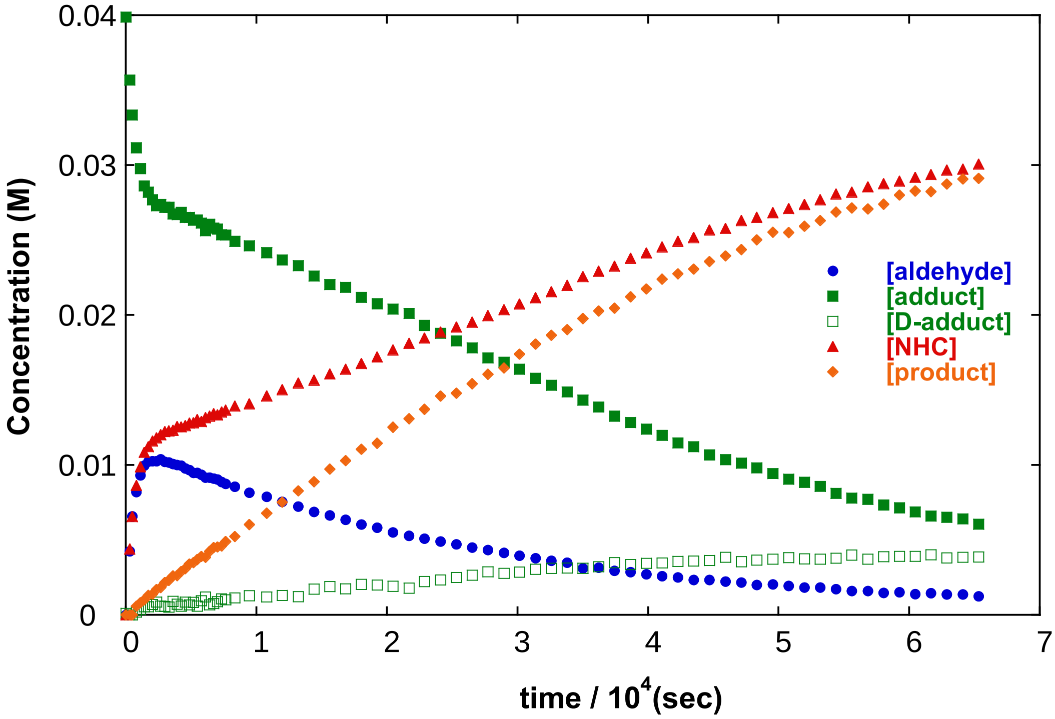

Figure S60. Reaction profile for dissociation of 3-(hydroxybenzyl)azolium adduct in Et_3N:$Et_3N·HCl$ and CD_3OD at 25 °C.

Table 3, Entry 3

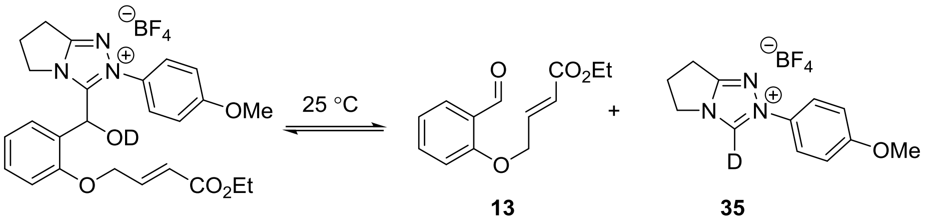

13 **35**

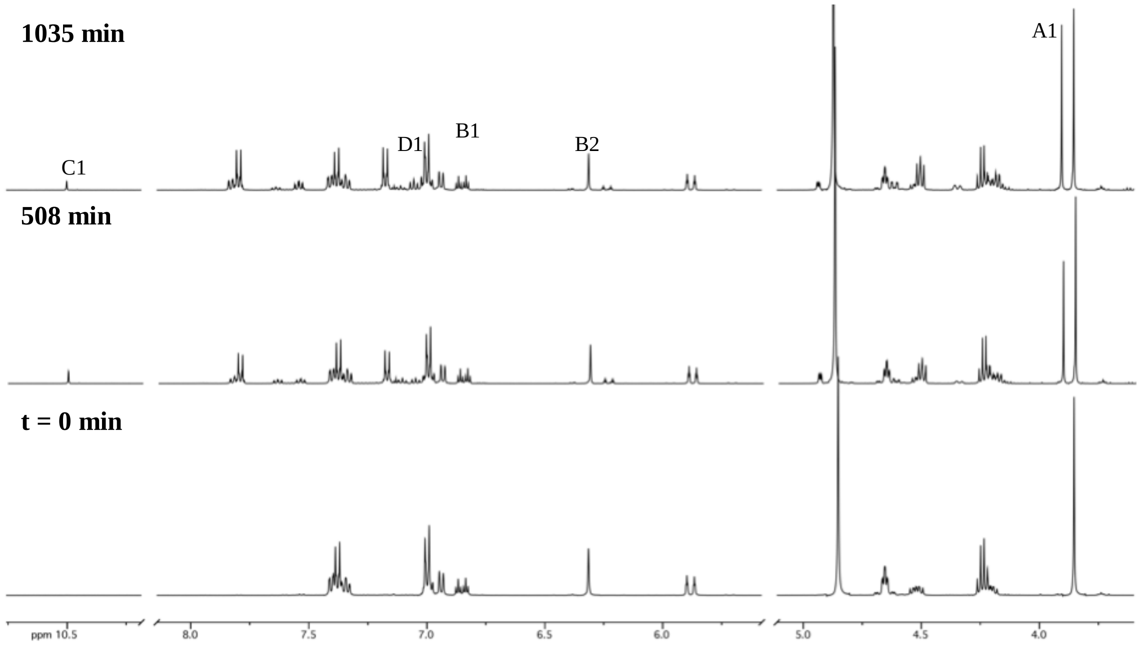

Figure S61. ^{1}H NMR spectra for dissociation of 3-(hydroxybenzyl)azolium adduct in Et_3N:$Et_3N{\cdot}HCl$ and CD_3OD at 25 °C.

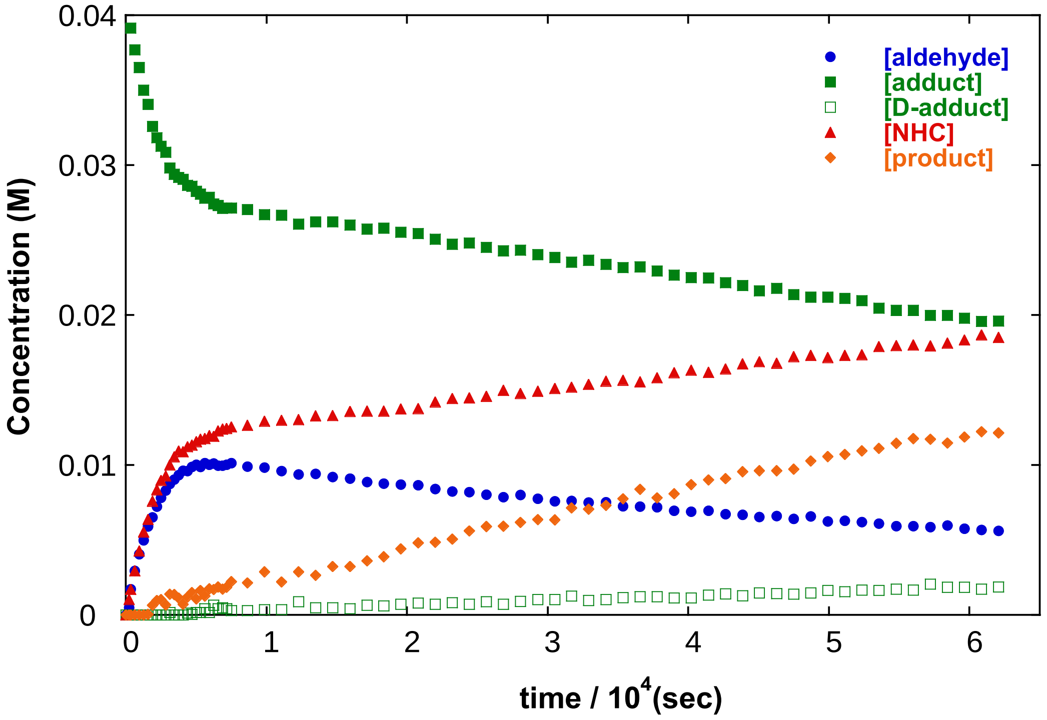

Figure S62. Reaction profile for dissociation of 3-(hydroxybenzyl)azolium adduct in Et_3N:$Et_3N{\cdot}HCl$ and CD_3OD at 25 °C.

Table 3, Entry 5

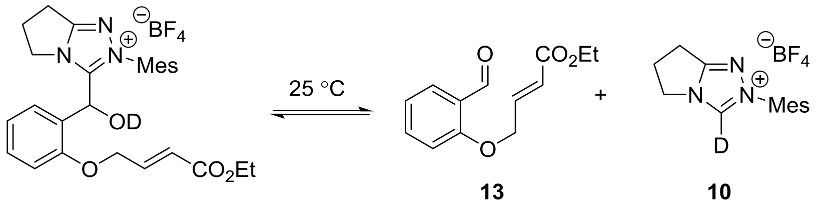

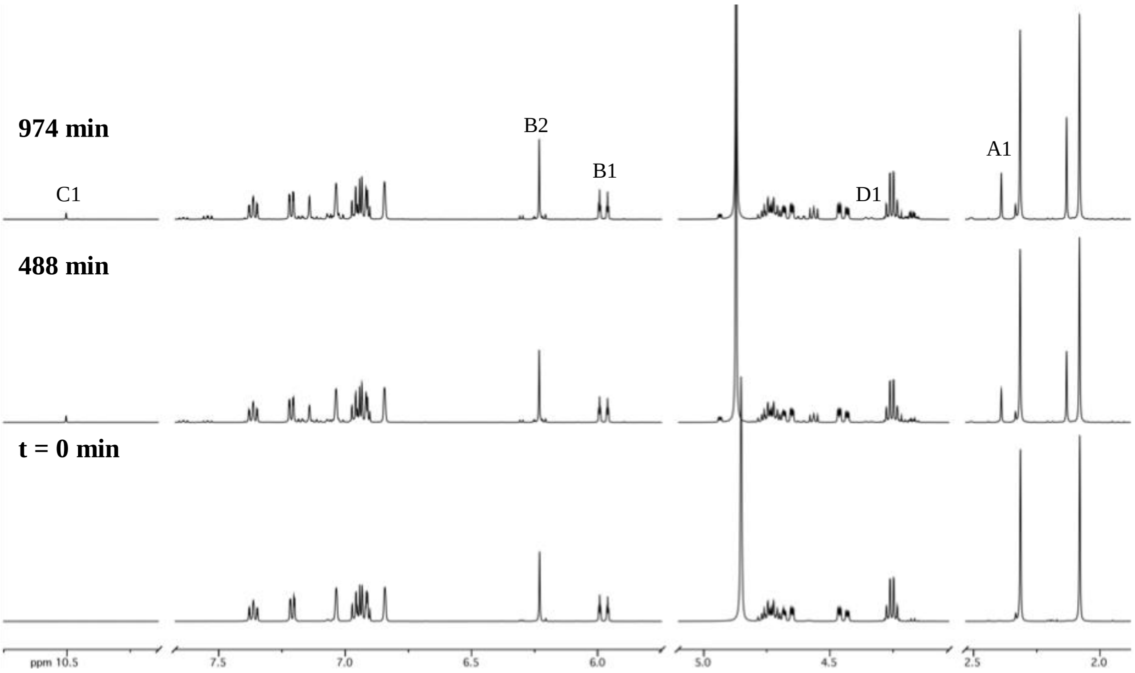

Figure S63. ^{1}H NMR spectra for dissociation of 3-(hydroxybenzyl)azolium adduct in Et_3N:Et_3N·HCl and CD_3OD at 25 °C.

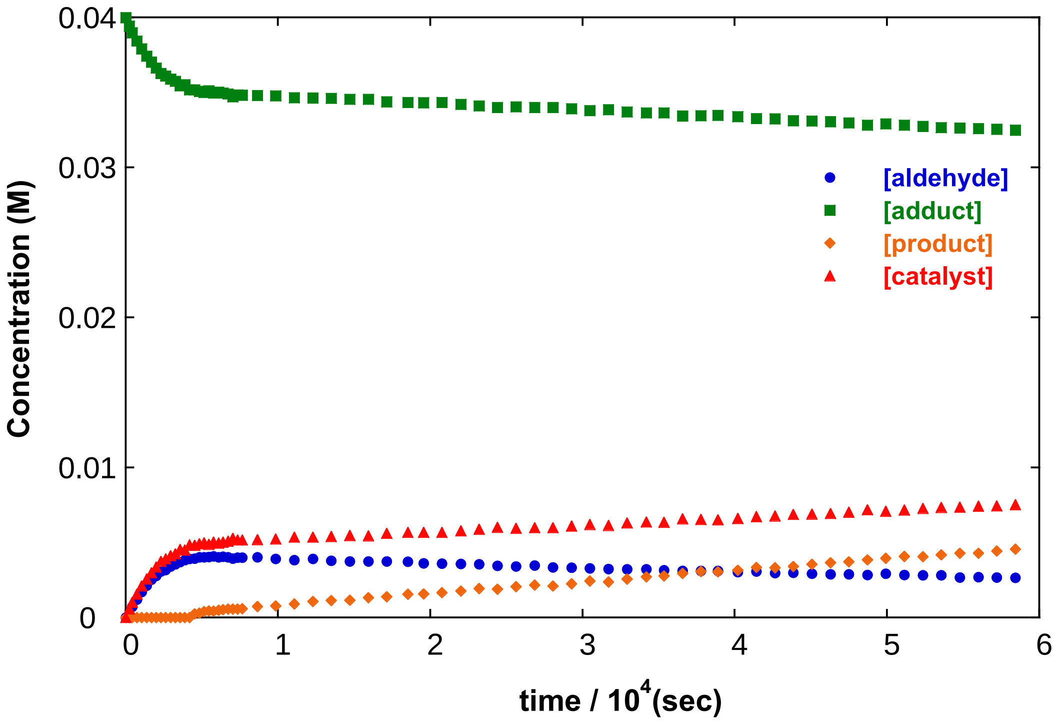

Figure S64. Reaction profile for dissociation of 3-(hydroxybenzyl)azolium adduct in Et_3N:Et_3N·HCl and CD_3OD at 25 °C.

Determination of Rate Constants: Decay of 3-(Hydroxybenzyl)azolium Salt to Equilibrium

From experiments using an initial adduct concentration of 0.04 M, monitoring the initial decrease of adduct at 25 °C, it was possible to determine the decay towards equilibrium before significant (>2%) product formation. The equilibrium constant (K^{diss}, M) for this process can be described by Equation 22, assuming that $[\text{aldehyde}]_{eq} = [\text{NHC}]_{eq}$.

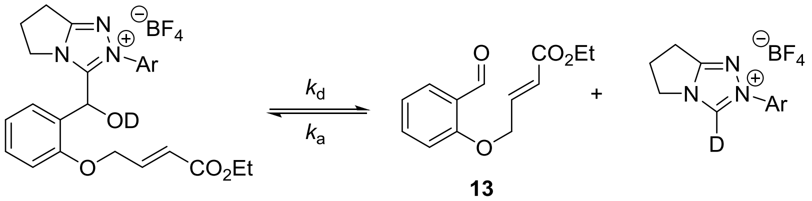

13

Scheme S3. Base catalysed 3-(hydroxybenzyl)azolium salt equilibrium.

$$K^{\mathrm{diss}} = \frac{k_d}{k_a} = \frac{[\text{NHC}]_{eq}[\text{aldehyde}]_{eq}}{[\text{H-adduct}]_{eq}} = \frac{([\text{aldehyde}]_{eq})^2}{[\text{H-adduct}]_{eq}} \qquad \text{(Eq 22)}$$

The concentration of adduct can be expressed in Equation 23, which assuming [NHC] = [aldehyde], may be written as Equation 24.

$$\frac{d[\text{H-adduct}]}{dt} = -k_d[\text{H-adduct}] + k_a[NHC][\text{aldehyde}] \qquad \text{(Eq 23)}$$

$$\frac{d[\text{H-adduct}]}{dt} = -k_d[\text{H-adduct}] + k_d \frac{[\text{H-adduct}]_e}{([\text{H-adduct}]_0 - [\text{H-adducte}]_e)^2} - ([\text{H-adduct}]_0 - [\text{H-adduct}])^2 \qquad \text{(Eq 24)}$$

With initial concentrations of $[\text{adduct}]_0 = a$ and $[\text{NHC}]_0 = [\text{aldehyde}]_0 = 0$, integration of Equation 24 gives Equation 25, where $x = ([\text{H-adduct}]_0 - [\text{H-adduct}])$ and $x_e = ([\text{H-adduct}]_0 - [\text{H-adduct}]_e)$.[5] Therefore the slope of a semilogarithmic plot of $(ax_e + x(a - x_e))/(a(x_e - x))$ against time divided by $(x_e)/(2a\text{-}x_e^2)$ can be used to estimate values for k_d in the build up to equilibrium. This analysis was not possible for the *N*-2,6-$(OMe)_2C_6H_3$ substituted adduct, as the position of the equilibrium lies very far towards 3-(hydroxybenzyl)azolium salt, making accurate measurements towards equilibrium difficult.

$$\frac{x_e}{(2a^2 - x_e)} \ln \frac{ax_e + x(a - x_e)}{a(x_e - x)} = k_d t \qquad \text{(Eq 25)}$$

Semilogarithmic plots of $(ax_e + x(a - x_e))/(a(x_e - x))$ for all adducts, followed over the first three half-lives, are shown in Figure S60, with k_d values obtained by application of Eqution 25.

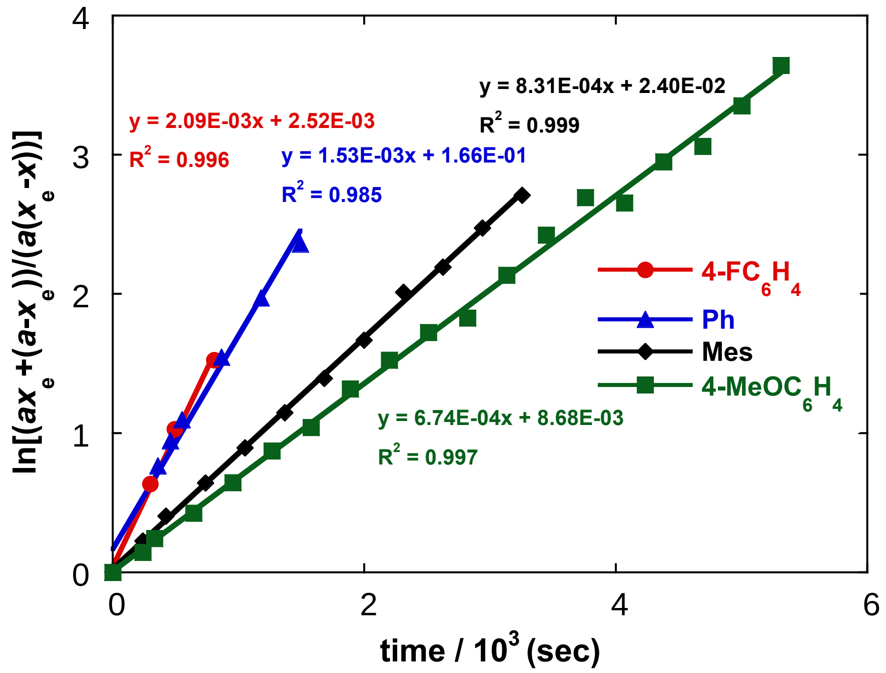

Figure S65. Semilogarithmic plots of $(ax_e + x(a - x_e))/(a(x_e - x))$ against time, obtained from partitioning experiments of 3-(hydroxybenzyl)azolium salts (0.04 M) in Et_3N:Et_3N·HCl and CD_3OD at 25 °C.

Additionally, values for the equilibrium constant, K^{diss} (M), can be estimated from the concentrations of species present once equilibrium had been reached using Equation 22. From the k_d and K^{diss} values, second order rate constants for adduct association (k_a, M^{-1} s^{-1}) were obtained.

Data for the initial dissociation up to equilibrium concentrations was also fitted using Berkeley Madonna, as previously described. Plots are shown below.

Table S2, Entry 6

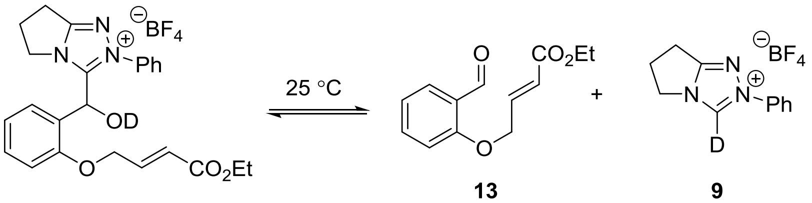

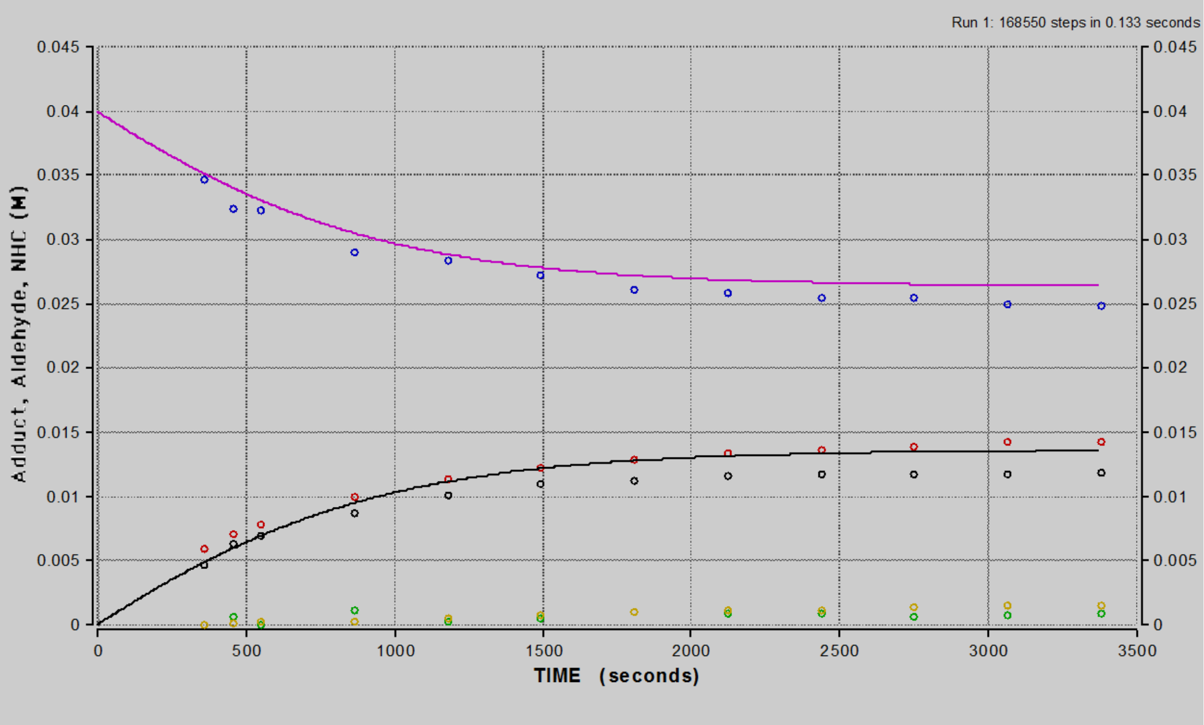

Figure S66. Plot showing 3-(hydroxybenzyl)azolium adduct dissociation at 25 °C, up to the equilibrium concentrations. Open circles show the experimental data, with the solid line representing the fit to the kinetic model. Fitting data from t = 0 to t = 3500 s from Figure S58.

Table S2, Entry 7

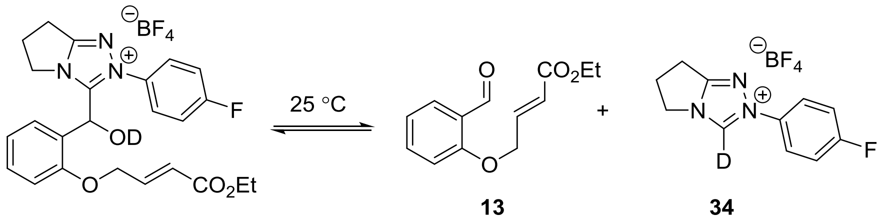

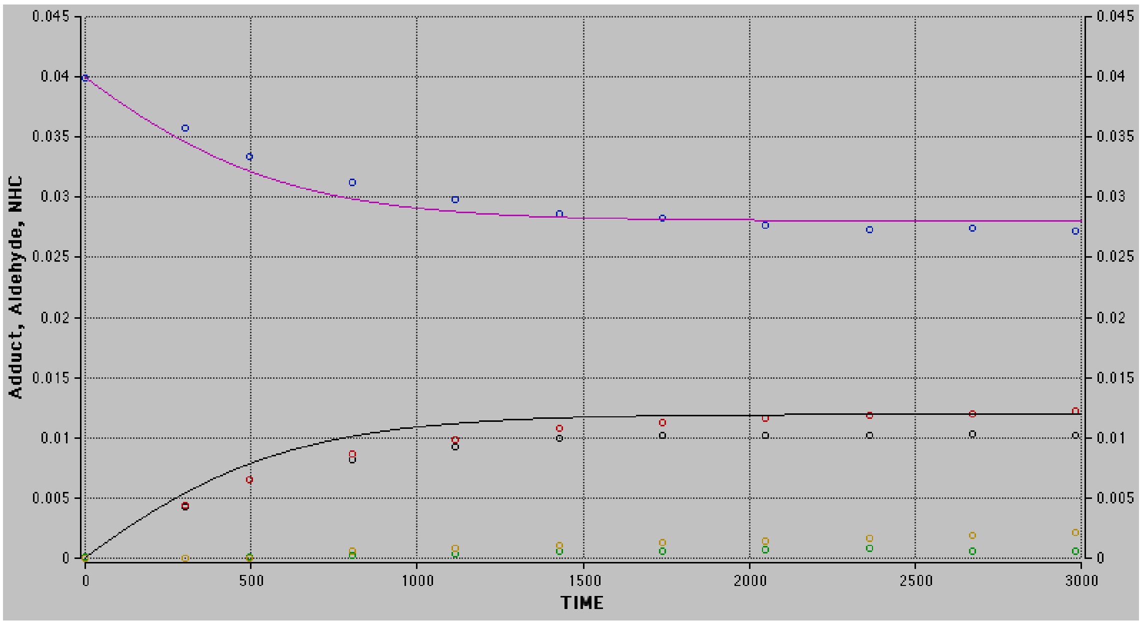

Figure S67. Plot showing 3-(hydroxybenzyl)azolium adduct dissociation at 25 °C, up to the equilibrium concentrations. Open circles show the experimental data, with the solid line representing the fit to the kinetic model. Fitting data from t = 0 to t = 3000 s from Figure S60.

Table S2, Entry 8

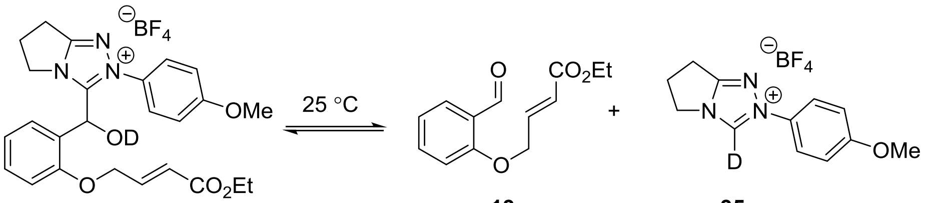

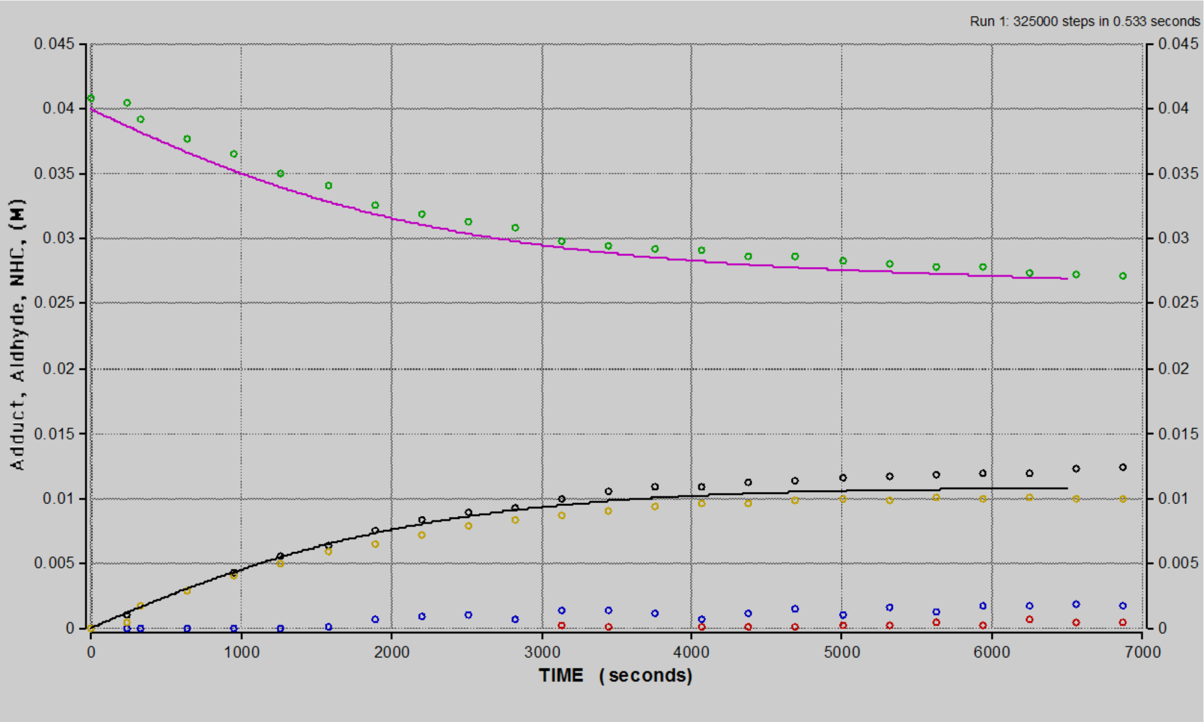

Figure S68. Plot showing 3-(hydroxybenzyl)azolium adduct dissociation at 25 °C, up to the equilibrium concentrations. Open circles show the experimental data, with the solid line representing the fit to the kinetic model. Fitting data from t = 0 to t = 7000 s from Figure S62.

Table S2, Entry 9

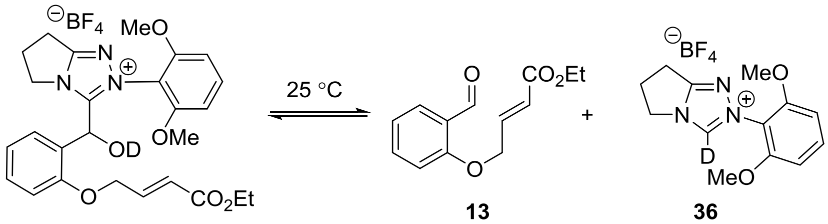

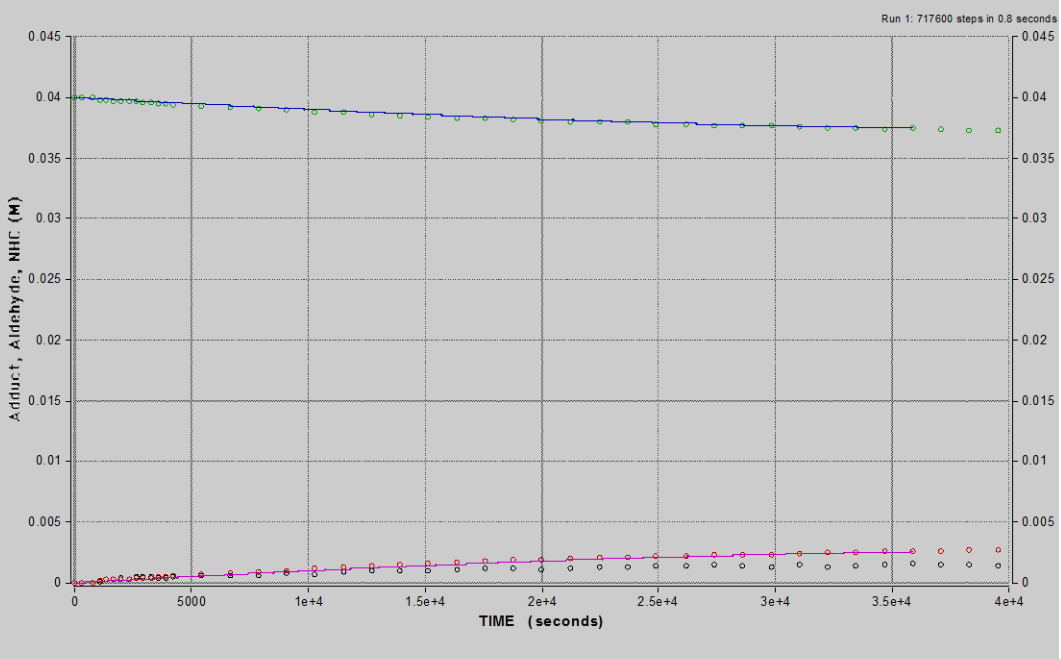

Figure S69. Plot showing 3-(hydroxybenzyl)azolium adduct dissociation at 25 °C, up to the equilibrium concentrations. Open circles show the experimental data, with the solid line representing the fit to the kinetic model.

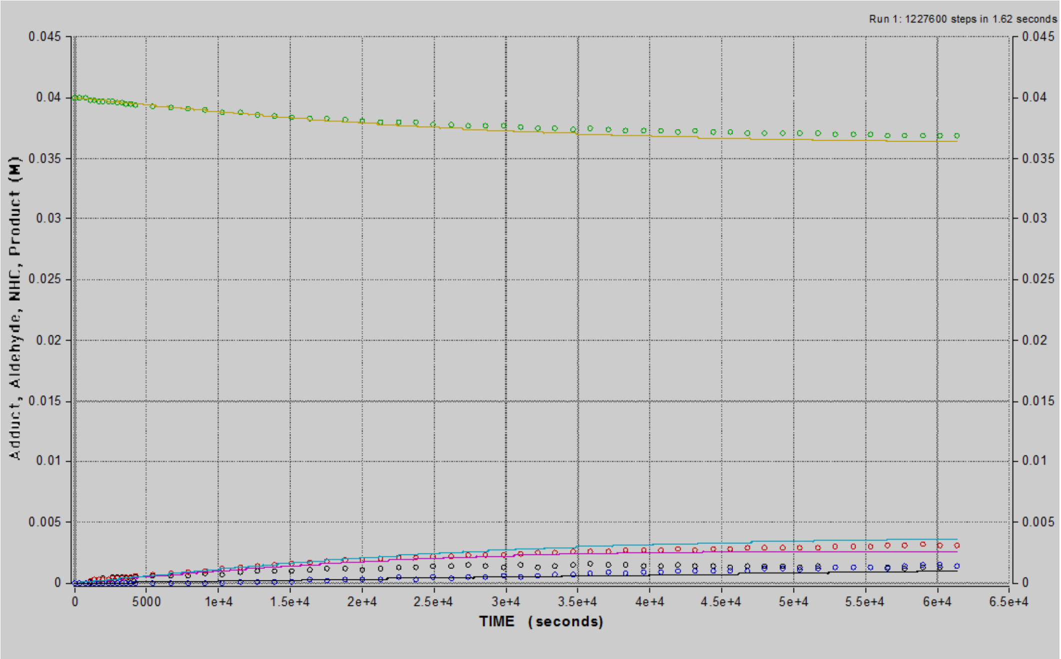

Figure S70. Plot showing results of fitting using Berkeley Madonna for 3-(hydroxybenzyl)azolium adduct dissociation at 25 °C. Open circles show the experimental data, with the solid line representing the fit to the kinetic model.

Table S2, Entry 10

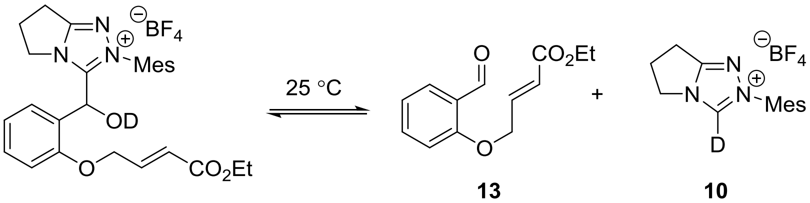

13 **10**

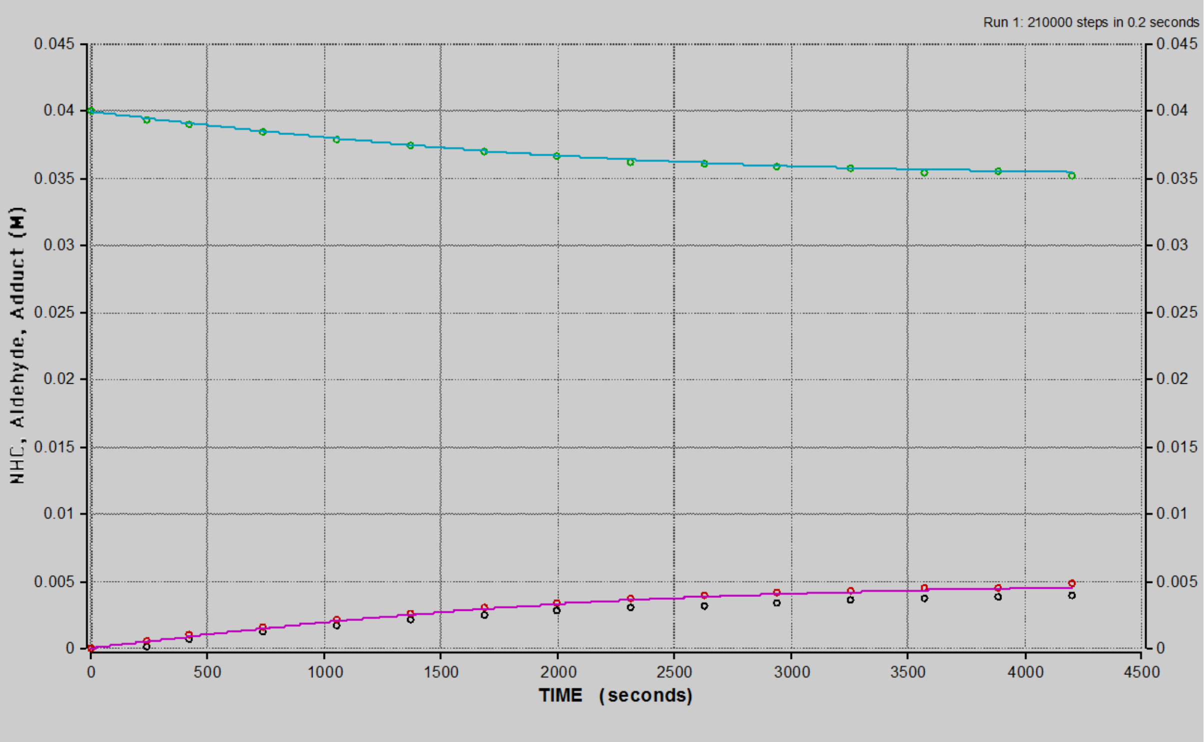

Figure S71. Plot showing 3-(hydroxybenzyl)azolium adduct dissociation at 25 °C, up to the equilibrium concentrations. Open circles show the experimental data, with the solid line representing the fit to the kinetic model. Fitting data from t = 0 to t = 4500 s from Figure S64.

Determination of Rate and Equilibrium Constants using Substituted Benzaldehydes in CD_3OD (Table 4, Table S4)

Data was obtained in a similar manner to the experiments in Table 2 using the appropriate substituted benzaldehyde and NHC precursor **9**.

Table 4, Table S4 Entry 1

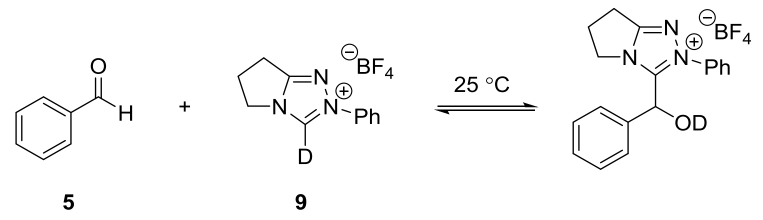

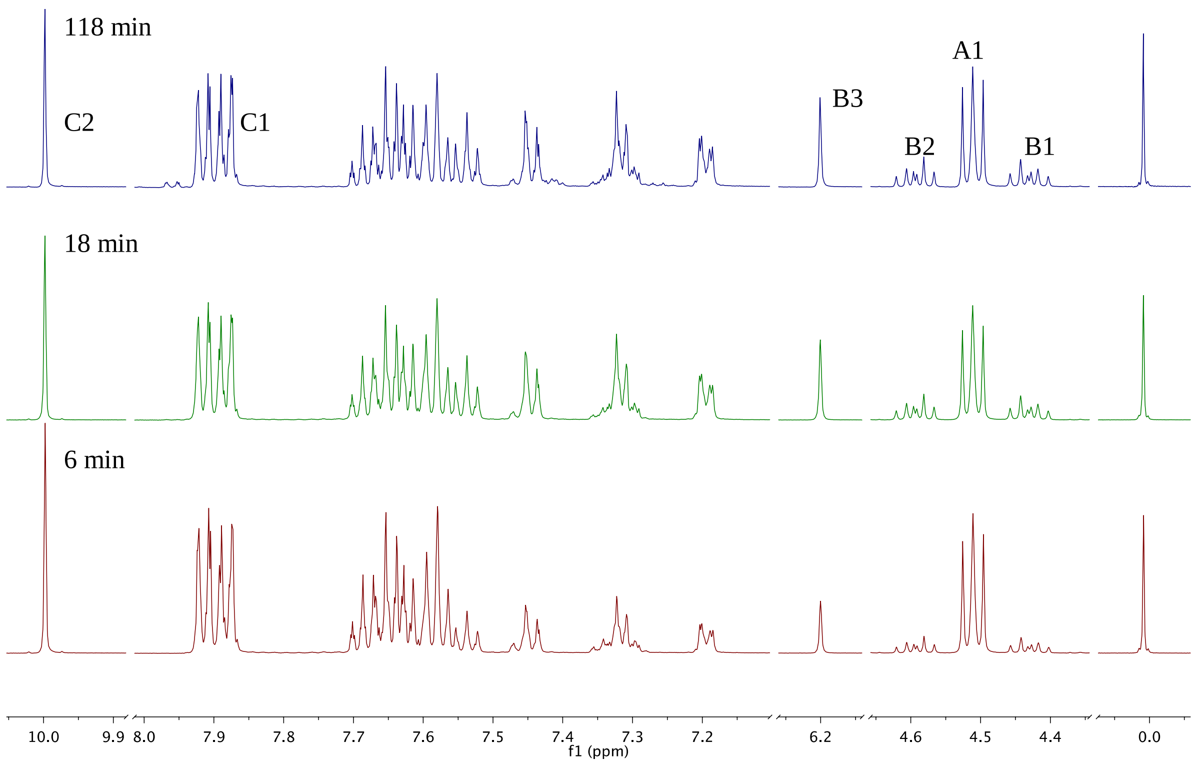

Figure S72. Representative ^{1}H NMR spectra (500 MHz) for the reaction of benzaldehyde **5** with *N*-Ph NHC precursor **9** in Et_3N:Et_3N·HCl and CD_3OD at 25 °C. A1 = NHC precursor C*H*$_2$, B1 = HB adduct C*H*H, B2 = HB adduct CH*H*, B3 = HB adduct C(α)*H*, C1 = Benzaldehyde aromatic *H*, C2 = PhC*H*O.

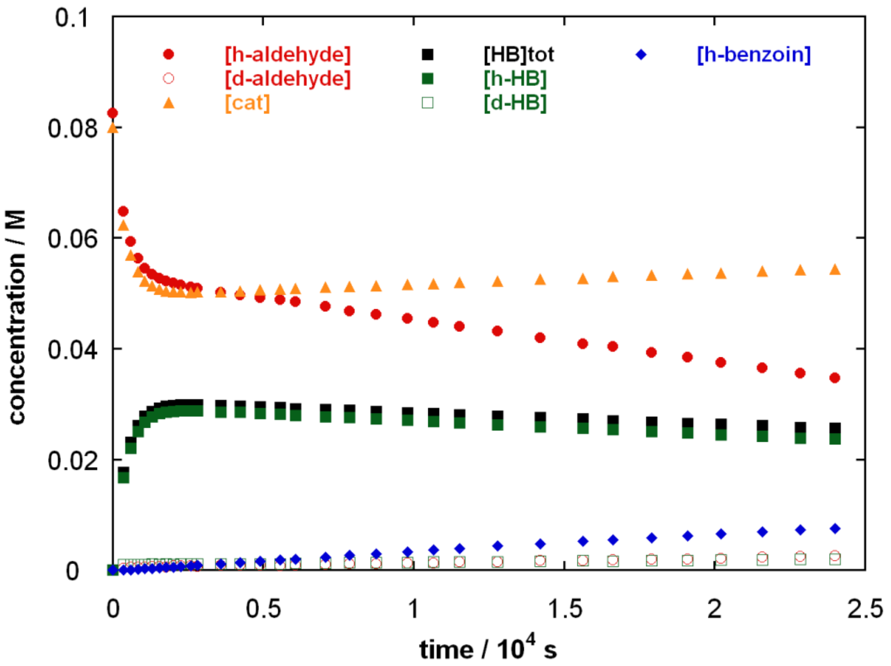

Figure S73. Reaction profile displaying concentration of species present against time for the reaction of benzaldehyde **5** with *N*-Ph NHC precursor **9** in Et_3N:$Et_3N·HCl$ and CD_3OD at 25 °C. [h-HB] = H-adduct; [d-HB] = D-adduct.

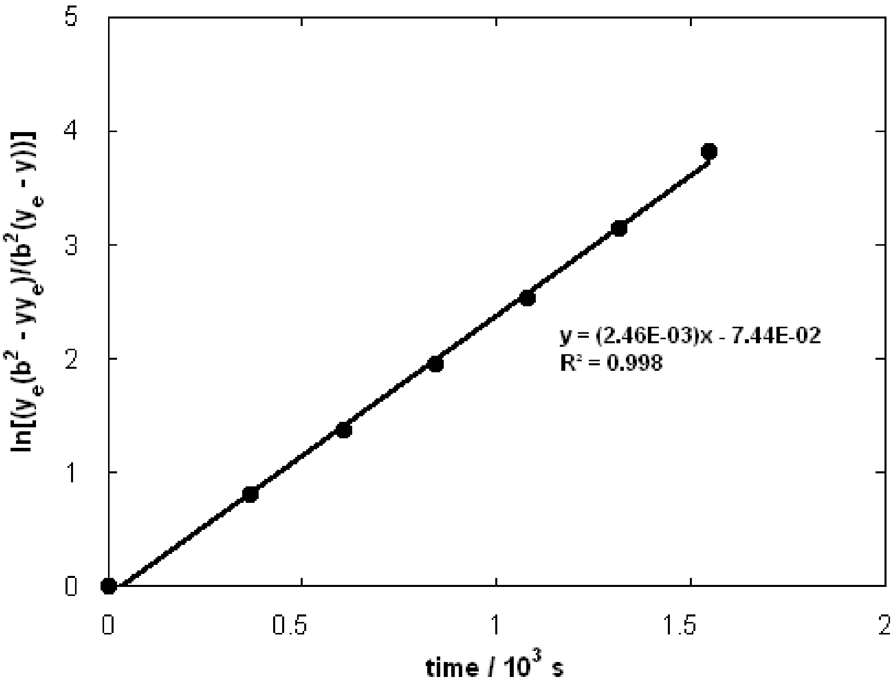

Figure S74. Semilogarithmic plots of $(y_e(b^2–yy_e)/(b^2(y_e–y))$ against time, obtained from the reaction of benzaldehyde **5** with *N*-Ph NHC precursor **9** in Et_3N:$Et_3N·HCl$ and CD_3OD at 25 °C.

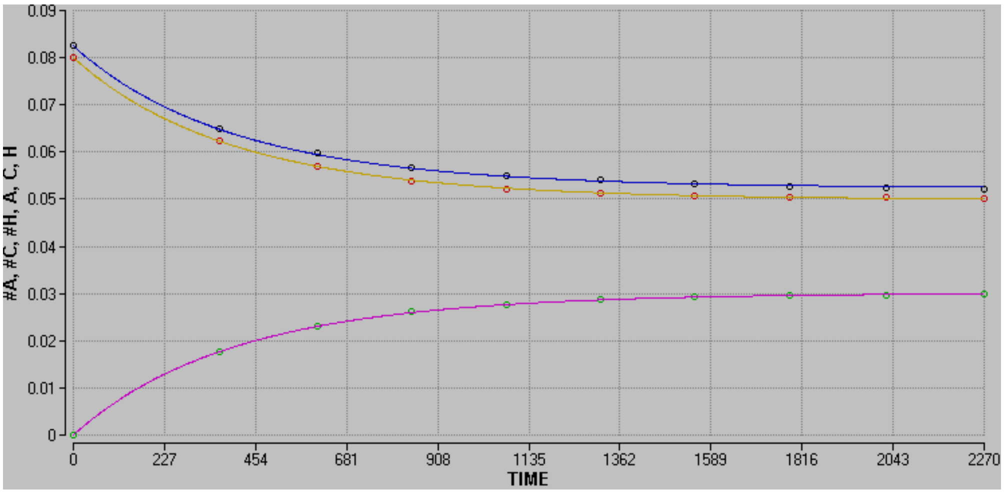

Figure S75. Plot showing the reaction of benzaldehyde **5** with *N*-Ph NHC precursor **9** in Et_3N:Et_3N·HCl and CD_3OD at 25 °C up to the equilibrium concentrations. Open circles show the experimental data, with the solid line representing the fit to the kinetic model. Fitting data from t = 0 to t = 2270 s from Figure S73.

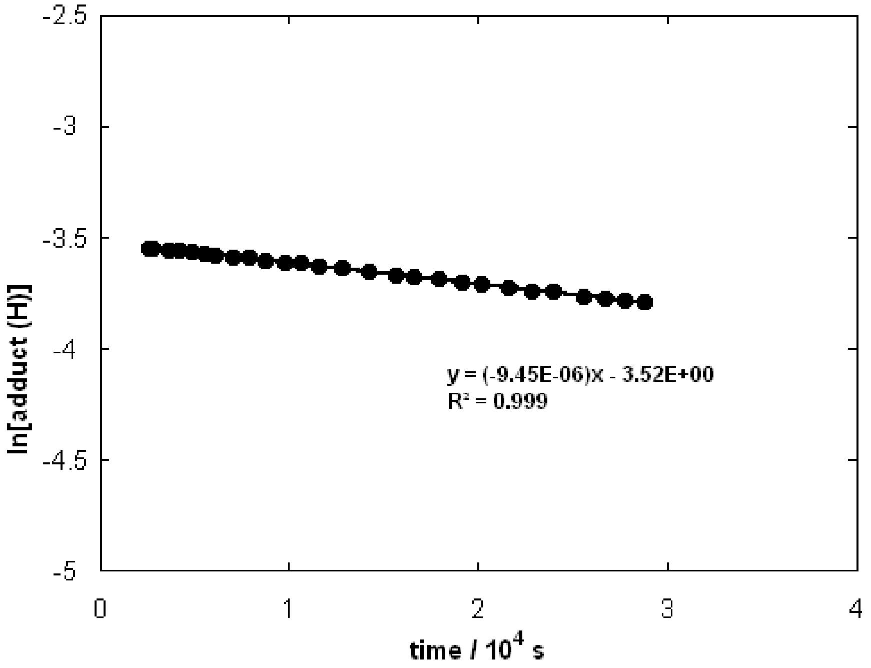

Figure S76. Plot of ln[H-adduct] against time used to determine k_2.

Table 4, Table S4 Entry 2

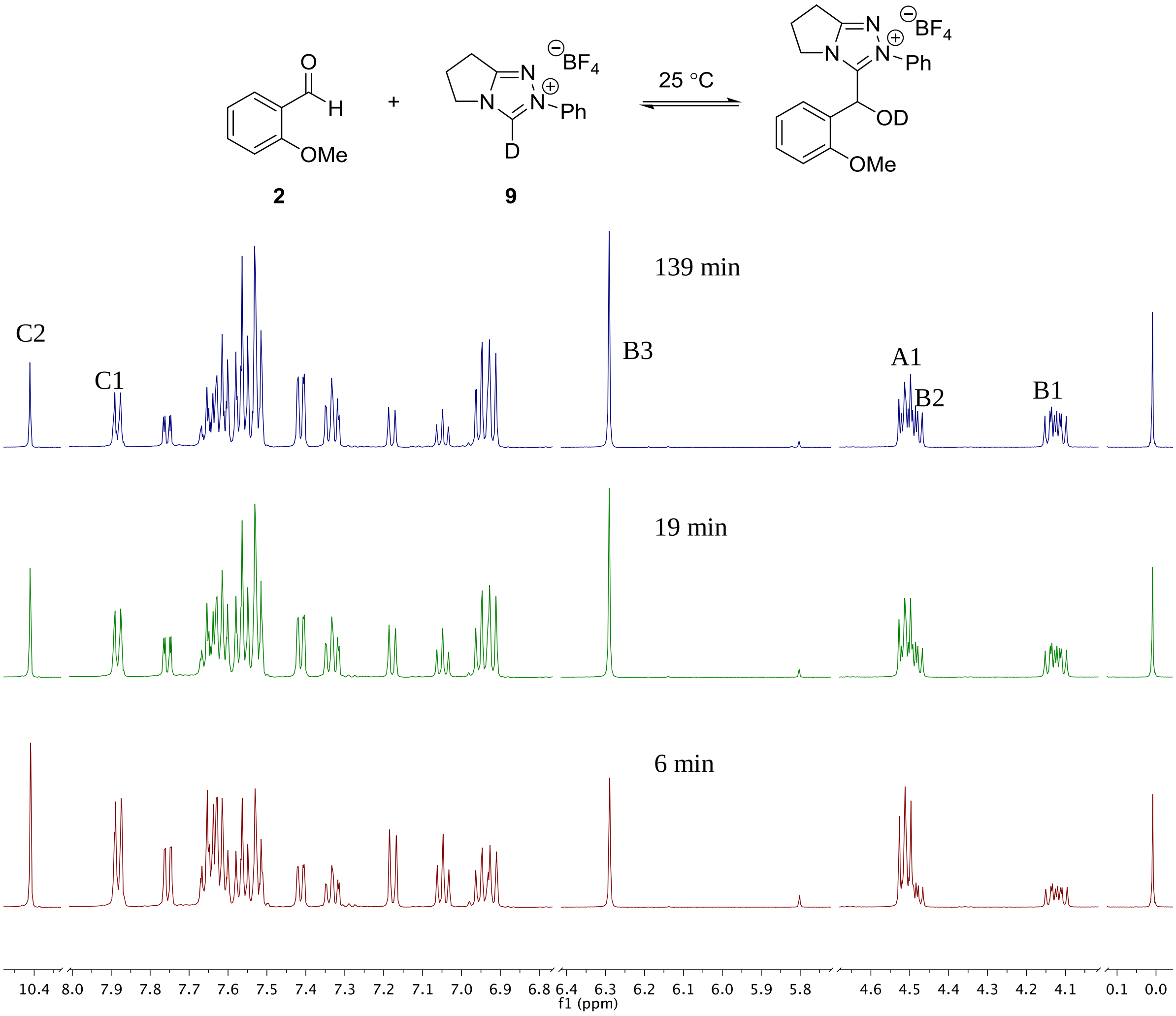

Figure S77. Representative ^{1}H NMR spectra (500 MHz) for the reaction of 2-methoxybenzaldehyde **2** with *N*-Ph NHC precursor **9** in Et_3N:Et_3N·HCl and CD_3OD at 25 °C. A1 = NHC precursor CH_2, B1 = HB adduct C*H*H, B2 = HB adduct CH*H*, B3 = HB adduct C(α)*H*, C1 = 2MeO-PhCHO aromatic *H*, C2 = 2MeO-PhC*H*O.

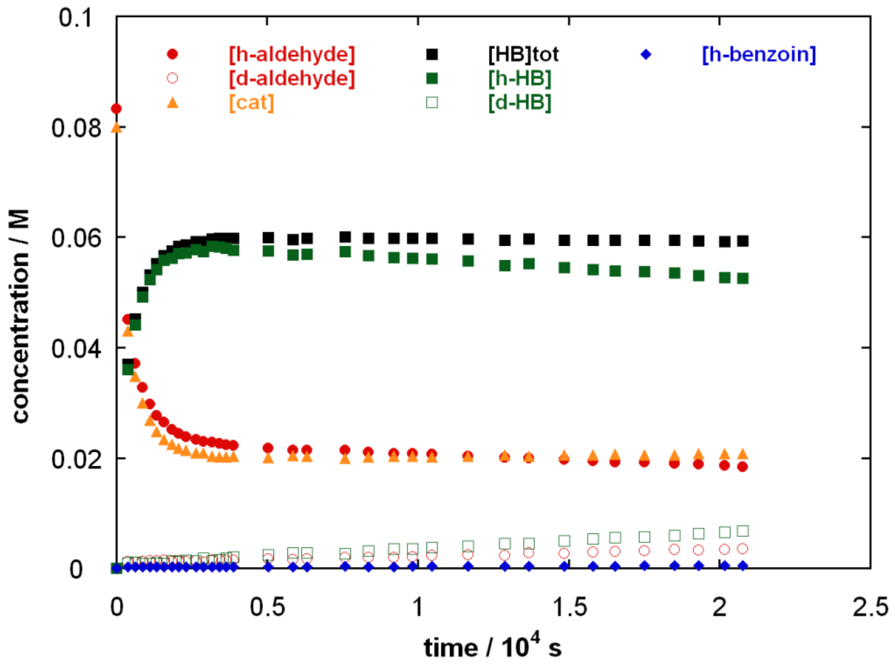

Figure S78. Reaction profile displaying concentration of species present against time for the reaction of 2-methoxybenzaldehyde **2** with *N*-Ph NHC precursor **9** in Et_3N:$Et_3N{\cdot}HCl$ and CD_3OD at 25 °C. [h-HB] = H-adduct; [d-HB] = D-adduct.

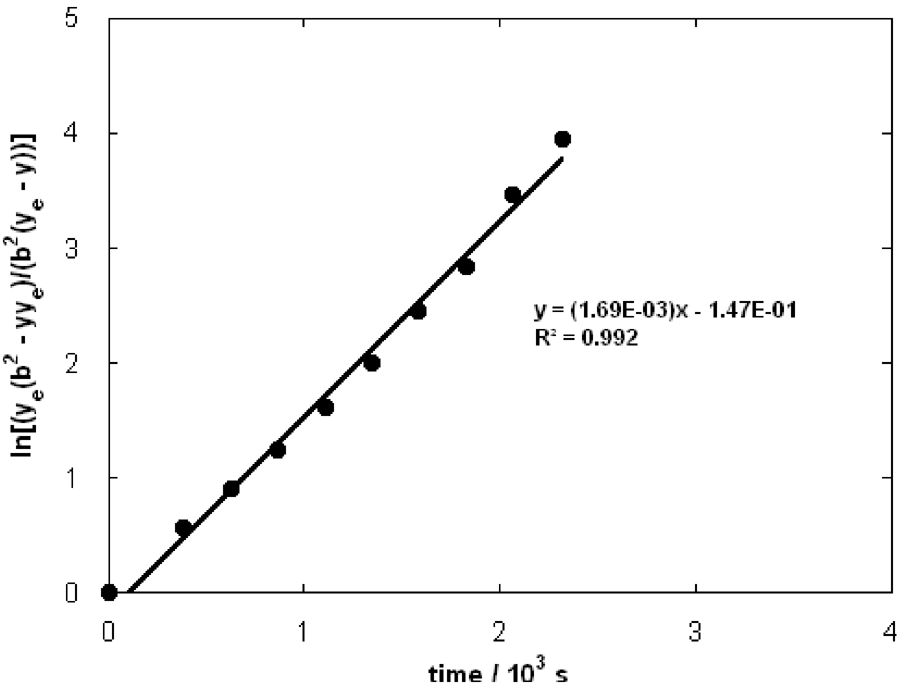

Figure S79. Semilogarithmic plots of $(y_e(b^2–yy_e)/(b^2(y_e–y))$ against time, obtained from the reaction of 2-methoxybenzaldehyde **2** with *N*-Ph NHC precursor **9** in Et_3N:$Et_3N{\cdot}HCl$ and CD_3OD at 25 °C.

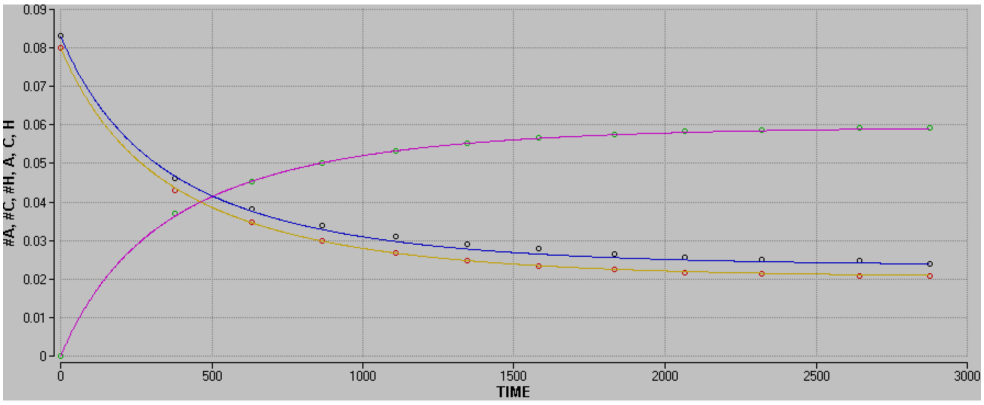

Figure S80. Plot showing the reaction of 2-methoxybenzaldehyde **2** with *N*-Ph NHC precursor **9** in Et_3N:$Et_3N{\cdot}HCl$ and CD_3OD at 25 °C up to the equilibrium concentrations. Open circles show the experimental data, with the solid line representing the fit to the kinetic model. Fitting data from t = 0 to t = 3000 s from Figure S78.

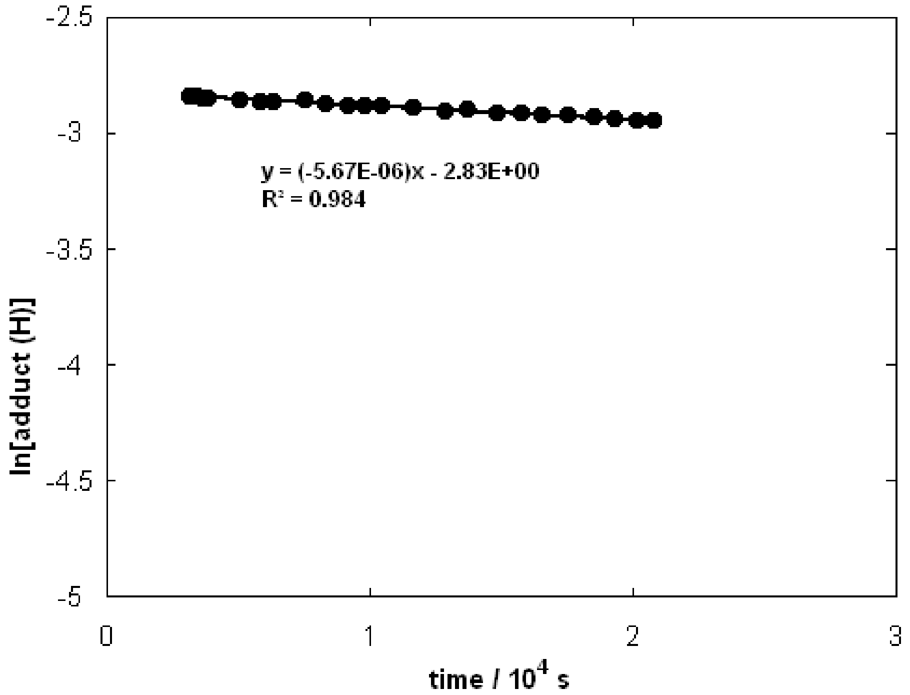

Figure S81. Plot of ln[H-adduct] against time used to determine k_2.

Table 4, Table S4 Entry 3

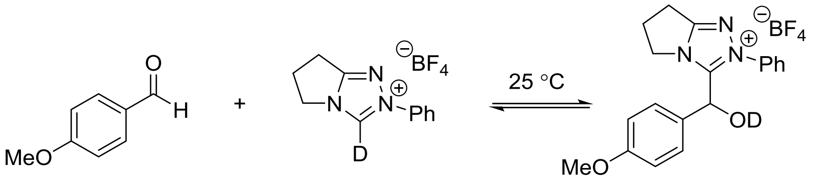

9

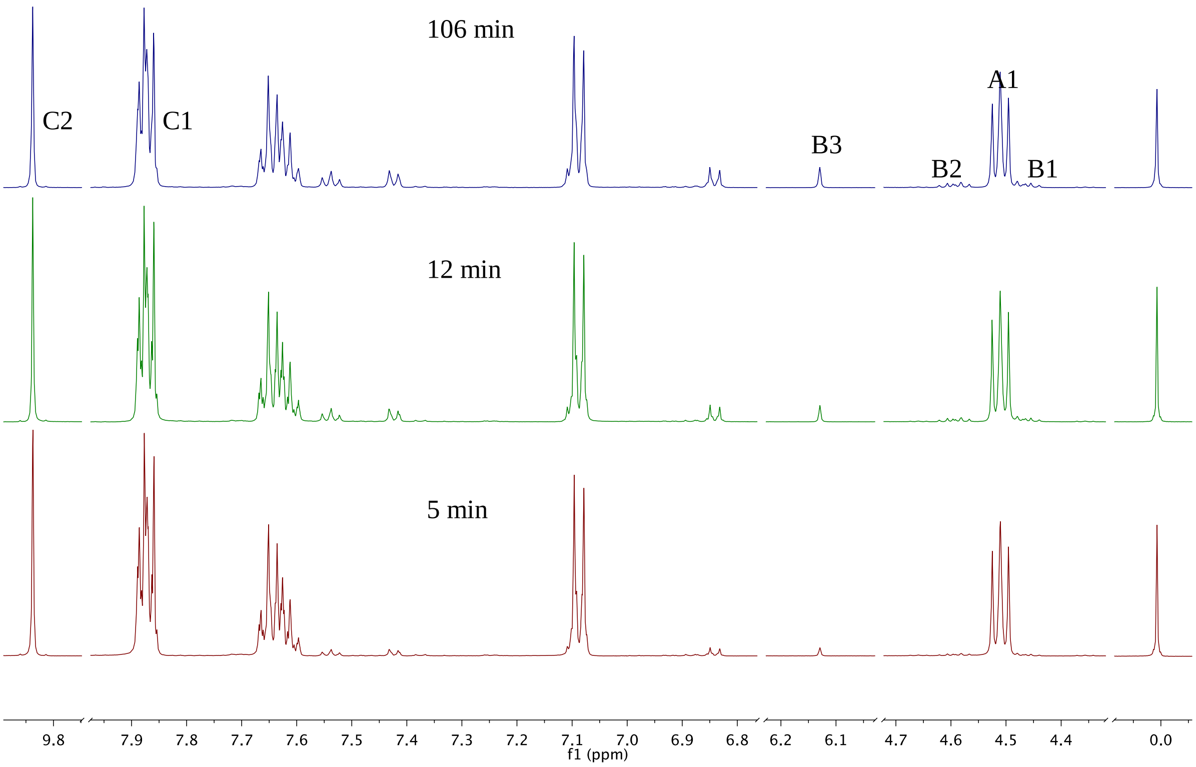

Figure S82. Representative ^{1}H NMR spectra (500 MHz) for the reaction of 4-methoxybenzaldehyde with *N*-Ph NHC precursor **9** in Et_3N:Et_3N·HCl and CD_3OD at 25 °C. A1 = NHC precursor CH_2, B1 = HB adduct C*H*H, B2 = HB adduct CH*H*, B3 = HB adduct C(α)*H*, C1 = 4MeO-PhCHO aromatic *H*, C2 = 4MeO-PhC*H*O.

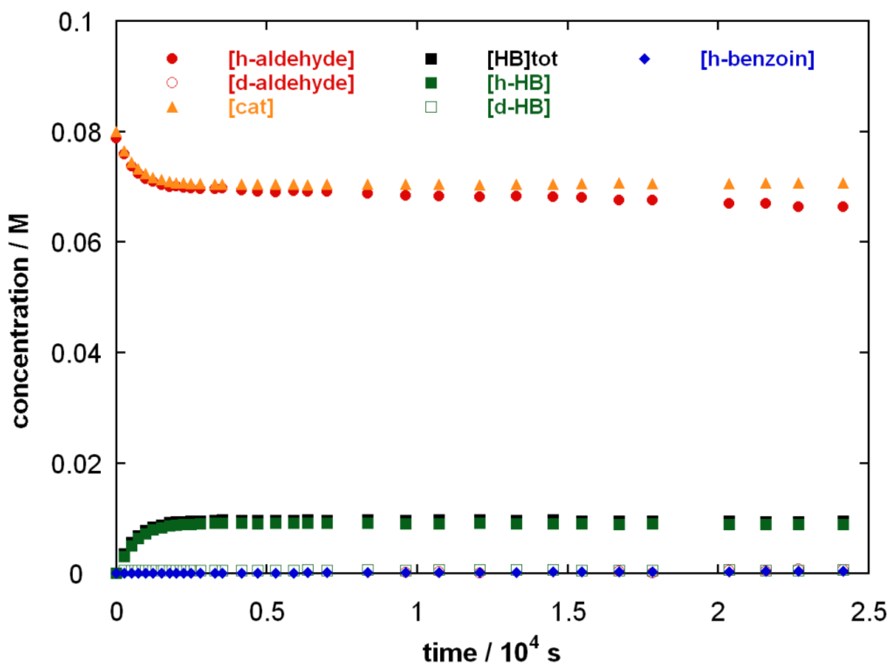

Figure S83. Reaction profile displaying concentration of species present against time for the reaction of 4-methoxybenzaldehyde with *N*-Ph NHC precursor **9** in Et_3N:$Et_3N\cdot HCl$ and CD_3OD at 25 °C. [h-HB] = H-adduct; [d-HB] = D-adduct.

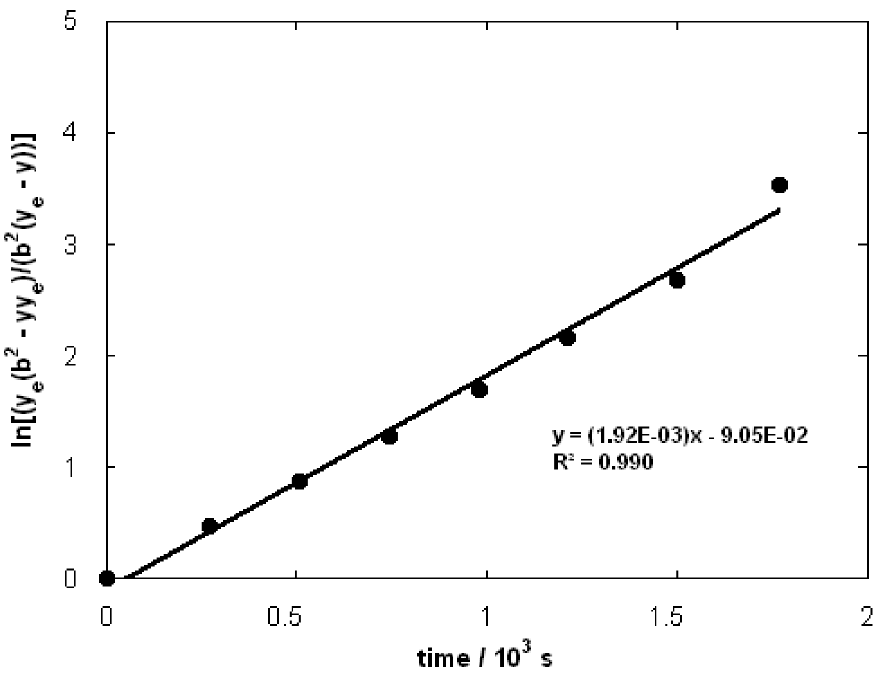

Figure S84. Semilogarithmic plots of $(y_e(b^2{-}yy_e)/(b^2(y_e{-}y))$ against time, obtained from the reaction of 4-methoxybenzaldehyde with *N*-Ph NHC precursor **9** in Et_3N:$Et_3N\cdot HCl$ and CD_3OD at 25 °C.

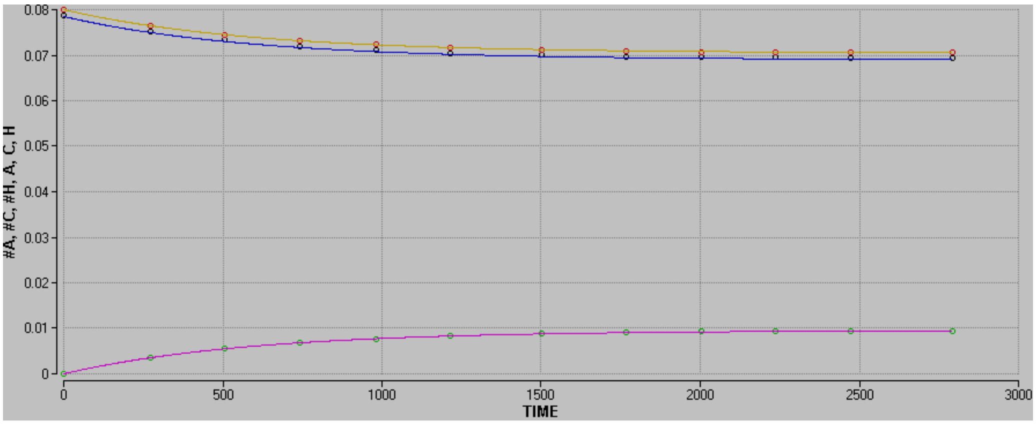

Figure S85. Plot showing the reaction of 4-methoxybenzaldehyde with *N*-Ph NHC precursor **9** in Et_3N:Et_3N·HCl and CD_3OD at 25 °C up to the equilibrium concentrations. Open circles show the experimental data, with the solid line representing the fit to the kinetic model. Fitting data from t = 0 to t = 3000 s from Figure S83.

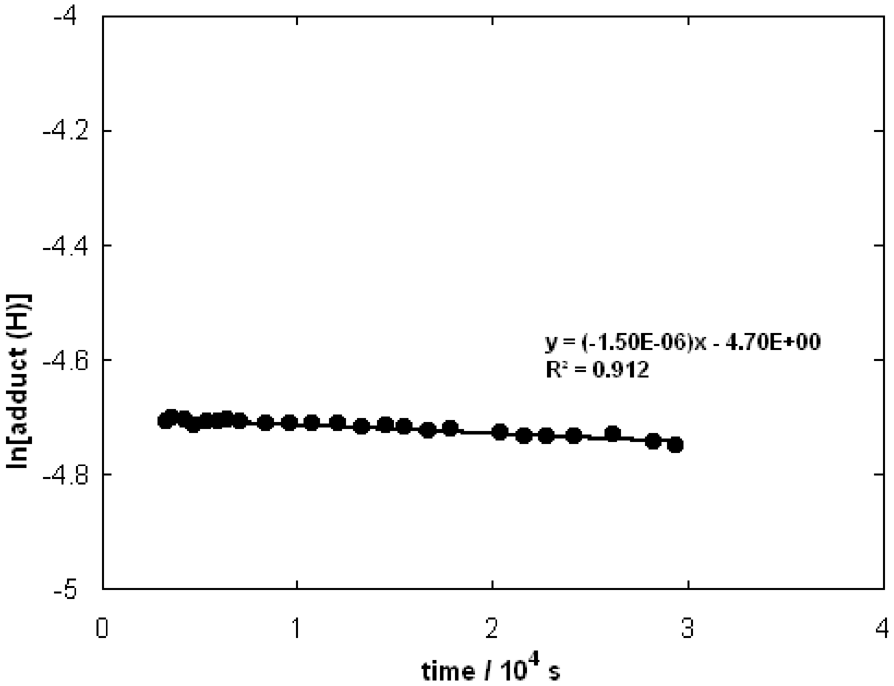

Figure S86. Plot of ln[H-adduct] against time used to determine k_2.

Table 4, Table S4 Entry 4

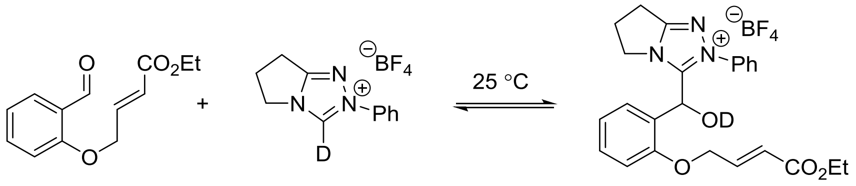

13 **9**

Representative spectra, reaction profile and information for determination of k_1, k_{-1} and K^{exp} were described in the information for Table S2 Entry 6.

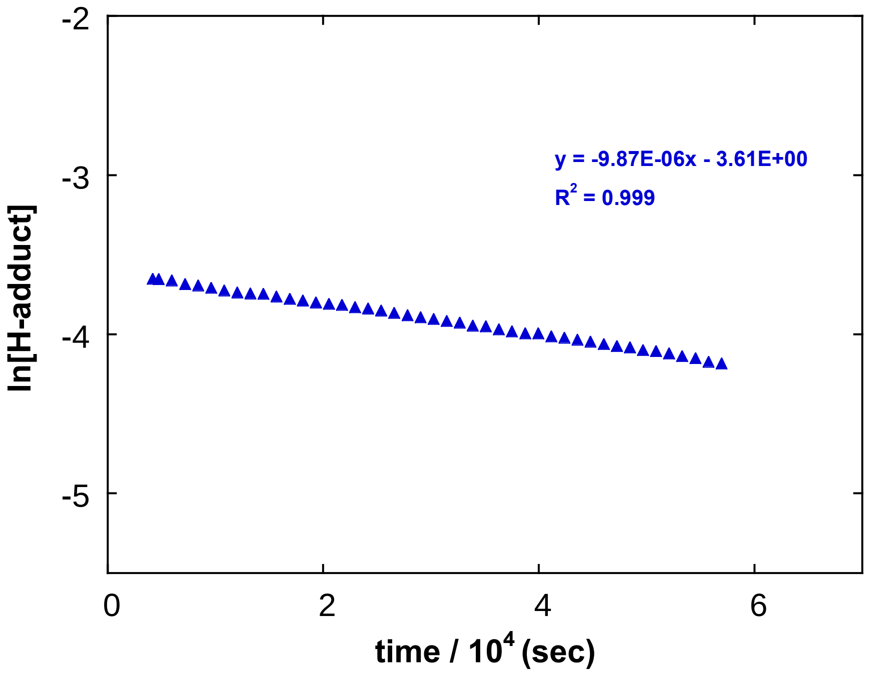

Figure S87. Plot of ln[H-adduct] against time used to determine k_2.

Table 4, Table S4 Entry 5

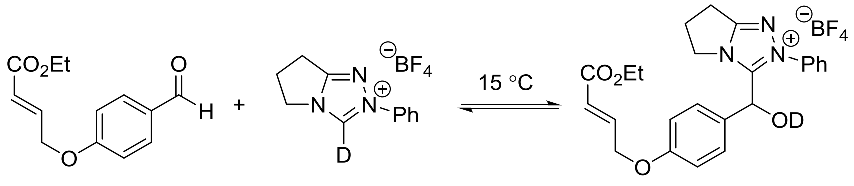

S1 **9**

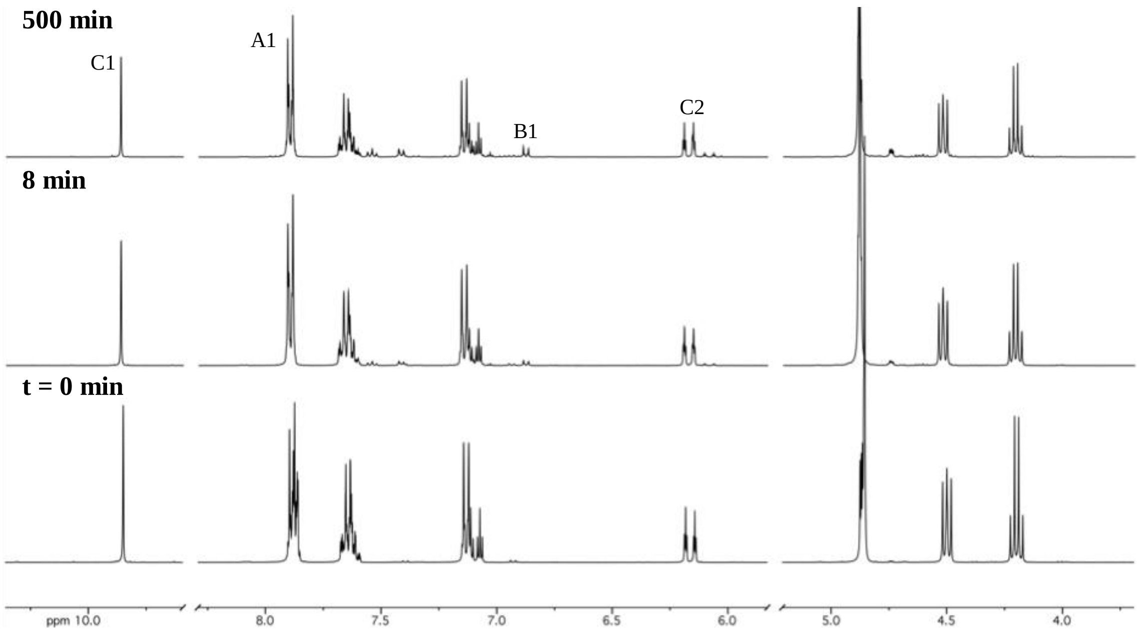

Figure S88. ^{1}H NMR spectra for the reaction of aldehyde **S1** with *N*-Ph NHC precursor **9** in Et_3N:Et_3N·HCl and CD_3OD at 15 °C. A1 = NHC precursor, B1 = HB adduct, C1 = aldehyde C*H*O, C2 = aldehyde.

The concentration of NHC precursor was determined using the signal at 7.90 ppm (A1), which corresponds to two aromatic protons on the phenyl ring. The concentration was calculated *via* Equation 26, relative to the total amount of aldehyde-derived species (C2 + B1).

$$[\text{catalyst}] = \frac{1}{f_{\text{ald}}} \times \frac{(A_{\text{A1}}/2)}{(A_{\text{C2}} + A_{\text{B1}})} \times 0.04 \qquad \text{(Eq 26)}$$

The concentration of aldehyde was determined in a similar fashion to previous examples, using Equation 27, using the singlet at 9.86 ppm (C1), correcting for the hemi-acetal equilibrium (f_{ald} = 0.985).

$$[\text{aldehyde}] = \frac{1}{f_{\text{ald}}} \times \frac{A_{\text{C1}}}{(A_{\text{C2}} + A_{\text{B1}})} \times 0.04 \qquad \text{(Eq 27)}$$

The total concentration of 3-(hydroxybenzyl)azolium salt was obtained from the doublet of triplets at 6.88 ppm, using Equation 28.

$$[\text{adduct}]_{\text{tot}} = \frac{1}{f_{\text{ald}}} \times \frac{A_{\text{B1}}}{(A_{\text{C2}} + A_{\text{B1}})} \times 0.04 \qquad \text{(Eq 28)}$$

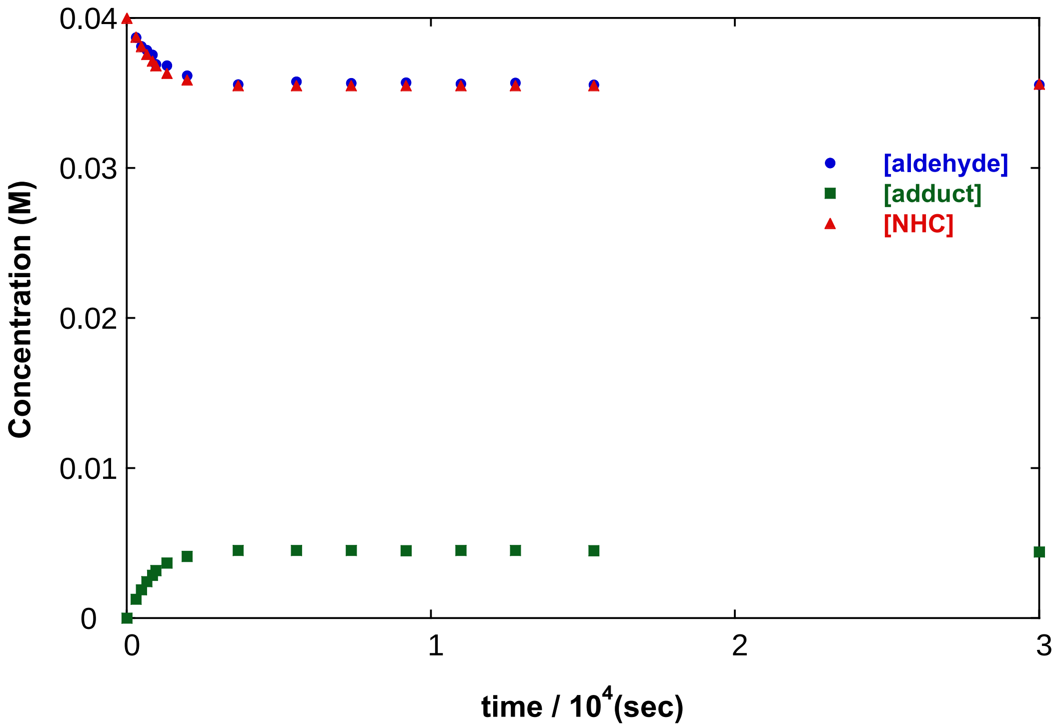

Figure S89. Reaction profile displaying concentration of species present against time for the reaction of aldehyde **S1** with *N*-Ph NHC precursor **9** in Et_3N:Et_3N·HCl and CD_3OD at 15 °C.

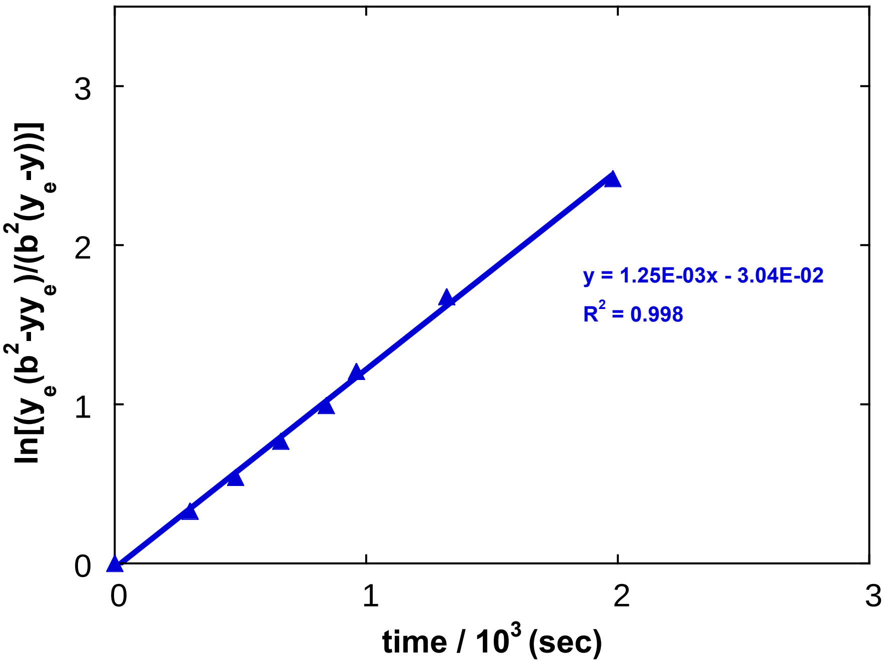

Figure S90. Semilogarithmic plots of $(y_e(b^2–yy_e)/(b^2(y_e–y))$ against time, obtained from the reaction of aldehyde **S1** with *N*-Ph NHC precursor **9** in Et_3N:Et_3N·HCl and CD_3OD at 15 °C.

Table 4, Table S4 Entry 6

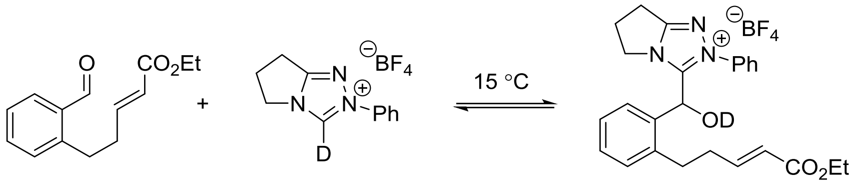

S3 **9**

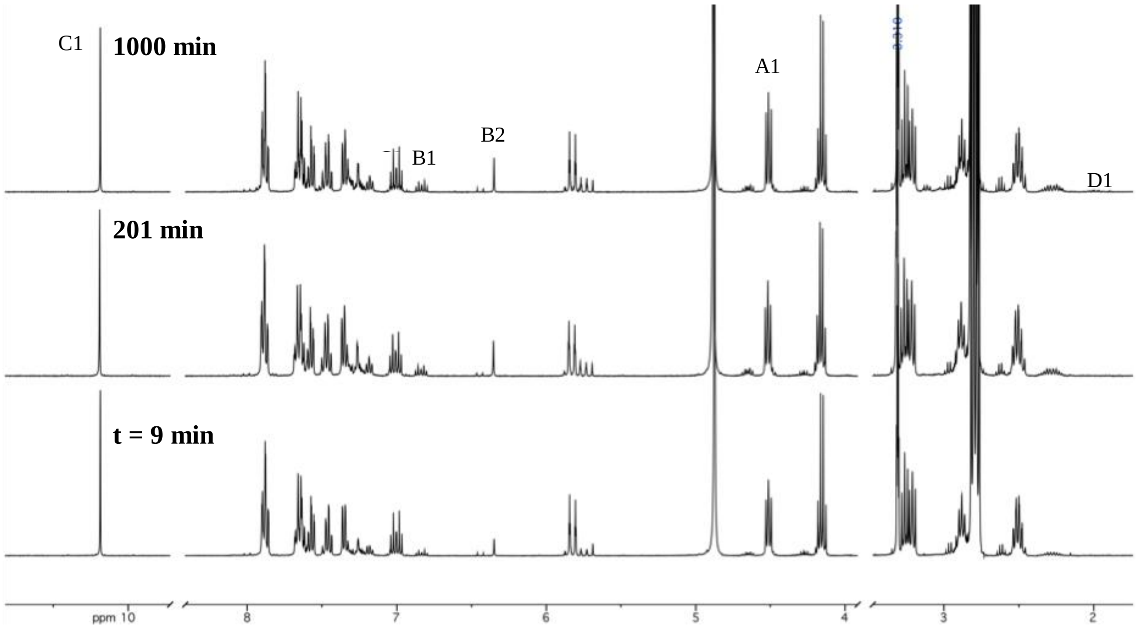

Figure S91. Representative ^{1}H NMR spectra (500 MHz) for the reaction of aldehyde **S3** with *N*-Ph NHC precursor **9** in Et_3N:Et_3N·HCl and CD_3OD at 15 °C. A1 = NHC precursor, B1 = HB adduct, B2 = HB adduct, C1 = aldehyde C*H*O, D1 = product.

The concentration of aldehyde was determined in a similar fashion to previous examples, using the singlet at 10.18 ppm, correcting for the hemi-acetal equilibrium (f_{ald} = 0.910). The concentration was calculated *via* Equation 29, relative to the total amount of aldehyde-derived species (C2 + B1 + D1).

$$[\text{aldehyde}] = \frac{1}{f_{\text{ald}}} \times \frac{A_{\text{C1}}}{(A_{\text{C2}} + A_{\text{B1}} + A_{\text{D1}})} \times 0.04 \qquad \text{(Eq 29)}$$

The total concentration of 3-(hydroxybenzyl)azolium salt was obtained from the doublet of triplets at 6.83 ppm, using Equation 30.

$$[\text{adduct}]_{\text{tot}} = \frac{1}{f_{\text{ald}}} \times \frac{A_{\text{B1}}}{(A_{\text{C2}} + A_{\text{B1}} + A_{\text{D1}})} \times 0.04 \qquad \text{(Eq 30)}$$

The concentration of NHC precursor was determined using the triplet at 4.49 ppm, corresponding to the backbone CH_2 (A1). However, there was overlap with the same proton on the 3-(hydroxybenzyl)azolium so a correction was made to account for this (Equation 31).

$$[\text{catalyst}] = \frac{((A_{\text{A1}}/2) - A_{\text{B1}})}{(A_{\text{C2}} + A_{\text{B1}} + A_{\text{D1}})} \times 0.04 \qquad \text{(Eq 31)}$$

The presence of deuterated 3-(hydroxybenzyl)azolium salt was checked for using the signal at 6.35 ppm (corresponding to the C(α)-H) and Equations 32 and 33, as previously described. However, none was observed over the course of the experiment.

$$[\text{H-adduct}] = \frac{A_{\text{B2}}}{(A_{\text{C2}} + A_{\text{B1}} + A_{\text{D1}})} \times 0.04 \qquad \text{(Eq 32)}$$

$$[\text{D-adduct}] = [\text{adduct}]_{\text{tot}} - [\text{H-adduct}] \qquad \text{(Eq 33)}$$

The amount of Stetter product was assigned using the signal at 1.98 ppm (D1), allowing [product] to be calculated *via* Equation 34.

$$[\text{product}] = \frac{A_{\text{D1}}}{(A_{\text{C2}} + A_{\text{B1}} + A_{\text{D1}})} \times 0.04 \qquad \text{(Eq 34)}$$

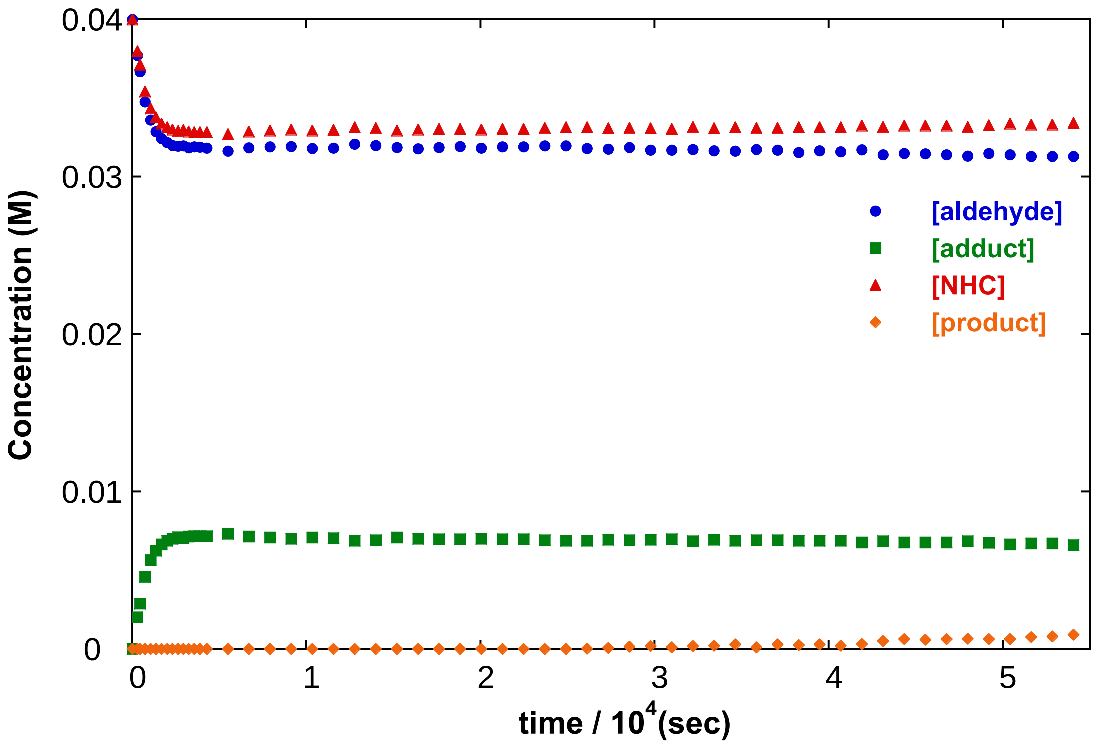

Figure S92. Reaction profile displaying concentration of species present against time for the reaction of aldehyde **S3** with *N*-Ph NHC precursor **9** in Et_3N:Et_3N·HCl and CD_3OD at 15 °C.

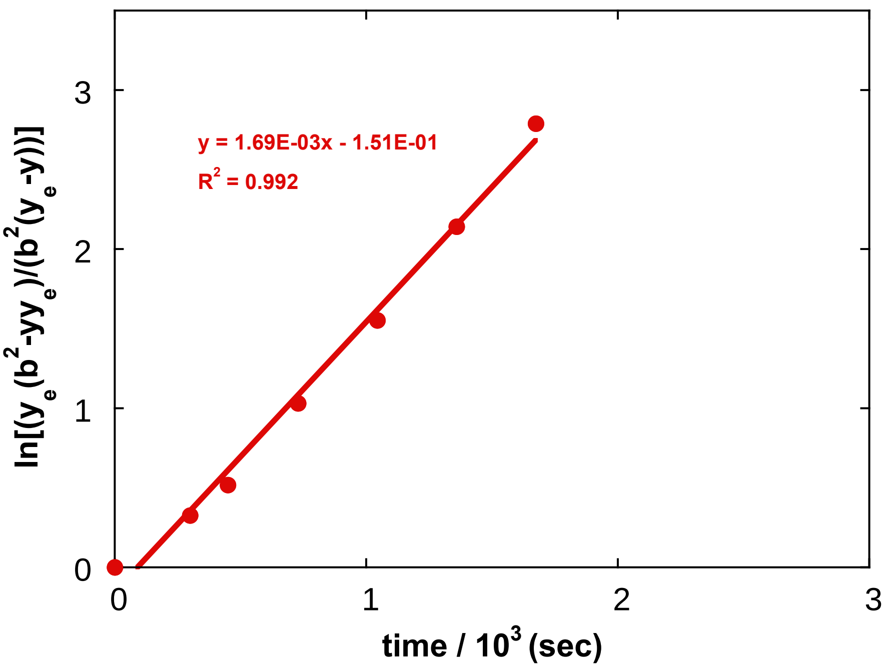

Figure S93. Semilogarithmic plot of $(y_e(b^2–yy_e)/(b^2(y_e–y))$ against time, obtained from the reaction of aldehyde **S3** with *N*-Ph NHC precursor **9** in Et_3N:Et_3N·HCl and CD_3OD at 15 °C.

Table 4, Table S4 Entry 7

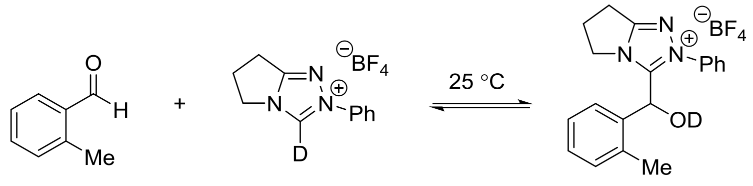

12 **9**

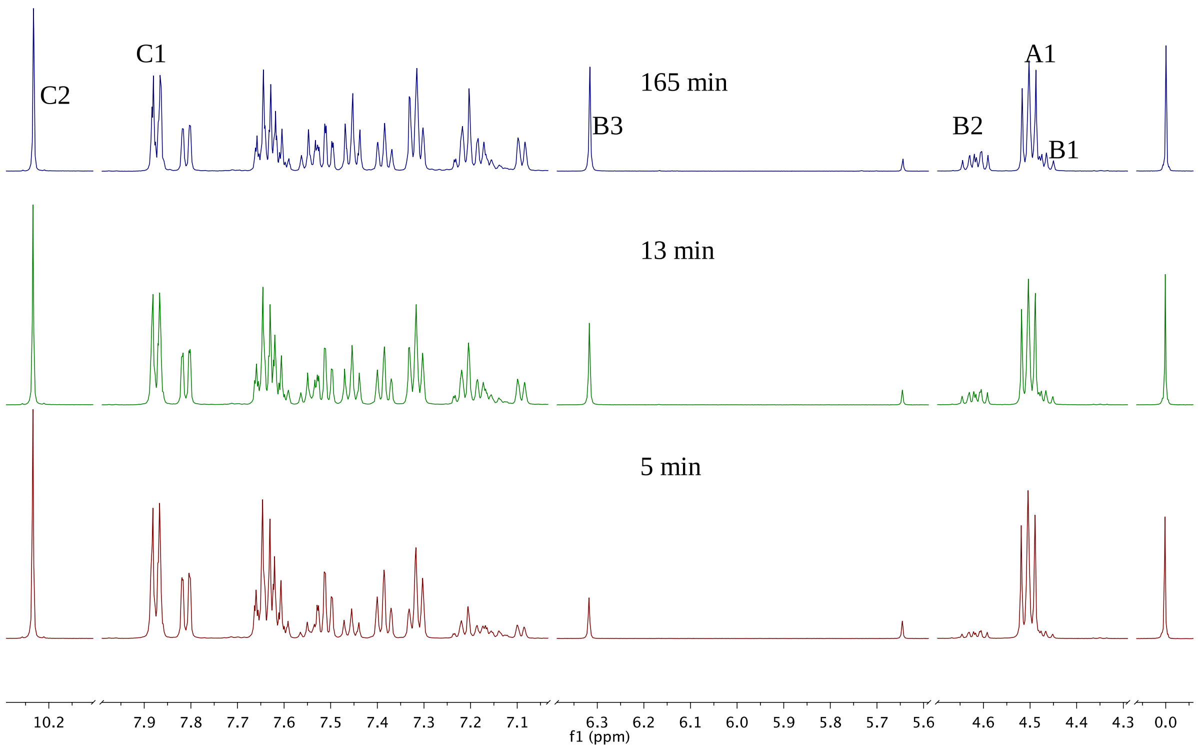

Figure S94. Representative ^{1}H NMR spectra (500 MHz) for the reaction of 2-tolualdehyde **12** with *N*-Ph NHC precursor **9** in Et_3N:Et_3N·HCl and CD_3OD at 25 °C. A1 = NHC precursor CH_2, B1 = HB adduct C*H*H, B2 = HB adduct CH*H*, B3 = HB adduct C(α)*H*, C1 = 2Me-PhCHO aromatic *H*, C2 = 2Me-PhC*H*O.

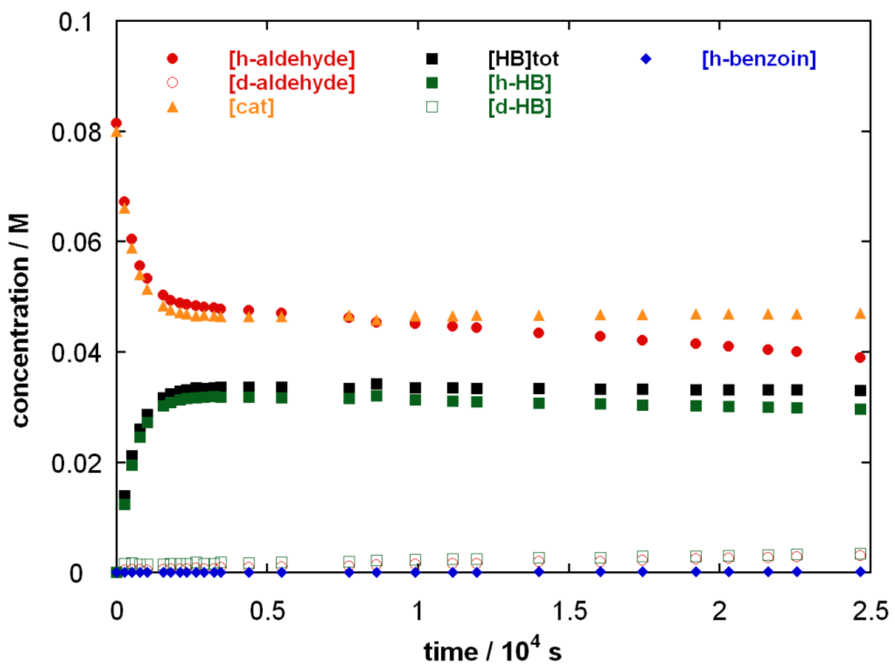

Figure S95. Reaction profile displaying concentration of species present against time for the reaction of 2-tolualdehyde **12** with *N*-Ph NHC precursor **9** in Et_3N:$Et_3N·HCl$ and CD_3OD at 25 °C. [h-HB] = H-adduct; [d-HB] = D-adduct.

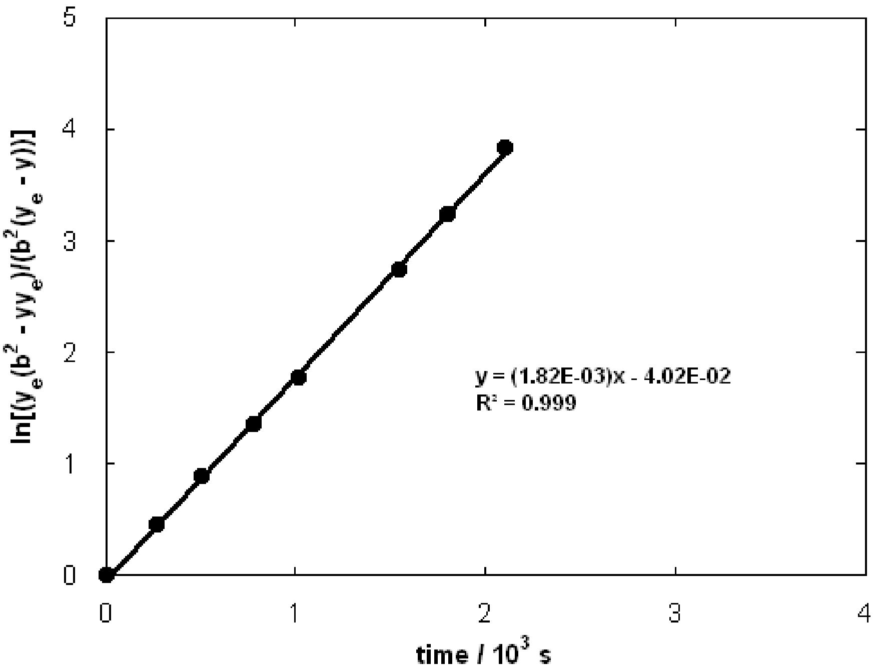

Figure S96. Semilogarithmic plots of $(y_e(b^2–yy_e)/(b^2(y_e–y))$ against time, obtained from the reaction of 2-tolualdehyde **12** with *N*-Ph NHC precursor **9** in Et_3N:$Et_3N·HCl$ and CD_3OD at 25 °C.

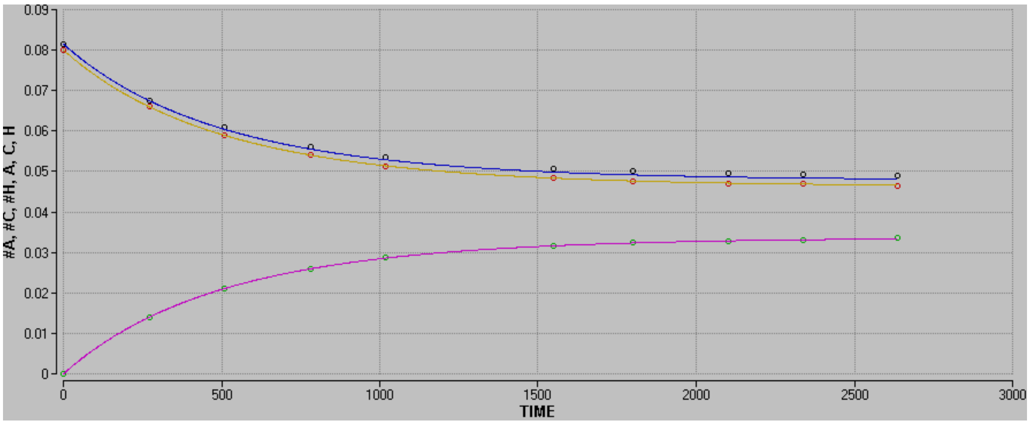

Figure S97. Plot showing the reaction of 2-tolualdehyde **12** with *N*-Ph NHC precursor **9** in Et_3N:Et_3N·HCl and CD_3OD at 25 °C up to the equilibrium concentrations. Open circles show the experimental data, with the solid line representing the fit to the kinetic model. Fitting data from t = 0 to t = 3000 s from Figure S95.

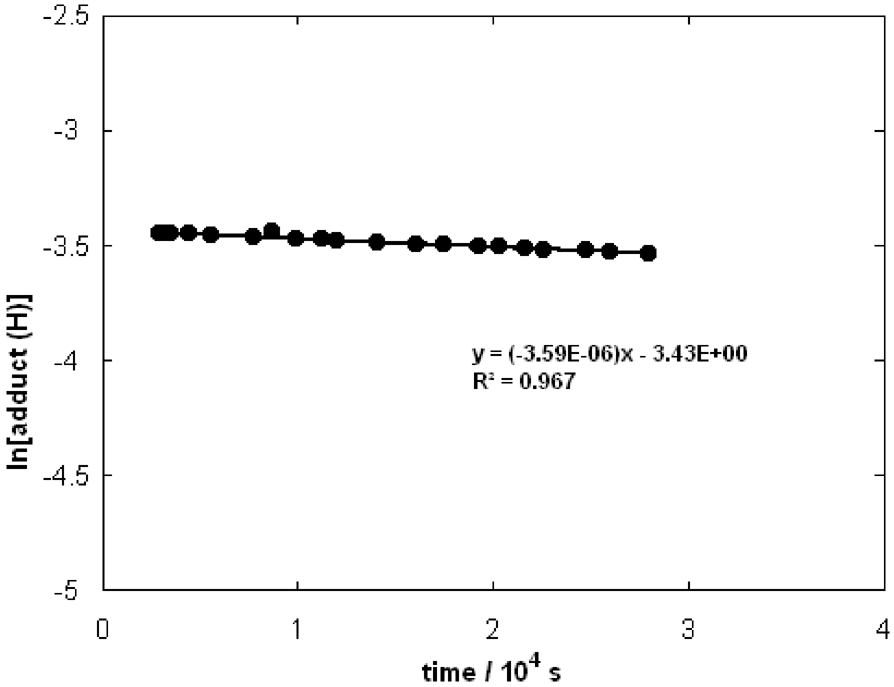

Figure S98. Plot of ln[H-adduct] against time used to determine k_2.

Table 4, Table S4 Entry 8

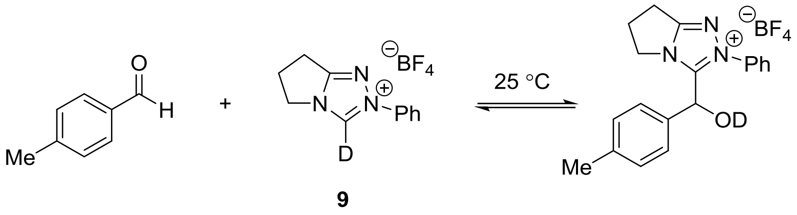

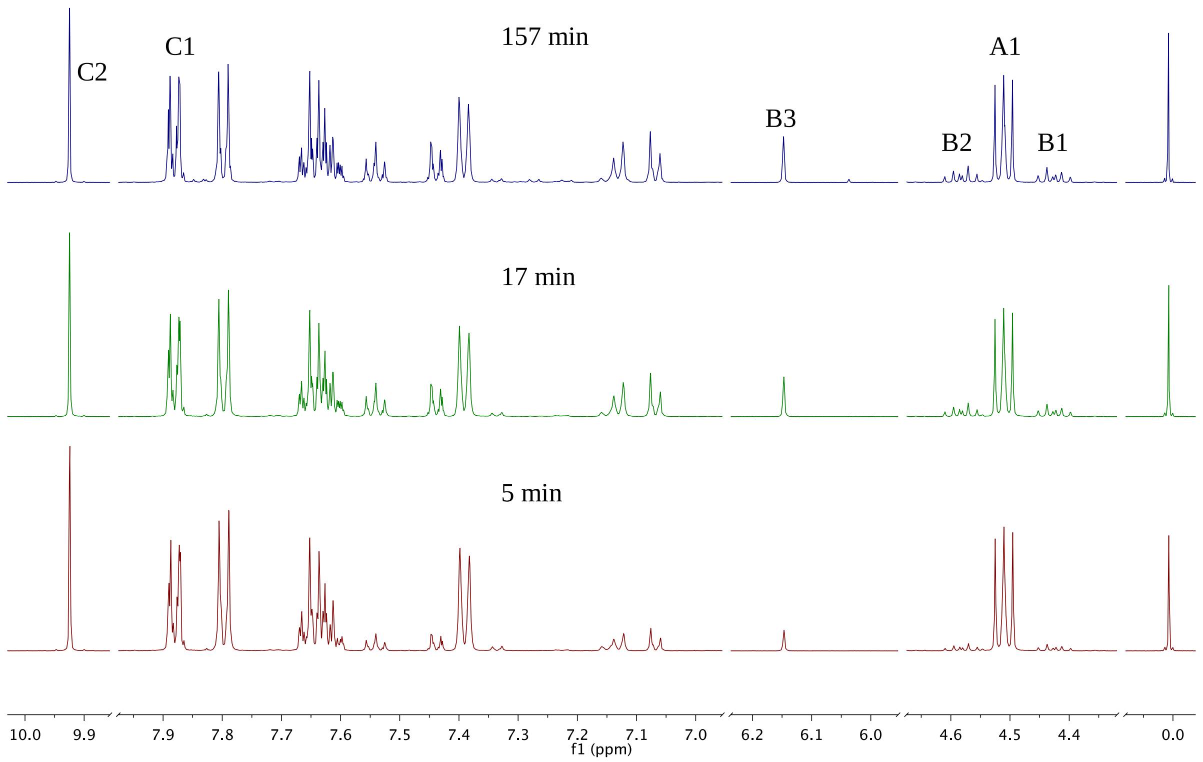

Figure S99. Representative ^{1}H NMR spectra (500 MHz) for the reaction of 4-tolualdehyde with *N*-Ph NHC precursor **9** in Et_3N:Et_3N·HCl and CD_3OD at 25 °C. A1 = NHC precursor CH_2, B1 = HB adduct C*H*H, B2 = HB adduct CH*H*, B3 = HB adduct C(α)*H*, C1 = 4Me-PhCHO aromatic *H*, C2 = 4Me-PhC*H*O.

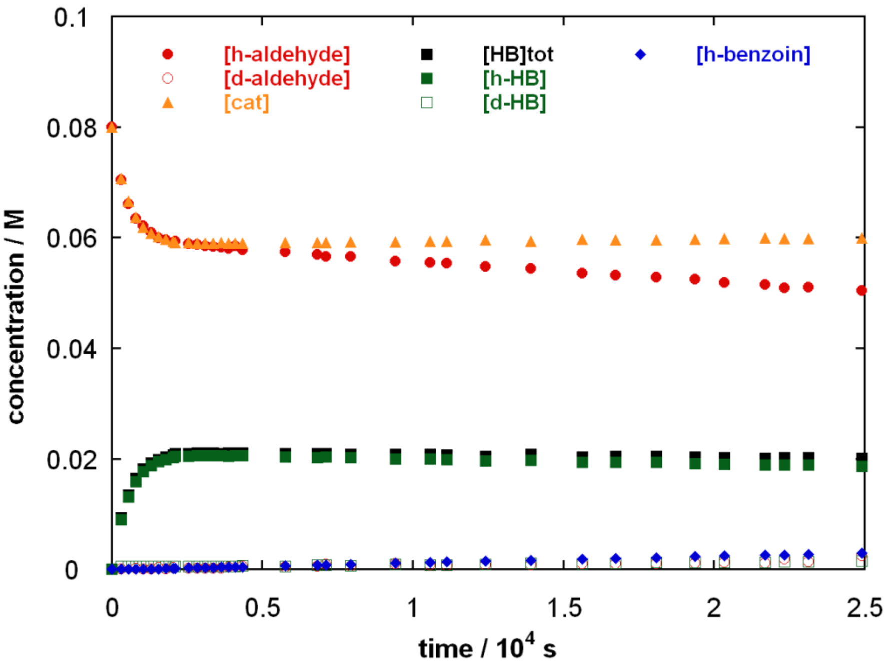

Figure S100. Reaction profile displaying concentration of species present against time for the reaction of 4-tolualdehyde with *N*-Ph NHC precursor **9** in Et_3N:Et_3N·HCl and CD_3OD at 25 °C. [h-HB] = H-adduct; [d-HB] = D-adduct.

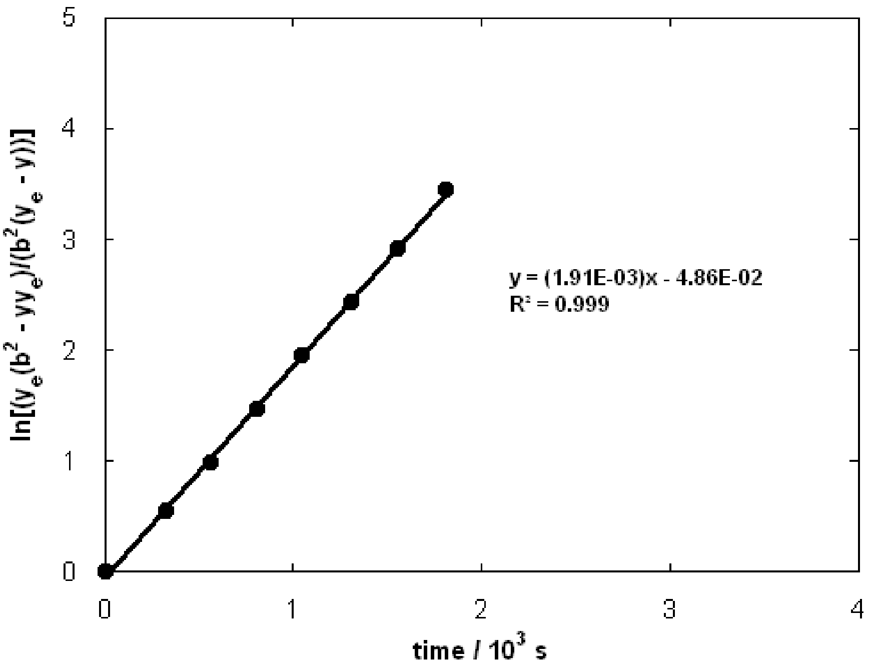

Figure S101. Semilogarithmic plots of $(y_e(b^2–yy_e)/(b^2(y_e–y))$ against time, obtained from the reaction of 4-tolualdehyde with *N*-Ph NHC precursor **9** in Et_3N:Et_3N·HCl and CD_3OD at 25 °C.

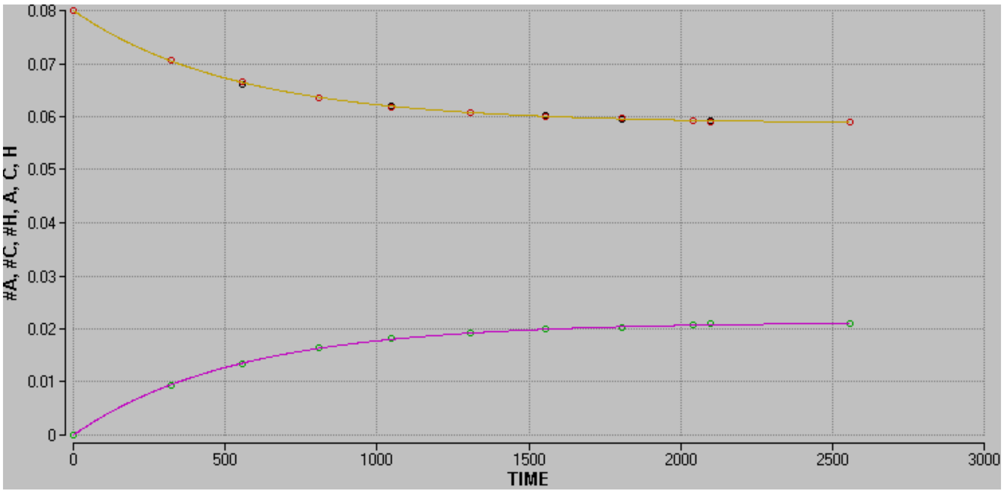

Figure S102. Plot showing the reaction of 4-tolualdehyde with *N*-Ph NHC precursor **9** in Et_3N:$Et_3N·HCl$ and CD_3OD at 25 °C up to the equilibrium concentrations. Open circles show the experimental data, with the solid line representing the fit to the kinetic model. Fitting data from t = 0 to t = 3000 s from Figure S100.

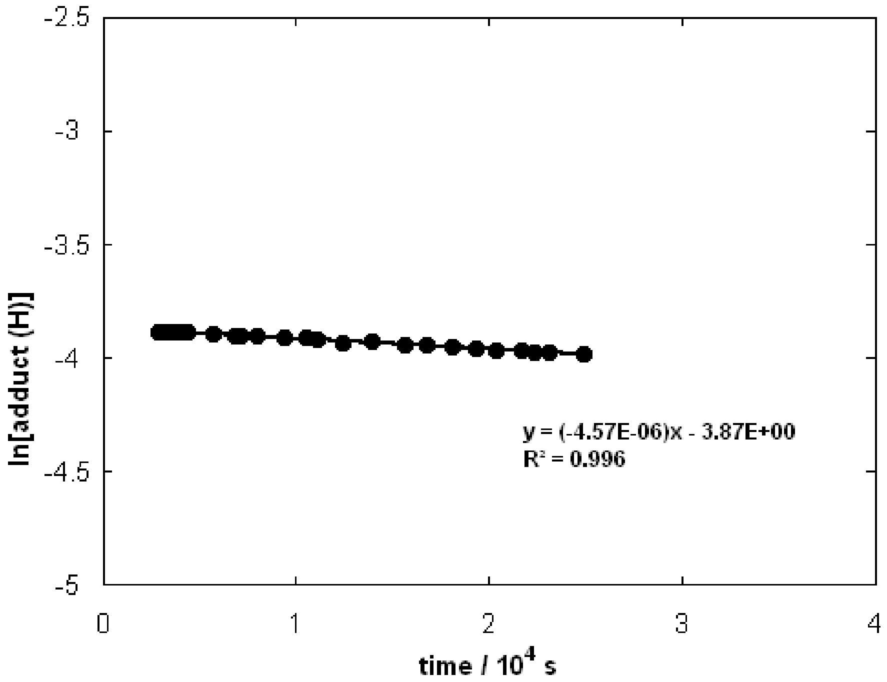

Figure S103. Plot of ln[H-adduct] against time used to determine k_2.

Determination of Rate and Equilibrium Constants for 3-(Hydroxybenzyl)azolium Adduct Formation in CD_3OD (Table S3)

Data was obtained in a similar manner to the experiments in Table 2 using the benzaldehyde **5** and the appropriate NHC precursor.

Table S3, Entry 1

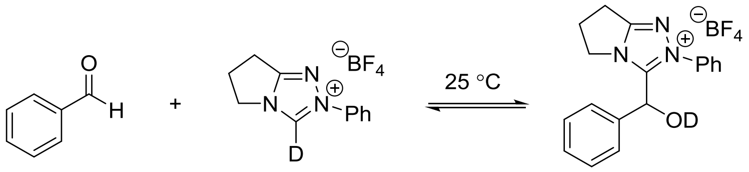

5 **9**

Data as for Table 4 and Table S4, Entry 1

Table S3, Entry 2

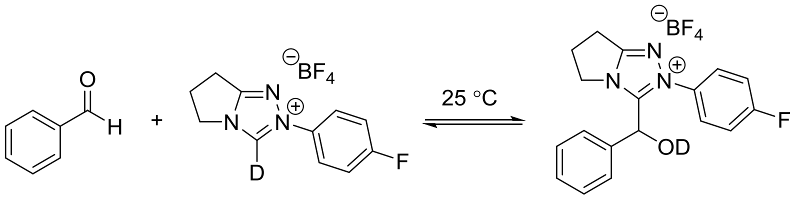

5 **34**

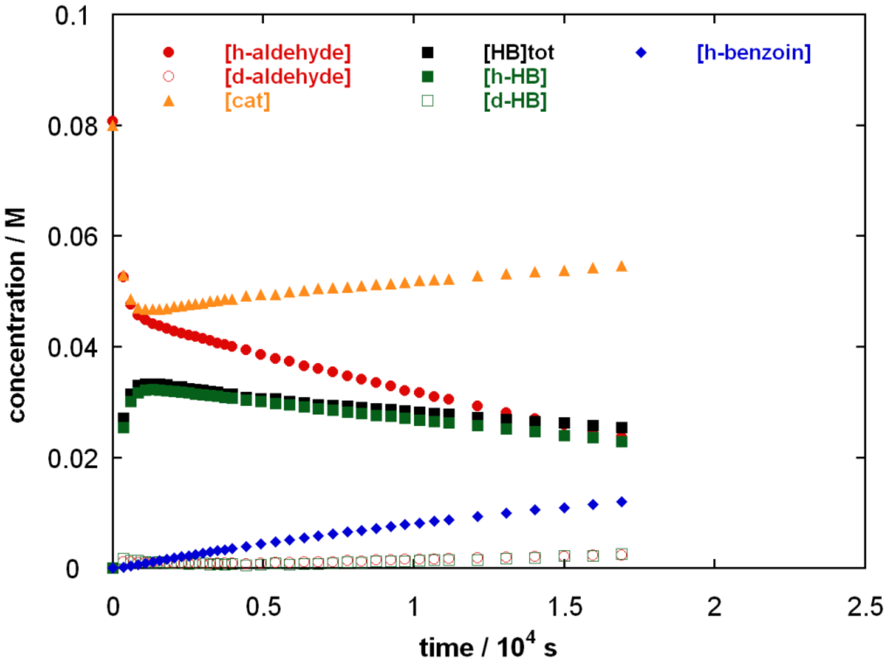

Figure S104. Reaction profile displaying concentration of species present against time for the reaction of benzaldehyde **5** with NHC precursor **34** in Et_3N:$Et_3N{\cdot}HCl$ and CD_3OD at 25 °C. [h-HB] = H-adduct; [d-HB] = D-adduct.

Table S3, Entry 3

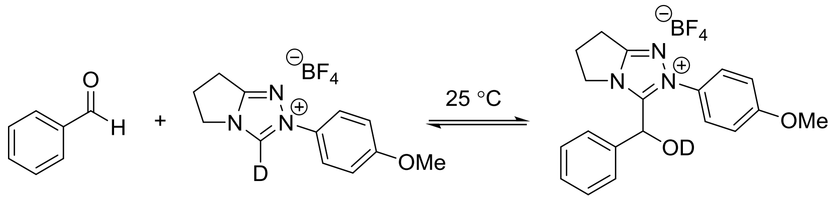

5 35

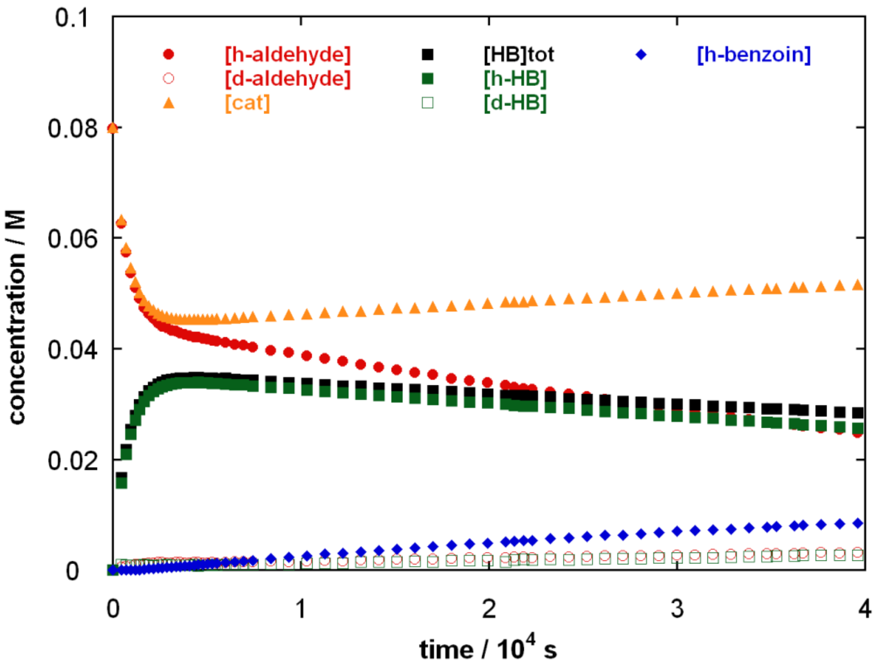

Figure S105. Reaction profile displaying concentration of species present against time for the reaction of benzaldehyde **5** with NHC precursor **35** in Et_3N:$Et_3N·HCl$ and CD_3OD at 25 °C. [h-HB] = H-adduct; [d-HB] = D-adduct.

Table S3, Entry 4

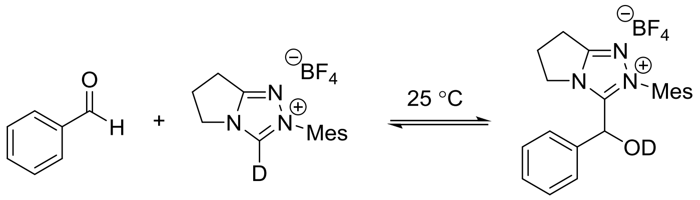

5 **10**

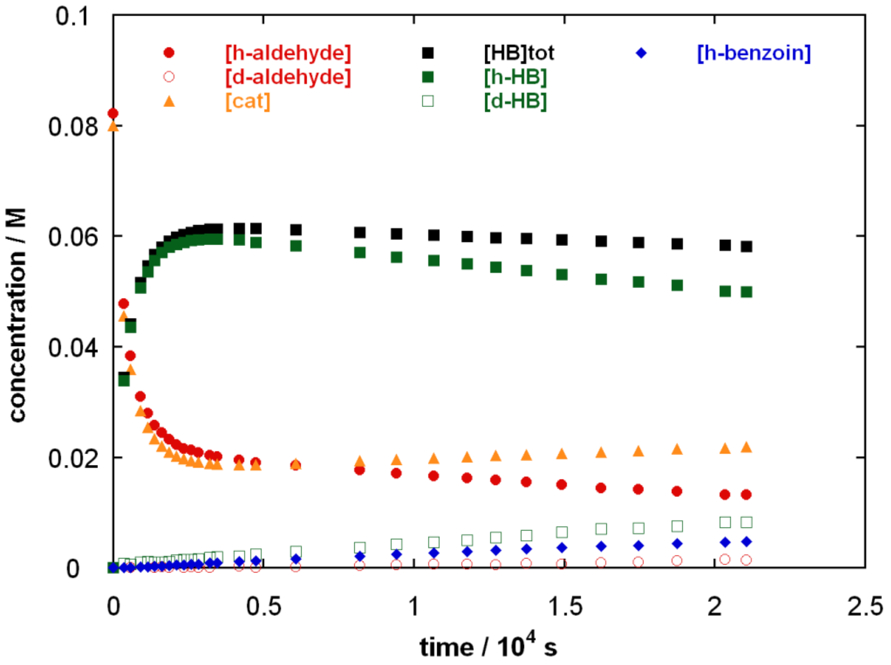

Figure S106. Reaction profile displaying concentration of species present against time for the reaction of benzaldehyde **5** with NHC precursor **10** in Et_3N:Et_3N·HCl and CD_3OD at 25 °C. [h-HB] = H-adduct; [d-HB] = D-adduct.

Determination of Rate and Equilibrium Constants for 3-(Hydroxybenzyl)azolium Adduct Dissociation in CD_3OD (Table S5)

Data was obtained in a similar manner to the experiments in Table 3 using the appropriate 3-(hydroxybenzyl)azolium adducts.

Table S5, Entry 1

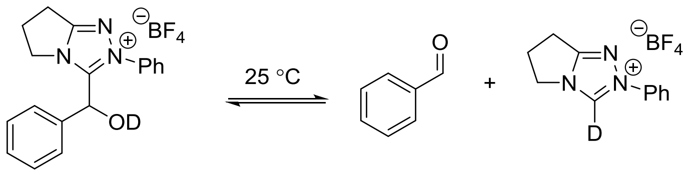

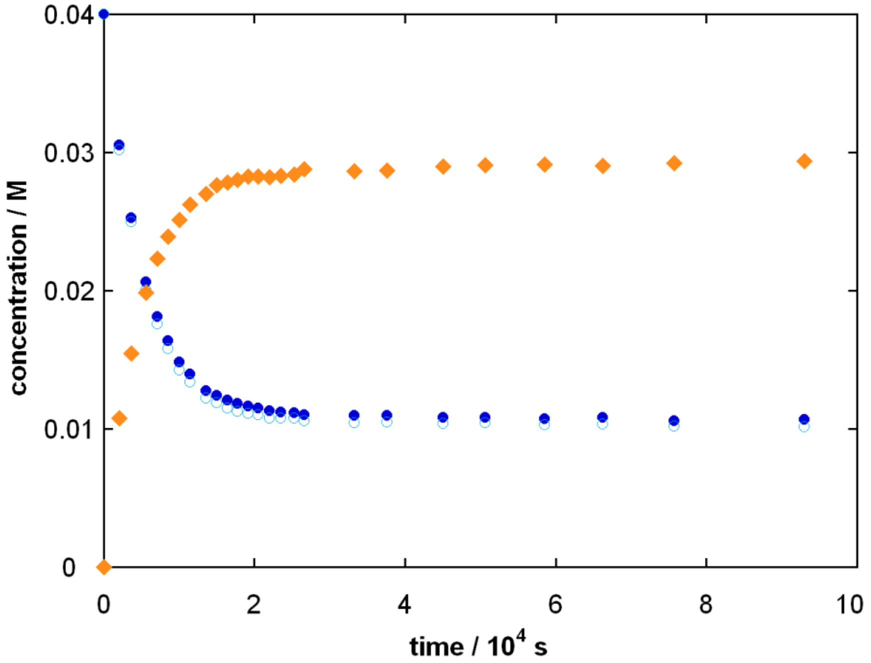

Figure S107. Reaction profile for dissociation of 3-(hydroxybenzyl)azolium adduct (blue) and appearance of NHC **9** (yellow) in Et_3N:$Et_3N\cdot HCl$ and CD_3OD at 25 °C.

Table S5, Entry 2

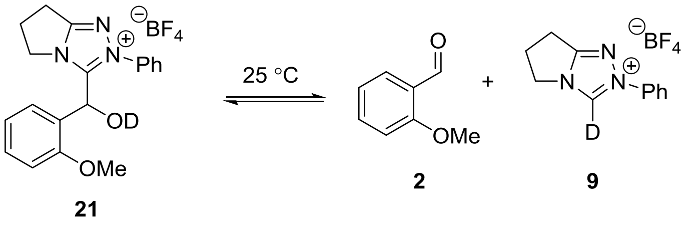

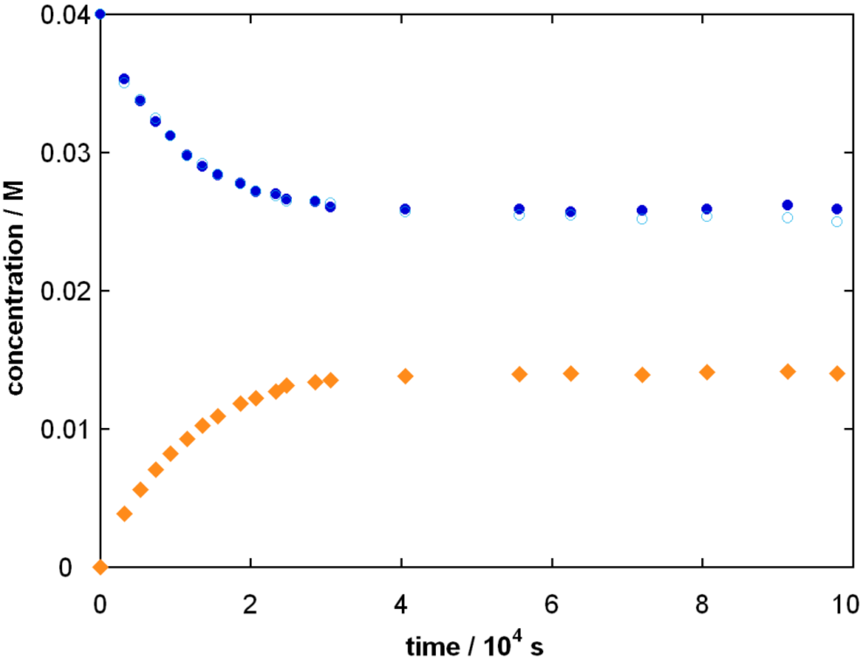

Figure S108. Reaction profile for dissociation of 3-(hydroxybenzyl)azolium adduct (blue) and appearance of NHC **9** (yellow) in Et_3N:Et_3N·HCl and CD_3OD at 25 °C.

Table S5, Entry 3

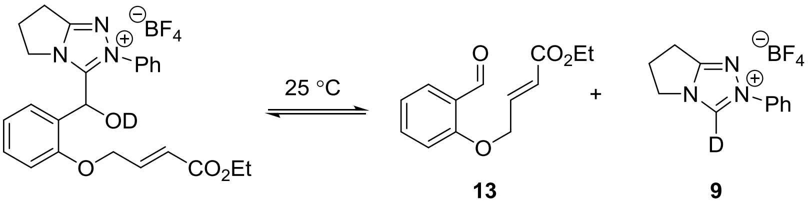

Data as for Table 3, Entry 1.

Table S5, Entry 4

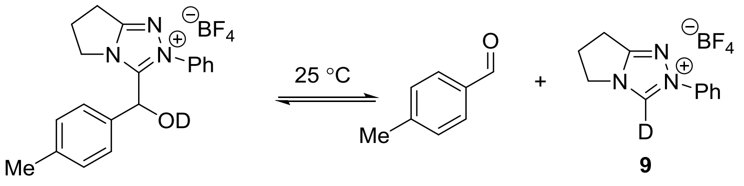

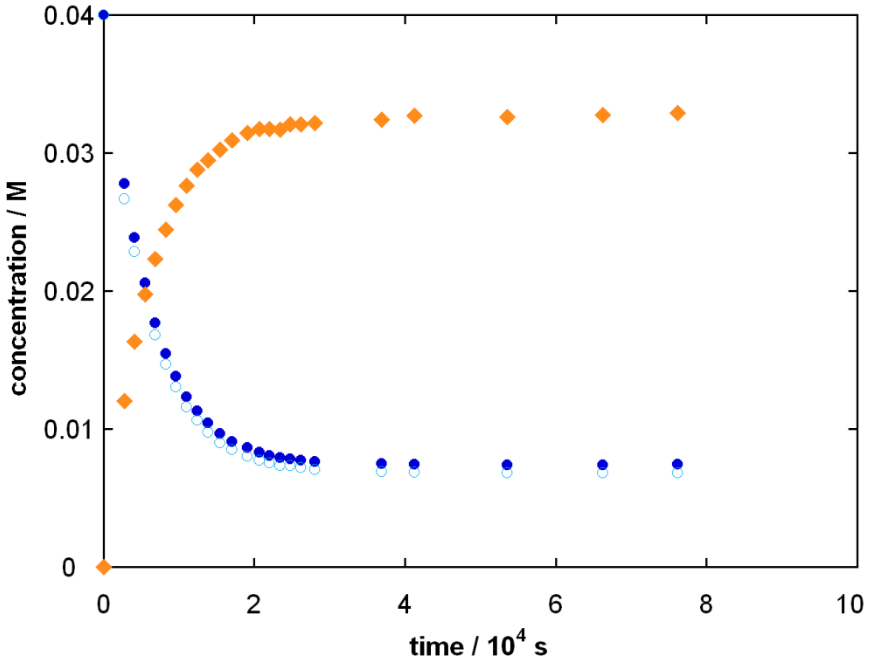

Figure S109. Reaction profile for dissociation of 3-(hydroxybenzyl)azolium adduct (blue) and appearance of NHC **9** (yellow) in Et_3N:Et_3N·HCl and CD_3OD at 25 °C.

Cross-Benzoin Reaction (Scheme 3a)

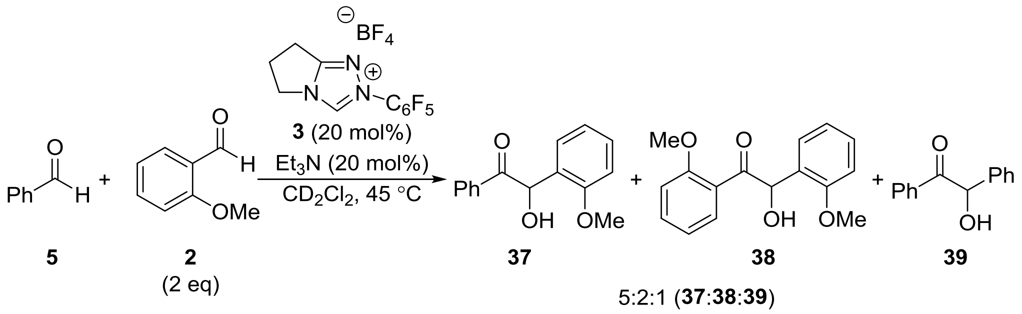

NHC precatalyst **3** (30 mg, 80 μmol), benzaldehyde **5** (41.0 μL, 0.400 mmol) 2-methoxybenzaldehyde **2** (109 mg, 0.800 mmol), Et_3N (11.0 μL, 80 μmol) and anhydrous CH_2Cl_2 (2 mL) were added to a flame-dried Schelenk under N_2. The reaction was heated at 45 °C for 16 h before being cooled to rt. The solution was diluted with CH_2Cl_2 (10 mL) and washed with 1 M HCl (2 × 10 mL) and brine (10 mL) before being dried ($MgSO_4$) and concentrated in vacuo. The crude product was purified by Biotage® Isolera™ 4 [SNAP KP-Sil 10 g, 36 mLmin^{-}1 , hexane:EtOAc (100:0 1CV, 100:0 to 80:20 15 CV, 80:20 10 CV)] to give:

Cross-benzoin **37** (38 mg, 40%) as a white solid with data in accordance with the literature.[6] mp 54–56 °C (hexane:Et_2O) {Lit.[7] 57–59 °C}; ^{1}H NMR (400 MHz, CD_2Cl_2) δ_H: 3.86 (3H, s, OC*H*$_3$), 4.40 (1H, d, *J* 5.9, O*H*), 6.24 (1H, d, *J* 5.9, C*H*OH), 6.88-6.92 (2H, m, C(2)ArC(3,4)*H*), 7.15 (1H, dd, *J* 7.9, 1.8, C(2)ArC(5)*H*), 7.25-7.29 (1H, m, C(2)ArC(6)*H*), 7.36-7.40 (2H, m, C(1)ArC(3,5)*H*), 7.49-7.53 (1H, m, C(1)ArC(4)*H*), 7.91-7.94 (2H, m, C(1)ArC(2,6)*H*).

Homo-benzoin **38** (29 mg, 26%) as a white solid with data in accordance with the literature.[8] mp 97–98 °C {Lit.[8] 101-103}; ^{1}H NMR (400 MHz, CD_2Cl_2) δ_H: 3.70 (3H, s, OC*H*$_3$), 3.74 (3H, s, OC*H*$_3$), 4.29 (1H, d, *J* 5.8, O*H*), 6.05 (1H, d, *J* 5.8, C*H*OH), 6.77-6.86 (3H, m, Ar*H*), 6.93 (1H, t, *J* 7.5, ArC*H*), 7.13-7.23 (2H, m, Ar*H*), 7.39 (1H, t, *J* 7.8 Ar*H*), 7.63 (1H, dd, *J* 7.8, 1.8, Ar*H*).

Cross-Benzoin Reaction using (1-D)-2-Methoxybenzaldehyde

An alternative mechanism in which Breslow intermediate **41** reacts with benzaldehyde **5** to form an adduct (analogous to **42**/**43**) that undergoes a 1,2-hydride shift to eliminate the NHC would also lead to major product **37**. This possibility has been ruled out based upon a cross-benzoin reaction using (1-D)-2-methoxybenzaldehyde ***d*-2** (Scheme S4). Major cross-product ***d*-37** was isolated with *ca.* 80% D-incorporation, suggesting that the product is derived from onwards reaction of minor adduct **24** through Breslow intermediate **40**. The slight loss in deuterium content is accounted for by protonation of Breslow intermediate **41** as all steps are known to be reversible.

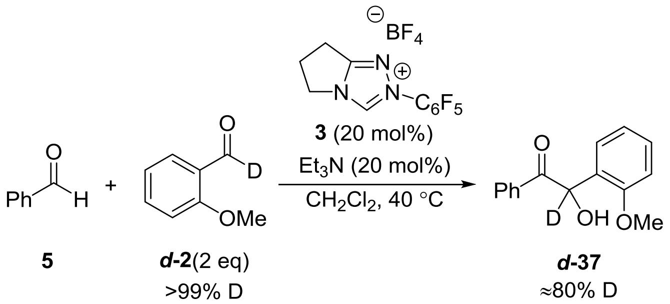

Scheme S4. Cross-benzoin using (1-D)-2-methoxybenzaldehyde ***d*-2**.

Deuterium incorporation was determined by ^{1}H NMR spectroscopy by comparison of the integral for the C(1)ArC(2,6)*H* (δ_H = 7.93-7.98) signal with the integral for any C*H*OH present (δ_H = 6.28).

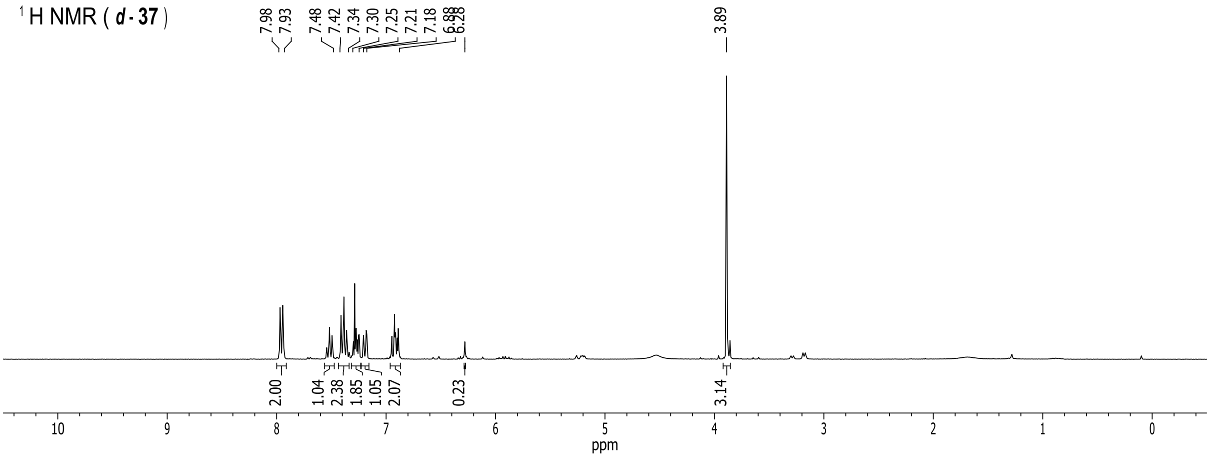

Figure S110. ^{1}H NMR spectra of ***d*-37** (*ca.* 80% D) from reaction of ***d*-2** (>99% D) with benzaldehyde **5** using NHC precatalyst **3**.

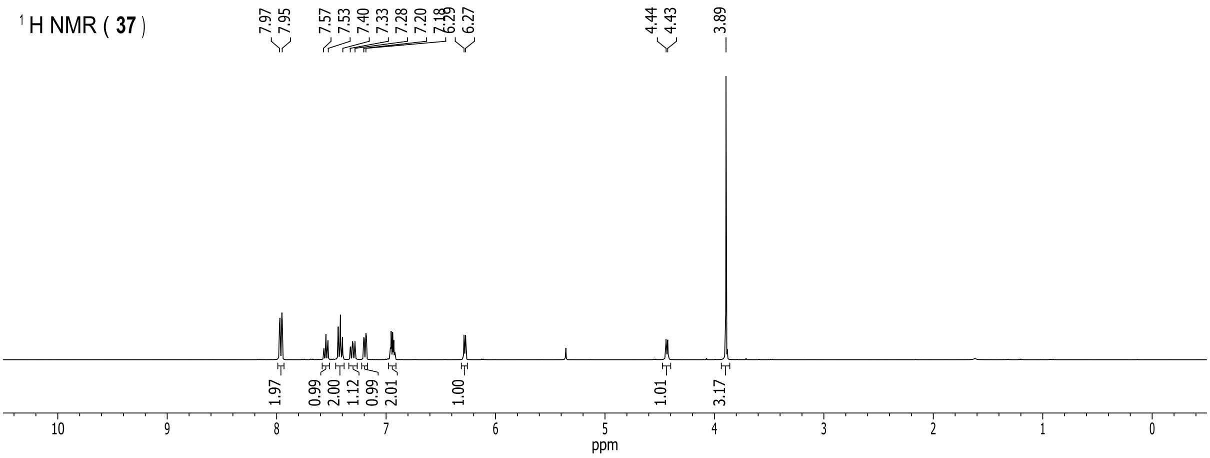

Figure S111. ^{1}H NMR spectra of **37**.

Competition Experiment (Scheme 3b)

Individual Components

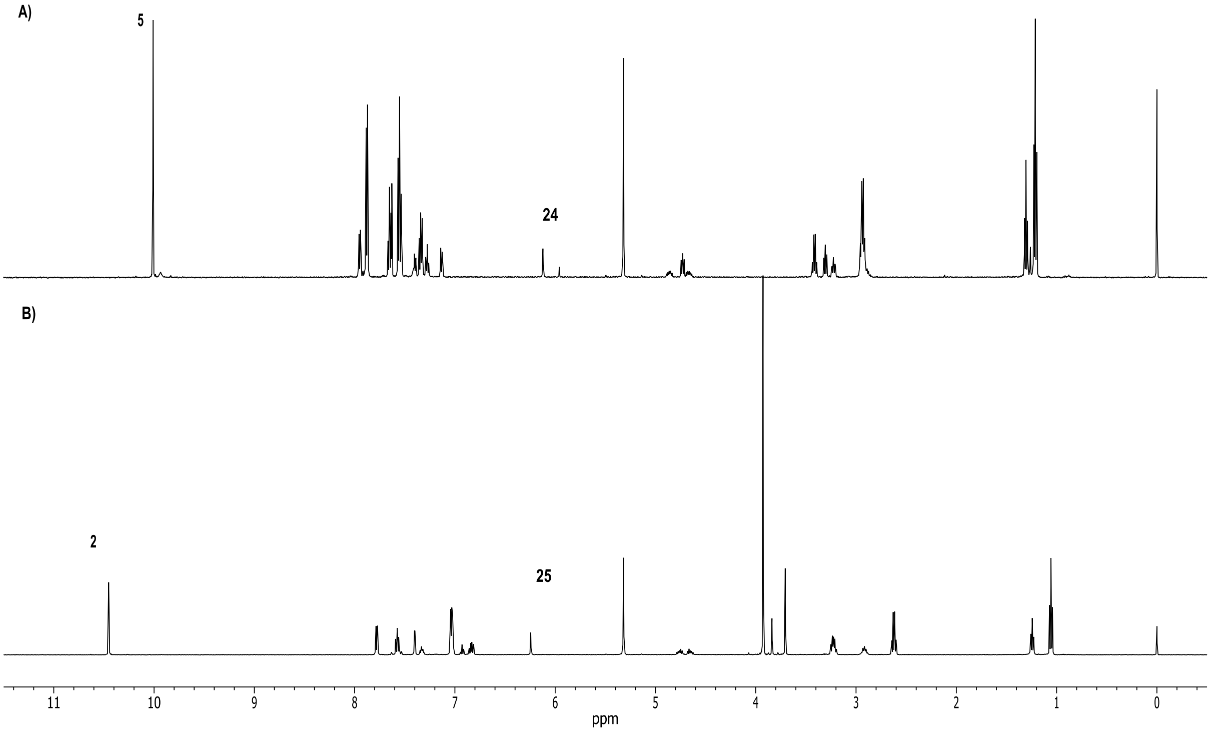

Figure S112. ^{1}H NMR spectra for reaction of: A) Benzaldehyde **5** (0.01 M) with NHC precursor **11** (0.002 M) and Et_3N (0.002 M) in CD_2Cl_2 at 25 °C; B) 2-Methoxybenzaldehyde **2** (0.01 M) with NHC precursor **11** (0.002 M) and Et_3N (0.002 M) in CD_2Cl_2 at 25 °C.

Competition Experiment

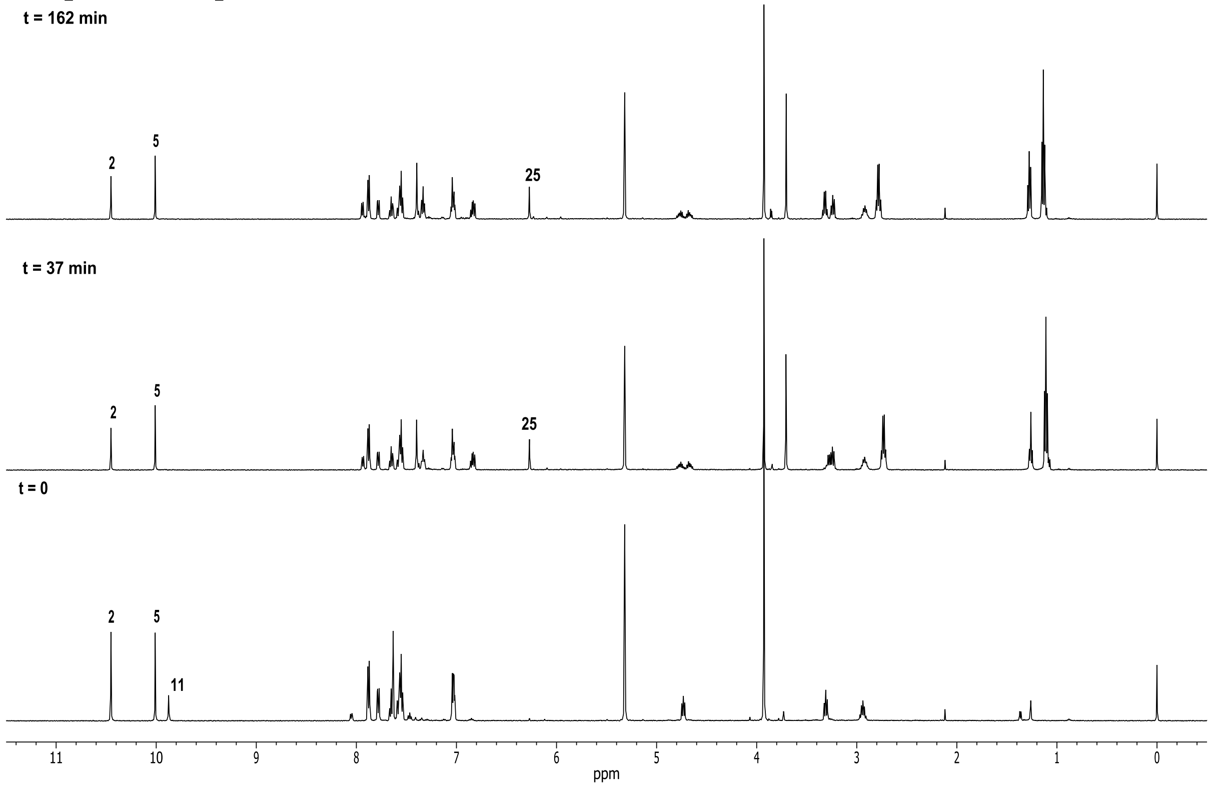

Figure S113. Representative ^{1}H NMR spectra (500 MHz) for reaction of 2-methoxybenzaldehyde **2** (0.02 M) and benzaldehyde **5** (0.02 M) with NHC precursor **11** (0.008 M) and Et_3N (0.008 M) in CD_2Cl_2 at 25 °C.

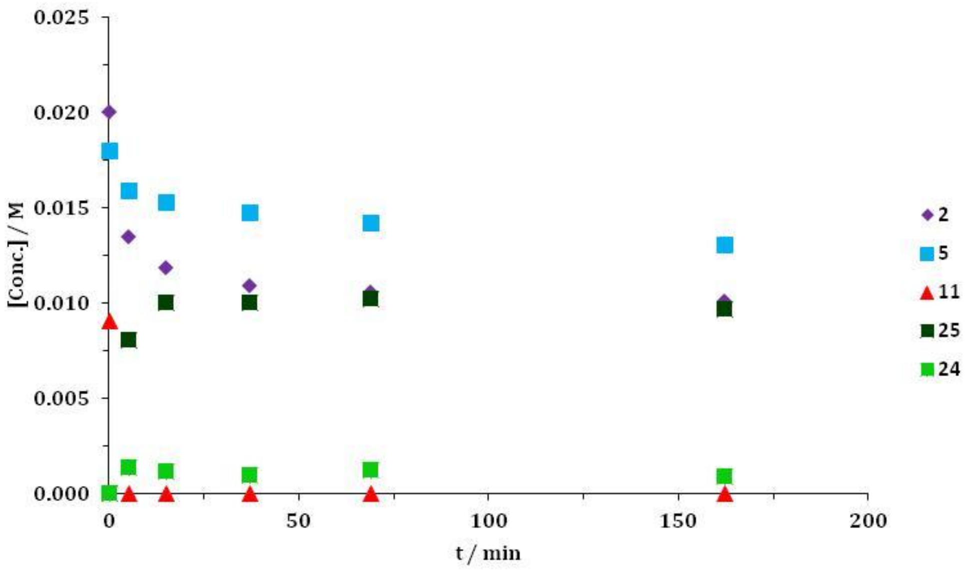

Figure S114. Reaction profile displaying concentration of species present against time for reaction of 2-methoxybenzaldehyde **2** (0.02 M) and benzaldehyde **5** (0.02 M) with NHC precursor **11** (0.008 M) and Et_3N (0.008 M) in CD_2Cl_2 at 25 °C.

Cross-Benzoin 37 Retreatment Experiment

Retreating cross-benzoin **37** with NHC precatalyst **11** gave a small amount of *retro*-benzoin (*ca*. 10%), with no peaks corresponding to NHC-ketone adduct **42** observed.

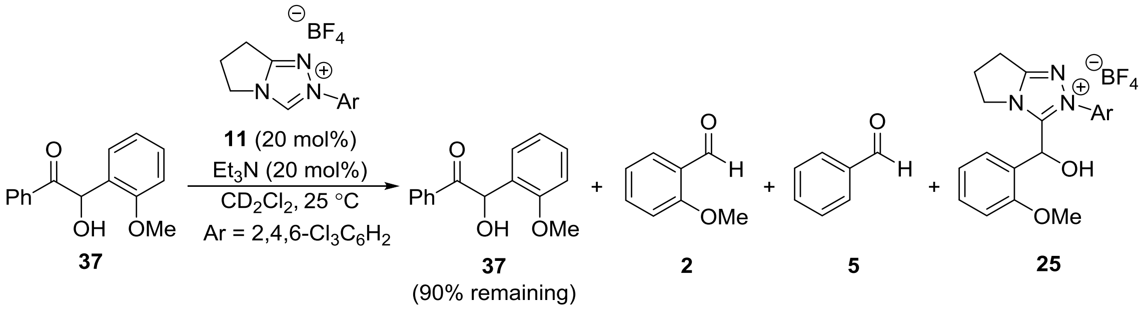

Scheme S5. Retreatment of cross-benzoin **37** with NHC precatalyst **11**

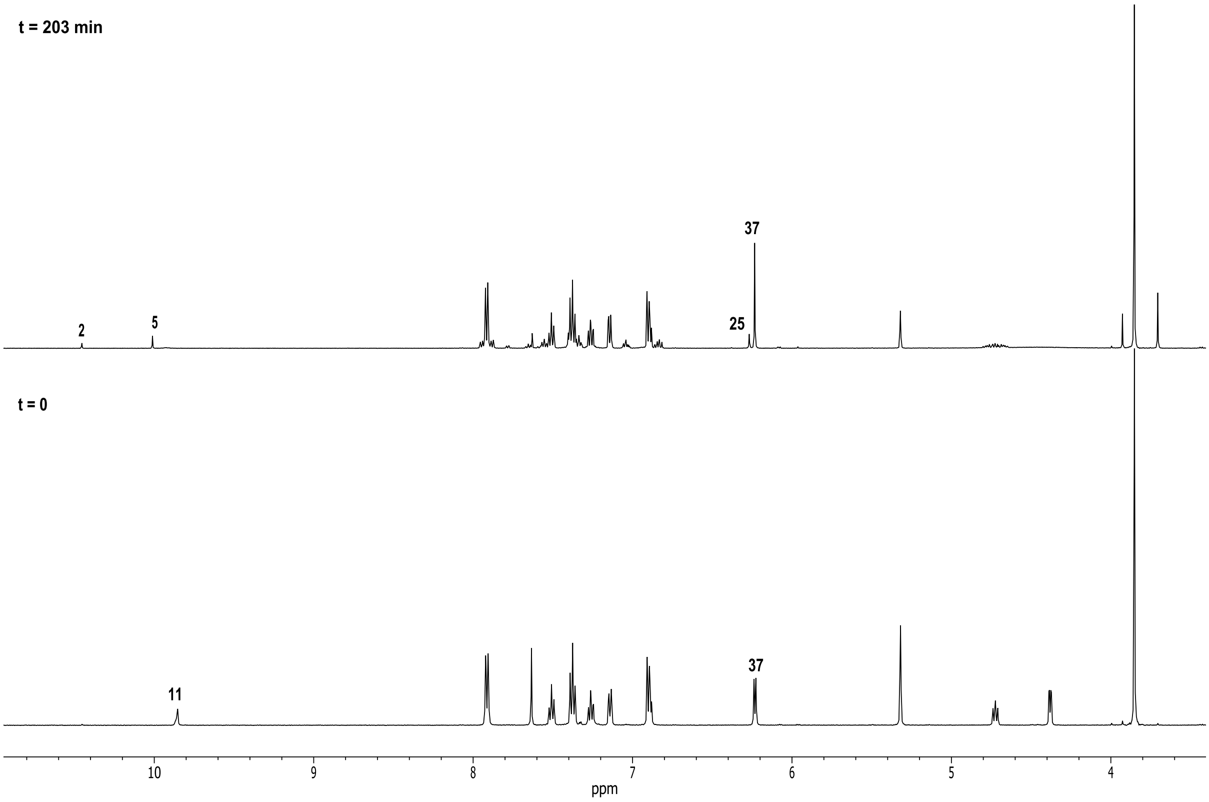

Figure S115. Representative ^{1}H NMR spectra (500 MHz) for reaction of cross-benzoin **37** (0.04 M) with NHC precursor **11** (0.008 M) and Et_3N (0.008 M) in CD_2Cl_2 at 25 °C.

Homo-Benzoin 38 Retreatment Experiment

Retreating homo-benzoin **38** with NHC precatalyst **11** gave a small amount of *retro*-benzoin (*ca*. 12%), with no peaks corresponding to NHC-ketone adduct **43** observed.

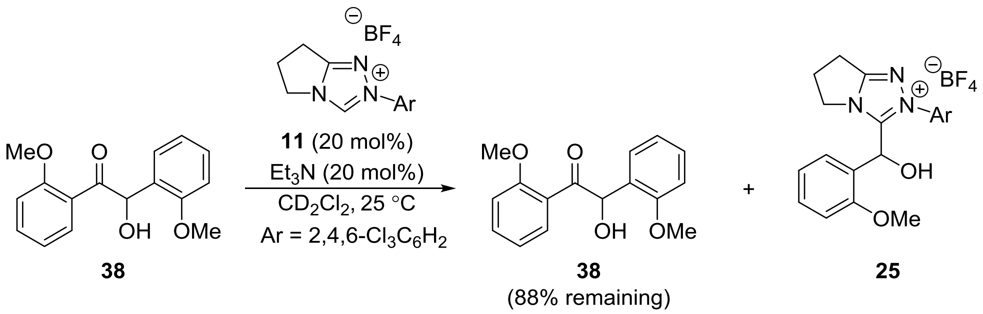

Scheme S6. Retreatment of homo-benzoin **38** with NHC precatalyst **11**

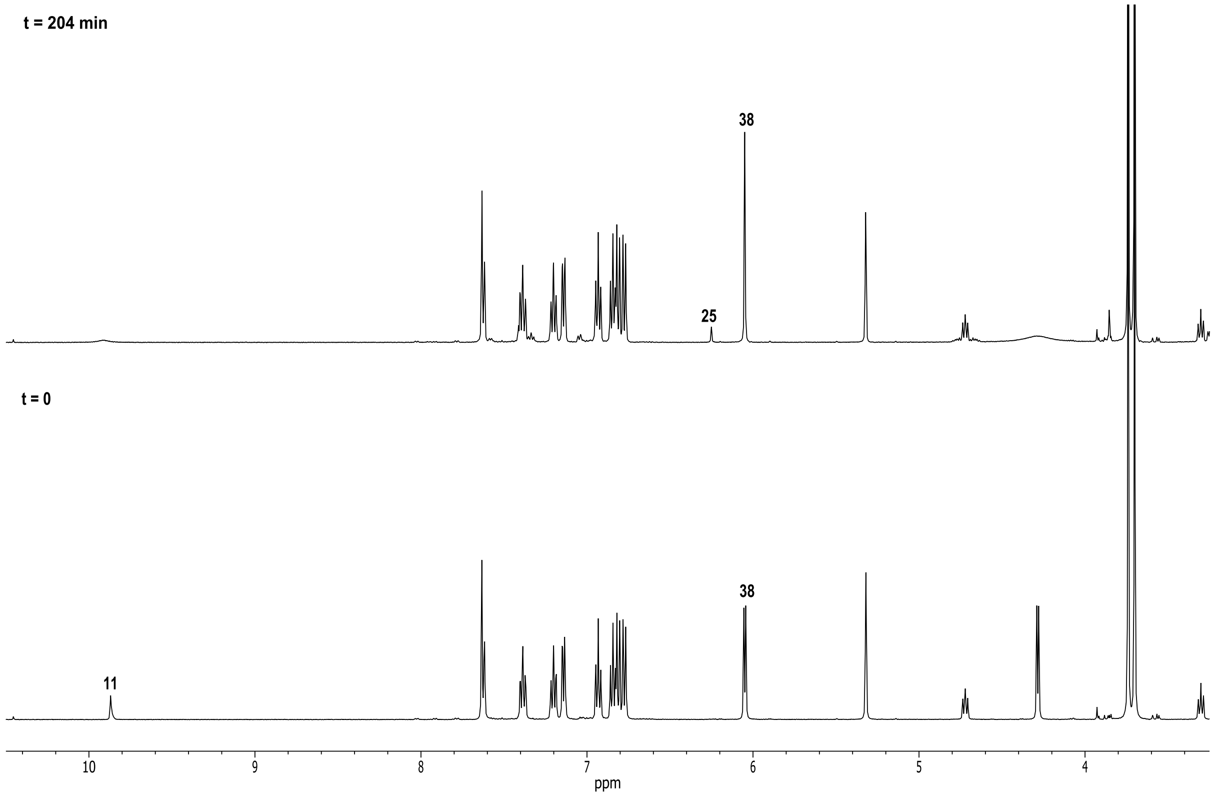

Figure S116. Representative ^{1}H NMR spectra (500 MHz) for reaction of homo-benzoin **38** (0.04 M) with NHC precursor **11** (0.008 M) and Et_3N (0.008 M) in CD_2Cl_2 at 25 °C.

Acetophenone Control Experiment

A control experiment treating acetophenone with NHC precatalyst **11** gave no observable products, showing that NHC-ketone adducts are not readily formed.

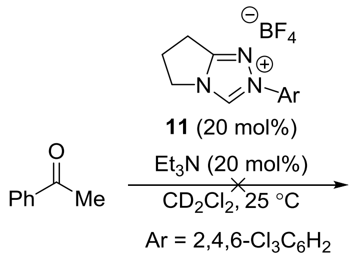

Scheme S7. Control experiment treating acetophenone with NHC precatalyst **11**

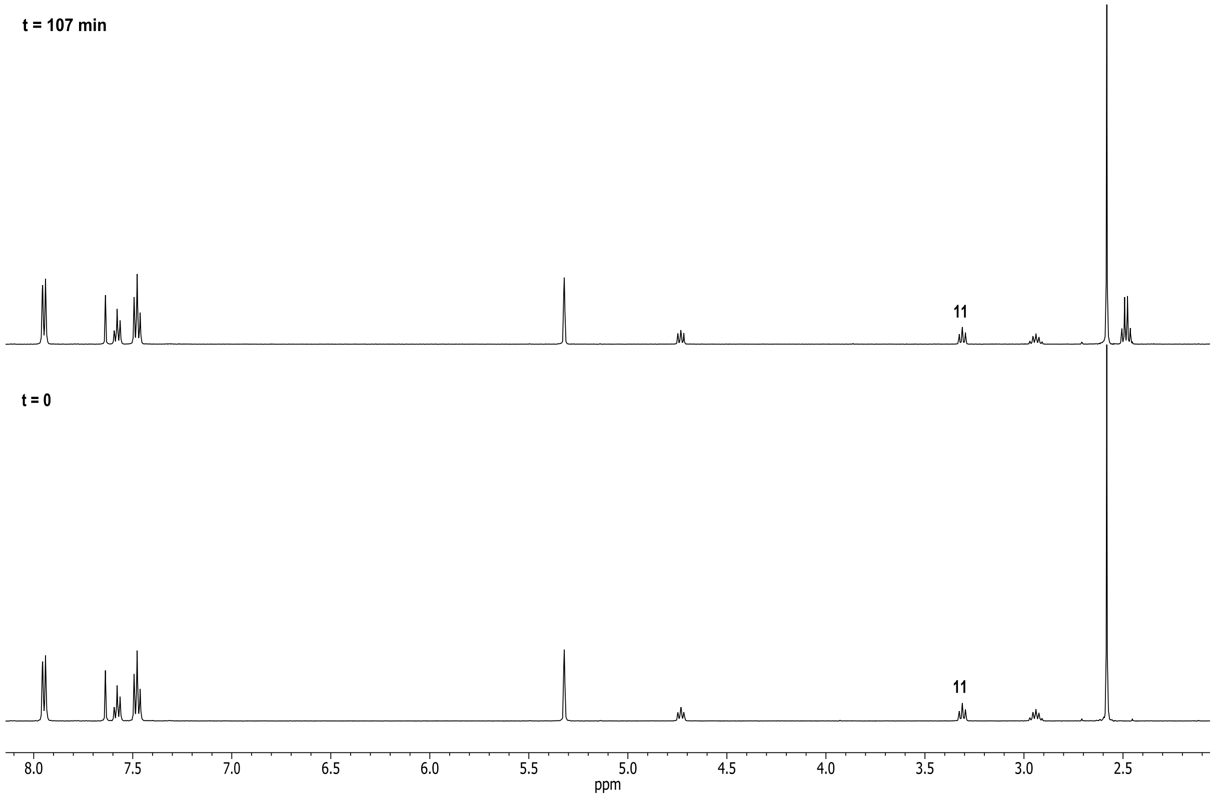

Figure S117. Representative ^{1}H NMR spectra (500 MHz) for treatment of acetophenone (0.04 M) with NHC precursor **11** (0.008 M) and Et_3N (0.008 M) in CD_2Cl_2 at 25 °C.

References

[1] T. Shimizu, Y. Hayashi, Y. Kitora, K. Teramura, *Bull. Chem. Soc. Jpn*. **1982**, *55*, 2450-2455.

[2] F. Sieber, P. Wentworth Jr., J. D. Toker, A. D. Wentworth, W. A. Metz, N. N. Reed, K. D. Janda, *J. Org. Chem*. **1999**, *64*, 5188-5192.

[3] S. B. Boga, A. B. Alhassan, D. Hesk, *Tetrahedron Lett.* **2014**, *55*, 4442-4444.

[4] C. J. Collett, R. S. Massey, O. R. Maguire, A. S. Batsanov, A. C. O'Donoghue, A. D. Smith, *Chem. Sci.* **2013**, *4*, 1514-1522.

[5] *Comprehensive Chemical Kineticsl, Vol. 2* (Eds.: C. H. Bamford, C. F. H. Tipper), Elsevier Scientific, Amsterdam, **1969**.

[6] I. Piel, M. D. Pawelczyk, K. Hirano, R. Froehlich, F. Glorius, *Eur. J. Org. Chem*. **2011**, 5475-5484

[7] R. E. Koenigkramer, H. Zimmer, *J. Org. Chem.* **1980**, *45*, 3994-3998

[8] T. J. Donohoe, A. Jahanshahi, M. J. Tucker, F. L. Bhatti, I. A. Roslan, M. Kabeshov, G. Wrigley, *Chem. Commun*. **2011**, *47*, 5849-5851

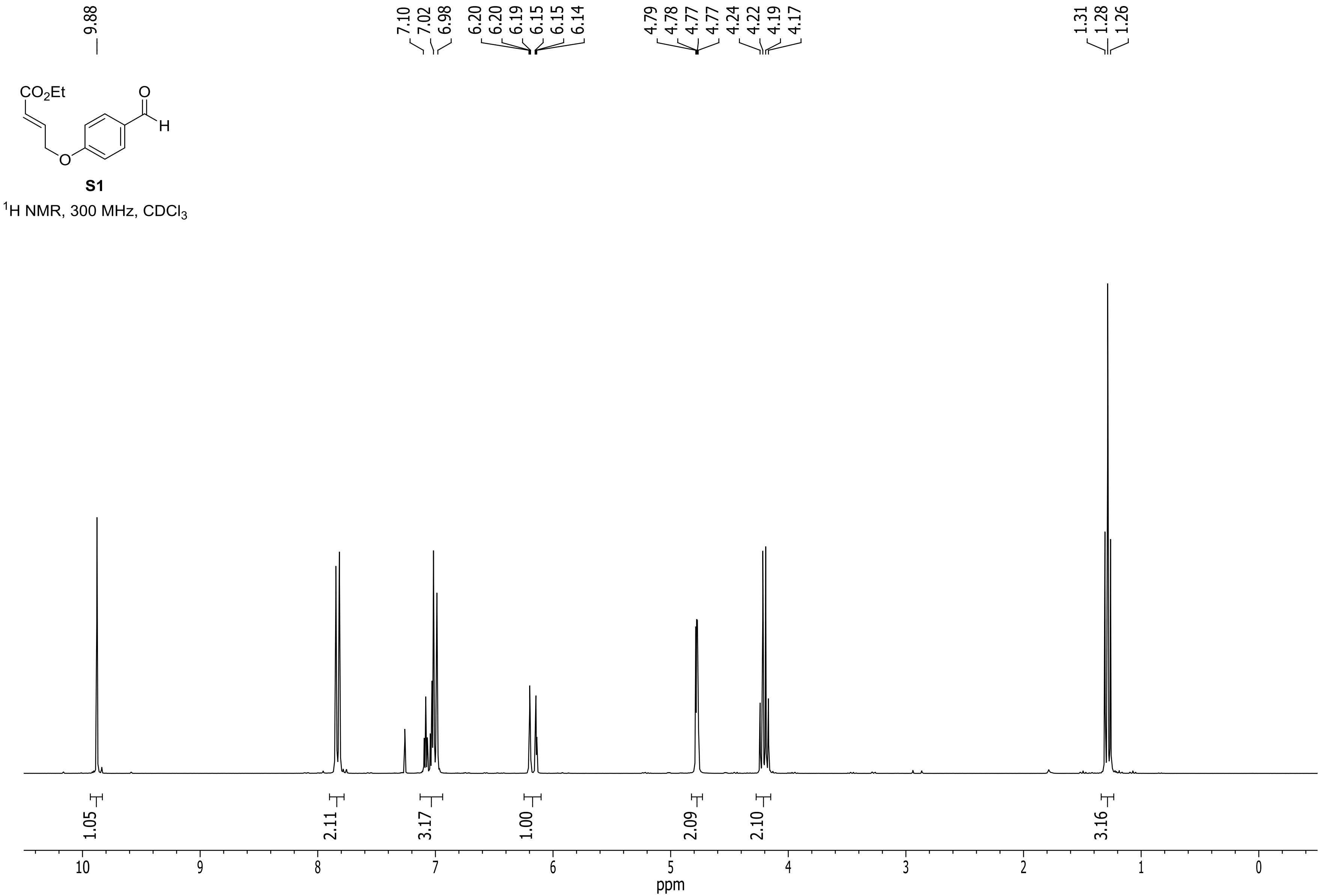
S1
1H NMR, 300 MHz, CDCl3
9.88
7.10
7.02
6.98
6.20
6.20
6.19
6.15
6.15
6.14
4.79
4.78
4.77
4.77
4.24
4.22
4.19
4.17
1.31
1.28
1.26
1.05
2.11
3.17
1.00
2.09
2.10
3.16
10
9
8
7
6
5
4
3
2
1
0
ppm

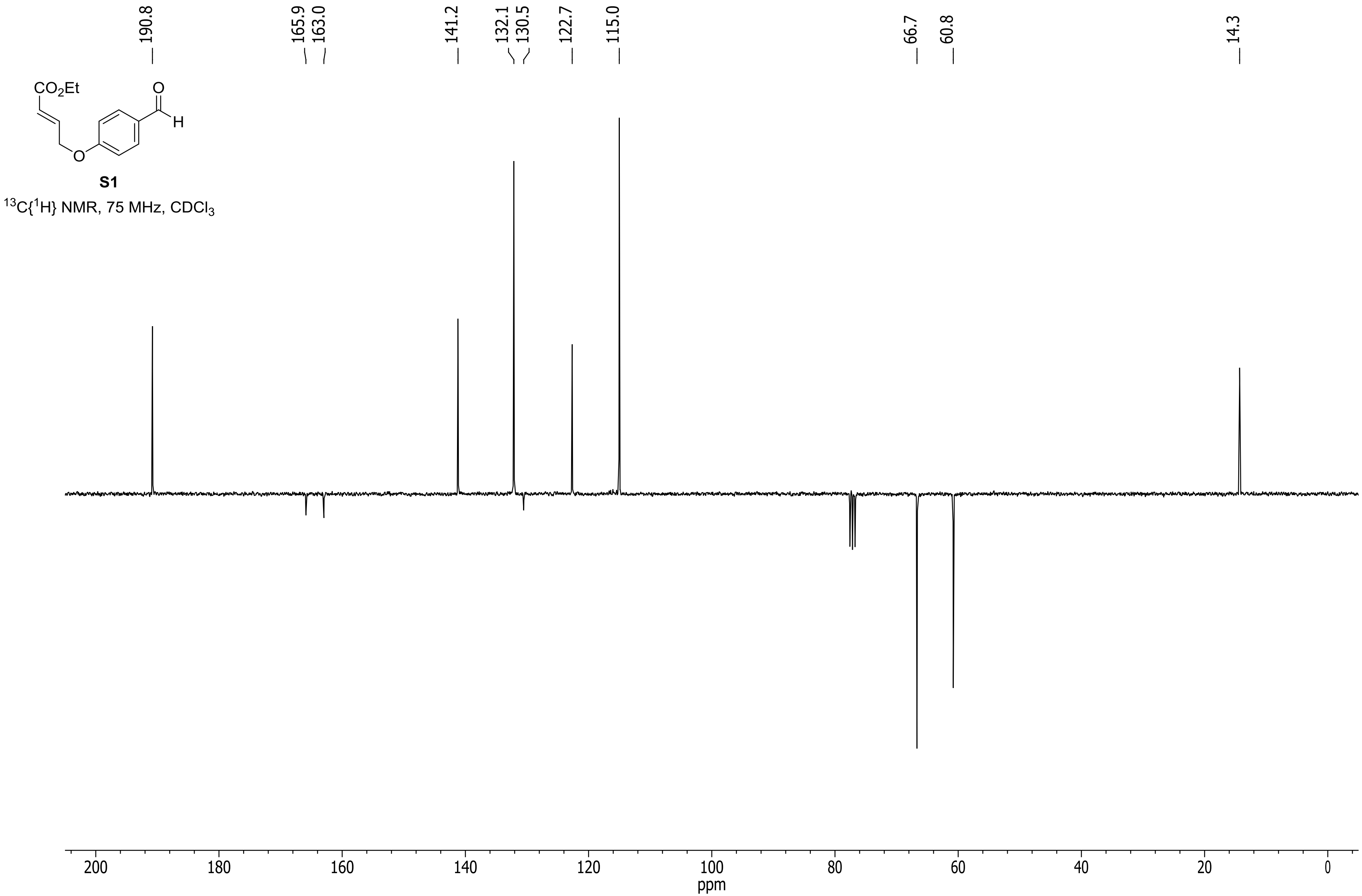
190.8
165.9
163.0
141.2
132.1
130.5
122.7
115.0
66.7
60.8
14.3
CO2Et
O
H
O
S1
13C{1H} NMR, 75 MHz, CDCl3
200
180
160
140
120
100
80
60
40
20
0
ppm

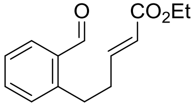

S3

$^{13}C\{^{1}H\}$ NMR, 75 MHz, CD_2Cl_2

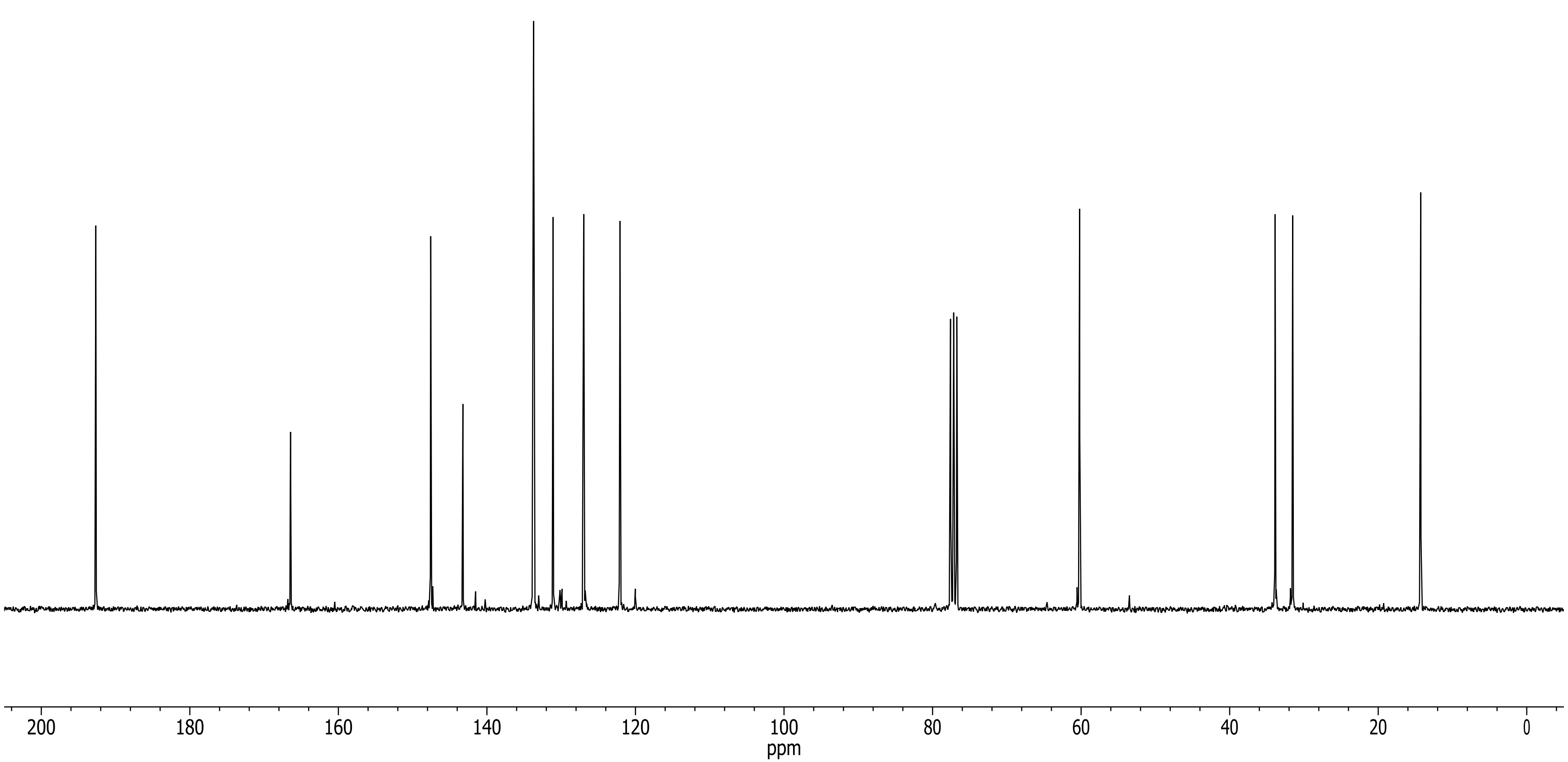

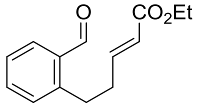

S3

$^{13}C\{^1H\}$ NMR, 75 MHz, CD_2Cl_2

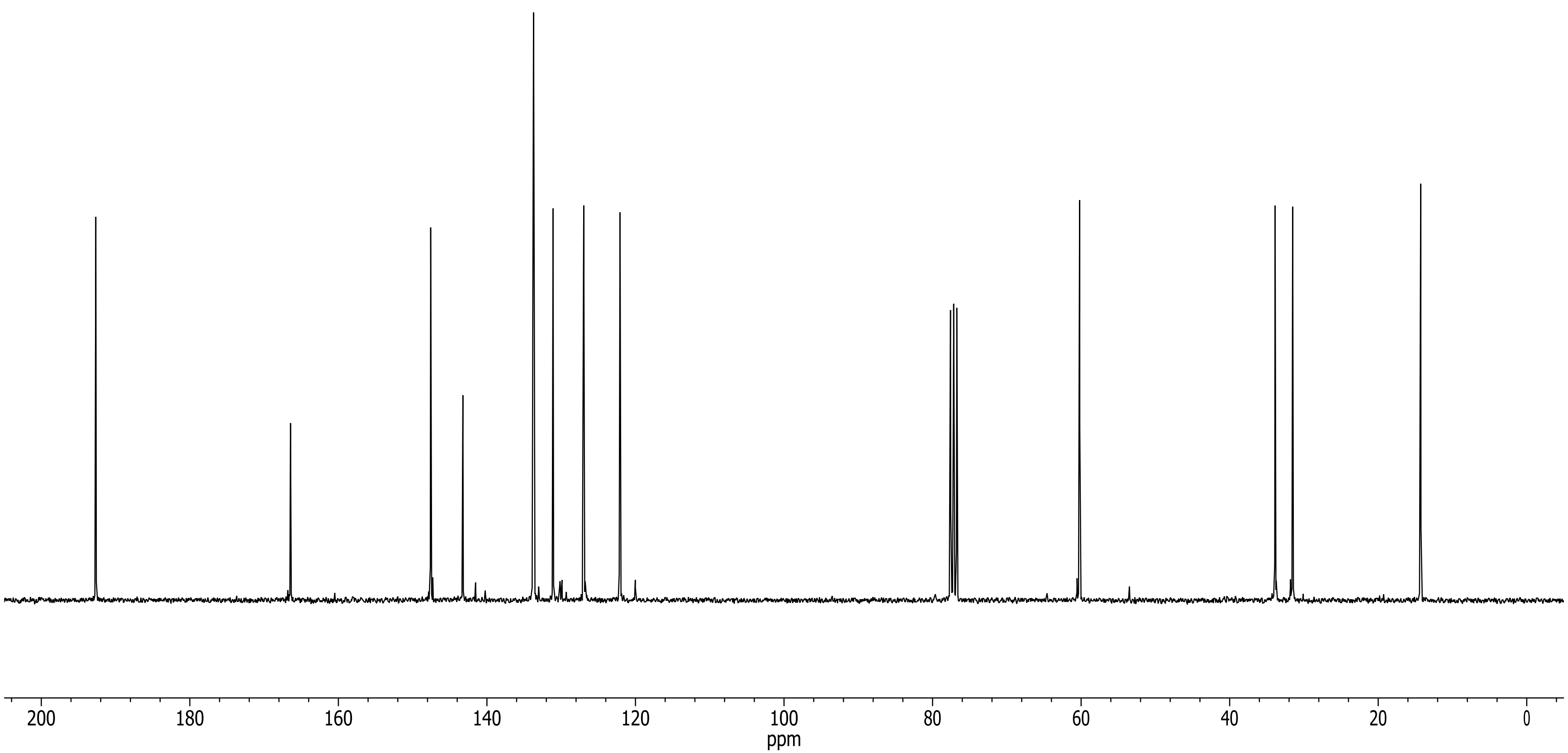

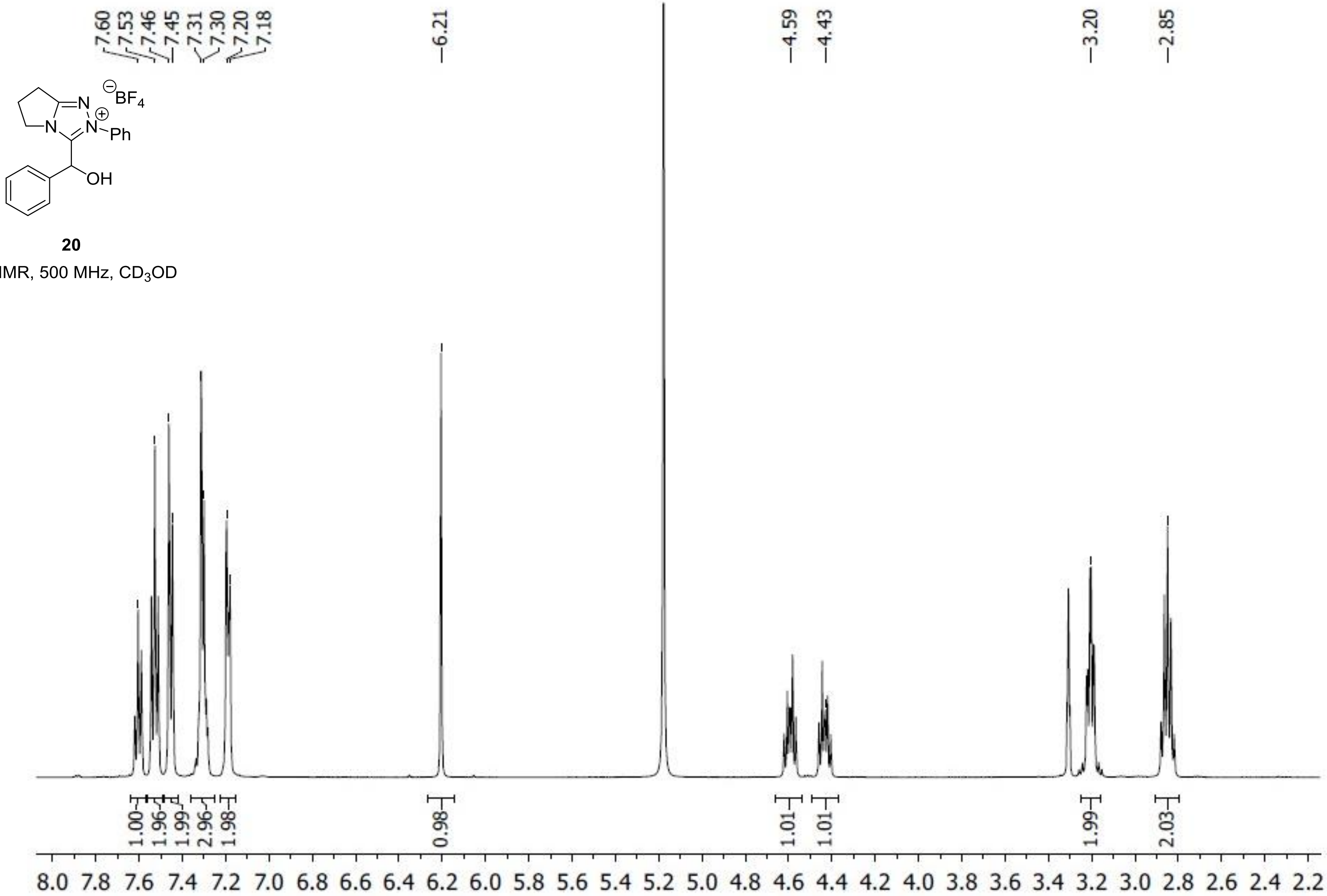

20

^{1}H NMR, 500 MHz, CD_3OD

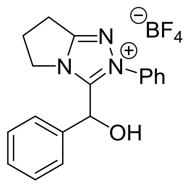

20

$^{13}C\{^{1}H\}$ NMR, 125 MHz, CD_3OD

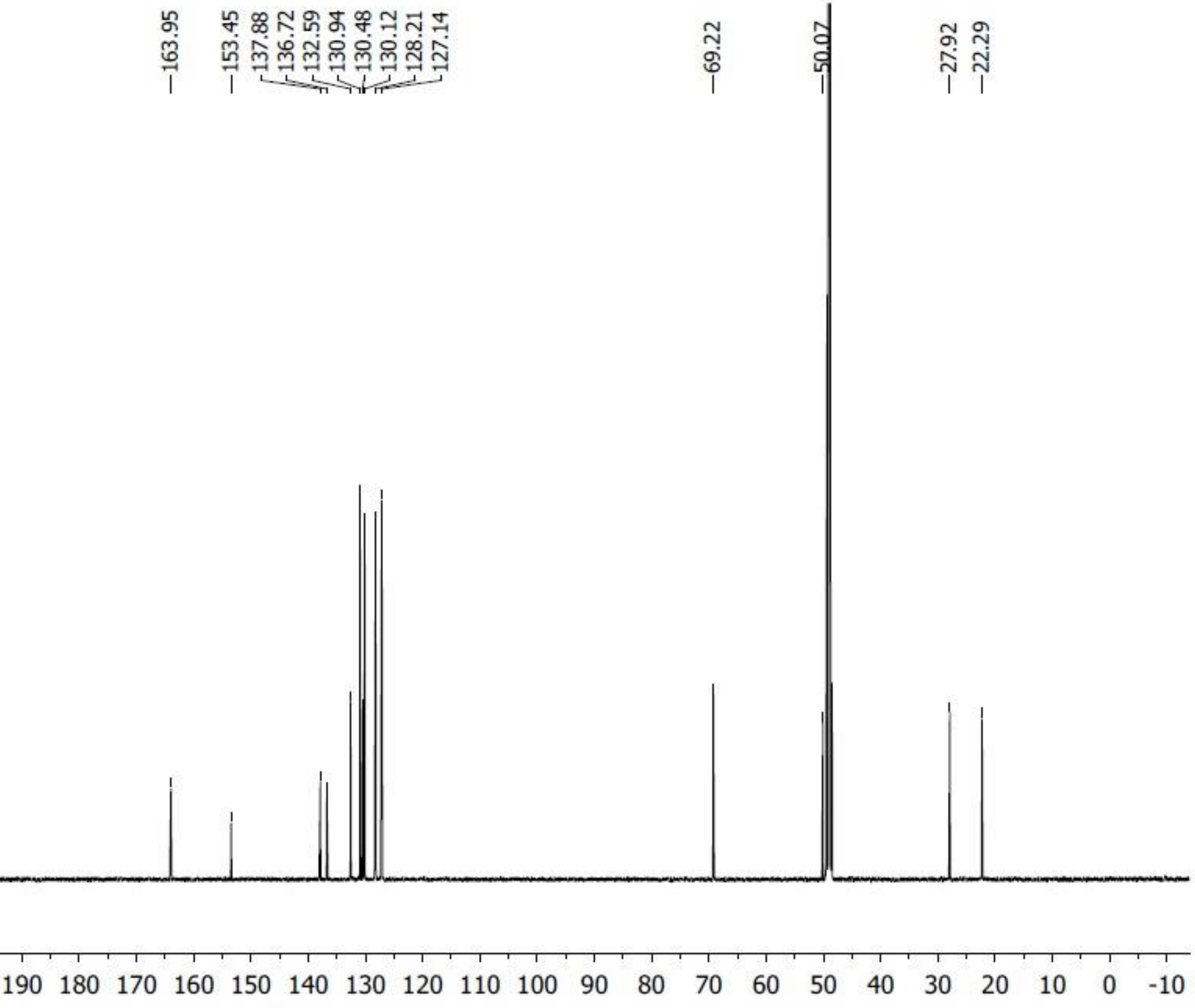

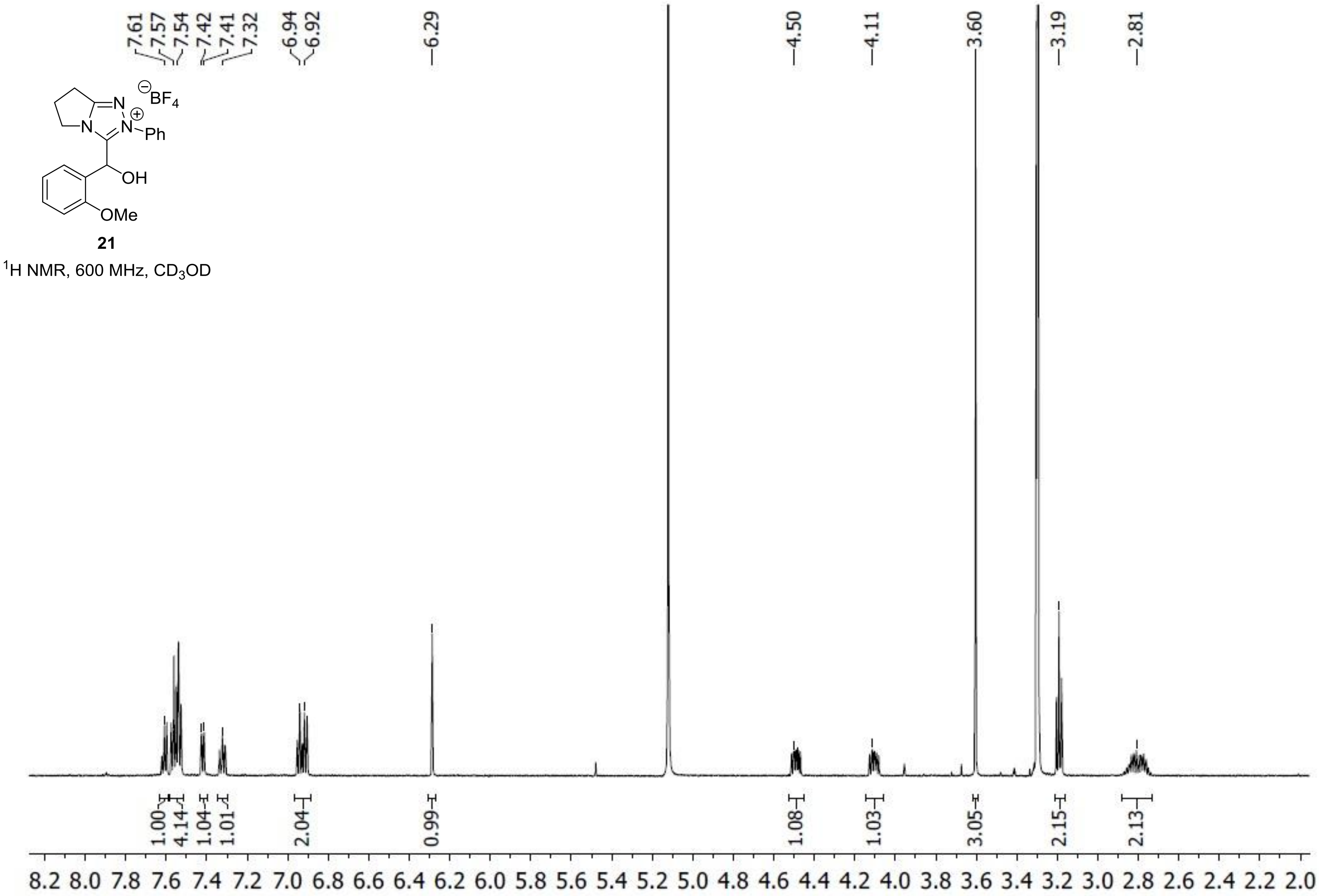

21

^{1}H NMR, 600 MHz, CD_3OD

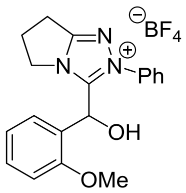

21

$^{13}C\{^{1}H\}$ NMR, 151 MHz, CD_3OD

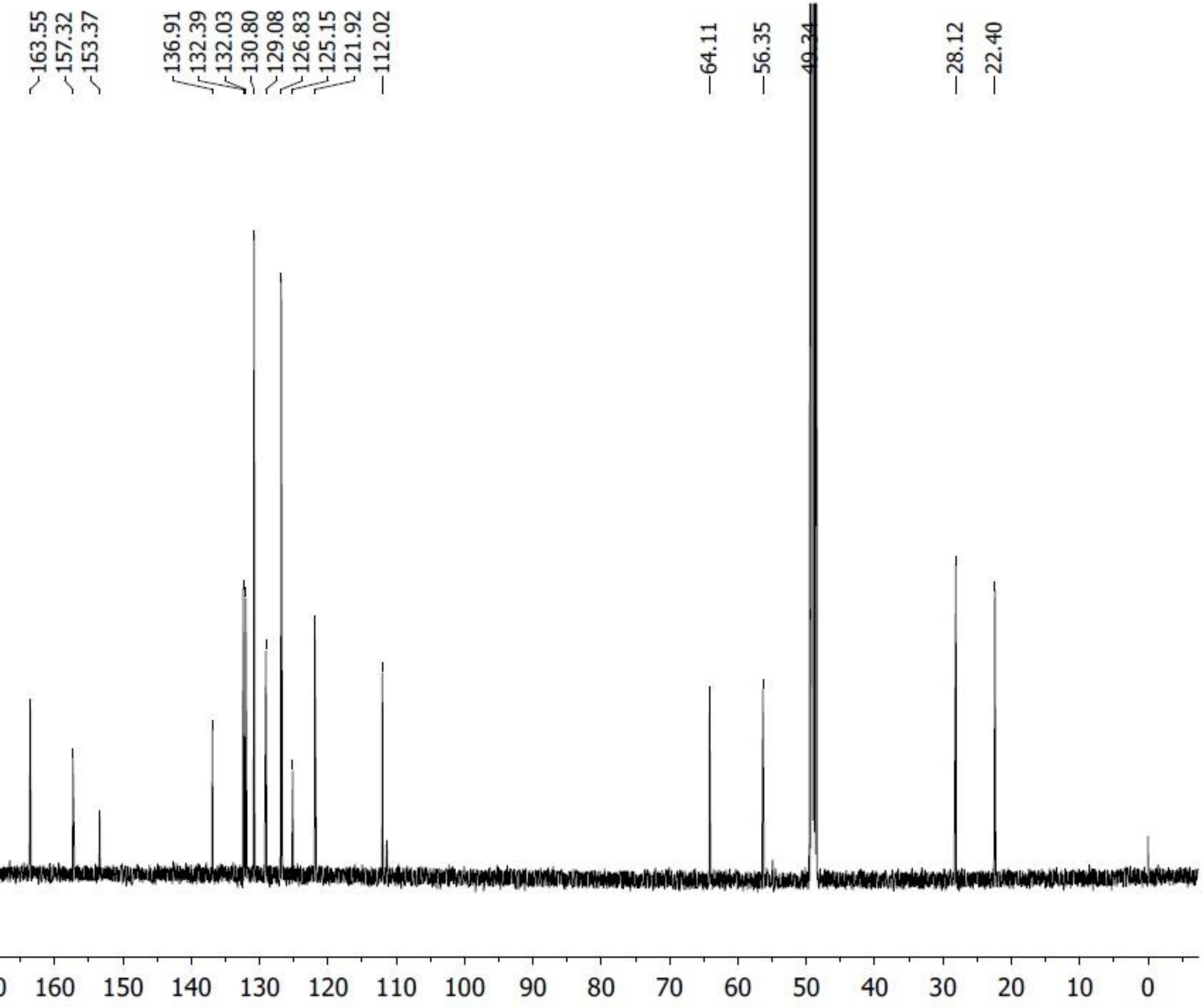

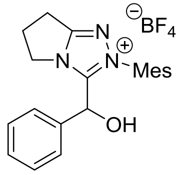

22

^{1}H NMR, 500 MHz, CD_3OD

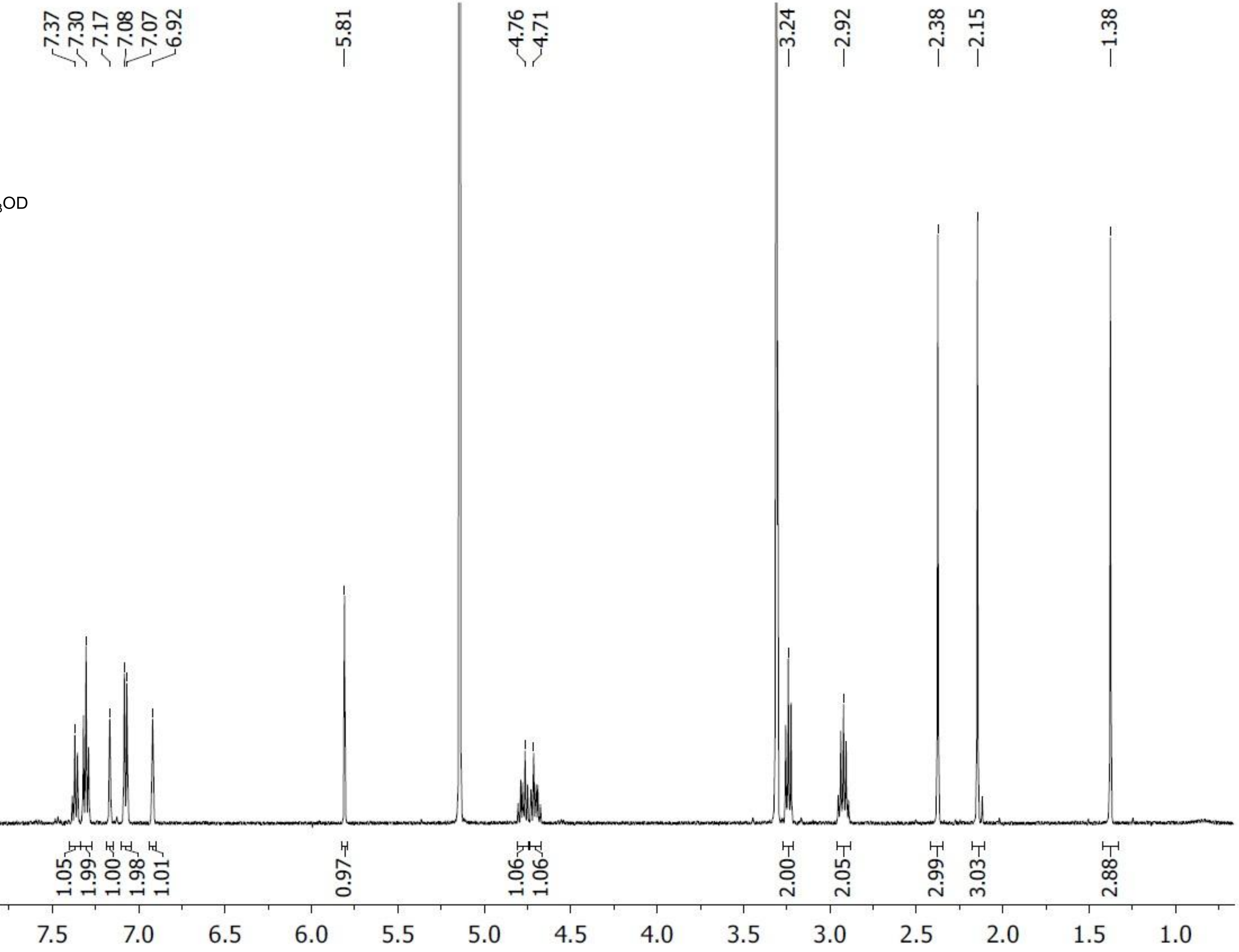

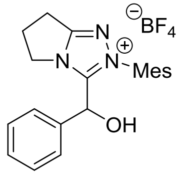

22

$^{13}C\{^1H\}$ NMR, 125 MHz, CD_3OD

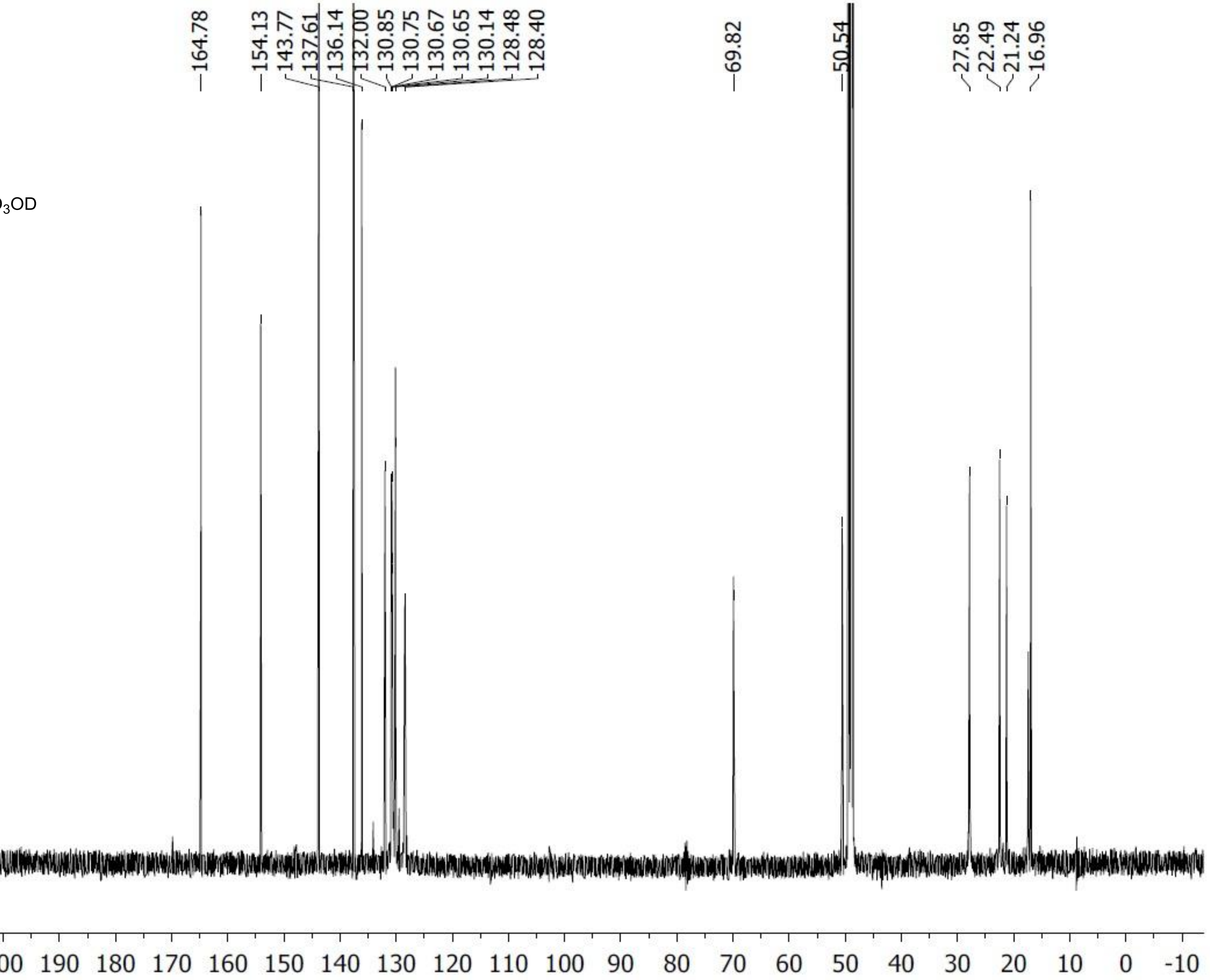

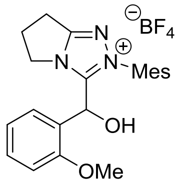

23

^{1}H NMR, 400 MHz, CD_2Cl_2

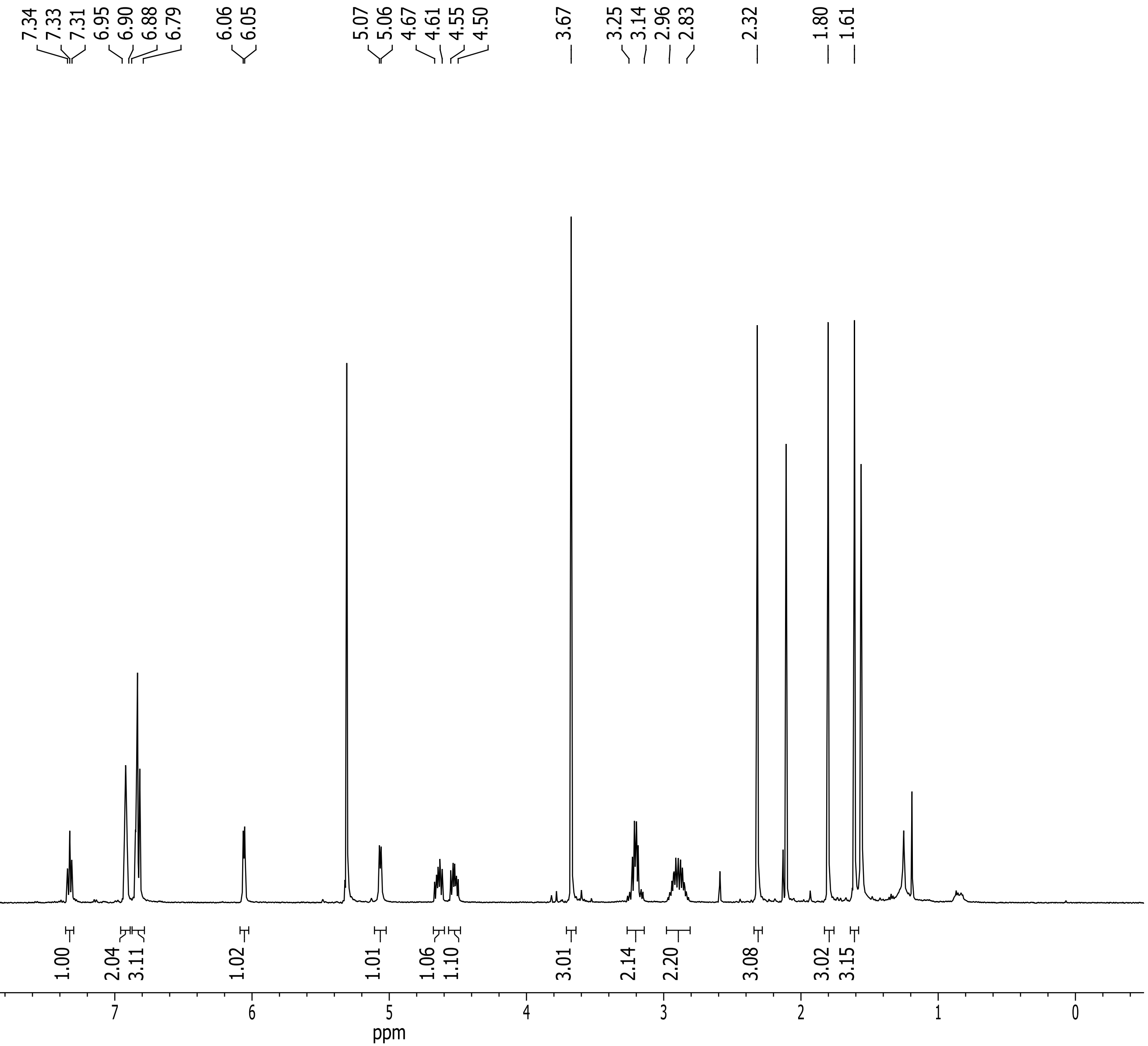

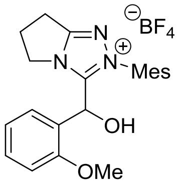

23

$^{13}C\{^{1}H\}$ NMR, 125 MHz, CD_2Cl_2

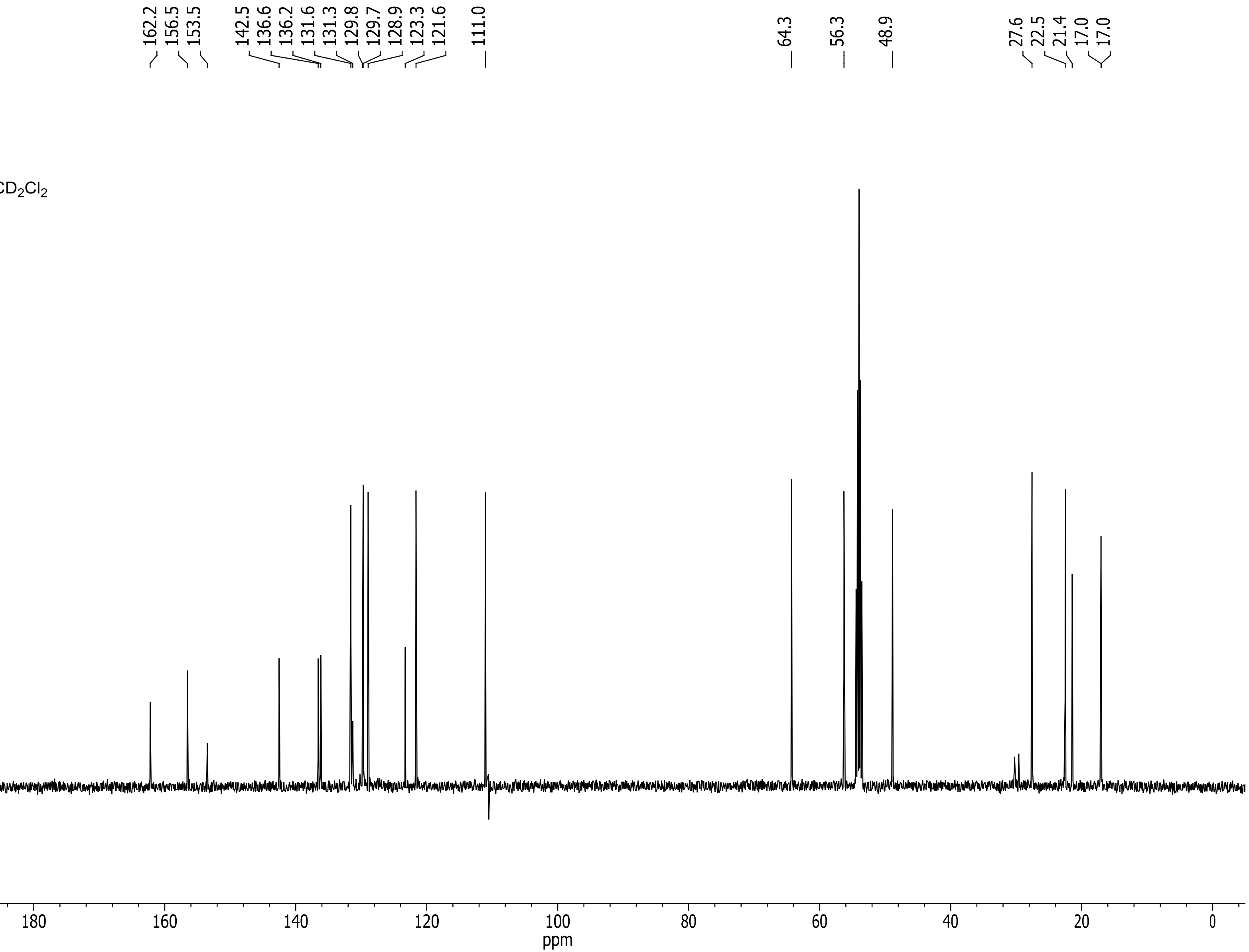

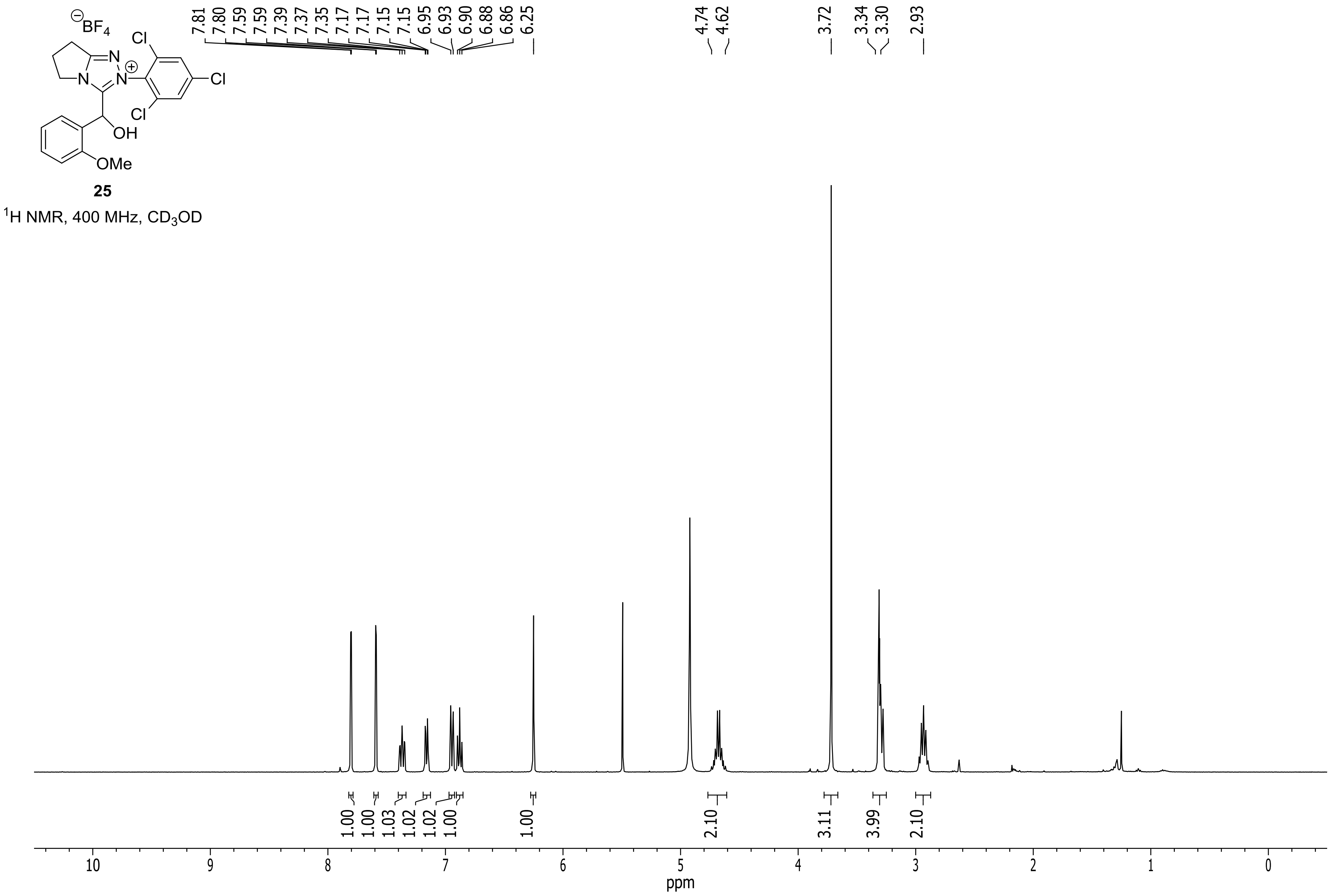
BF4
Cl
N
N
N
Cl
Cl
OH
OMe
25
1H NMR, 400 MHz, CD3OD
7.81
7.80
7.59
7.59
7.39
7.37
7.35
7.17
7.17
7.15
7.15
6.95
6.93
6.90
6.88
6.86
6.25
4.74
4.62
3.72
3.34
3.30
2.93
1.00
1.00
1.03
1.02
1.02
1.00
1.00
2.10
3.11
3.99
2.10
10
9
8
7
6
5
4
3
2
1
0
ppm

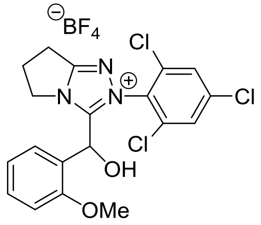

25

$^{13}C\{^1H\}$ NMR, 100 MHz, CD_3OD

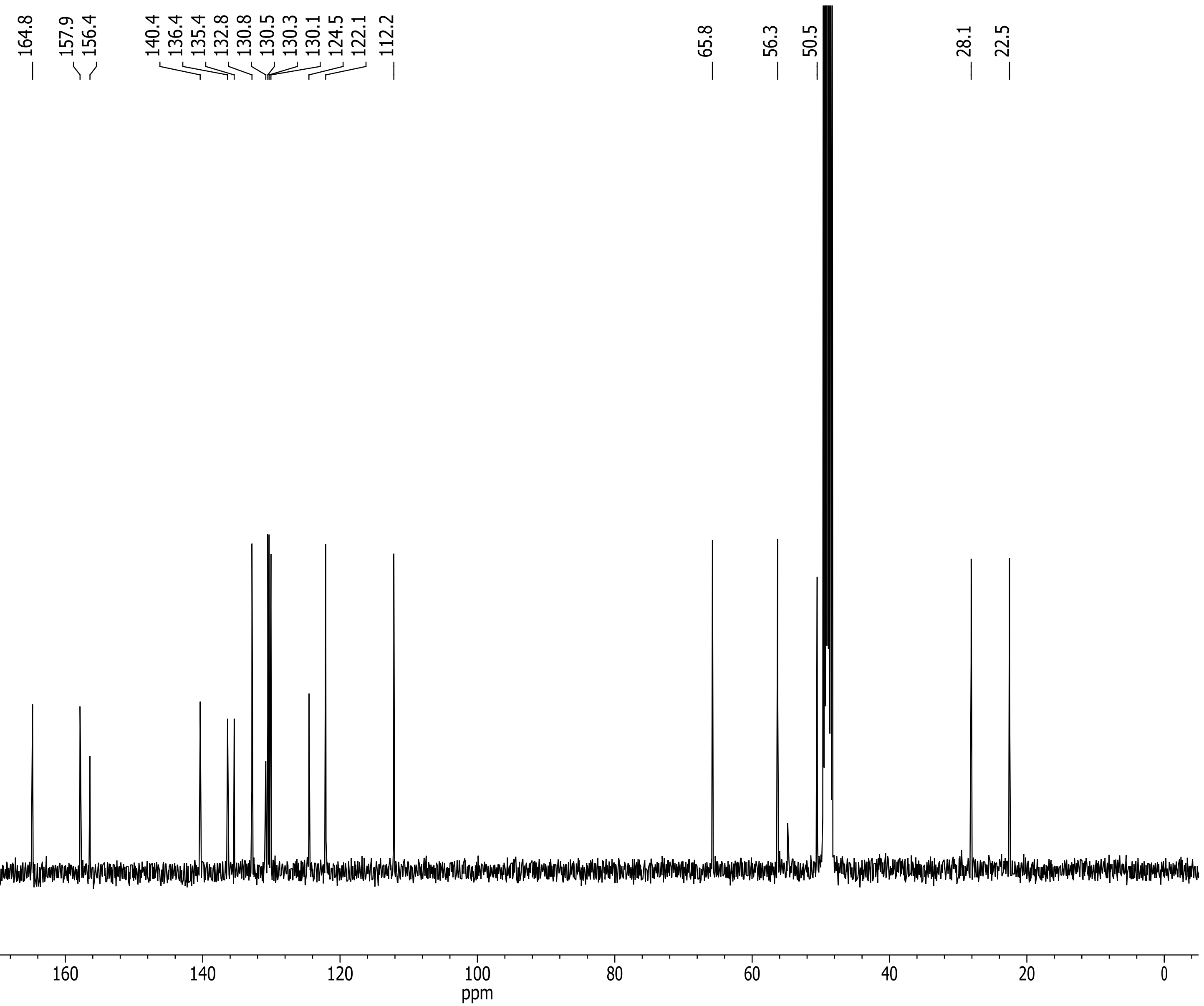

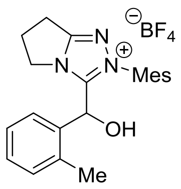

26

^{1}H NMR, 500 MHz, CD_2Cl_2

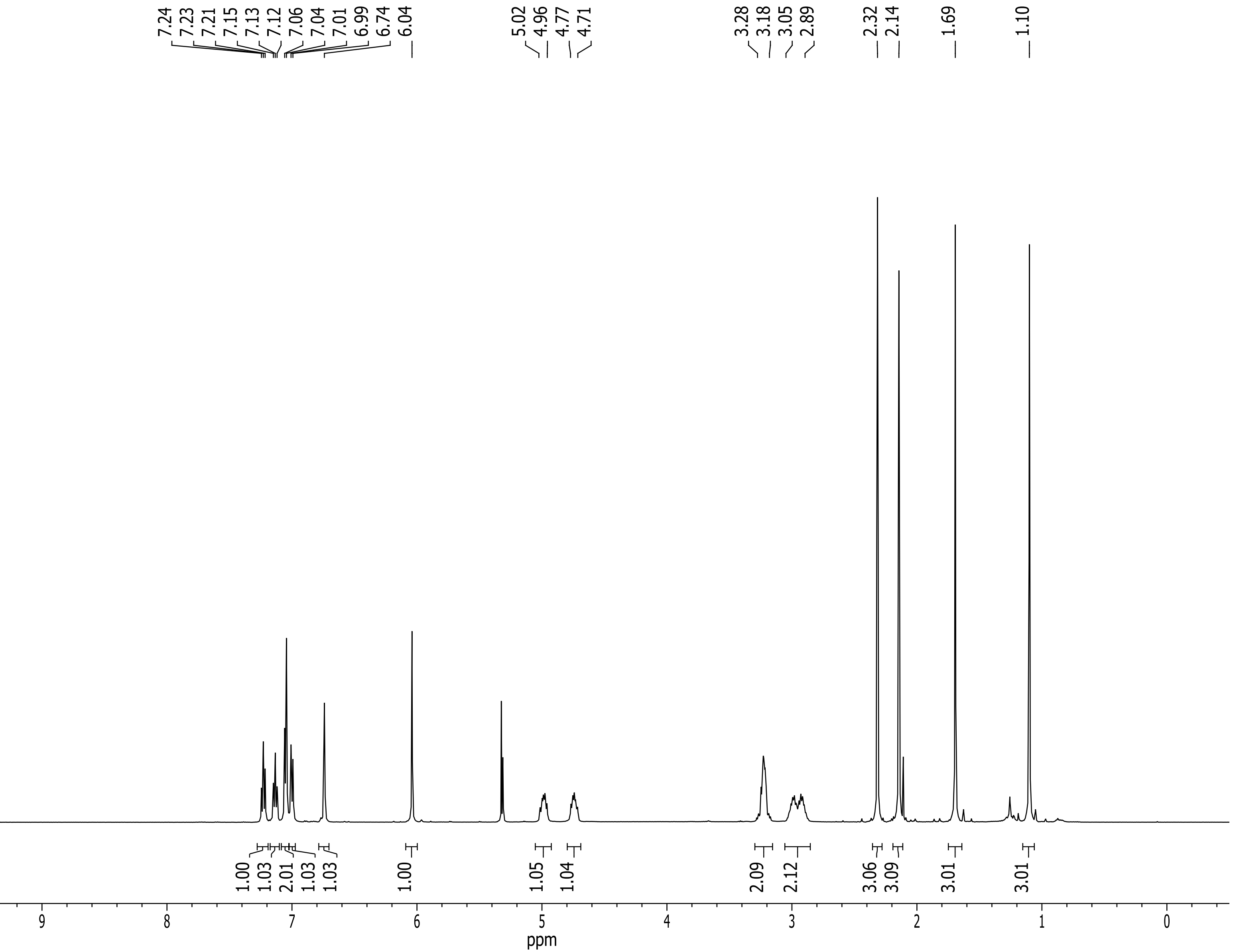

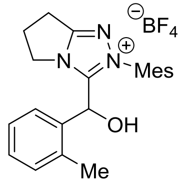

26

$^{13}C\{^{1}H\}$ NMR, 125 MHz, CD_2Cl_2

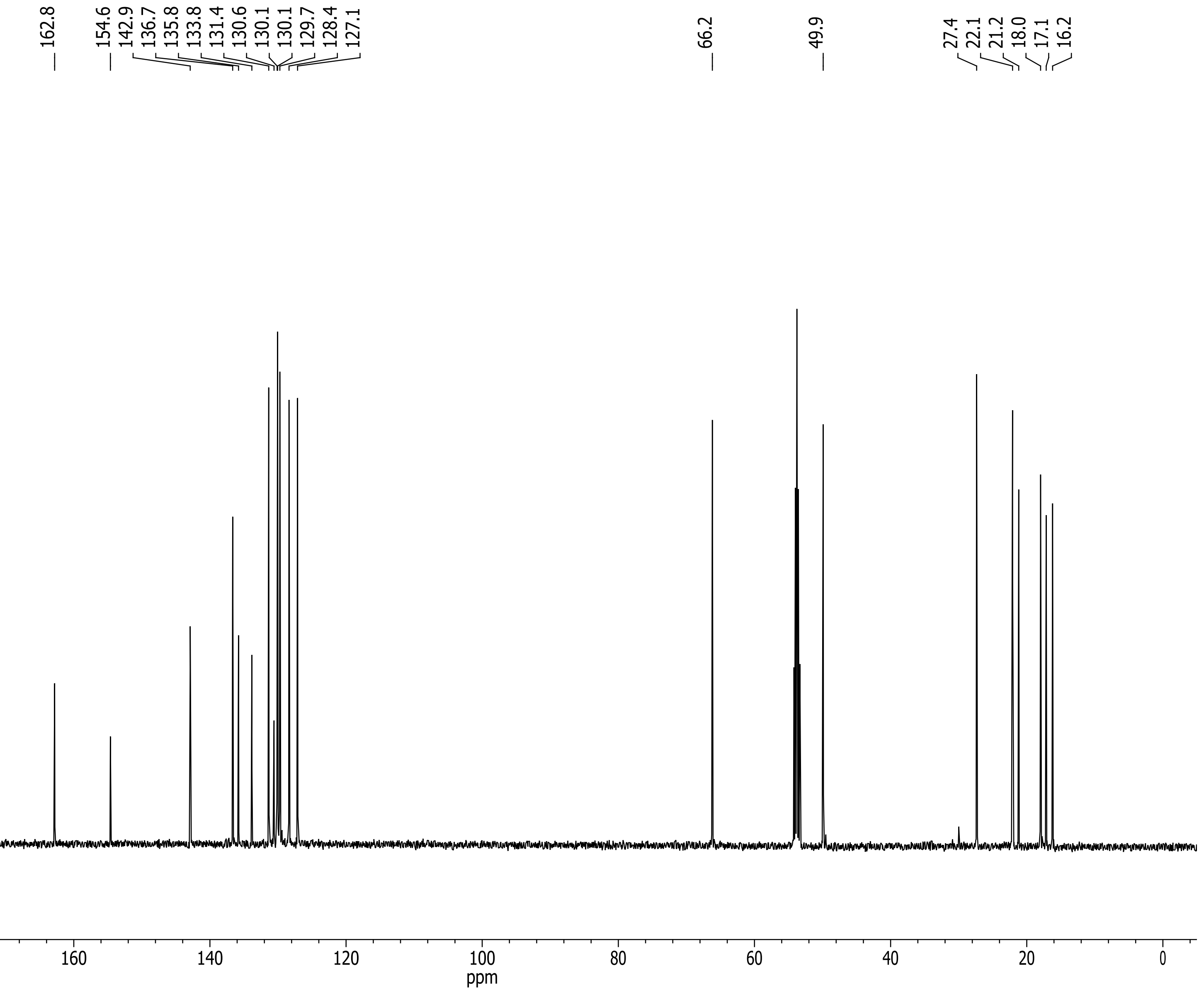

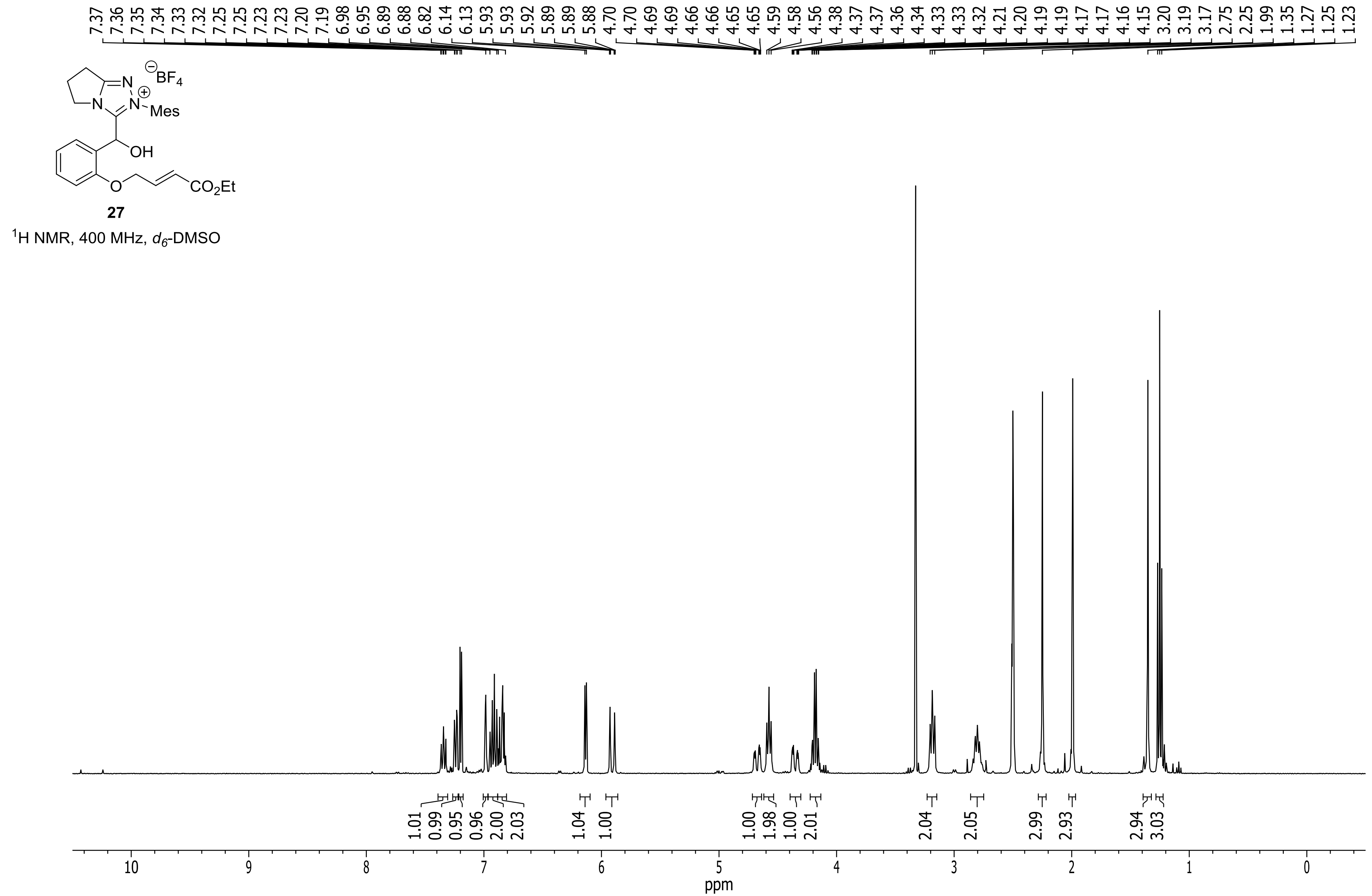
BF4
N
N
Mes
N
OH
O
CO2Et
27
1H NMR, 400 MHz, d6-DMSO
ppm

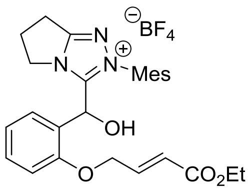

27

$^{13}C\{^1H\}$ NMR, 100 MHz, d_6-DMSO

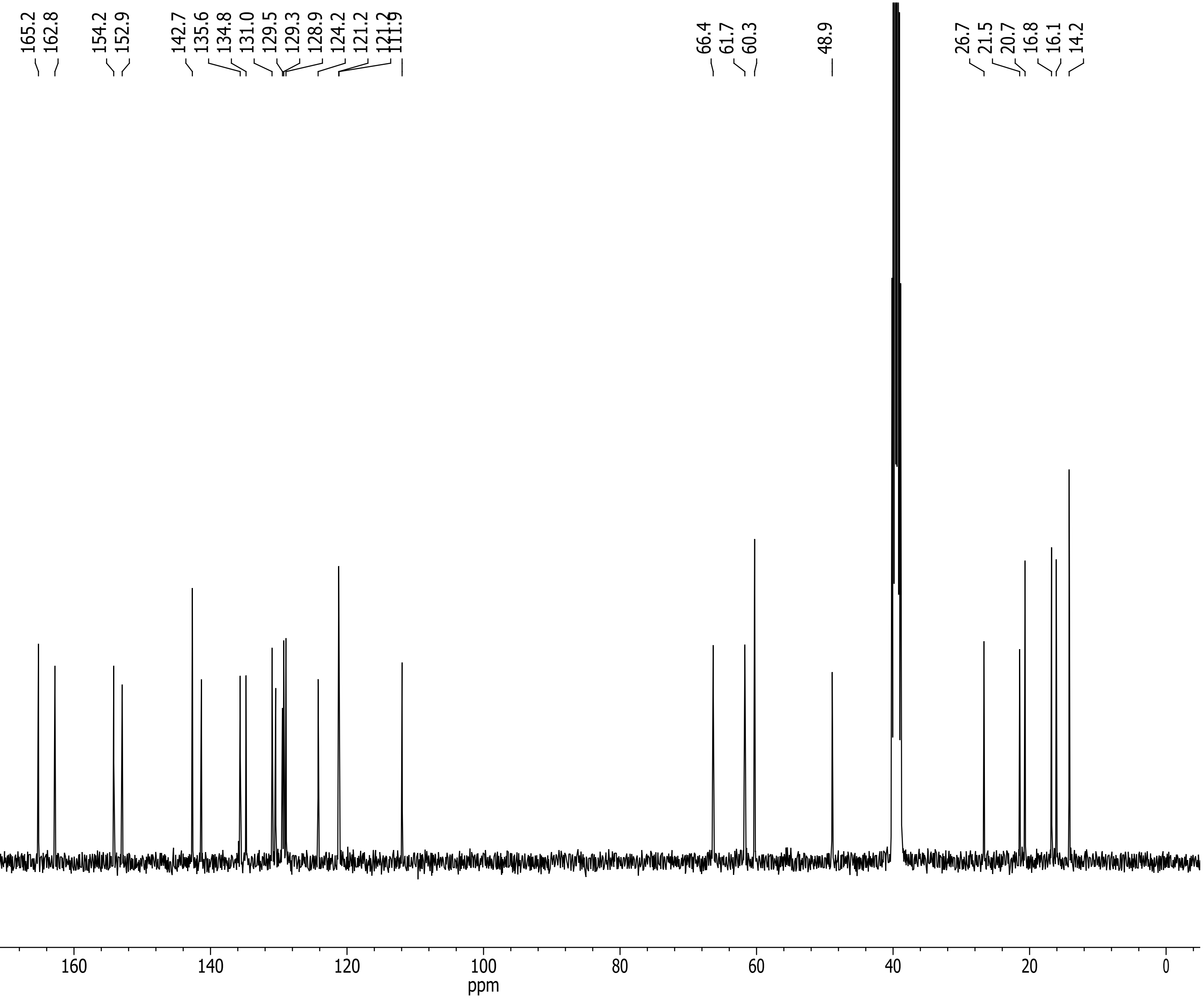

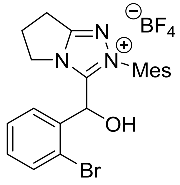

28

^{1}H NMR, 500 MHz, CD_2Cl_2

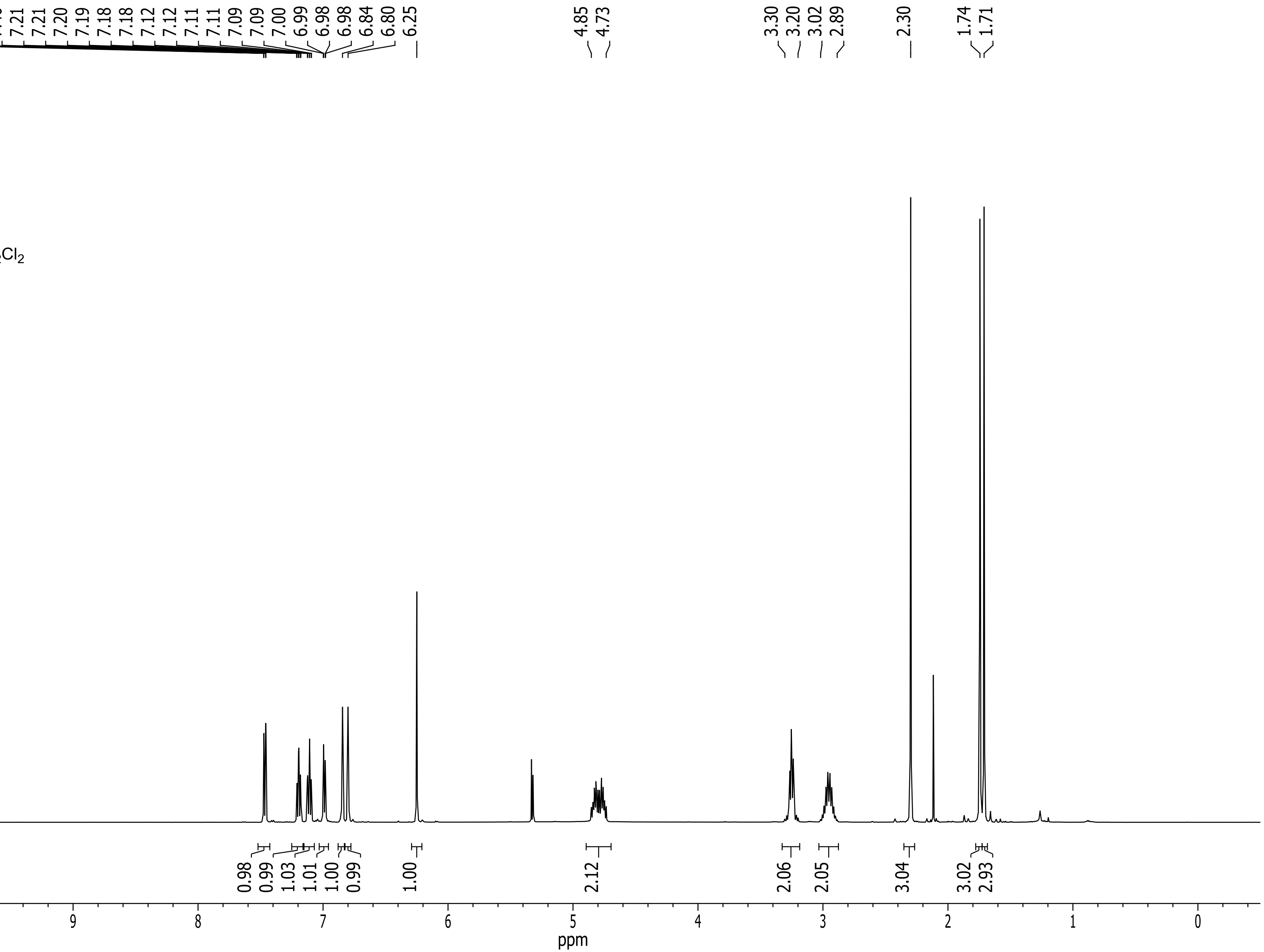

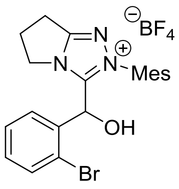

28

$^{13}C\{^{1}H\}$ NMR, 125 MHz, CD_2Cl_2

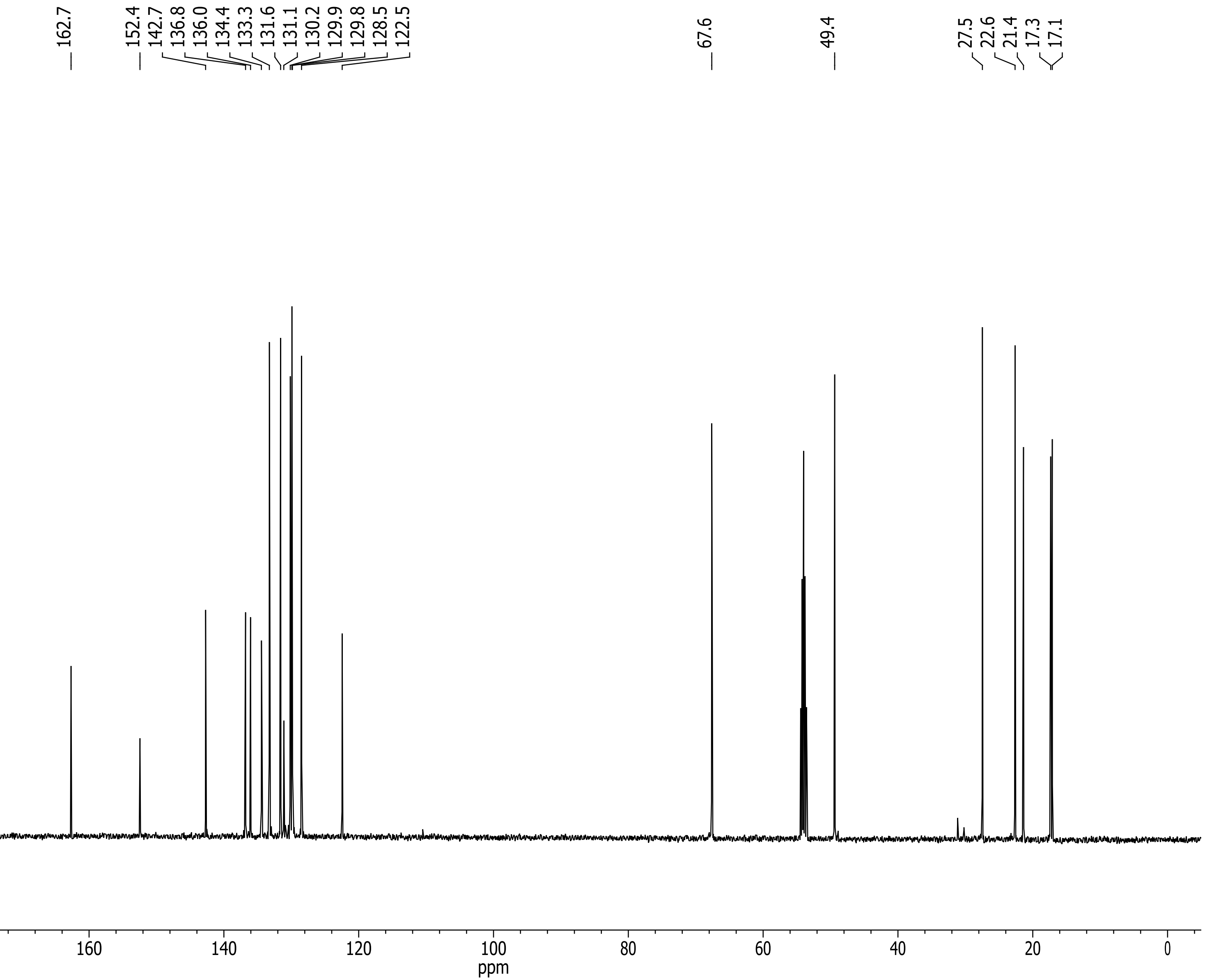

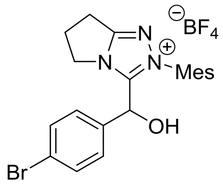

29

^{1}H NMR, 500 MHz, CD_3OD

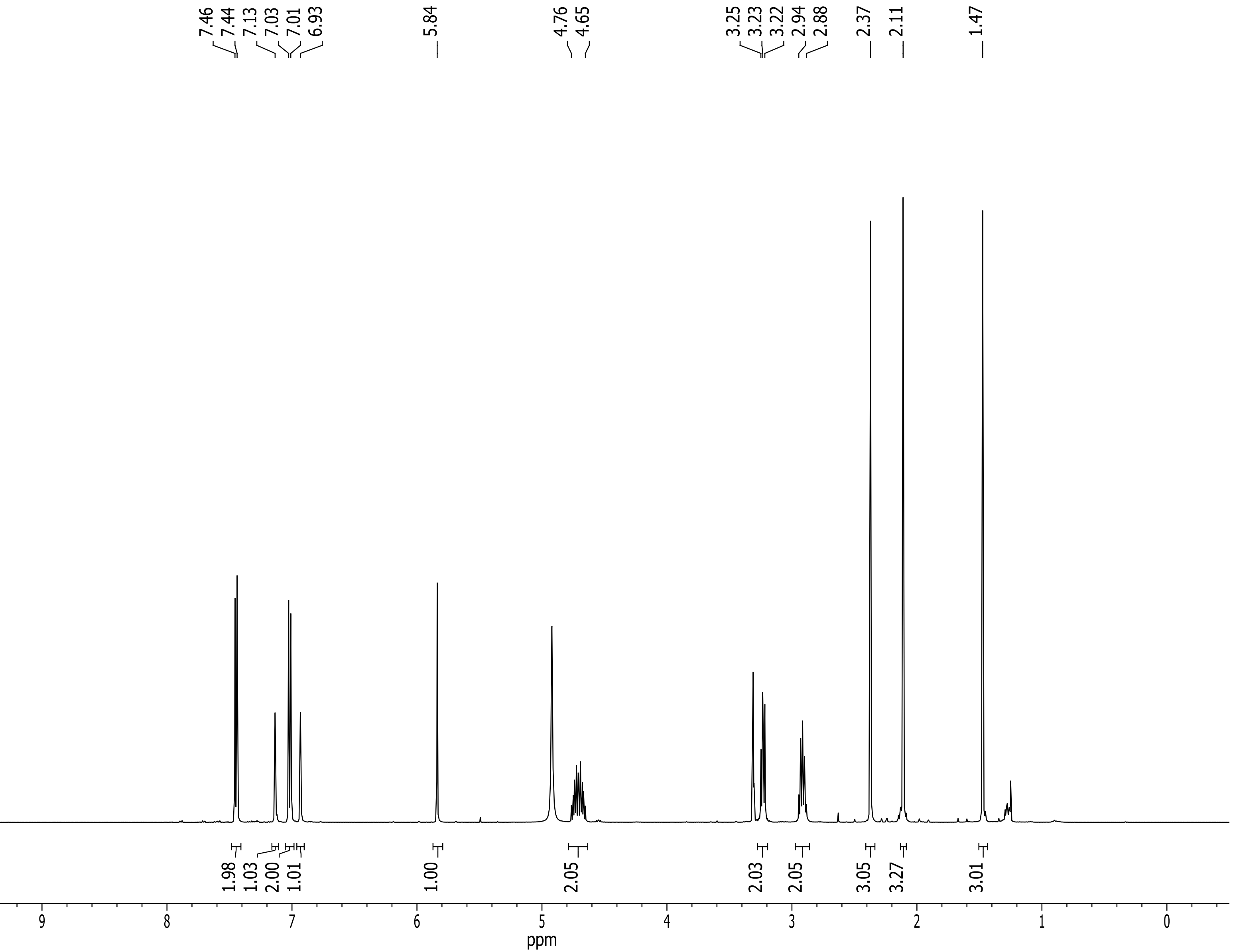

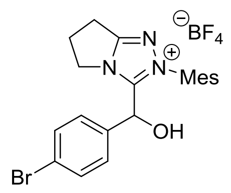

29

$^{13}C\{^{1}H\}$ NMR, 125 MHz, CD_3OD

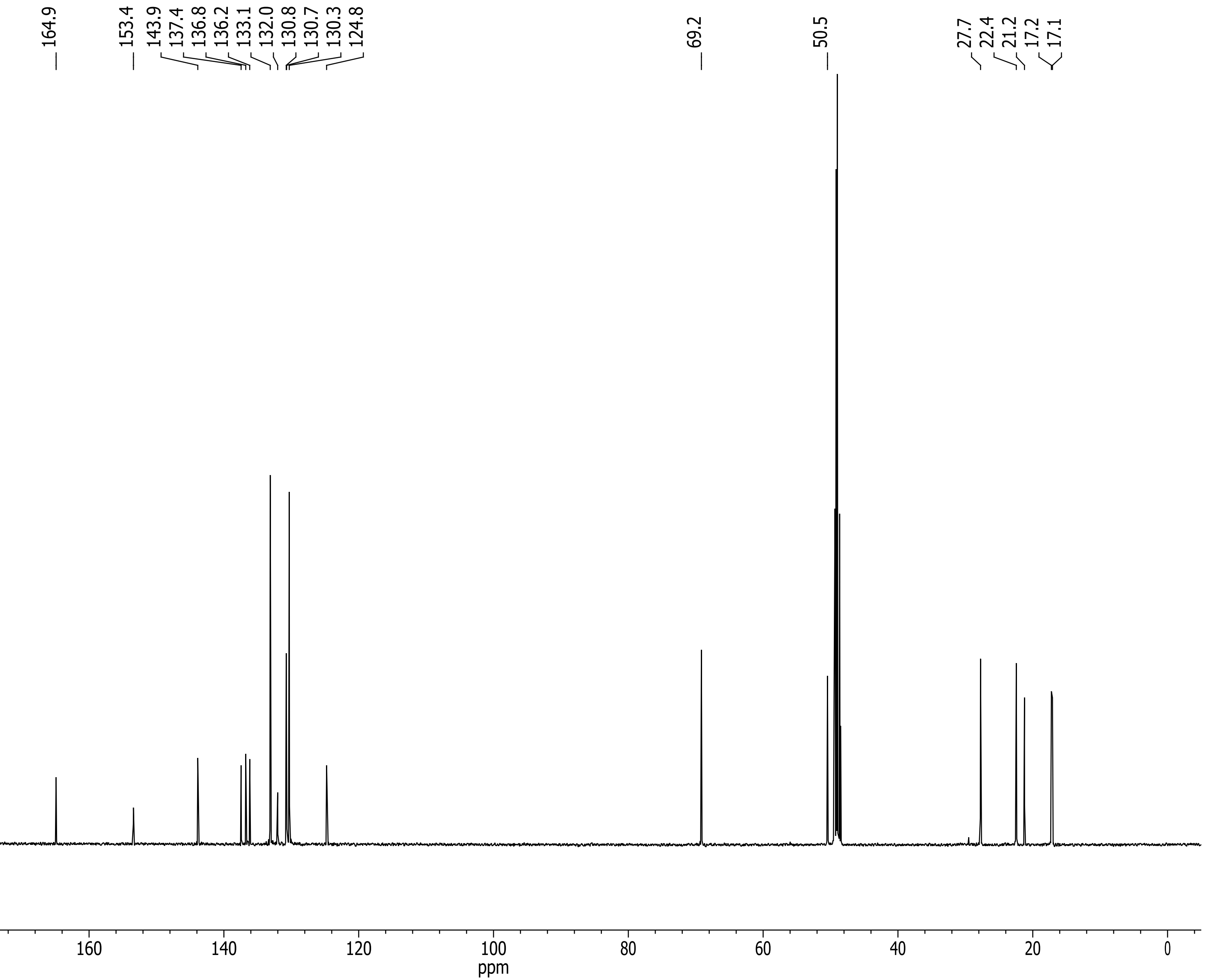

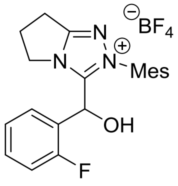

30

^{1}H NMR, 500 MHz, CD_2Cl_2

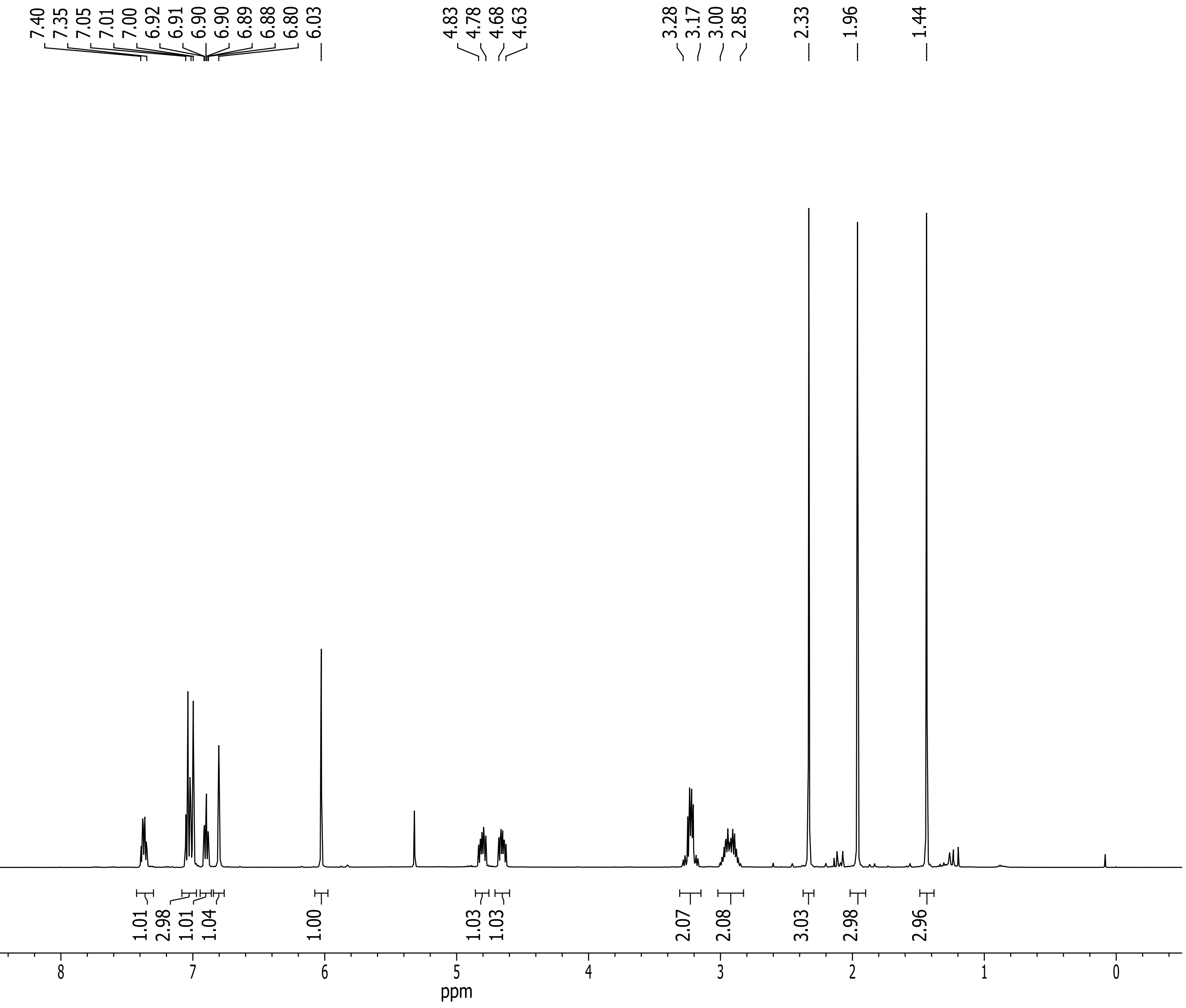

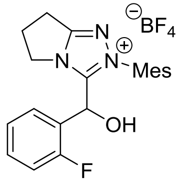

30

$^{19}F\{^1H\}$ NMR, 470 MHz, CD_2Cl_2

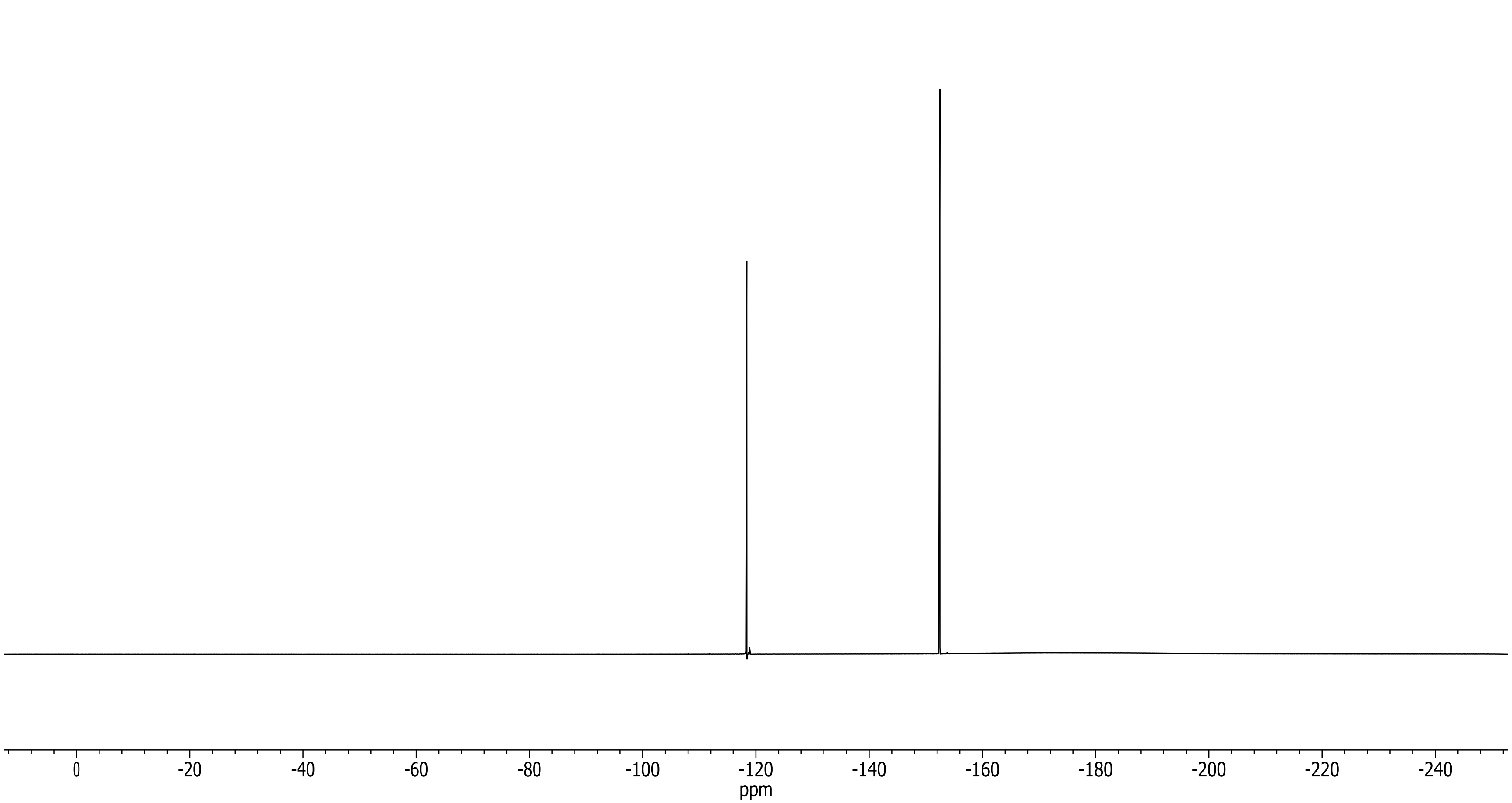

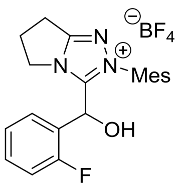

30

$^{13}C\{^{1}H\}$ NMR, 125 MHz, CD_2Cl_2

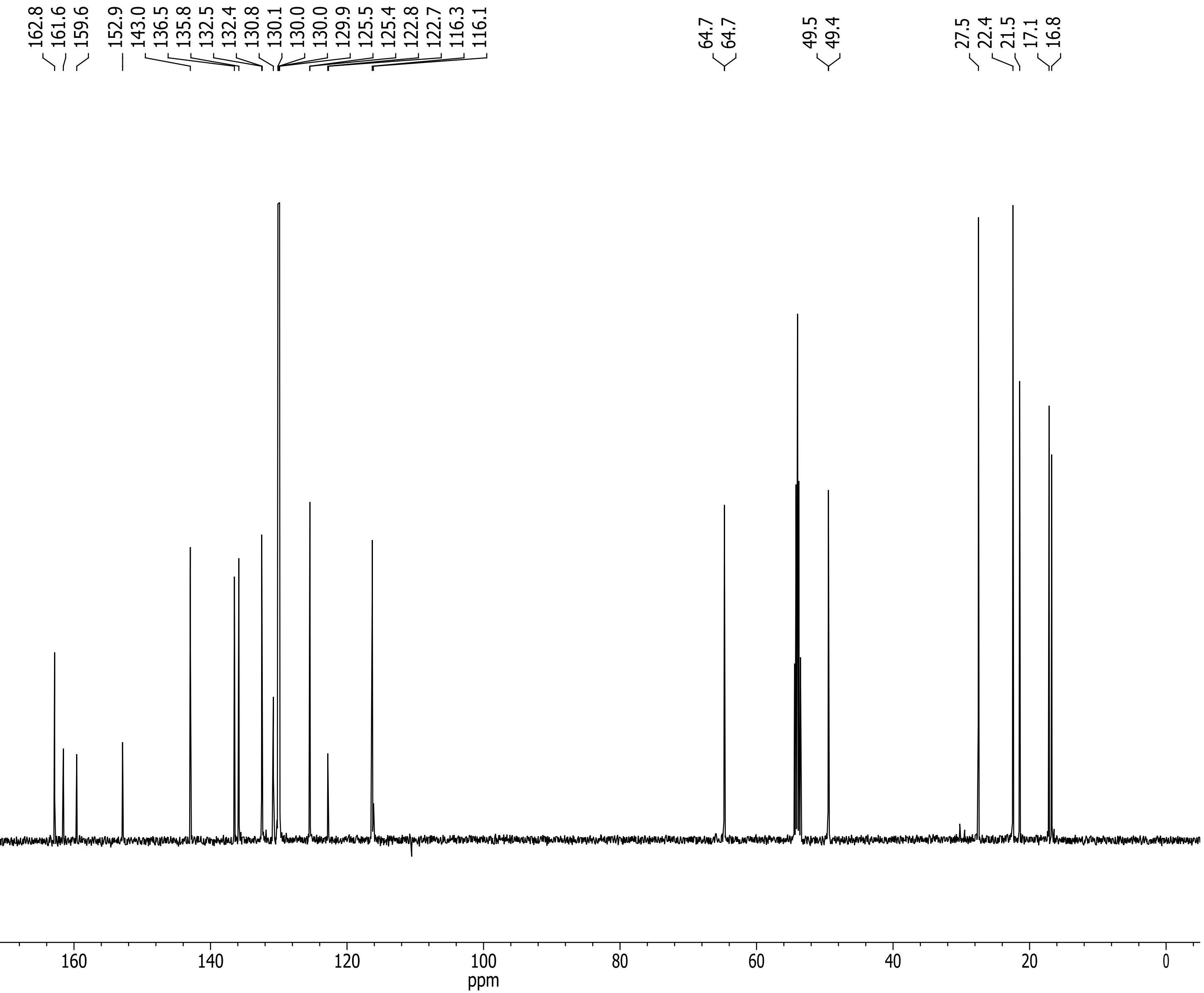

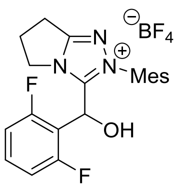

31

^{1}H NMR, 500 MHz, CD_2Cl_2

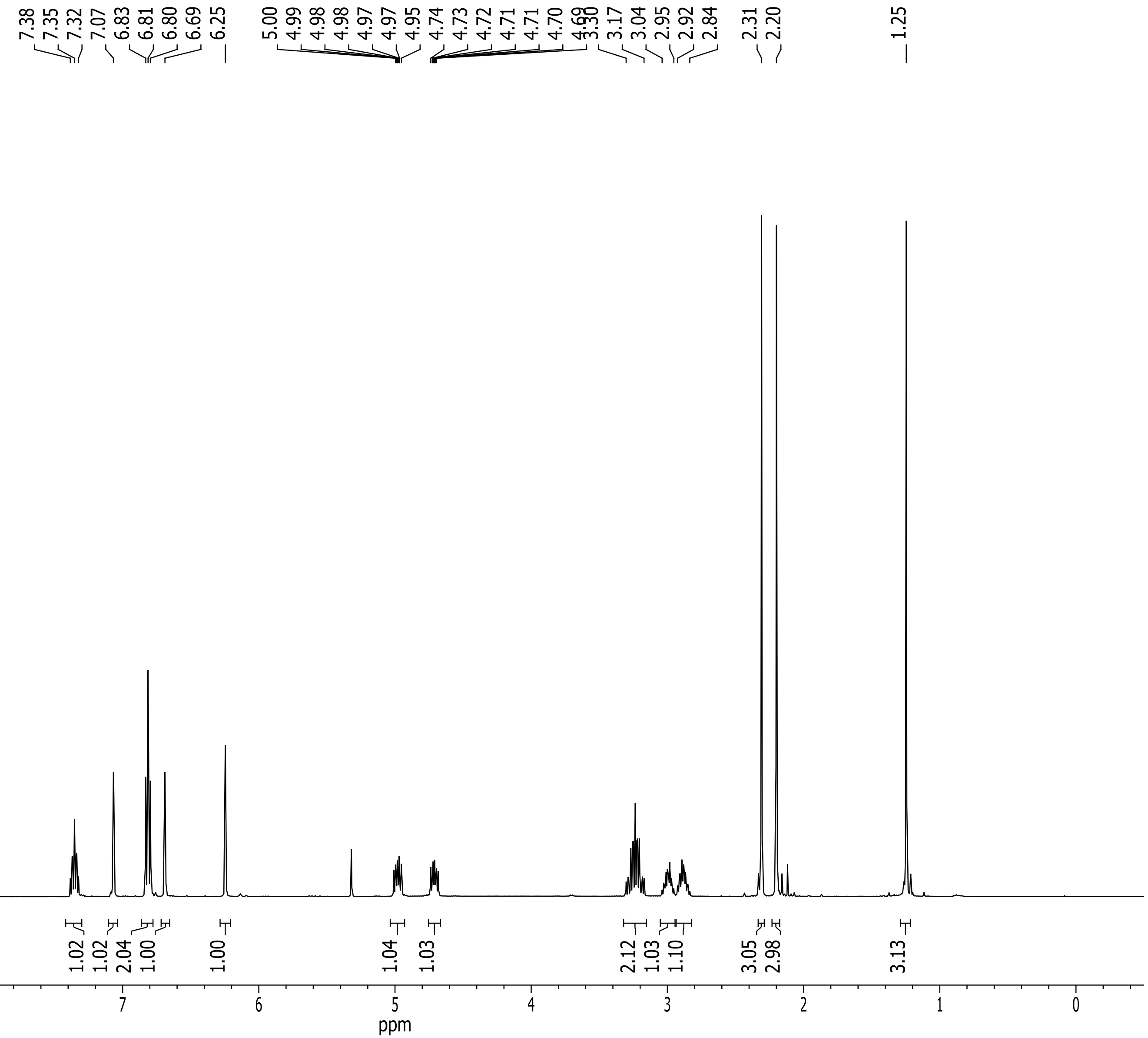

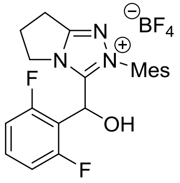

31

$^{19}F\{^1H\}$ NMR, 470 MHz, CD_2Cl_2

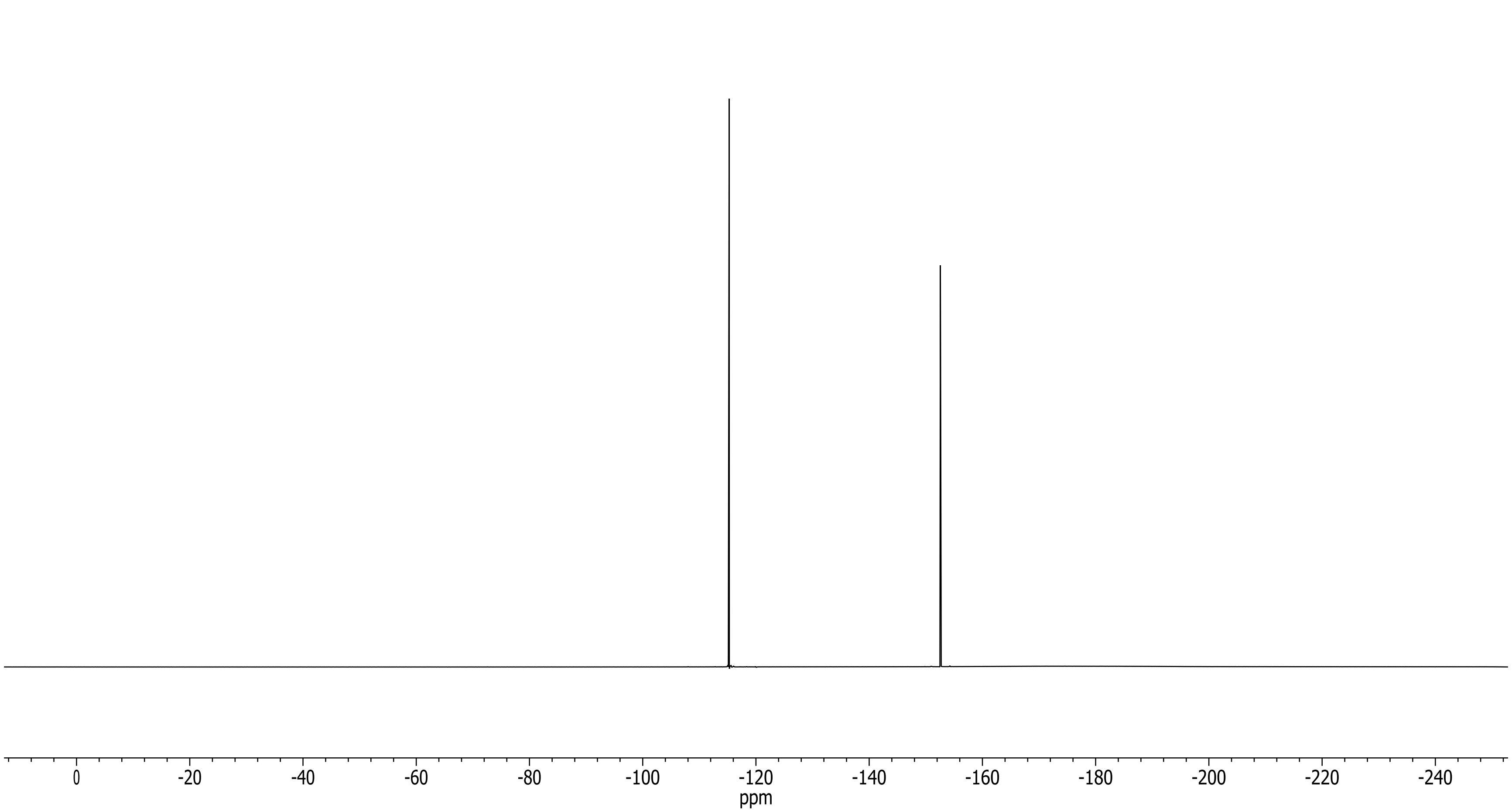

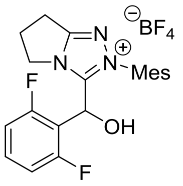

31

$^{13}C\{^{1}H\}$ NMR, 125 MHz, CD_2Cl_2

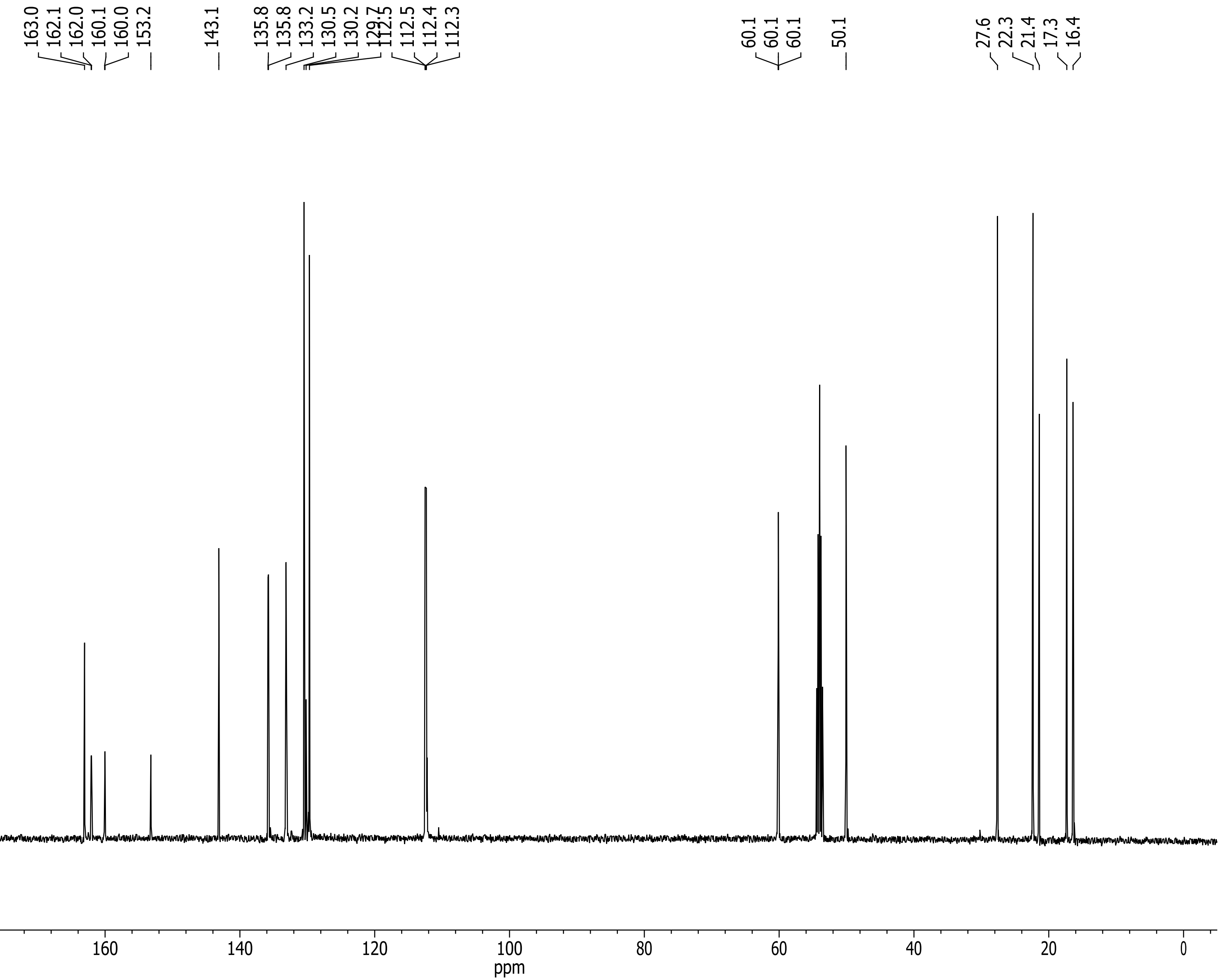

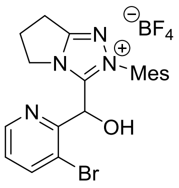

33

^{1}H NMR, 400 MHz, CD_2Cl_2

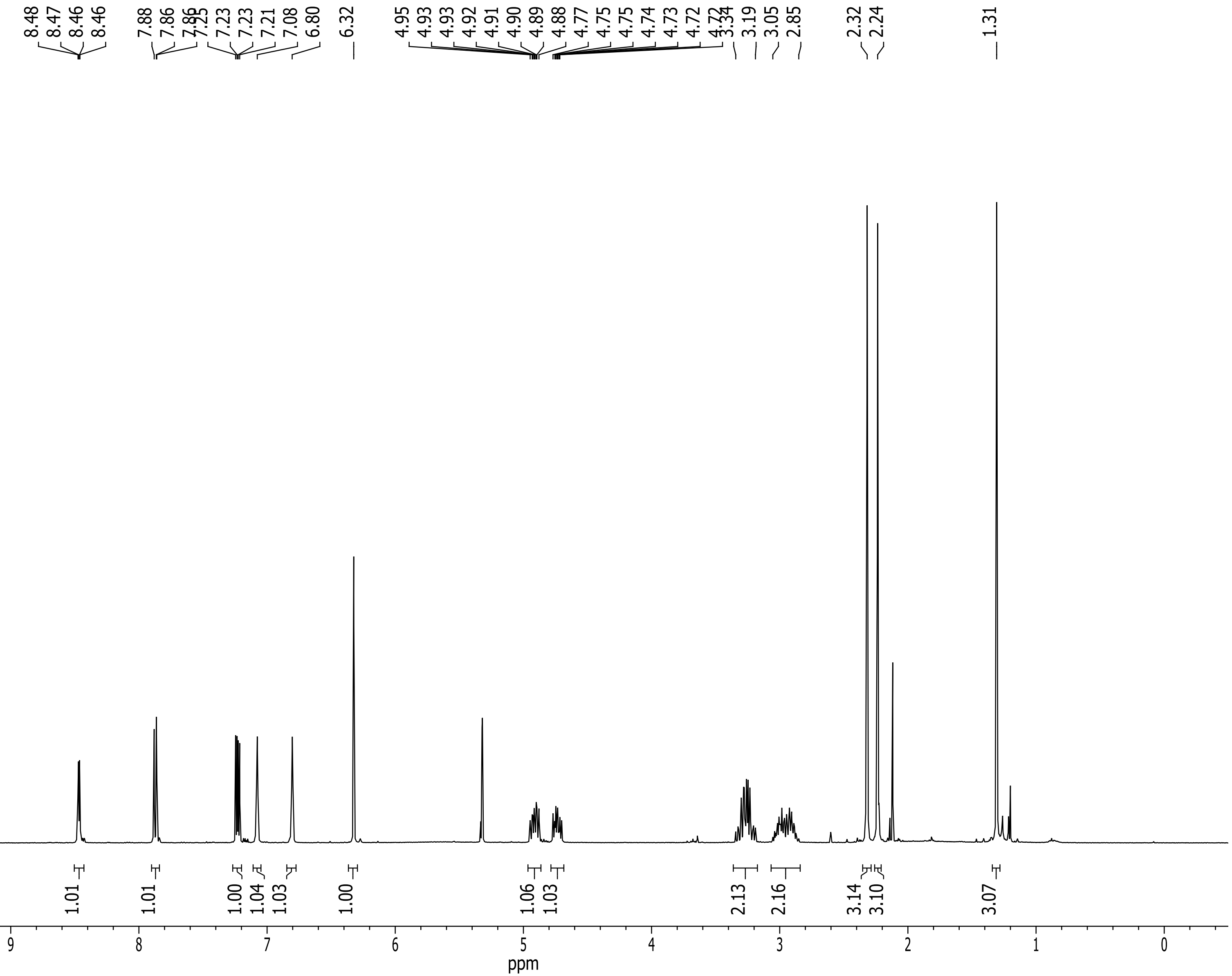

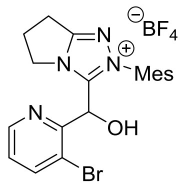

33

$^{13}C\{^{1}H\}$ NMR, 100 MHz, CD_2Cl_2

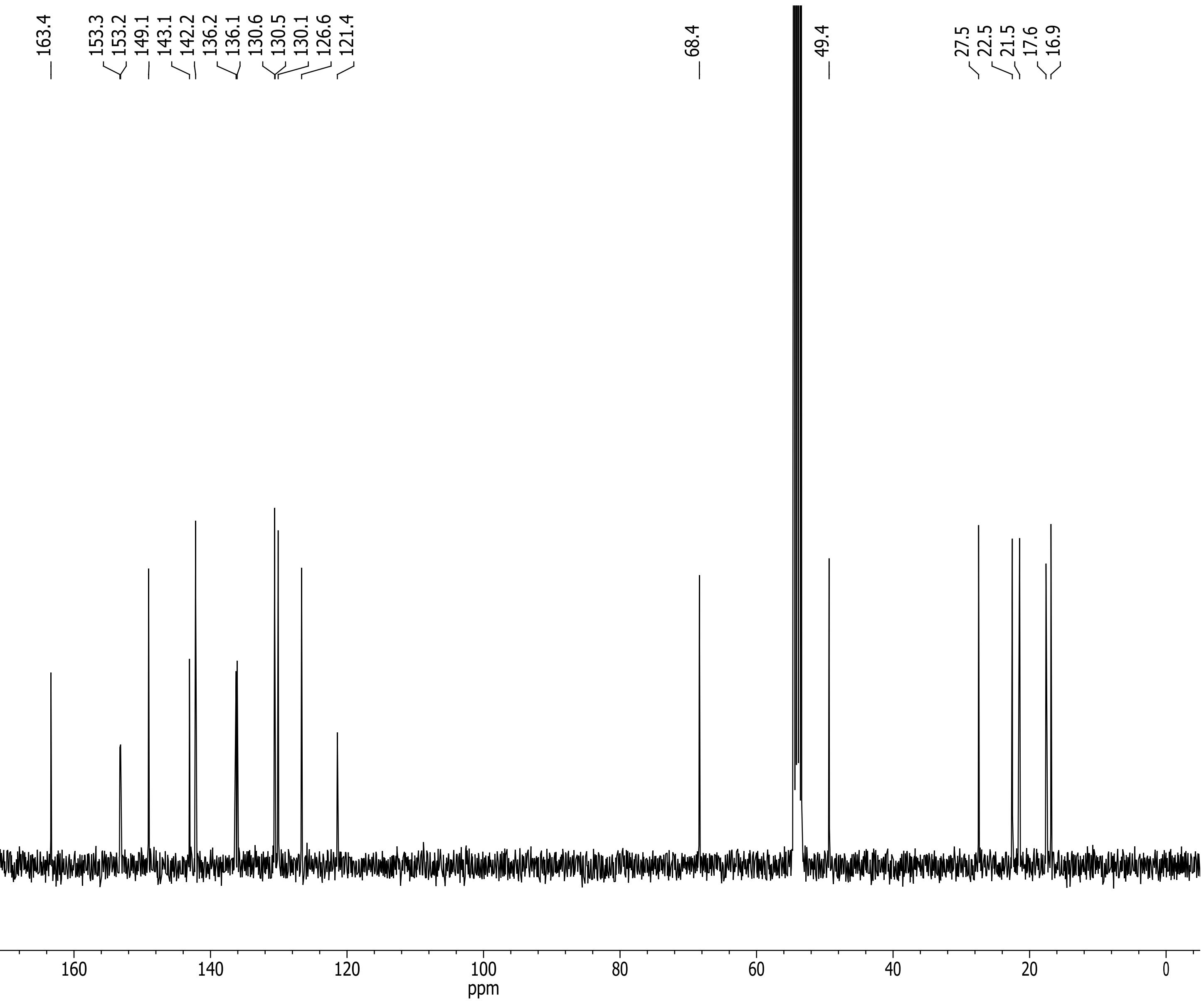

Supplement: Supplementary file 1 [file anie0054-6887-sd1.pdf]
